# Integrative network analyses of transcriptomics data reveal potential drug targets for acute radiation syndrome

**DOI:** 10.1038/s41598-021-85044-5

**Published:** 2021-03-10

**Authors:** Robert Moore, Bhanwar Lal Puniya, Robert Powers, Chittibabu Guda, Kenneth W. Bayles, David B. Berkowitz, Tomáš Helikar

**Affiliations:** 1 Department of Biochemistry, University of Nebraska-Lincoln, Lincoln, NE USA; 2 Department of Chemistry, University of Nebraska-Lincoln, Lincoln, NE USA; 3 Department of Genetics, Cell Biology & Anatomy, University of Nebraska Medical Center, Omaha, NE USA; 4 Department of Pathology and Microbiology, University of Nebraska Medical Center, Omaha, NE USA

**Keywords:** Computational biology and bioinformatics, Functional clustering, Target identification, Transcriptomics

## Abstract

Recent political unrest has highlighted the importance of understanding the short- and long-term effects of gamma-radiation exposure on human health and survivability. In this regard, effective treatment for acute radiation syndrome (ARS) is a necessity in cases of nuclear disasters. Here, we propose 20 therapeutic targets for ARS identified using a systematic approach that integrates gene coexpression networks obtained under radiation treatment in humans and mice, drug databases, disease-gene association, radiation-induced differential gene expression, and literature mining. By selecting gene targets with existing drugs, we identified potential candidates for drug repurposing. Eight of these genes (BRD4, NFKBIA, CDKN1A, TFPI, MMP9, CBR1, ZAP70, IDH3B) were confirmed through literature to have shown radioprotective effect upon perturbation. This study provided a new perspective for the treatment of ARS using systems-level gene associations integrated with multiple biological information. The identified genes might provide high confidence drug target candidates for potential drug repurposing for ARS.

## Introduction

Given the increased capacity of countries to produce enormous radioactive catastrophe and the heightened tensions within the political climate, treatment, and prevention of Acute Radiation Syndrome (ARS) is paramount. ARS is an understudied disease that describes whole body exposure to high doses of radiation (> 0.7 Gy) in a short period of time^1^. The pathophysiology of ARS is characterized by nausea, vomiting, and diarrhea^1^. Additionally, exposure of (0.7–2 Gy) irradiation leads to a depletion of lymphocytes, granulocytes, and hepatocytes^1,2^. The progression of the disease generally follows through three clinically distinct phases. Nausea, headaches, diarrhea, fever, altered consciousness, and fatigue characterize the potential outcomes of the initial—prodromal—response phase^2^. During the second, latent phase, the patient displays no symptoms of ARS. The third stage is the manifest illness phase, where the symptoms of ARS become apparent. There are currently three existing FDA approved treatments for ARS Neupogen, Neulasta, and Leukine^3^. However, these approved drugs are not perfect. Additional treatment options show some disease-mitigating properties. For instance, the treatment of mice by captopril, an angiotensin-converting enzyme (ACE) inhibitor, showed increased survival at thirty days post whole body irradiation^4^. Additional studies have shown the efficacy of Insulin-Like Growth factor 1 in mitigating the deleterious effects of radiation on mice populations^5^. However, these treatment methods only address symptoms of ARS and require strict dosing protocols to acquire adequate efficacy. Thus, additional treatment options are necessary to more effectively challenge ARS.

However, ARS is an understudied disease, and the discovery of new treatments faces significant combinatorial complexity. To deal with this complexity new treatment options can be elucidated using systems- and network-based approaches that can help identify critical regulatory genes within the disease state. Weighted Gene Coexpression Network Analysis (WGCNA)^6^ can be used to characterize modules of correlated genes in gene regulatory networks. Genes in these modules have high levels of coexpression with a central eigen gene, which represents the first principal component of a given module. The central eigen genes have been found to be highly correlated to the most connected genes within a module called hubs whose expression levels are representative of highly correlated genes in the same module. These hub genes can be predictive of novel drug targets. For example, such analyses have been used to characterize genes and pathways associated with Alzheimer’s disease^7^, Schizophrenia ^8^, Amyotrophic Lateral Sclerosis^9^, diabetic kidney disease^10^, and to determine potential drug targets in pathogens^11^, and other complex human diseases^12^.

To further address challenges with the discovery and development of treatments for complex diseases, repurposing of existing drugs to new diseases has become a preferred alternative to elucidating novel drugs due to high attrition rate and time. For instance, the FDA-approved radiation countermeasures Neupogen (CSF3R), Neulasta (CSF3R), and Leukine (CSF2RA) are repurposed drugs^3^. Neupogen and Neulasta are used in chemotherapy-induced neutropenia and Leukine is used in bone marrow transplant recipients^13^.

In this study, we used a network-based systems analysis of five transcriptomics datasets obtained under radiation treatment and integrated them with public drug information and disease association. This led us to predict eight targets with repurposable drugs/compounds available.

## Results

**Figure 1:**
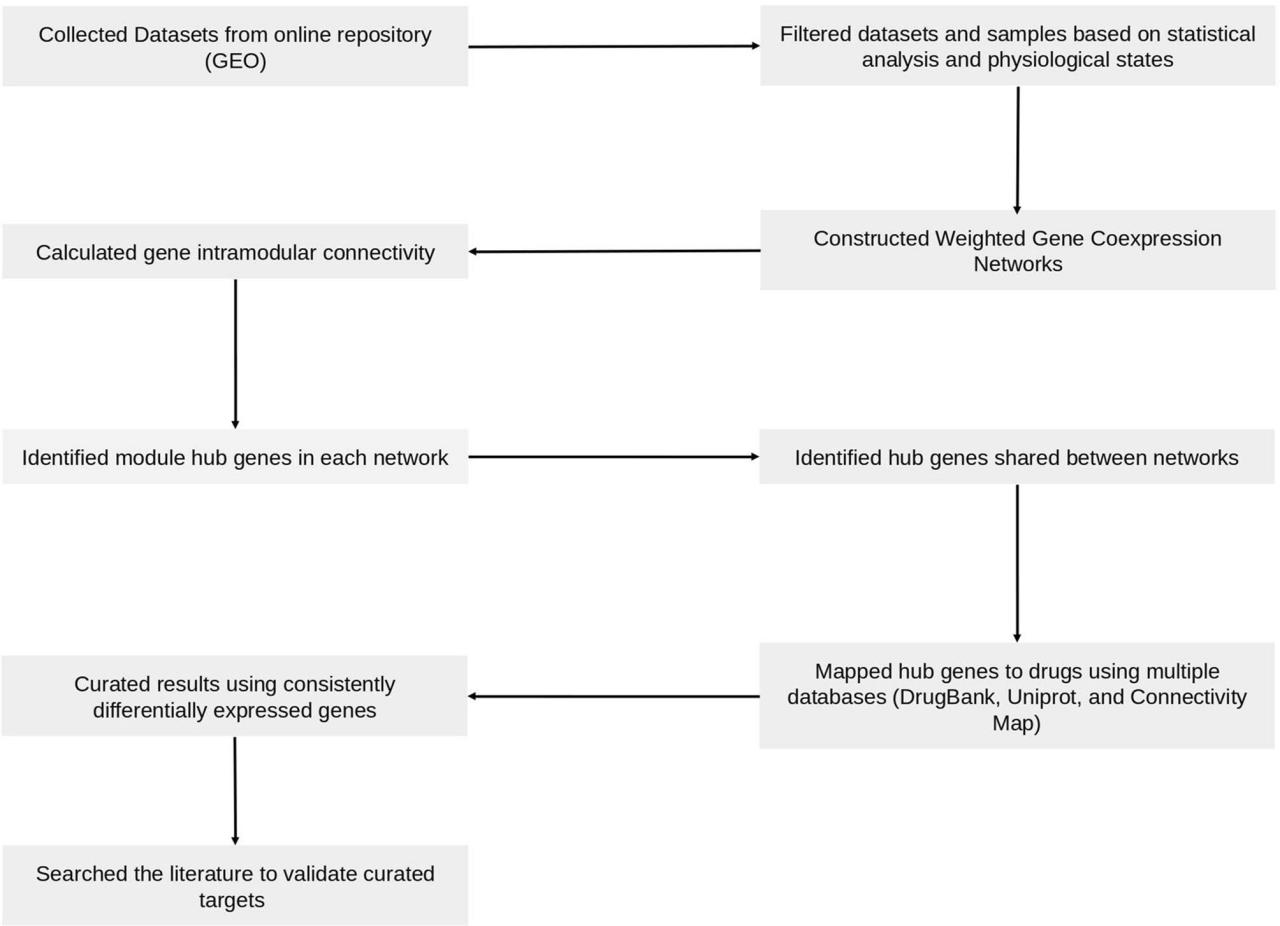
General workflow to construct gene coexpression network to predict potential drug targets.

The workflow used to identify drug targets for ARS is shown in Fig. 1. First, we collected ARS relevant data from public repositories. Second, we constructed gene coexpression networks from transcriptomics data across different species. We used them to identify important genes (hub genes). In the absence of relevant and high-quality human data, we aimed to identify consensus across the available ARS transcriptomics data for different species to maximize the quality of predictions. Using a consensus method increases the likelihood that efficacy in a mouse model would also be observed in human trials, which may minimize the drug development costs. Next, to predict potential drug targets for ARS, these hub genes were mapped with external databases of drugs and diseases, differentially expressed genes under radiation treatments from 6 h to 7 days following radiation of 0.5–10 Gy (different doses and time). The human samples were selected from pre and post-irradiation subjects. These subjects received total body irradiation (TBI) prior to various transplantations. Finally, using literature mining, we predicted the drug targets for ARS.

### Weighted gene coexpression network analysis revealed salient expression networks

We collected five transcriptomics datasets relevant to ARS that were obtained from mice and humans from the GEO database. Using these five datasets, we constructed five weighted gene coexpression networks. A minimum of 28 to a maximum of 169 samples was used for network construction (see Materials and methods). The total number of genes included in these datasets were between ~ 10,000 to ~ 18,000. The scale independence within each network was achieved using a thresholding power (β) parameter. Across all networks, the selected β was ranging from 3 to 20 (Supplementary Data 1). The number of modules (clusters of genes) identified in each network ranged from 5 to 23 (Table 1). Genes in each module in a particular network have the highest topological overlap (i.e., robust measure than correlation). These genes have significant correlations among expression profiles across different doses of radiation or time points after radiation exposure.

**Table 1:** Details of constructed gene coexpression networks under radiation treatments.

Species	Dataset	Number of samples	Total number of modules (sub-networks)	Total number of genes
Mouse	GSE104121	48	5	18,167
	GSE6874	28	10	11,670
	GSE10640	169	22	14,835
Human	GSE6874	70	8	12,845
	GSE10640	60	23	10,918

### Biological function analysis reveals conserved pathways across networks

**Figure 2:**
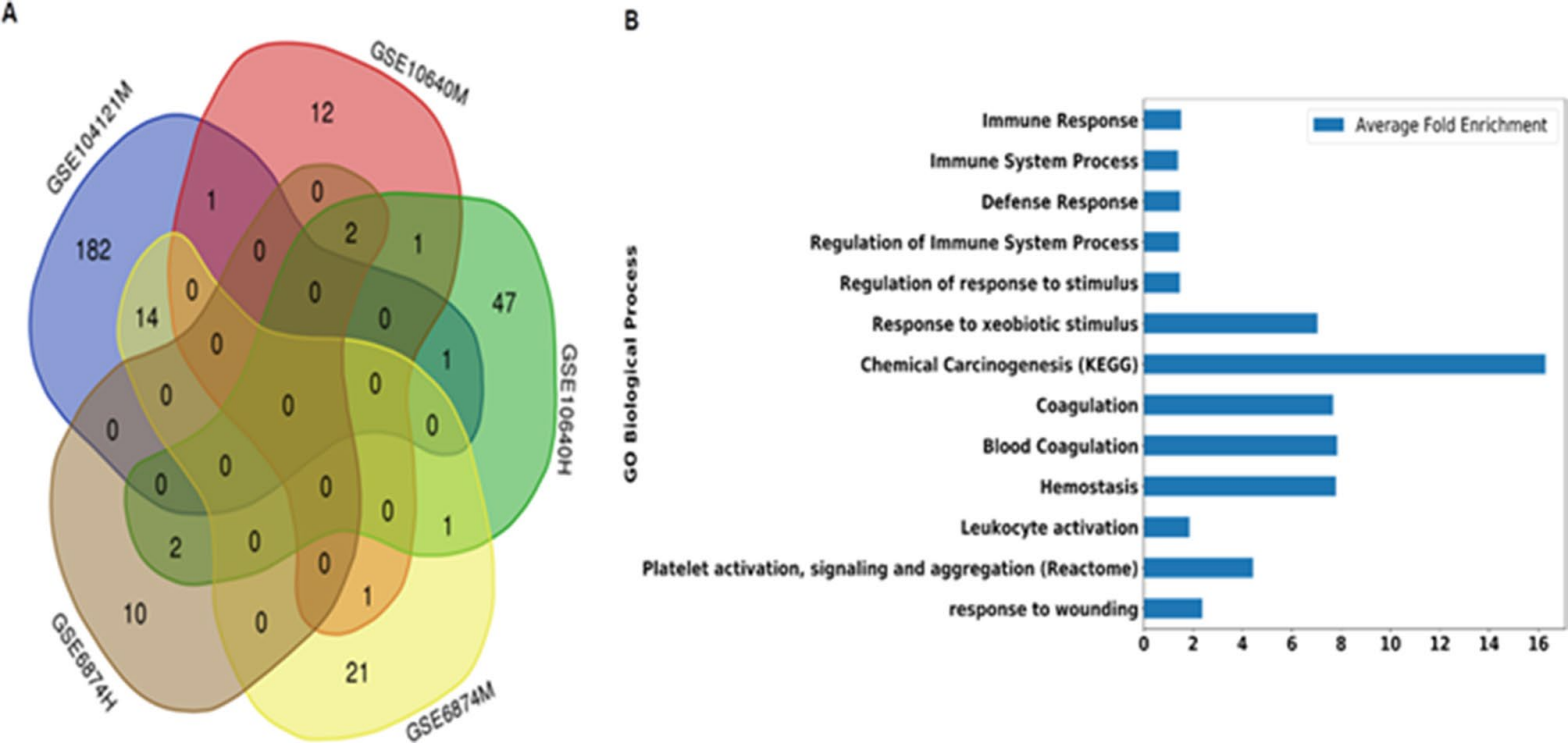
Comparison of enriched biological pathways among different networks. (**A**) Overlap of the Gene Ontology terms (Biological Processes, Molecular Functions, and Cellular Compartments) and pathways (KEGG pathways, Reactome pathways, BioCyc) in each network based on 5% False Discovery Rate (FDR). The venn diagram was created using http://bioinformatics.psb.ugent.be/webtools/Venn/14 (**B**) Average fold enrichment of biological processes and pathways that were enriched in more than one network.

In the next step, we found the biological significance of modules identified in the constructed networks. Pathway enrichment analysis showed enrichment with biological processes in at least one module across five datasets (Supplementary Data 2). Next, we identified biological processes that were identified by multiple datasets. Among these, 23 gene ontology (GO), KEGG, and Reactome terms were enriched in more than one dataset (Fig. 2A). These terms included biological processes and pathways related to immune response, xenobiotic stimulus, chemical carcinogenesis, blood coagulation, and white blood cell activation (Fig. 2B) (P-value < 0.05 and False Discovery Rate < 5%). The highest fold enrichment across all the networks (average fold enrichment) was found in chemical carcinogenesis (~ 16 fold), blood coagulation and hemostasis (~ 8 fold), and response to xenobiotic stimuli (~ 7 fold).

### Hub genes found across multiple networks

**Figure 3:**
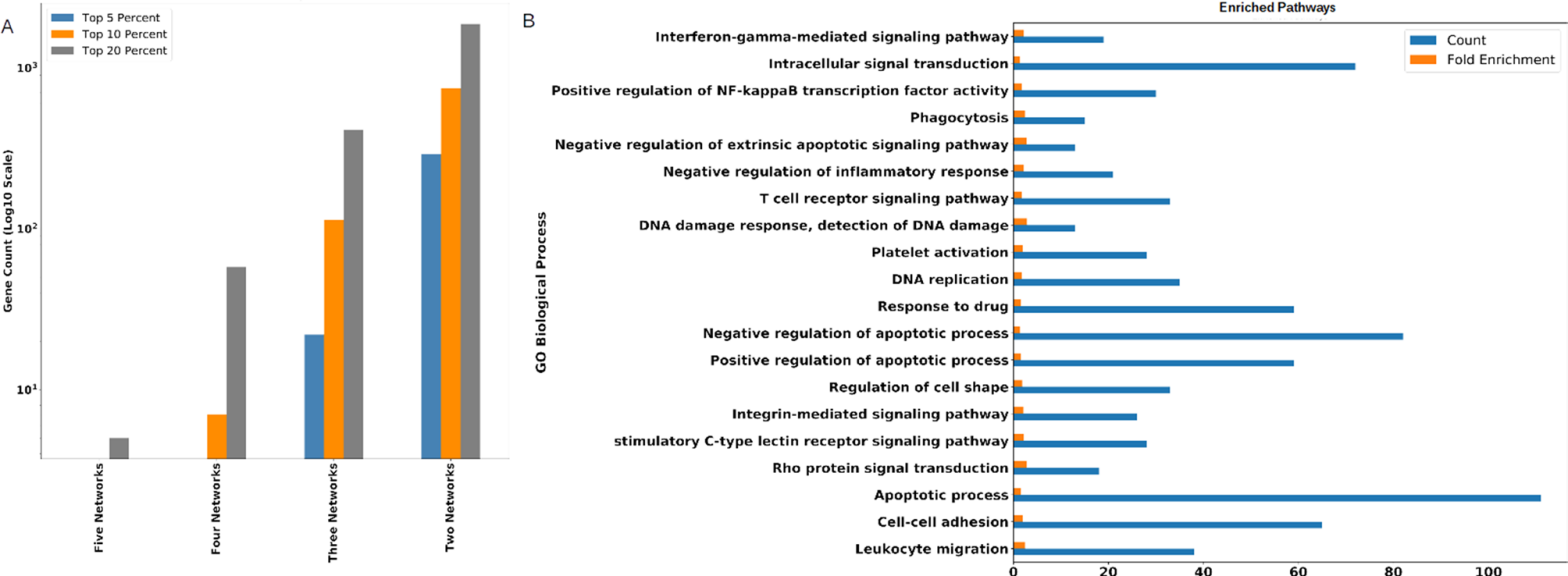
Identification and analysis of hub genes. (**A**) top 5%, top 10%, and top 20% hub genes identified for each dataset that are identified by five, four, three, and two networks. (**B**) GO biological processes enriched in 2,351 hub genes with False Discovery Rate < 5%.

In the constructed networks, intramodular connectivity was used as a measure to identify hub genes (see Materials and methods). Because of the limited data availability of ARS-relevant human data, we identified hub genes that appeared in multiple datasets as high confidence. To select the top hub genes, first, we used criteria of maximum overlap of hub genes across the different datasets. We identified the top 5, 10, and 20 percent hub genes and compared them across all five datasets. In our analysis, the top 20% resulted in more hub genes that were overlapped in the maximum number of datasets (Fig. 3A). Among these, we found five hub genes (Myeloid Cell Nuclear Differentiation Antigen (MNDA), Syntaxin 11 (STX11), intracellular tyrosine kinase (IKT), Slingshot Protein Phosphatase 2 (SSH2), Keratin 9 (KRT9) ) shared by all networks, 58 hub genes shared by four networks, and 412 hub genes that were shared by three networks. On the contrary, in both the top 5% and 10% hub genes, no hub genes were shared by all networks. Four networks shared only seven genes in the top 10% of hub genes, while not a single hub gen was shared by four out of five networks in the top 5%. Thus, the top 20% cutoff allowed optimal comparisons amongst the networks.

Next, among the top 20% hub genes (Supplementary Data 3), we chose genes that were identified within more than one gene coexpression network. This analysis resulted in 2,351 hub genes identified within at least two coexpression networks (Supplementary Data 3). Enrichment analysis of the selected hub genes showed GO biological processes related to leukocyte migration, apoptosis, DNA replication, T cell signaling, NF-κB, and IFN-γ signaling (Fig. 3B).

Furthermore, we have investigated the biological processes that are enriched in differentially expressed in response to high radiation dose (i.e., 10 Gy). Many of the biological processes and pathways related to commonly enriched in multiple networks (Fig. 2B) and in hub genes (Fig. 3B) were also enriched in genes upregulated in high radiation doses. We found the gene ontology terms *regulation of platelet activation* (GO:0,010,543; FDR = 0.035), *blood coagulation* (GO:0,007,596; FDR = 0.027), *leukocyte differentiation* (GO:0,002,521, FDR = 0.007), *response to stimulus* (GO:0,050,896; FDR = 0.0003), *immune systems process* (GO:0,002,376, FDR = 0.0042), *cell–cell adhesion* (GO:0,098,609; FDR = 0.045), *intracellular signal transduction* (GO:0,035,556; FDR = 0.045), and v*esicle-mediated transport* (GO:0,016,192; FDR = 0.003) as enriched in consistently upregulated genes (upregulated in more samples than downregulated). A full table of all enriched biological processes is provided as Supplementary Data 4.

### Network analysis predicts high confidence drug targets for ARS

To identify drug targets for potential drug repurposing, we identified genes that are targets of existing preclinical and FDA-approved drugs (Materials and methods) and then selected genes that were differentially expressed in response to radiation treatments.

**Figure 4:**
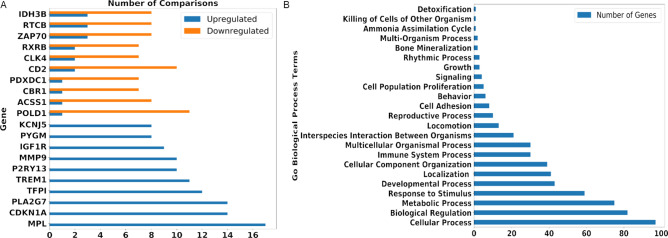
(**A**) Hub genes found in multiple networks with consistent regulation depicted with the count of samples upregulated/downregulated. (**B**) Count of genes in GO Biological processes amongst 101 consistently differentially regulated genes.

Among the identified 2,351 hub genes, 520 genes were mapped with existing preclinical and approved drugs/compounds (see Materials and Methods). Next, the selected hub genes were compared with differentially expressed genes in ARS across 32 conditions (see Materials and Methods). To validate our findings, first, we investigated already known drug targets for ARS that are part of identified hub genes. The gene target CSF3R of two ARS drugs Neulasta and Neupogen appears in our list and they were differentially expressed in 21 different samples (upregulated in 12 samples and downregulated in 9 samples). Next, we investigated all the hub genes for differential expression. Of the 520 genes with existing drugs, 293 genes were differentially expressed in at least eight (25%) of 32 conditions (see Supplementary Data 5). Among these, we identified 81 genes that were consistently up-regulated, and 20 genes that were consistently down-regulated (Fig. 4A, Supplementary Data 5). We then analyzed these 101 differentially expressed hub genes with existing drugs and compounds. These genes include 75 genes involved in (based on Gene Ontology) metabolic processes, 59 genes in response to a stimulus, 30 genes in immune system processes, 5 genes in cell population and proliferation, and 4 genes in cell signaling (Fig. 4B).

**Figure 5:**
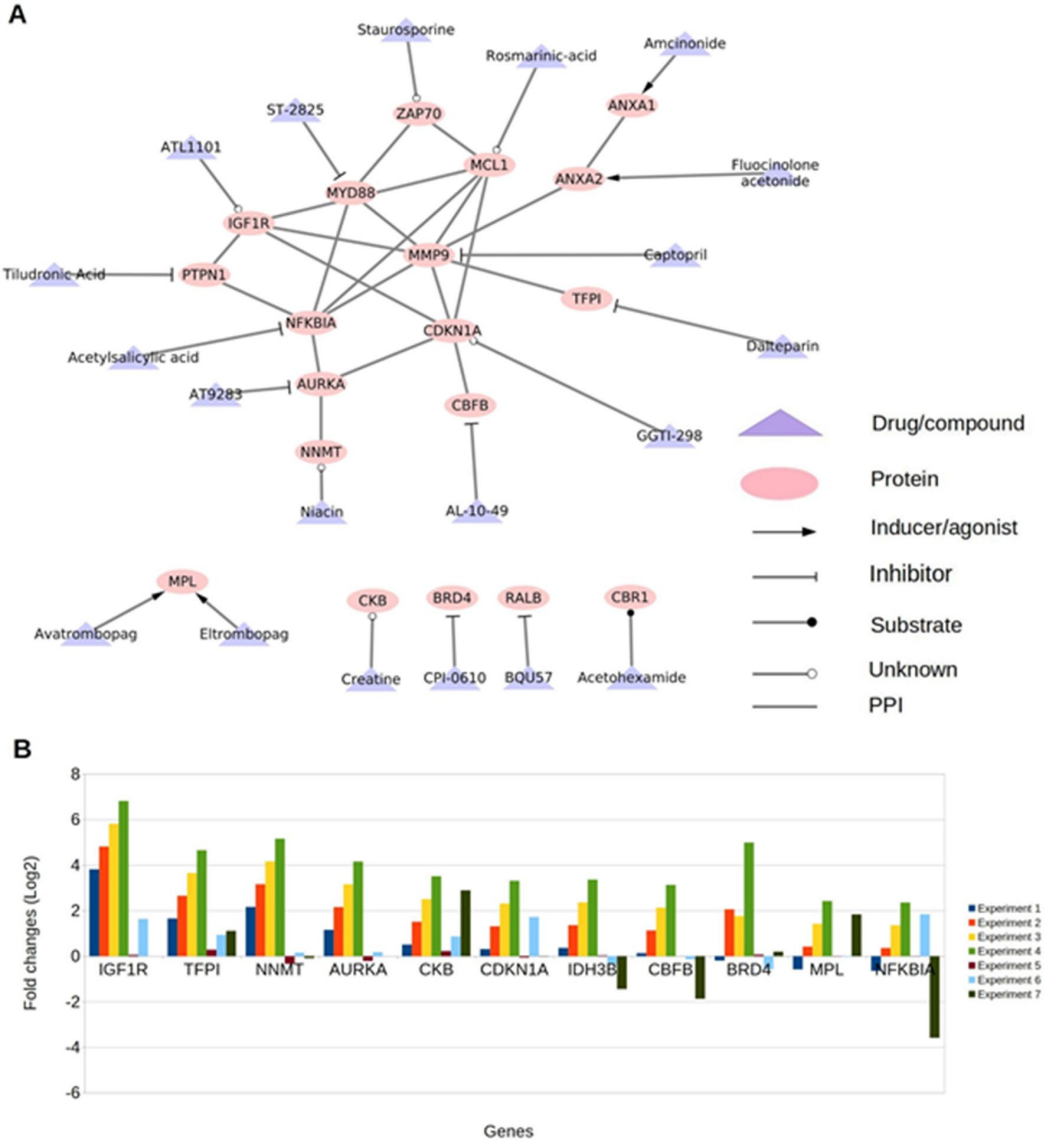
Drug-target network of potential targets and gene expression. (**A**) drug-target network. PPI = protein–protein associations obtained using STRING database.The network was created using Cytoscape version 3.7.2^15^. (**B**) Fold changes of genes showing consistent differential expression of mice in high radiation doses (i.e., 10 Gy and 12 Gy) from GSE10640, GSE6874, and GSE104121. Experiment 1 and 2 were from BLK mice 24 h post-irradiation with 10 Gy, Experiment 3–6 were from BalbC (3,5,6) and BLK (4) 6hrs post-irradiation with 10 Gy, and Experiment 7 was performed on BalbC mice 6hrs post-irradiation with 12 Gy.

Next, literature mining revealed that 20 (of 101) genes were relevant to the ARS (Table 2). Of these 20 genes, we identified eight genes whose response to radiation was incongruent with prognostic favorability (indicated with bold font in Table 2): Bromodomain-Containing Protein 4 (BRD4), NF-kB Inhibitor Alpha (NFKBIA)), Cyclin-Dependent Kinase Inhibitor 1A (CDKN1A), Tissue Factor Pathway Inhibitor (TFPI), Matrix Metallopeptidase 9 (MMP9), Carbonyl Reductase 1 (CBR1), Zeta Chain Of T Cell Receptor Associated Protein Kinase 70 (ZAP70), and Isocitrate Dehydrogenase (NAD( +)) 3 Beta (IDH3B). A drug and protein target map of selected targets is shown in Fig. 5A. Of 20, 11 genes were also consistently upregulated in response to high radiation doses (10 Gy and 12 Gy) (Fig. 5B).

**Table 2:** Potential drug targets identified for acute radiation syndrome.

Gene name	Protein name	Gene Function^a^	Drug bank	CMAP drug/compound	Involvement In disease	Type of regulation	Literature support	Predicted regulation for treatment ARS	Status of drug
MYD88	Innate Immune Signal Transduction Adaptor	Adapter protein involved in the Toll-like receptor and IL-1 receptor signaling pathway in the innate immune response		ST-2825	MYD88 deficiency	Up	MyD88 may protect against long term damage and fibrosis following radiation injury^16^	Activation	Discovery and Development
**BRD4**	Bromodomain-Containing Protein 4	Chromatin reader protein that recognizes and binds acetylated histones and plays a key role in the transmission of epigenetic memory across cell divisions and transcription regulation. Remains associated with acetylated chromatin throughout the entire cell cycle and provides epigenetic memory for postmitotic G1 gene transcription by preserving acetylated chromatin status and maintaining high-order chromatin structure		CPI-0610	A chromosomal aberration involving BRD4 is found in a rare, aggressive, and lethal carcinoma arising in midline organs of young people	Up	Inhibition of BRD4 increases cancer cell survival following irradiation^17^	Inhibition	Phase 2
AURKA	Aurora Kinase A	Mitotic serine/threonine kinases that contribute to the regulation of cell cycle progression	AT9283			Up	Inhibition of AURKA increases radiosensitivity in Hepatocellular Carcinoma^18^	Activation	Phase 2
CKB	Creatine Kinase B	Reversibly catalyzes the transfer of phosphate between ATP and various phosphogens (e.g. creatine phosphate). Creatine kinase isoenzymes play a central role in energy transduction in tissues with large, fluctuating energy demands, such as skeletal muscle, heart, brain and spermatozoa	Creatine			Up	Measured Creatine levels can give a prognostic marker for radiation dose^19^	Activation	Approved
NNMT	Nicotinamide N-Methyltransferase	Catalyzes the N-methylation of nicotinamide and other pyridines to form pyridinium ions. This activity is important for biotransformation of many drugs and xenobiotic compounds	Niacin			Up	Overexpression of NNMT protects Mesenchymal Cancer stem cells from radiation therapy^20^	Activation	Approved
PTPN1	Protein Tyrosine Phosphatase Non-receptor Type 1	Tyrosine-protein phosphatase which acts as a regulator of endoplasmic reticulum unfolded protein response	Tiludronic Acid	Tiludronate		Up	Expression of PTPN1 improves survival of irradiated mice^21^	Activation	Approved
**NFKBIA**	NF-Kappa-B Inhibitor Alpha	Inhibits the activity of dimeric NF-kappa-B/REL complexes by trapping REL dimers in the cytoplasm through masking of their nuclear localization signals. On cellular stimulation by immune and proinflammatory responses, becomes phosphorylated promoting ubiquitination and degradation, enabling the dimeric RELA to translocate to the nucleus and activate transcription	Acetylsalicylic acid	Aspirin	Ectodermal dysplasia and immunodeficiency 2	Up	Blocking NF-kB increases apoptosis and decrease in growth in several cancer lineages^22^	Inhibition	Approved
MCL1	MCL1 Apoptosis Regulator	Involved in the regulation of apoptosis versus cell survival, and in the maintenance of viability but not of proliferation. Mediates its effects by interactions with a number of other regulators of apoptosis. Isoform 1 inhibits apoptosis. Isoform 2 promotes apoptosis		Rosmarinic-acid		Up	MCL1 Protects against radiation-induced apoptosis^23^	Activation	Launched
ANXA1	Annexin A1	Plays important roles in the innate immune response as an effector of glucocorticoid-mediated responses and regulator of the inflammatory process	Amcinonide	Amcinonide		Up	ANXA1 serum concentration following glucocorticoid treatment improves prognosis following radiation-induced lung injury^24^	Activation	Launched
RALB	RAS like proto-oncogene	Multifunctional GTPase involved in a variety of cellular processes including gene expression, cell migration, cell proliferation, oncogenic transformation and membrane trafficking		BQU57		Up	RALB confers radioresistance to multiple tumor types^25^	Activation	Preclinical
CBFB	Core-Binding Factor Subunit Beta	Forms the heterodimeric complex core-binding factor (CBF) with RUNX family proteins (RUNX1, RUNX2, and RUNX3)		AL-10–49		Up	Suppresses Cancer ^26^	Activation	Preclinical
ANXA2	Annexin A2	Calcium-regulated membrane-binding protein whose affinity for calcium is greatly enhanced by anionic phospholipids. It binds two calcium ions with high affinity. May be involved in the heat-stress response	Fluocinolone acetonide			Up	Prevents apoptosis^27^	Activation	Approved
MPL	MPL Proto-Oncogene, Thrombopoietin Receptor	Receptor for thrombopoietin that acts as a primary regulator of megakaryopoiesis and platelet production. May represent a regulatory molecule specific for TPO-R-dependent immune responses	Eltrombopag	Avatrombopag	Congenital amegakaryocytic thrombocytopenia	Up	c-MPL agonist confers complete survival in mice exposed to ionizing radiation^28^	Activation	Launched, Launched
**CDKN1A**	Cyclin-Dependent Kinase Inhibitor 1A	May be involved in p53/TP53 mediated inhibition of cellular proliferation in response to DNA damage. Binds to and inhibits cyclin-dependent kinase activity, preventing phosphorylation of critical cyclin-dependent kinase substrates and blocking cell cycle progression		GGTI-298		Up	CDKN1A is shown to promote cell cycle arrest in G1 phase^29^. G1 phase cell cycle arrest is seen in cells with wild-type p53 gene following irradiation^30^	Inhibition	Preclinical
**TFPI**	Tissue Factor Pathway Inhibitor	Inhibits factor X (X(a)) directly and, in a Xa-dependent way, inhibits VIIa/tissue factor activity, presumably by forming a quaternary Xa/LACI/VIIa/TF complex. It possesses an antithrombotic action and also the ability to associate with lipoproteins in plasma	Dalteparin	Dalteparin		UP	TFPI inhibition can inhibit hemophilia^31^. Radiation-induced coagulopathy decreases clotting ability^32^	Inhibition	Approved
**MMP9**	Matrix Metallopeptidase 9	May play an essential role in local proteolysis of the extracellular matrix and in leukocyte migration. Could play a role in bone osteoclastic resorption. Cleaves KiSS1 at a Gly-|-Leu bond. Cleaves type IV and type V collagen into large C-terminal three quarter fragments and shorter N-terminal one quarter fragments. Degrades fibronectin but not laminin or Pz-peptide	Captopril		Intervertebral disc disease	UP	Captopril is an inhibitor of MMP9 and has shown increased survival in mice depending on dosage timing^33^	Inhibition	Launched
IGF1R	Insulin-Like Growth Factor 1 Receptor	Receptor tyrosine kinase which mediates actions of insulin-like growth factor 1 (IGF1). Binds IGF1 with high affinity and IGF2 and insulin (INS) with a lower affinity. The activated IGF1R is involved in cell growth and survival control. IGF1R is crucial for tumor transformation and survival of malignant cell	ATL1101		Insulin-like growth factor resistance	UP	Overexpression of IGF1R is known to confer increased radioresistance in chemotherapy^34^	Activation	Investigational
**CBR1**	Carbonyl Reductase 1	NADPH-dependent reductase with broad substrate specificity. Catalyzes the reduction of a wide variety of carbonyl compounds including quinones, prostaglandins, menadione, plus various xenobiotics	Acetohexamide			Down	Carbonyl Reductase confers radioprotectivity in head and neck squamous cell carcinoma^35^	Activation	Launched
**ZAP70**	Zeta Chain of T Cell Receptor Associated Protein Kinase 70	Tyrosine kinase that plays an essential role in regulation of the adaptive immune response. Regulates motility, adhesion and cytokine expression of mature T-cells, as well as thymocyte development. Contributes also to the development and activation of primary B-lymphocytes	Staurosporine		Immunodeficiency 48	Down	Zap70 Deficiency leads to a loss of T cells^36^ Radiation induces lymphopenia^37^	Activation	Preclinical
**IDH3B**	Isocitrate Dehydrogenase (NAD(+)) 3 Beta	Plays a structural role to facilitate the assembly and ensure the full activity of the enzyme catalyzing the decarboxylation of isocitrate (ICT) into alpha-ketoglutarate	NADH	Coenzyme i	Retinitis Pigmentosa	Down	Isocitrate Dehydrogenase generates NADH. NADH is radioprotective for mouse intestine^38^	Activation	Approved, Phase 2

^a^The gene functions were obtained from the Uniprot database^39^.

## Discussion

The onset of Acute Radiation Syndrome (ARS) upon exposure to high doses of radiation in a short period is a burgeoning problem as access to highly radioactive substances becomes ubiquitous. Thus, the importance of characterizing and treating this disease is an emerging endeavor. As such, we created gene correlation networks to help identify hub genes across multiple transcriptomic data for ARS to identify potential therapeutic options. Our transcriptomics-based gene networks were enriched with biological processes, including immune response and immune system process that were supported by another study^40^. While this study provided an overview of potential treatments for ARS there were significant limitations. The use of multiple species, multiple time points and multiple radiation doses means our predictions are a holistic screening of potential treatments for ARS. Within the immune response, pathways related to Blood Coagulation and platelet activation, signaling and aggregation were enriched processes in the networks. It has been shown previously that coagulopathy and lower platelet counts are physiologically-relevant symptoms of acute radiation^32^. Identification of genes that have been independently shown as relevant to ARS provides partial validation to the constructed networks. Evidence was found for eight hub genes identified as potential targets (BRD4, NFKBIA, CDKN1A, TFPI, MMP9, CBR1, ZAP70, IDH3B), that altering their expression may induce radioprotective effects. These eight genes and associated drugs that have the potential for repurposing are discussed below.

### Upregulated genes

**Bromodomain-Containing Protein 4 (BRD4):** BRD4 Inhibition was shown to condense chromatin structure and decrease the DNA damage signaling pathway resulting in less DNA repair. Further, inhibition of BRD4 demonstrated a relaxation of the chromatin structure and more repair signalling^17^. Thus, inhibition of BRD4 using CPI-0610, a phase two clinical trial molecule, could result in a better prognosis for ARS patients. **NF-kB inhibitor alpha (NFKBIA):** NF-kB inhibition has been shown to increase apoptosis through the loss of regulation of the JNK pathway^22^. Additionally, increased apoptotic activity is induced by irradiation^41^. Thus inhibition of **NFKBIA** by Acetylsalicylic acid could increase NF-kB activity and confer an antiapoptotic state for the irradiated cells. **Cyclin-dependent kinase inhibitor alpha (CDKN1A)**: It has been shown that active and overexpressed **CDKN1A** increases cell cycle arrest in the G1 phase^29^. Arrest in this phase of the cell cycle was seen in irradiated cells, and cell cycle arrest is a known promoter of apoptosis^30^. A preclinical compound GGTI-298 is under investigation that can inhibit CDKN1A. **Tissue Factor Pathway inhibitor (TFPI):** It has been shown that inhibition of **TFPI** reduces hemophilia bleeding through activating coagulation pathways^31^. Since hemophilia is an indication of Radiation-Induced Disseminated Intravascular Coagulopathy (RDIC) that fresh frozen plasma, platelets or cryoprecipitated antihemophilic factor (AHF) ^30^. It is possible that inhibition of TFPI or its combination with human derived products such as AHF may improve the patients’ outcome by mitigating the effects of hemophilia in ARS. FDA-approved drug, dalteparin, is an inhibitor available for TFPI. **Matrix Metallopeptidase 9 (MMP9):** has been suggested to increase fibrosis following damage^42^; fibrosis is increased in cells that receive ionizing radiation^43^. Additionally, captopril is an FDA-approved and known inhibitor of **MMP9**^13^ that improves the survival of mice when exposed to high doses of radiation^33^. This protection was highly dependent on the timing of the treatment and suggests further time based inquiries of the data may elucidate time dependent physiological manifestations of ARS.

### Downregulated genes

**Carbonyl Reductase 1 (CBR1)** encodes a protein that reduces the level of reactive oxygen species, especially lipid aldehydes. Further, it has been shown that CBR1 confers radioprotectivity to head and neck squamous cell carcinomas^35^. Thus increasing the activity level of **CBR1** may decrease the oxidative stress induced by radiation. FDA-approved acetohexamide currently used to treat type II diabetes can be used as an activator of CBR1. **Zeta Chain of T Cell Receptor Associated Protein Kinase 70 (ZAP70)** regulates T-cell signaling via interaction with cytoplasmic tyrosine kinases^44^. Loss-of-function in ZAP70 has been shown to lead to a systemic loss of functional T cells^36^ in mice post radiation^37^. Thus, we hypothesize that upregulating ZAP70 may lead to regaining functional T cells following radiation. Staurosporine is an experimental drug known to modulate the ZAP70 function and could be further explored as a potential repurposing option for ARS. Finally, **Isocitrate Dehydrogenase (NAD( +)) 3 Beta (IDH3B)** facilitates the transfer of Hydrogen from Isocitrate to NAD(+); exogenous NADH has been shown to protect mice intestines after radiation injury^38^. Thus, we hypothesize that the increase of IDH3B can lead to higher levels of NADH in the system and thus improve the prognosis following radiation injury. The compound coenzyme I (in Phase 2 of clinical trials) is indeed known to interact with IDH3B and could be explored for repurposing in ARS.

In addition to the above-discussed genes, all other reported genes elucidate a physiological state that is induced under various radiation regiments. We predicted the possible activation of 12 other genes that have drugs approved by the FDA or in preclinical and clinical phases. Based on published literature, the activation of these genes may have radioprotective properties, and thus the development of corresponding activators may be useful. These include MYD88 (Myeloid Differentiation Primary Response Protein), which is found as upregulated in response to radiation and has been found to protect against radiation injuries^16^. The upregulation of Aurora Kinase A (AURKA) may also lead to protective properties against radiation as its inhibition has been found to increase radiosensitivity in hepatocellular carcinoma^18^. Similarly, activation of Protein Tyrosine Phosphatase Non-receptor Type 1 (PTPN1) has been found to increase survival in mice after irradiation. Finally, Nicotinamide N-Methyltransferase (NNMT) has been found to protect cancer cells from radiation.

Further delineation based on timing and dose could further specify which treatment and response best suit the target. Additional studies can be conducted to determine the efficacy of modulating these genes individually or in combination. Additionally, the data was garnered primarily from mice which will heavily influence the predicted treatments. Finally, while this modulation can confer protection against acute radiation syndrome, some of these genes can also provide radioprotective properties to tumor cells and hence may not represent a long-term treatment option for patients with ARS.

## Conclusions

This study uses systems-level gene associations integrated with multiple biological information levels and provides a new perspective for the treatment of Acute Radiation Syndrome (ARS). The study identified eight genes with existing drugs and relevance to ARS, which may serve as high confidence drug target candidates for drug repurposing, potentially providing treatment of ARS.

### Dataset collection

Gene expression datasets GSE104121^45^, GSE10640^46^, and GSE6874^47^ related to ARS were collected from the GEO database^48^. These datasets were generated from studies using humans and mice. In GSE104121, peripheral whole blood samples were collected from 48 mice following 1–12 Gy total body irradiation (TBI), at 6 h, 16 h, 24 h, and 48 h after the treatment. GSE10640 was generated using human and mouse samples. Human data were collected prior to and following the total body irradiation (TBI) of patients with 1.5–2 Gy. From this dataset we used 70 human samples (36 Pre Irradiation 34 post irradiation). These samples came from 6 h after TBI of 1.5–2.0 Gy in preparation for transplantation. The mouse data were collected following 6 h, 24 h, and 7 days of TBI with 0.5, 2, and 10 Gy. GSE6874 consists of two datasets GSE6871 (human) and GSE6873 (mouse). Human data were collected from 60 samples (33 pre-irradiation and 27 post-irradiation) pre- and post-irradiated (with 1.5–2 Gy TBI) patients. Mouse data were collected following TBI varied from 0.5 Gy to 10 Gy.

### Differential gene expression analysis

Differential gene expression analysis was conducted for each dataset using *limma*^49^ and *affy*^50^ R/Bioconductor packages. The comparison was made between healthy samples and the various conditions, including radiation dose and time post-irradiation. Additionally, within GSE10640, there were multiple species of mice used (Balbc and Blk); hence, differential expression was performed between healthy Balbc vs. Irradiated and similarly for the Blk mice. A total of 32 comparisons for differential expression analysis were performed across all five datasets (Supplementary Data 5). Two-fold change was considered as a significant differential expression.

### Weighted gene coexpression network construction

Prior to the construction of the network, datasets were quantile normalized using the *limma* package in R. Next, genes were filtered based on the coefficient of variation (CV) to remove spurious correlations among genes. A total of five networks were constructed consisting of two human (one from GSE10640 one from GSE6874), and three mice networks (GSE10640, GSE6874, GSE104121).

### Pearson’s correlation coefficient between genes within each dataset

Assuming the biological networks show scale-free topology, the absolute correlation between genes was raised with a soft thresholding power (β) (Supplementary Data 1). All networks were fitted using an R^2 cutoff of 0.80 ^6^. The clustering of genes was performed based on the dissimilarities of genes using topological overlap measure (TOM). Average Linkage Hierarchical clustering was used for module detection in each network^6^.

### Hub genes identification

Hub genes were identified in each correlation network characterized by the genes with the highest intramodular connectivity value^6^. This value determines the level of module membership a gene has; thus, a higher value of intramodular connectivity corresponds to more significant connectivity in the module. Genes among the top 20 percent were selected to ensure the highest confidence in the genes selected while simultaneously not introducing false positives by lowering the threshold.

### Identification of known drugs and correlation with diseases

To investigate if a hub gene is an existing drug target, we used the repurposing tool^51^ in the ConnectivityMap^52^ and DrugBank databases^13^. Additionally, we utilized UniProt ^39^ and OMIM databases^53^ to map genes to associated diseases.

### Functional enrichment analysis

Gene Ontology (GO) biological processes^54^, KEGG pathways^55^, and Reactome pathways^56^ terms were utilized for enrichment analysis.

Enrichment of genes within each module in the networks was performed using the anRichment^57^ and anRichmentMethods packages in R. Pathways with Benjamini–Hochberg (FDR) value < 0.05 were considered to be enriched.

A functional analysis of hub genes was performed using DAVID^58^, STRING^59^ and UniProt databases. In the DAVID database and STRING database, biological processes and pathways with FDR < 0.05 were considered as enriched.

## Supplementary Information

Supplementary Information 1.

Supplementary Information 2.

## Data Availability

The publicly available datasets analyzed during the current study are available in the Gene Expression Omnibus. GSE10640 for human and mice irradiated PBMCs—https://www.ncbi.nlm.nih.gov/geo/query/acc.cgi?acc=GSE10640. GSE6874 For human and mice irradiated PBMCs—https://www.ncbi.nlm.nih.gov/geo/query/acc.cgi?acc=GSE6874.
