# Author Correction: Integrative network analyses of transcriptomics data reveal potential drug targets for acute radiation syndrome

**DOI:** 10.1038/s41598-021-86614-3

**Published:** 2021-03-23

**Authors:** Robert Moore, Bhanwar Lal Puniya, Robert Powers, Chittibabu Guda, Kenneth W. Bayles, David B. Berkowitz, Tomáš Helikar

**Affiliations:** 1 Department of Biochemistry, University of Nebraska-Lincoln, Lincoln, NE USA; 2 Department of Chemistry, University of Nebraska-Lincoln, Lincoln, NE USA; 3 Department of Genetics, Cell Biology & Anatomy, University of Nebraska Medical Center, Omaha, NE USA; 4 Department of Pathology and Microbiology, University of Nebraska Medical Center, Omaha, NE USA

Correction to: *Scientific Reports* 10.1038/s41598-021-85044-5, published online 10 March 2021

The original version of this Article contained a typographical error in the spelling of the author David B. Berkowitz, which was incorrectly given as David Berkowitz. This has now been corrected in the PDF and HTML versions of the Article, and in the accompanying Supplementary Information file.

