# Supplementary material for: Integrative network analyses of transcriptomics data reveal potential drug targets for acute radiation syndrome
Source: Sci Rep. 2021 Mar 10;11:5585. doi: 10.1038/s41598-021-85044-5 (PMC7946886; doi:10.1038/s41598-021-85044-5)
Supplement: Supplementary file 2 — Supplementary Information 2. [file 41598_2021_85044_MOESM2_ESM.docx]

**Integrative network analyses of transcriptomics data reveal potential drug targets for acute radiation syndrome**

# Robert Moore^1#^, Bhanwar Lal Puniya^1#^, Robert Powers^2^, Chittibabu Guda^3^, Kenneth W Bayles^4^, David B Berkowitz^2^, Tomáš Helikar^1^*

# ^1^ Department of Biochemistry, University of Nebraska-Lincoln, Lincoln, NE, USA ^2^ Department of Chemistry, University of Nebraska-Lincoln, Lincoln, NE, USA ^3^ Department of Genetics, Cell Biology & Anatomy, University of Nebraska Medical Center, Omaha, NE, USA ^4^ Department of Pathology and Microbiology, University of Nebraska Medical Center, Omaha, NE, USA

# **^#^ These authors contributed equally to this work**

# ***To whom correspondence should be addressed:** Tomáš Helikar, Ph.D., Department of Biochemistry, University of Nebraska-Lincoln, USA, Email:[thelikar2@unl.edu](mailto:thelikar2@unl.edu)

supplementary Data 1

| Data set | Soft thresholding power used for network construction |
| --- | --- |
| GSE10640 Human | 16 |
| GSE10640 Mice | 16 |
| GSE6874 Human | 6 |
| GSE6874 Mice | 3 |
| GSE104121 | 20 |

supplementary Data 2

| Dataset | Pathway | FDR | Enrichment |
| --- | --- | --- | --- |
| GSE104121 | signal transduction | 0.0008663008591 | 1.372015734 |
| GSE104121 | negative regulation of response to stimulus | 0.0007289484745 | 1.830788352 |
| GSE104121 | cell surface receptor signaling pathway | 0.0007274165989 | 1.659130049 |
| GSE104121 | negative regulation of biological process | 0.0006861723929 | 1.39837689 |
| GSE104121 | negative regulation of signal transduction | 0.0004932151583 | 2.02162102 |
| GSE104121 | intracellular signal transduction | 0.0001415035095 | 1.66067919 |
| GSE104121 | regulation of response to stimulus | 7.12E-05 | 1.546254075 |
| GSE104121 | regulation of cell communication | 7.55E-06 | 1.661629829 |
| GSE104121 | regulation of signaling | 6.07E-06 | 1.664176125 |
| GSE104121 | regulation of signal transduction | 6.33E-07 | 1.787386856 |
| GSE104121 | protein metabolic process | 4.55E-07 | 1.0623238 |
| GSE104121 | Metabolism of xenobiotics by cytochrome P450 (KEGG) | 3.61E-07 | 20.10118023 |
| GSE104121 | organic cyclic compound metabolic process | 3.36E-07 | 1.062704189 |
| GSE104121 | humoral immune response mediated by circulating immunoglobulin | 3.19E-07 | 26.53085068 |
| GSE104121 | sterol esterase activity | 3.19E-07 | 54.71987952 |
| GSE104121 | cellular protein catabolic process | 3.10E-07 | 1.183148378 |
| GSE104121 | organonitrogen compound metabolic process | 2.83E-07 | 1.05683595 |
| GSE104121 | biosynthetic process | 2.78E-07 | 1.064021652 |
| GSE104121 | cellular biosynthetic process | 2.76E-07 | 1.065545065 |
| GSE104121 | Immune System (REACTOME) | 2.76E-07 | 1.119264311 |
| GSE104121 | cellular macromolecule biosynthetic process | 2.68E-07 | 1.074526042 |
| GSE104121 | envelope | 2.38E-07 | 1.150278041 |
| GSE104121 | glucuronosyltransferase activity | 2.35E-07 | 38.30391566 |
| GSE104121 | regulation of coagulation | 2.20E-07 | 17.09996235 |
| GSE104121 | cellular protein-containing complex assembly | 1.98E-07 | 1.157605029 |
| GSE104121 | organic substance biosynthetic process | 1.98E-07 | 1.065435337 |
| GSE104121 | organelle envelope | 1.72E-07 | 1.151679111 |
| GSE104121 | regulation of hemostasis | 1.62E-07 | 17.65157404 |
| GSE104121 | Synthesis of (16-20)-hydroxyeicosatetraenoic acids (HETE) (REACTOME) | 1.59E-07 | 40.31991122 |
| GSE104121 | Neutrophil degranulation (REACTOME) | 1.56E-07 | 1.209178905 |
| GSE104121 | small molecule biosynthetic process | 1.56E-07 | 5.004203694 |
| GSE104121 | aspartate family amino acid metabolic process | 1.52E-07 | 29.18393574 |
| GSE104121 | regulation of blood coagulation | 1.41E-07 | 17.9409441 |
| GSE104121 | iron ion binding | 1.26E-07 | 11.11497553 |
| GSE104121 | alpha-amino acid catabolic process | 1.21E-07 | 18.23995984 |
| GSE104121 | cellular amino acid biosynthetic process | 1.19E-07 | 22.90599608 |
| GSE104121 | cellular aromatic compound metabolic process | 1.11E-07 | 1.066166596 |
| GSE104121 | negative regulation of proteolysis | 1.11E-07 | 7.441903614 |
| GSE104121 | coagulation | 1.06E-07 | 11.29140371 |
| GSE104121 | peptidase regulator activity | 1.05E-07 | 8.970472052 |
| GSE104121 | Serotonergic synapse (KEGG) | 9.48E-08 | 13.13277108 |
| GSE104121 | hemostasis | 8.82E-08 | 11.47352312 |
| GSE104121 | blood coagulation | 8.01E-08 | 11.5668038 |
| GSE104121 | RNA metabolic process | 6.87E-08 | 1.08014729 |
| GSE104121 | nucleic acid metabolic process | 6.87E-08 | 1.074039363 |
| GSE104121 | endoplasmic reticulum | 6.77E-08 | 3.221309716 |
| GSE104121 | Porphyrin and chlorophyll metabolism (KEGG) | 6.51E-08 | 32.42659527 |
| GSE104121 | translation | 5.77E-08 | 1.219550449 |
| GSE104121 | negative regulation of coagulation | 5.02E-08 | 25.25532901 |
| GSE104121 | negative regulation of hemostasis | 5.02E-08 | 25.25532901 |
| GSE104121 | heterocycle metabolic process | 4.15E-08 | 1.068558488 |
| GSE104121 | negative regulation of blood coagulation | 3.97E-08 | 25.91994293 |
| GSE104121 | L13a-mediated translational silencing of Ceruloplasmin expression (REACTOME) | 3.86E-08 | 1.471136031 |
| GSE104121 | cellular amino acid catabolic process | 3.68E-08 | 16.95545563 |
| GSE104121 | structural constituent of ribosome | 3.62E-08 | 1.399213825 |
| GSE104121 | catalytic complex | 2.95E-08 | 1.141179197 |
| GSE104121 | SRP-dependent cotranslational protein targeting to membrane (REACTOME) | 2.94E-08 | 1.506443296 |
| GSE104121 | GTP hydrolysis and joining of the 60S ribosomal subunit (REACTOME) | 2.71E-08 | 1.47188091 |
| GSE104121 | negative regulation of endopeptidase activity | 2.40E-08 | 11.26585755 |
| GSE104121 | enzyme inhibitor activity | 2.40E-08 | 7.072637489 |
| GSE104121 | Pentose and glucuronate interconversions (KEGG) | 2.40E-08 | 36.47991968 |
| GSE104121 | peptide biosynthetic process | 2.26E-08 | 1.220427081 |
| GSE104121 | protein-containing complex subunit organization | 2.25E-08 | 1.117194525 |
| GSE104121 | Drug metabolism - cytochrome P450 (KEGG) | 1.74E-08 | 22.3346447 |
| GSE104121 | Post-translational protein modification (REACTOME) | 1.70E-08 | 1.183025044 |
| GSE104121 | macromolecule catabolic process | 1.57E-08 | 1.152956335 |
| GSE104121 | Ribosome, eukaryotes (KEGG) | 1.54E-08 | 1.488630622 |
| GSE104121 | Synthesis of epoxy (EET) and dihydroxyeicosatrienoic acids (DHET) (REACTOME) | 1.47E-08 | 54.71987952 |
| GSE104121 | peptidase inhibitor activity | 1.44E-08 | 10.45602793 |
| GSE104121 | endopeptidase regulator activity | 9.05E-09 | 10.79997622 |
| GSE104121 | Ribosome (KEGG) | 7.71E-09 | 1.42894496 |
| GSE104121 | Formation of a pool of free 40S subunits (REACTOME) | 6.32E-09 | 1.507807828 |
| GSE104121 | nucleobase-containing compound metabolic process | 6.09E-09 | 1.072930132 |
| GSE104121 | endopeptidase inhibitor activity | 5.73E-09 | 11.16732235 |
| GSE104121 | serine-type endopeptidase inhibitor activity | 5.07E-09 | 17.05554686 |
| GSE104121 | vitamin binding | 4.74E-09 | 14.6671842 |
| GSE104121 | Glycine, serine and threonine metabolism (KEGG) | 4.48E-09 | 32.83192771 |
| GSE104121 | Eukaryotic Translation Initiation (REACTOME) | 4.47E-09 | 1.475339277 |
| GSE104121 | Cap-dependent Translation Initiation (REACTOME) | 4.47E-09 | 1.475339277 |
| GSE104121 | sulfur amino acid metabolic process | 3.24E-09 | 33.96406315 |
| GSE104121 | Ascorbate and aldarate metabolism (KEGG) | 3.04E-09 | 46.07989854 |
| GSE104121 | mitochondrial protein complex | 2.82E-09 | 1.331083881 |
| GSE104121 | steroid metabolic process | 2.82E-09 | 8.755180723 |
| GSE104121 | response to chemical | 2.54E-09 | 2.420893546 |
| GSE104121 | cellular nitrogen compound biosynthetic process | 1.74E-09 | 1.084839355 |
| GSE104121 | nuclear lumen | 1.56E-09 | 1.090691337 |
| GSE104121 | ribosomal subunit | 1.11E-09 | 1.385253495 |
| GSE104121 | Nonsense-Mediated Decay (NMD) (REACTOME) | 1.10E-09 | 1.492204152 |
| GSE104121 | Nonsense Mediated Decay (NMD) enhanced by the Exon Junction Complex (EJC) (REACTOME) | 1.10E-09 | 1.492204152 |
| GSE104121 | Nonsense Mediated Decay (NMD) independent of the Exon Junction Complex (EJC) (REACTOME) | 1.08E-09 | 1.529981472 |
| GSE104121 | Glucuronate pathway (uronate pathway) (KEGG) | 1.08E-09 | 51.50106308 |
| GSE104121 | gene expression | 1.06E-09 | 1.078183807 |
| GSE104121 | cellular process | 8.95E-10 | 1.030342621 |
| GSE104121 | lipid catabolic process | 8.95E-10 | 8.663980924 |
| GSE104121 | negative regulation of peptidase activity | 6.78E-10 | 10.5111633 |
| GSE104121 | negative regulation of hydrolase activity | 6.21E-10 | 7.635332026 |
| GSE104121 | Ascorbate biosynthesis, animals, glucose-1P => ascorbate (KEGG) | 6.14E-10 | 54.71987952 |
| GSE104121 | ribosome | 6.11E-10 | 1.36291453 |
| GSE104121 | Translation (REACTOME) | 6.04E-10 | 1.466232244 |
| GSE104121 | Linoleic acid metabolism (KEGG) | 5.94E-10 | 31.26850258 |
| GSE104121 | arachidonic acid epoxygenase activity | 5.35E-10 | 41.03990964 |
| GSE104121 | Innate Immune System (REACTOME) | 4.05E-10 | 1.165619843 |
| GSE104121 | RNA binding | 4.01E-10 | 1.179649363 |
| GSE104121 | cellular macromolecule catabolic process | 3.81E-10 | 1.18272266 |
| GSE104121 | organic substance catabolic process | 3.25E-10 | 3.410304106 |
| GSE104121 | primary metabolic process | 3.15E-10 | 1.049437069 |
| GSE104121 | epoxygenase P450 pathway | 2.20E-10 | 44.77081051 |
| GSE104121 | Formation of Fibrin Clot (Clotting Cascade) (REACTOME) | 1.61E-10 | 35.30314808 |
| GSE104121 | Metabolism of amino acids and derivatives (REACTOME) | 1.14E-10 | 10.70606338 |
| GSE104121 | macromolecule metabolic process | 9.86E-11 | 1.055734491 |
| GSE104121 | cellular protein metabolic process | 8.16E-11 | 1.082673456 |
| GSE104121 | fatty acid derivative metabolic process | 5.42E-11 | 15.63425129 |
| GSE104121 | long-chain fatty acid metabolic process | 4.69E-11 | 21.23458011 |
| GSE104121 | organic substance metabolic process | 4.29E-11 | 1.04944124 |
| GSE104121 | cellular nitrogen compound metabolic process | 4.13E-11 | 1.074313077 |
| GSE104121 | ribonucleoprotein complex | 3.70E-11 | 1.208783439 |
| GSE104121 | Metabolism of lipids and lipoproteins (REACTOME) | 3.27E-11 | 5.739155537 |
| GSE104121 | nuclear part | 2.93E-11 | 1.091165417 |
| GSE104121 | nitrogen compound metabolic process | 2.45E-11 | 1.054270996 |
| GSE104121 | organic acid catabolic process | 2.37E-11 | 11.79590217 |
| GSE104121 | carboxylic acid catabolic process | 2.37E-11 | 11.79590217 |
| GSE104121 | arachidonic acid monooxygenase activity | 1.48E-11 | 43.77590361 |
| GSE104121 | bupropion degradation (BIOCYC) | 1.48E-11 | 43.77590361 |
| GSE104121 | intracellular organelle lumen | 1.21E-11 | 1.093965541 |
| GSE104121 | cellular catabolic process | 1.21E-11 | 3.46968853 |
| GSE104121 | membrane-enclosed lumen | 1.12E-11 | 1.197204137 |
| GSE104121 | organelle lumen | 1.12E-11 | 1.094114657 |
| GSE104121 | aromatase activity | 9.69E-12 | 45.5998996 |
| GSE104121 | cytoplasm | 4.88E-12 | 1.046331454 |
| GSE104121 | cellular amino acid metabolic process | 4.12E-12 | 10.88952826 |
| GSE104121 | icosanoid metabolic process | 3.79E-12 | 18.86892397 |
| GSE104121 | cytoplasmic part | 3.77E-12 | 1.057664023 |
| GSE104121 | metabolic process | 3.76E-12 | 1.049666307 |
| GSE104121 | response to xenobiotic stimulus | 3.26E-12 | 12.08927571 |
| GSE104121 | cell part | 2.71E-12 | 1.031296001 |
| GSE104121 | small molecule catabolic process | 2.43E-12 | 9.592329477 |
| GSE104121 | cell | 2.32E-12 | 1.031380075 |
| GSE104121 | Metabolism of proteins (REACTOME) | 1.97E-12 | 1.18779592 |
| GSE104121 | protein activation cascade | 1.90E-12 | 27.35993976 |
| GSE104121 | nicotine degradation III (BIOCYC) | 1.72E-12 | 41.51163274 |
| GSE104121 | fatty acid metabolic process | 7.17E-13 | 8.873493976 |
| GSE104121 | unsaturated fatty acid metabolic process | 6.57E-13 | 21.31943358 |
| GSE104121 | cellular macromolecule metabolic process | 4.10E-13 | 1.068111125 |
| GSE104121 | nicotine degradation II (BIOCYC) | 2.42E-13 | 38.62579731 |
| GSE104121 | alpha-amino acid metabolic process | 1.04E-13 | 14.64334804 |
| GSE104121 | arachidonic acid metabolic process | 6.53E-14 | 34.7004114 |
| GSE104121 | cellular response to xenobiotic stimulus | 3.02E-14 | 17.43288197 |
| GSE104121 | oxidoreductase activity, acting on paired donors, with incorporation or reduction of molecular oxygen | 2.20E-14 | 14.39996829 |
| GSE104121 | nucleus | 1.09E-14 | 1.07380258 |
| GSE104121 | organic substance metabolic process | 6.74E-15 | 1.827241084 |
| GSE104121 | cellular metabolic process | 1.86E-15 | 1.059585465 |
| GSE104121 | primary metabolic process | 1.72E-15 | 1.881755421 |
| GSE104121 | oxidoreductase activity | 1.61E-15 | 6.492189095 |
| GSE104121 | catalytic activity | 9.47E-16 | 2.33643819 |
| GSE104121 | Cytochrome P450 - arranged by substrate type (REACTOME) | 6.54E-16 | 24.4799461 |
| GSE104121 | exogenous drug catabolic process | 5.97E-16 | 39.28606735 |
| GSE104121 | metabolic process | 5.26E-16 | 1.813524191 |
| GSE104121 | catabolic process | 5.26E-16 | 3.69470665 |
| GSE104121 | Xenobiotics (REACTOME) | 3.68E-16 | 49.05920233 |
| GSE104121 | organelle part | 3.14E-16 | 1.071711886 |
| GSE104121 | drug catabolic process | 1.64E-16 | 18.5491117 |
| GSE104121 | xenobiotic metabolic process | 9.46E-17 | 24.02336174 |
| GSE104121 | steroid hydroxylase activity | 6.31E-17 | 32.42659527 |
| GSE104121 | cellular lipid metabolic process | 5.46E-17 | 6.046395527 |
| GSE104121 | tetrapyrrole binding | 2.95E-17 | 18.23995984 |
| GSE104121 | organelle | 1.39E-17 | 1.050023142 |
| GSE104121 | monooxygenase activity | 1.36E-17 | 21.04610751 |
| GSE104121 | heme binding | 9.24E-18 | 19.31289865 |
| GSE104121 | Gene Expression (REACTOME) | 5.32E-18 | 1.221488704 |
| GSE104121 | oxidation-reduction process | 2.06E-18 | 6.65287289 |
| GSE104121 | oxidoreductase activity, acting on paired donors, with incorporation or reduction of molecular oxygen, reduced flavin or flavoprotein as one donor, and incorporation of one atom of oxygen | 1.75E-18 | 39.79627601 |
| GSE104121 | Chemical carcinogenesis (KEGG) | 1.74E-18 | 29.84720701 |
| GSE104121 | protein-containing complex | 1.33E-18 | 1.097704675 |
| GSE104121 | intracellular organelle | 1.13E-18 | 1.052777017 |
| GSE104121 | Biological oxidations (REACTOME) | 8.38E-19 | 15.11598882 |
| GSE104121 | intracellular organelle part | 8.08E-19 | 1.079738626 |
| GSE104121 | Phase 1 - Functionalization of compounds (REACTOME) | 5.78E-19 | 20.06395582 |
| GSE104121 | cofactor binding | 4.88E-19 | 8.94459569 |
| GSE104121 | drug metabolic process | 3.40E-19 | 7.616686526 |
| GSE104121 | monocarboxylic acid metabolic process | 2.47E-19 | 8.745186224 |
| GSE104121 | intracellular | 2.16E-19 | 1.047041834 |
| GSE104121 | intracellular part | 2.16E-19 | 1.047041834 |
| GSE104121 | intracellular membrane-bounded organelle | 6.10E-20 | 1.062607902 |
| GSE104121 | membrane-bounded organelle | 2.66E-20 | 1.059516325 |
| GSE104121 | Retinol metabolism (KEGG) | 1.84E-20 | 33.53799067 |
| GSE104121 | lipid metabolic process | 1.87E-21 | 5.765937718 |
| GSE104121 | Metabolic pathways (KEGG) | 7.23E-24 | 5.849549803 |
| GSE104121 | extracellular region | 3.58E-24 | 4.333022134 |
| GSE104121 | extracellular region part | 1.34E-24 | 5.047368096 |
| GSE104121 | Complement and coagulation cascades (KEGG) | 2.96E-25 | 37.61991717 |
| GSE104121 | extracellular space | 7.70E-27 | 5.678046076 |
| GSE104121 | Steroid hormone biosynthesis (KEGG) | 4.94E-29 | 41.69133678 |
| GSE104121 | Metabolism (REACTOME) | 8.97E-30 | 5.27868403 |
| GSE104121 | oxoacid metabolic process | 3.95E-30 | 8.151375156 |
| GSE104121 | carboxylic acid metabolic process | 6.67E-31 | 8.527773431 |
| GSE104121 | small molecule metabolic process | 6.67E-31 | 5.653714489 |
| GSE104121 | organic acid metabolic process | 2.71E-33 | 8.497330688 |
| GSE6874 M | Gap junction (KEGG) | 0.04899210914 | 15.71623094 |
| GSE6874 M | Renin secretion (KEGG) | 0.01936839462 | 19.2881016 |
| GSE6874 M | response to wounding | 0.01274873375 | 2.51686593 |
| GSE6874 M | Cell junction organization (REACTOME) | 0.00349996199 | 7.581229018 |
| GSE6874 M | coagulation | 0.002558585225 | 4.057361456 |
| GSE6874 M | wound healing | 0.002224552648 | 2.907087819 |
| GSE6874 M | blood coagulation | 0.00212993919 | 4.126130294 |
| GSE6874 M | hemostasis | 0.00212993919 | 4.126130294 |
| GSE6874 M | Response to elevated platelet cytosolic Ca2+ (REACTOME) | 0.0001032244184 | 5.07170182 |
| GSE6874 M | Platelet degranulation (REACTOME) | 0.0001032244184 | 5.07170182 |
| GSE6874 M | nucleoplasm | 1.10E-05 | 1.205974863 |
| GSE6874 M | intracellular organelle | 3.52E-06 | 1.06820207 |
| GSE6874 M | Platelet activation, signaling and aggregation (REACTOME) | 2.09E-06 | 4.419783721 |
| GSE6874 M | membrane-bounded organelle | 1.43E-06 | 1.077600212 |
| GSE6874 M | intracellular membrane-bounded organelle | 1.12E-06 | 1.08451849 |
| GSE6874 M | Hemostasis (REACTOME) | 3.38E-07 | 3.379899203 |
| GSE6874 M | nucleus | 2.24E-08 | 1.134728965 |
| GSE6874 M | nuclear lumen | 1.38E-08 | 1.21056766 |
| GSE6874 M | intracellular organelle lumen | 1.38E-08 | 1.19819438 |
| GSE6874 M | membrane-enclosed lumen | 1.38E-08 | 1.197204137 |
| GSE6874 M | organelle lumen | 1.38E-08 | 1.197204137 |
| GSE6874 M | nuclear part | 3.98E-09 | 1.203051384 |
| GSE6874 H | innate immune response | 0.03950278805 | 1.204278781 |
| GSE6874 H | immune effector process | 0.02203429534 | 1.175825983 |
| GSE6874 H | defense response | 0.02102863408 | 1.150746247 |
| GSE6874 H | cellular response to cytokine stimulus | 0.0183239832 | 1.183421758 |
| GSE6874 H | regulation of immune response | 0.0183239832 | 1.198077136 |
| GSE6874 H | regulation of immune system process | 0.002349724658 | 1.170162994 |
| GSE6874 H | immune response | 0.0003186379708 | 1.159548697 |
| GSE6874 H | immune system process | 0.0002702144546 | 1.128143505 |
| GSE10640 M | antigen receptor-mediated signaling pathway | 0.0481412271 | 9.819480519 |
| GSE10640 M | immune response | 0.0481412271 | 1.697775577 |
| GSE10640 M | lymphocyte differentiation | 0.0481412271 | 2.45089141 |
| GSE10640 M | lymphocyte activation | 0.0481412271 | 2.008255503 |
| GSE10640 M | B cell activation | 0.02924403303 | 7.603580048 |
| GSE10640 M | regulation of response to stimulus | 0.02924403303 | 1.374260673 |
| GSE10640 M | B cell receptor signaling pathway | 0.02090450021 | 19.8277972 |
| GSE10640 M | platelet formation | 0.02083011459 | 17.44175317 |
| GSE10640 M | platelet morphogenesis | 0.02083011459 | 17.44175317 |
| GSE10640 M | T cell differentiation | 0.02083011459 | 2.928056109 |
| GSE10640 M | immune system process | 0.02083011459 | 1.528725024 |
| GSE10640 M | leukocyte activation | 0.02083011459 | 1.943583242 |
| GSE10640 M | Platelet activation, signaling and aggregation (REACTOME) | 0.004575633473 | 4.434344027 |
| GSE10640 H | signal transduction involved in mitotic G2 DNA damage checkpoint | 0.04865480108 | 207.1774194 |
| GSE10640 H | positive regulation of T-helper 2 cell differentiation | 0.02637143573 | 62.55681818 |
| GSE10640 H | response to wounding | 0.01886228231 | 2.240001395 |
| GSE10640 H | immune response | 0.01851982251 | 1.672830388 |
| GSE10640 H | response to stimulus | 0.01829956813 | 1.22671905 |
| GSE10640 H | immune system process | 0.01796314355 | 1.51954859 |
| GSE10640 H | defense response | 0.01134695134 | 1.790671738 |
| GSE10640 H | response to stress | 0.0001715784617 | 1.546536776 |
| GSE10640 H | cytoplasm | 8.51E-07 | 1.14619731 |
| GSE10640 H | cell activation | 6.43E-07 | 1.68747303 |
| GSE10640 H | leukocyte activation | 2.94E-07 | 1.757872001 |
| GSE10640 H | regulation of immune system process | 1.20E-07 | 1.70114595 |
| GSE10640 H | immune response | 1.98E-08 | 1.625126518 |
| GSE10640 H | immune system process | 5.83E-09 | 1.498066016 |

Supplementary Data 3

| Datasets | total | Elements Entrez |
| --- | --- | --- |
| GSE104121M GSE10640H GSE10640M GSE6874H GSE6874M | 5 | 4332 |
|  |  | 8676 |
|  |  | 3702 |
|  |  | 85464 |
|  |  | 3857 |
| GSE10640H GSE10640M GSE6874H GSE6874M | 5 | 1843 |
|  |  | 79717 |
|  |  | 7535 |
|  |  | 10622 |
|  |  | 2268 |
| GSE104121M GSE10640H GSE10640M GSE6874H | 32 | 374403 |
|  |  | 89891 |
|  |  | 7409 |
|  |  | 4176 |
|  |  | 5777 |
|  |  | 3716 |
|  |  | 11157 |
|  |  | 1844 |
|  |  | 7018 |
|  |  | 25970 |
|  |  | 54206 |
|  |  | 23406 |
|  |  | 51203 |
|  |  | 914 |
|  |  | 55176 |
|  |  | 1236 |
|  |  | 5341 |
|  |  | 7305 |
|  |  | 10130 |
|  |  | 64333 |
|  |  | 5880 |
|  |  | 23133 |
|  |  | 653361 |
|  |  | 976 |
|  |  | 4071 |
|  |  | 8497 |
|  |  | 23382 |
|  |  | 80774 |
|  |  | 6850 |
|  |  | 83442 |
|  |  | 5580 |
|  |  | 3321 |
| GSE104121M GSE10640H GSE10640M GSE6874M | 2 | 221476 |
|  |  | 80135 |
| GSE104121M GSE10640H GSE6874H GSE6874M | 15 | 283899 |
|  |  | 6100 |
|  |  | 5199 |
|  |  | 706 |
|  |  | 84650 |
|  |  | 226 |
|  |  | 10109 |
|  |  | 84106 |
|  |  | 301 |
|  |  | 9445 |
|  |  | 529 |
|  |  | 51177 |
|  |  | 9214 |
|  |  | 6993 |
|  |  | 4792 |
| GSE104121M GSE10640M GSE6874H GSE6874M | 4 | 51530 |
|  |  | 7941 |
|  |  | 81565 |
|  |  | 1831 |
| GSE10640H GSE10640M GSE6874H | 99 | 5825 |
|  |  | 55055 |
|  |  | 58472 |
|  |  | 57221 |
|  |  | 563 |
|  |  | 3832 |
|  |  | 3880 |
|  |  | 79781 |
|  |  | 6404 |
|  |  | 94032 |
|  |  | 83540 |
|  |  | 4015 |
|  |  | 51669 |
|  |  | 90288 |
|  |  | 9532 |
|  |  | 57526 |
|  |  | 3875 |
|  |  | 9069 |
|  |  | 1393 |
|  |  | 27141 |
|  |  | 898 |
|  |  | 1728 |
|  |  | 11167 |
|  |  | 10803 |
|  |  | 5425 |
|  |  | 4089 |
|  |  | 9787 |
|  |  | 83543 |
|  |  | 3394 |
|  |  | 89886 |
|  |  | 30968 |
|  |  | 833 |
|  |  | 10541 |
|  |  | 1466 |
|  |  | 4154 |
|  |  | 50617 |
|  |  | 2357 |
|  |  | 22868 |
|  |  | 57655 |
|  |  | 116225 |
|  |  | 123169 |
|  |  | 29099 |
|  |  | 3033 |
|  |  | 478 |
|  |  | 115817 |
|  |  | 57614 |
|  |  | 84515 |
|  |  | 54875 |
|  |  | 29088 |
|  |  | 23598 |
|  |  | 26586 |
|  |  | 120425 |
|  |  | 1063 |
|  |  | 23765 |
|  |  | 2200 |
|  |  | 29093 |
|  |  | 10615 |
|  |  | 222826 |
|  |  | 1462 |
|  |  | 4542 |
|  |  | 5551 |
|  |  | 821 |
|  |  | 2674 |
|  |  | 6790 |
|  |  | 11041 |
|  |  | 4085 |
|  |  | 55701 |
|  |  | 10351 |
|  |  | 699 |
|  |  | 891 |
|  |  | 1503 |
|  |  | 50856 |
|  |  | 23203 |
|  |  | 55916 |
|  |  | 1278 |
|  |  | 22801 |
|  |  | 221 |
|  |  | 4547 |
|  |  | 10635 |
|  |  | 475 |
|  |  | 5176 |
|  |  | 3965 |
|  |  | 1841 |
|  |  | 129401 |
|  |  | 84940 |
|  |  | 240 |
|  |  | 5090 |
|  |  | 28998 |
|  |  | 6566 |
|  |  | 1515 |
|  |  | 5284 |
|  |  | 57648 |
|  |  | 2622 |
|  |  | 4837 |
|  |  | 2147 |
|  |  | 23248 |
|  |  | 4175 |
|  |  | 57508 |
|  |  | 4487 |
| GSE10640H GSE10640M GSE6874M | 6 | 11215 |
|  |  | 150094 |
|  |  | 8895 |
|  |  | 3396 |
|  |  | 79752 |
|  |  | 80324 |
| GSE104121M GSE10640H GSE10640M | 27 | 4046 |
|  |  | 1019 |
|  |  | 10084 |
|  |  | 80700 |
|  |  | 6622 |
|  |  | 58495 |
|  |  | 9173 |
|  |  | 729830 |
|  |  | 10567 |
|  |  | 10576 |
|  |  | 56033 |
|  |  | 84935 |
|  |  | 29926 |
|  |  | 23200 |
|  |  | 51302 |
|  |  | 6886 |
|  |  | 4738 |
|  |  | 1441 |
|  |  | 2235 |
|  |  | 64231 |
|  |  | 393 |
|  |  | 90273 |
|  |  | 1573 |
|  |  | 208 |
|  |  | 474344 |
|  |  | 10367 |
|  |  | 201973 |
| GSE10640H GSE6874H GSE6874M | 51 | 83464 |
|  |  | 90313 |
|  |  | 168537 |
|  |  | 51574 |
|  |  | 56302 |
|  |  | 57559 |
|  |  | 55080 |
|  |  | 1880 |
|  |  | 4063 |
|  |  | 93081 |
|  |  | 10142 |
|  |  | 66000 |
|  |  | 54537 |
|  |  | 493911 |
|  |  | 83787 |
|  |  | 1026 |
|  |  | 5095 |
|  |  | 22806 |
|  |  | 54874 |
|  |  | 2542 |
|  |  | 29909 |
|  |  | 16 |
|  |  | 9026 |
|  |  | 146206 |
|  |  | 89790 |
|  |  | 3899 |
|  |  | 22877 |
|  |  | 5188 |
|  |  | 4686 |
|  |  | 3397 |
|  |  | 9188 |
|  |  | 84679 |
|  |  | 84259 |
|  |  | 3708 |
|  |  | 11137 |
|  |  | 29997 |
|  |  | 912 |
|  |  | 64919 |
|  |  | 1521 |
|  |  | 23533 |
|  |  | 7424 |
|  |  | 26207 |
|  |  | 219931 |
|  |  | 23020 |
|  |  | 572 |
|  |  | 55920 |
|  |  | 54532 |
|  |  | 118788 |
|  |  | 57498 |
|  |  | 7264 |
|  |  | 8525 |
| GSE104121M GSE10640H GSE6874H | 136 | 686 |
|  |  | 64844 |
|  |  | 3851 |
|  |  | 8754 |
|  |  | 9973 |
|  |  | 9401 |
|  |  | 26289 |
|  |  | 9543 |
|  |  | 25978 |
|  |  | 11333 |
|  |  | 26045 |
|  |  | 3015 |
|  |  | 130271 |
|  |  | 326 |
|  |  | 6660 |
|  |  | 26973 |
|  |  | 8509 |
|  |  | 1791 |
|  |  | 57176 |
|  |  | 336 |
|  |  | 9770 |
|  |  | 5216 |
|  |  | 5901 |
|  |  | 6503 |
|  |  | 58527 |
|  |  | 3603 |
|  |  | 64785 |
|  |  | 22862 |
|  |  | 10871 |
|  |  | 10870 |
|  |  | 4814 |
|  |  | 23596 |
|  |  | 196383 |
|  |  | 84817 |
|  |  | 6059 |
|  |  | 5695 |
|  |  | 27258 |
|  |  | 23582 |
|  |  | 10957 |
|  |  | 10235 |
|  |  | 9141 |
|  |  | 91272 |
|  |  | 55071 |
|  |  | 22796 |
|  |  | 2539 |
|  |  | 9114 |
|  |  | 963 |
|  |  | 9776 |
|  |  | 9868 |
|  |  | 1152 |
|  |  | 221458 |
|  |  | 1536 |
|  |  | 56990 |
|  |  | 29974 |
|  |  | 51566 |
|  |  | 7374 |
|  |  | 3720 |
|  |  | 6668 |
|  |  | 85019 |
|  |  | 4060 |
|  |  | 55014 |
|  |  | 391 |
|  |  | 3241 |
|  |  | 8540 |
|  |  | 50848 |
|  |  | 6628 |
|  |  | 8997 |
|  |  | 4478 |
|  |  | 79827 |
|  |  | 3119 |
|  |  | 2909 |
|  |  | 64581 |
|  |  | 1024 |
|  |  | 3273 |
|  |  | 8878 |
|  |  | 26135 |
|  |  | 8601 |
|  |  | 79156 |
|  |  | 3570 |
|  |  | 213 |
|  |  | 53919 |
|  |  | 326624 |
|  |  | 51510 |
|  |  | 7351 |
|  |  | 23478 |
|  |  | 79685 |
|  |  | 7070 |
|  |  | 6574 |
|  |  | 132160 |
|  |  | 1979 |
|  |  | 5495 |
|  |  | 4731 |
|  |  | 4726 |
|  |  | 8833 |
|  |  | 55127 |
|  |  | 27299 |
|  |  | 22836 |
|  |  | 51386 |
|  |  | 1381 |
|  |  | 11235 |
|  |  | 51526 |
|  |  | 29761 |
|  |  | 93487 |
|  |  | 9051 |
|  |  | 10551 |
|  |  | 115362 |
|  |  | 10535 |
|  |  | 10116 |
|  |  | 200186 |
|  |  | 2159 |
|  |  | 3059 |
|  |  | 26011 |
|  |  | 4017 |
|  |  | 3684 |
|  |  | 9414 |
|  |  | 128346 |
|  |  | 85444 |
|  |  | 6554 |
|  |  | 9784 |
|  |  | 10620 |
|  |  | 3159 |
|  |  | 3417 |
|  |  | 84962 |
|  |  | 79622 |
|  |  | 8775 |
|  |  | 3101 |
|  |  | 6279 |
|  |  | 5998 |
|  |  | 64066 |
|  |  | 64407 |
|  |  | 4289 |
|  |  | 57140 |
|  |  | 1043 |
|  |  | 57610 |
|  |  | 9032 |
|  |  | 113878 |
| GSE104121M GSE10640H GSE6874M | 12 | 84240 |
|  |  | 57396 |
|  |  | 55466 |
|  |  | 64840 |
|  |  | 10657 |
|  |  | 128866 |
|  |  | 6597 |
|  |  | 25906 |
|  |  | 25989 |
|  |  | 23568 |
|  |  | 10892 |
|  |  | 60412 |
| GSE10640M GSE6874H GSE6874M | 5 | 7097 |
|  |  | 7096 |
|  |  | 284996 |
|  |  | 54765 |
|  |  | 1794 |
| GSE104121M GSE10640M GSE6874H | 25 | 26469 |
|  |  | 27101 |
|  |  | 6319 |
|  |  | 408 |
|  |  | 164668 |
|  |  | 10935 |
|  |  | 2793 |
|  |  | 340205 |
|  |  | 3161 |
|  |  | 55108 |
|  |  | 56063 |
|  |  | 55764 |
|  |  | 8407 |
|  |  | 51316 |
|  |  | 3384 |
|  |  | 951 |
|  |  | 81622 |
|  |  | 51225 |
|  |  | 50650 |
|  |  | 128864 |
|  |  | 274 |
|  |  | 339105 |
|  |  | 145226 |
|  |  | 220002 |
|  |  | 84807 |
| GSE104121M GSE10640M GSE6874M | 32 |  |
|  |  | 83548 |
|  |  | 23404 |
|  |  | 998 |
|  |  | 85363 |
|  |  | 84034 |
|  |  | 55182 |
|  |  | 81488 |
|  |  | 116844 |
|  |  | 6888 |
|  |  | 27250 |
|  |  | 6300 |
|  |  | 50804 |
|  |  | 4502 |
|  |  | 8761 |
|  |  | 1475 |
|  |  | 1476 |
|  |  | 3455 |
|  |  | 79723 |
|  |  | 64005 |
|  |  | 51466 |
|  |  | 9516 |
|  |  | 23355 |
|  |  | 8303 |
|  |  | 10875 |
|  |  | 7431 |
|  |  | 832 |
|  |  | 5496 |
|  |  | 945 |
|  |  | 338339 |
|  |  | 10049 |
|  |  | 231 |
| GSE104121M GSE6874H GSE6874M | 20 | 917 |
|  |  | 1368 |
|  |  | 27163 |
|  |  | 65003 |
|  |  | 1992 |
|  |  | 4715 |
|  |  | 643 |
|  |  | 29889 |
|  |  | 55283 |
|  |  | 5734 |
|  |  | 11337 |
|  |  | 10325 |
|  |  | 6237 |
|  |  | 10482 |
|  |  | 29101 |
|  |  | 684 |
|  |  | 7555 |
|  |  | 79962 |
|  |  | 4061 |
|  |  | 9929 |
| GSE10640H GSE10640M | 126 | 29 |
|  |  | 200844 |
|  |  | 7466 |
|  |  | 79874 |
|  |  | 5713 |
|  |  | 55197 |
|  |  | 8029 |
|  |  | 2037 |
|  |  | 2665 |
|  |  | 58508 |
|  |  | 2103 |
|  |  | 170954 |
|  |  | 11144 |
|  |  | 221074 |
|  |  | 94160 |
|  |  | 56938 |
|  |  | 65263 |
|  |  | 50615 |
|  |  | 2775 |
|  |  | 5801 |
|  |  | 10240 |
|  |  | 25841 |
|  |  | 4199 |
|  |  | 2954 |
|  |  | 26084 |
|  |  | 5118 |
|  |  | 8520 |
|  |  | 10569 |
|  |  | 55671 |
|  |  | 90102 |
|  |  | 3172 |
|  |  | 23373 |
|  |  | 9131 |
|  |  | 10539 |
|  |  | 64168 |
|  |  | 9368 |
|  |  | 83861 |
|  |  | 23284 |
|  |  | 54995 |
|  |  | 114990 |
|  |  | 200172 |
|  |  | 10763 |
|  |  | 4833 |
|  |  | 9568 |
|  |  | 8425 |
|  |  | 6756 |
|  |  | 5985 |
|  |  | 6119 |
|  |  | 29888 |
|  |  | 2967 |
|  |  | 11226 |
|  |  | 10335 |
|  |  | 255520 |
|  |  | 1237 |
|  |  | 5037 |
|  |  | 57172 |
|  |  | 51678 |
|  |  | 11004 |
|  |  | 79906 |
|  |  | 152110 |
|  |  | 6555 |
|  |  | 10478 |
|  |  | 6934 |
|  |  | 79415 |
|  |  | 3339 |
|  |  | 57599 |
|  |  | 11098 |
|  |  | 4719 |
|  |  | 5764 |
|  |  | 8943 |
|  |  | 1588 |
|  |  | 3312 |
|  |  | 6725 |
|  |  | 27121 |
|  |  | 134266 |
|  |  | 4751 |
|  |  | 55013 |
|  |  | 862 |
|  |  | 55689 |
|  |  | 54625 |
|  |  | 349565 |
|  |  | 25885 |
|  |  | 286077 |
|  |  | 113510 |
|  |  | 9942 |
|  |  | 85476 |
|  |  | 112399 |
|  |  | 9829 |
|  |  | 9245 |
|  |  | 159195 |
|  |  | 5420 |
|  |  | 6608 |
|  |  | 10454 |
|  |  | 65012 |
|  |  | 29105 |
|  |  | 139212 |
|  |  | 5609 |
|  |  | 93426 |
|  |  | 255743 |
|  |  | 5347 |
|  |  | 2780 |
|  |  | 55544 |
|  |  | 26284 |
|  |  | 7091 |
|  |  | 56181 |
|  |  | 11107 |
|  |  | 64111 |
|  |  | 51023 |
|  |  | 4882 |
|  |  | 10667 |
|  |  | 7035 |
|  |  | 4008 |
|  |  | 92703 |
|  |  | 3357 |
|  |  | 11076 |
|  |  | 284297 |
|  |  | 90529 |
|  |  | 6476 |
|  |  | 114131 |
|  |  | 8840 |
|  |  | 1281 |
|  |  | 7776 |
|  |  | 79983 |
|  |  | 83734 |
|  |  | 79053 |
|  |  | 56914 |
| GSE10640H GSE6874H | 642 | 5521 |
|  |  | 352909 |
|  |  | 3957 |
|  |  | 9126 |
|  |  | 5464 |
|  |  | 3824 |
|  |  | 55859 |
|  |  | 283297 |
|  |  | 10611 |
|  |  | 85349 |
|  |  | 3149 |
|  |  | 84945 |
|  |  | 2705 |
|  |  | 4144 |
|  |  | 26234 |
|  |  | 93210 |
|  |  | 80313 |
|  |  | 5324 |
|  |  | 10238 |
|  |  | 64777 |
|  |  | 89910 |
|  |  | 55906 |
|  |  | 79005 |
|  |  | 84618 |
|  |  | 8462 |
|  |  | 10256 |
|  |  | 3612 |
|  |  | 10161 |
|  |  | 9133 |
|  |  | 4923 |
|  |  | 23632 |
|  |  | 3694 |
|  |  | 5288 |
|  |  | 4115 |
|  |  | 11046 |
|  |  | 84886 |
|  |  | 8723 |
|  |  | 493856 |
|  |  | 55454 |
|  |  | 8910 |
|  |  | 2295 |
|  |  | 57630 |
|  |  | 10550 |
|  |  | 64332 |
|  |  | 3903 |
|  |  | 56158 |
|  |  | 6242 |
|  |  | 5625 |
|  |  | 2886 |
|  |  | 7159 |
|  |  | 55507 |
|  |  | 26085 |
|  |  | 256691 |
|  |  | 7262 |
|  |  | 5961 |
|  |  | 85406 |
|  |  | 27111 |
|  |  | 219995 |
|  |  | 4076 |
|  |  | 9790 |
|  |  | 55690 |
|  |  | 9665 |
|  |  | 221091 |
|  |  | 79738 |
|  |  | 114134 |
|  |  | 5369 |
|  |  | 10575 |
|  |  | 6792 |
|  |  | 10498 |
|  |  | 84124 |
|  |  | 255488 |
|  |  | 10287 |
|  |  | 9622 |
|  |  | 84706 |
|  |  | 50939 |
|  |  | 2125 |
|  |  | 221711 |
|  |  | 10487 |
|  |  | 63036 |
|  |  | 1164 |
|  |  | 79731 |
|  |  | 51691 |
|  |  | 55741 |
|  |  | 401285 |
|  |  | 51764 |
|  |  | 57585 |
|  |  | 3601 |
|  |  | 26160 |
|  |  | 90634 |
|  |  | 8546 |
|  |  | 64174 |
|  |  | 6529 |
|  |  | 3487 |
|  |  | 8894 |
|  |  | 10112 |
|  |  | 1429 |
|  |  | 55760 |
|  |  | 9263 |
|  |  | 2212 |
|  |  | 10047 |
|  |  | 3937 |
|  |  | 4620 |
|  |  | 284004 |
|  |  | 25937 |
|  |  | 128853 |
|  |  | 151835 |
|  |  | 254552 |
|  |  | 285172 |
|  |  | 3360 |
|  |  | 10391 |
|  |  | 10969 |
|  |  | 1181 |
|  |  | 245928 |
|  |  | 56979 |
|  |  | 51304 |
|  |  | 84528 |
|  |  | 7139 |
|  |  | 5424 |
|  |  | 116832 |
|  |  | 151313 |
|  |  | 83857 |
|  |  | 3593 |
|  |  | 133957 |
|  |  | 5709 |
|  |  | 9162 |
|  |  | 81493 |
|  |  | 93134 |
|  |  | 8038 |
|  |  | 127124 |
|  |  | 1360 |
|  |  | 23526 |
|  |  | 23624 |
|  |  | 9317 |
|  |  | 1737 |
|  |  | 2262 |
|  |  | 9498 |
|  |  | 8477 |
|  |  | 116211 |
|  |  | 202915 |
|  |  | 6650 |
|  |  | 5256 |
|  |  | 6988 |
|  |  | 866 |
|  |  | 84525 |
|  |  | 199713 |
|  |  | 183 |
|  |  | 390162 |
|  |  | 26577 |
|  |  | 7709 |
|  |  | 6241 |
|  |  | 50515 |
|  |  | 79856 |
|  |  | 114926 |
|  |  | 2492 |
|  |  | 220213 |
|  |  | 100130264 |
|  |  | 90485 |
|  |  | 221443 |
|  |  | 51651 |
|  |  | 401505 |
|  |  | 84639 |
|  |  | 27131 |
|  |  | 55028 |
|  |  | 64963 |
|  |  | 256227 |
|  |  | 54967 |
|  |  | 4782 |
|  |  | 11159 |
|  |  | 154791 |
|  |  | 79102 |
|  |  | 144321 |
|  |  | 3706 |
|  |  | 56547 |
|  |  | 10743 |
|  |  | 286451 |
|  |  | 22863 |
|  |  | 1000 |
|  |  | 2582 |
|  |  | 415 |
|  |  | 2597 |
|  |  | 60685 |
|  |  | 63897 |
|  |  | 6637 |
|  |  | 4619 |
|  |  | 9509 |
|  |  | 2528 |
|  |  | 5506 |
|  |  | 4493 |
|  |  | 54913 |
|  |  | 1285 |
|  |  | 64780 |
|  |  | 245938 |
|  |  | 134492 |
|  |  | 3300 |
|  |  | 3337 |
|  |  | 5322 |
|  |  | 2243 |
|  |  | 5654 |
|  |  | 80758 |
|  |  | 114770 |
|  |  | 6795 |
|  |  | 81889 |
|  |  | 64151 |
|  |  | 23126 |
|  |  | 8828 |
|  |  | 23576 |
|  |  | 7852 |
|  |  | 124446 |
|  |  | 5414 |
|  |  | 5562 |
|  |  | 27065 |
|  |  | 7436 |
|  |  | 256643 |
|  |  | 9938 |
|  |  | 7579 |
|  |  | 51367 |
|  |  | 6547 |
|  |  | 2139 |
|  |  | 287015 |
|  |  | 9793 |
|  |  | 55159 |
|  |  | 29968 |
|  |  | 10241 |
|  |  | 57017 |
|  |  | 608 |
|  |  | 9833 |
|  |  | 6036 |
|  |  | 23760 |
|  |  | 161247 |
|  |  | 80347 |
|  |  | 51268 |
|  |  | 4659 |
|  |  | 771 |
|  |  | 51317 |
|  |  | 1456 |
|  |  | 10631 |
|  |  | 6707 |
|  |  | 22824 |
|  |  | 156 |
|  |  | 864 |
|  |  | 1659 |
|  |  | 767 |
|  |  | 2173 |
|  |  | 55867 |
|  |  | 1768 |
|  |  | 285313 |
|  |  | 143689 |
|  |  | 10592 |
|  |  | 54842 |
|  |  | 5955 |
|  |  | 9768 |
|  |  | 388701 |
|  |  | 148231 |
|  |  | 63875 |
|  |  | 3615 |
|  |  | 25977 |
|  |  | 6297 |
|  |  | 8883 |
|  |  | 84722 |
|  |  | 348654 |
|  |  | 283431 |
|  |  | 5788 |
|  |  | 5371 |
|  |  | 6357 |
|  |  | 51299 |
|  |  | 3621 |
|  |  | 983 |
|  |  | 54742 |
|  |  | 9261 |
|  |  | 2803 |
|  |  | 9843 |
|  |  | 254268 |
|  |  | 10609 |
|  |  | 5872 |
|  |  | 112483 |
|  |  | 2653 |
|  |  | 134285 |
|  |  | 10930 |
|  |  | 7388 |
|  |  | 409 |
|  |  | 9569 |
|  |  | 340348 |
|  |  | 6683 |
|  |  | 50805 |
|  |  | 22845 |
|  |  | 871 |
|  |  | 60560 |
|  |  | 56981 |
|  |  | 56894 |
|  |  | 26575 |
|  |  | 6422 |
|  |  | 140609 |
|  |  | 84868 |
|  |  | 2852 |
|  |  | 261734 |
|  |  | 3310 |
|  |  | 127281 |
|  |  | 9777 |
|  |  | 27034 |
|  |  | 84928 |
|  |  | 9791 |
|  |  | 79022 |
|  |  | 57643 |
|  |  | 22948 |
|  |  | 7291 |
|  |  | 60385 |
|  |  | 29901 |
|  |  | 121355 |
|  |  | 29028 |
|  |  | 54876 |
|  |  | 83851 |
|  |  | 2266 |
|  |  | 517 |
|  |  | 253725 |
|  |  | 51151 |
|  |  | 834 |
|  |  | 7345 |
|  |  | 2194 |
|  |  | 54948 |
|  |  | 159963 |
|  |  | 92922 |
|  |  | 10675 |
|  |  | 80341 |
|  |  | 6855 |
|  |  | 55065 |
|  |  | 64946 |
|  |  | 7364 |
|  |  | 2168 |
|  |  | 875 |
|  |  | 136 |
|  |  | 84717 |
|  |  | 55244 |
|  |  | 10428 |
|  |  | 83650 |
|  |  | 64979 |
|  |  | 6573 |
|  |  | 91646 |
|  |  | 4772 |
|  |  | 3364 |
|  |  | 653689 |
|  |  | 440387 |
|  |  | 138065 |
|  |  | 5196 |
|  |  | 60681 |
|  |  | 93953 |
|  |  | 85415 |
|  |  | 55824 |
|  |  | 112942 |
|  |  | 90488 |
|  |  | 55070 |
|  |  | 501 |
|  |  | 6175 |
|  |  | 85440 |
|  |  | 7227 |
|  |  | 35 |
|  |  | 114876 |
|  |  | 9056 |
|  |  | 3713 |
|  |  | 9989 |
|  |  | 4436 |
|  |  | 84262 |
|  |  | 256236 |
|  |  | 2801 |
|  |  | 57699 |
|  |  | 11161 |
|  |  | 94274 |
|  |  | 7568 |
|  |  | 10913 |
|  |  | 10668 |
|  |  | 79180 |
|  |  | 1827 |
|  |  | 10529 |
|  |  | 9121 |
|  |  | 10023 |
|  |  | 51645 |
|  |  | 200232 |
|  |  | 84230 |
|  |  | 55352 |
|  |  | 84283 |
|  |  | 84660 |
|  |  | 908 |
|  |  | 7292 |
|  |  | 284 |
|  |  | 9709 |
|  |  | 1104 |
|  |  | 28978 |
|  |  | 79642 |
|  |  | 8290 |
|  |  | 57592 |
|  |  | 55450 |
|  |  | 2560 |
|  |  | 81926 |
|  |  | 10540 |
|  |  | 9258 |
|  |  | 51232 |
|  |  | 597 |
|  |  | 25884 |
|  |  | 25770 |
|  |  | 5950 |
|  |  | 1506 |
|  |  | 55076 |
|  |  | 51281 |
|  |  | 4651 |
|  |  | 6775 |
|  |  | 9489 |
|  |  | 23132 |
|  |  | 7342 |
|  |  | 11013 |
|  |  | 6941 |
|  |  | 153396 |
|  |  | 10799 |
|  |  | 56834 |
|  |  | 51495 |
|  |  | 51319 |
|  |  | 84513 |
|  |  | 254102 |
|  |  | 84689 |
|  |  | 23517 |
|  |  | 4907 |
|  |  | 5058 |
|  |  | 254528 |
|  |  | 58493 |
|  |  | 120775 |
|  |  | 91526 |
|  |  | 27287 |
|  |  | 90226 |
|  |  | 51738 |
|  |  | 25816 |
|  |  | 11214 |
|  |  | 284110 |
|  |  | 51362 |
|  |  | 89941 |
|  |  | 51728 |
|  |  | 221938 |
|  |  | 79576 |
|  |  | 80119 |
|  |  | 200576 |
|  |  | 3385 |
|  |  | 2058 |
|  |  | 2877 |
|  |  | 23329 |
|  |  | 9204 |
|  |  | 5983 |
|  |  | 50853 |
|  |  | 57551 |
|  |  | 79682 |
|  |  | 83449 |
|  |  | 7805 |
|  |  | 84451 |
|  |  | 10468 |
|  |  | 8996 |
|  |  | 22853 |
|  |  | 128240 |
|  |  | 132864 |
|  |  | 5763 |
|  |  | 58477 |
|  |  | 9465 |
|  |  | 86 |
|  |  | 147808 |
|  |  | 10655 |
|  |  | 222663 |
|  |  | 23239 |
|  |  | 10024 |
|  |  | 873 |
|  |  | 3099 |
|  |  | 55351 |
|  |  | 201134 |
|  |  | 7429 |
|  |  | 90019 |
|  |  | 8317 |
|  |  | 1163 |
|  |  | 81035 |
|  |  | 10361 |
|  |  | 23037 |
|  |  | 23524 |
|  |  | 634 |
|  |  | 64098 |
|  |  | 10712 |
|  |  | 84747 |
|  |  | 222389 |
|  |  | 7082 |
|  |  | 5146 |
|  |  | 400961 |
|  |  | 84814 |
|  |  | 2999 |
|  |  | 79969 |
|  |  | 202309 |
|  |  | 5986 |
|  |  | 79658 |
|  |  | 147339 |
|  |  | 84798 |
|  |  | 64755 |
|  |  | 127943 |
|  |  | 405753 |
|  |  | 136332 |
|  |  | 7133 |
|  |  | 7162 |
|  |  | 29995 |
|  |  | 3113 |
|  |  | 1646 |
|  |  | 9061 |
|  |  | 55336 |
|  |  | 23082 |
|  |  | 56300 |
|  |  | 137835 |
|  |  | 51244 |
|  |  | 55768 |
|  |  | 55323 |
|  |  | 9515 |
|  |  | 55090 |
|  |  | 91833 |
|  |  | 10650 |
|  |  | 83888 |
|  |  | 200315 |
|  |  | 26092 |
|  |  | 51278 |
|  |  | 57829 |
|  |  | 114132 |
|  |  | 10647 |
|  |  | 2135 |
|  |  | 84727 |
|  |  | 65217 |
|  |  | 23042 |
|  |  | 23657 |
|  |  | 10208 |
|  |  | 5376 |
|  |  | 91607 |
|  |  | 57161 |
|  |  | 388394 |
|  |  | 203547 |
|  |  | 51148 |
|  |  | 10978 |
|  |  | 284382 |
|  |  | 780 |
|  |  | 2209 |
|  |  | 22941 |
|  |  | 119687 |
|  |  | 51230 |
|  |  | 388730 |
|  |  | 7855 |
|  |  | 345778 |
|  |  | 54436 |
|  |  | 313 |
|  |  | 140901 |
|  |  | 130540 |
|  |  | 285175 |
|  |  | 158427 |
|  |  | 28987 |
|  |  | 140462 |
|  |  | 8178 |
|  |  | 5277 |
|  |  | 285381 |
|  |  | 516 |
|  |  | 1813 |
|  |  | 125206 |
|  |  | 55118 |
|  |  | 7371 |
|  |  | 1081 |
|  |  | 60481 |
|  |  | 9533 |
|  |  | 654817 |
|  |  | 79841 |
|  |  | 4485 |
|  |  | 8092 |
|  |  | 5053 |
|  |  | 11236 |
|  |  | 8741 |
|  |  | 23038 |
|  |  | 10095 |
|  |  | 2941 |
|  |  | 5132 |
|  |  | 64799 |
|  |  | 56999 |
|  |  | 130813 |
|  |  | 9338 |
|  |  | 284451 |
|  |  | 6443 |
|  |  | 7128 |
|  |  | 8727 |
|  |  | 154386 |
|  |  | 29945 |
|  |  | 9315 |
|  |  | 80013 |
|  |  | 9898 |
|  |  | 64943 |
|  |  | 197 |
|  |  | 57707 |
|  |  | 112464 |
|  |  | 54979 |
|  |  | 92667 |
|  |  | 3249 |
|  |  | 65109 |
|  |  | 494514 |
|  |  | 11181 |
|  |  | 610 |
|  |  | 441543 |
|  |  | 57701 |
|  |  | 9170 |
|  |  | 81037 |
|  |  | 1326 |
|  |  | 22849 |
|  |  | 3837 |
|  |  | 373856 |
|  |  | 23288 |
|  |  | 26033 |
|  |  | 90990 |
|  |  | 51149 |
|  |  | 9212 |
|  |  | 3703 |
|  |  | 5784 |
|  |  | 117289 |
|  |  | 2561 |
|  |  | 145567 |
|  |  | 55512 |
|  |  | 6296 |
|  |  | 9500 |
|  |  | 53833 |
|  |  | 9805 |
|  |  | 4645 |
|  |  | 222894 |
|  |  | 9947 |
|  |  | 4306 |
|  |  | 28988 |
|  |  | 100129540 |
|  |  | 10276 |
|  |  |  |
|  |  | 4236 |
|  |  | 5746 |
|  |  | 10465 |
|  |  | 54892 |
|  |  | 91752 |
|  |  | 2252 |
|  |  | 10861 |
|  |  | 5010 |
|  |  | 146556 |
|  |  | 25799 |
|  |  | 1366 |
|  |  | 84073 |
|  |  | 6635 |
|  |  | 51454 |
|  |  | 2215 |
|  |  | 1369 |
|  |  | 3698 |
| GSE10640H GSE6874M | 98 | 3185 |
|  |  | 9961 |
|  |  | 84298 |
|  |  | 55527 |
|  |  | 8776 |
|  |  | 147798 |
|  |  | 10450 |
|  |  | 23216 |
|  |  | 57448 |
|  |  | 54439 |
|  |  | 22827 |
|  |  | 80821 |
|  |  | 56919 |
|  |  | 80323 |
|  |  | 79798 |
|  |  | 79133 |
|  |  | 64210 |
|  |  | 23300 |
|  |  | 3373 |
|  |  | 84954 |
|  |  | 23341 |
|  |  | 10758 |
|  |  | 80255 |
|  |  | 84289 |
|  |  | 162494 |
|  |  | 28976 |
|  |  | 80204 |
|  |  | 9875 |
|  |  | 8602 |
|  |  | 6731 |
|  |  | 25900 |
|  |  | 197358 |
|  |  | 84516 |
|  |  | 7148 |
|  |  | 8266 |
|  |  | 203069 |
|  |  | 55974 |
|  |  | 644353 |
|  |  | 5509 |
|  |  | 4437 |
|  |  | 79158 |
|  |  | 55892 |
|  |  | 5664 |
|  |  | 80335 |
|  |  | 8412 |
|  |  | 10714 |
|  |  | 26512 |
|  |  | 79843 |
|  |  | 84532 |
|  |  | 1822 |
|  |  | 1743 |
|  |  | 6832 |
|  |  | 51368 |
|  |  | 90362 |
|  |  | 115727 |
|  |  | 85441 |
|  |  | 6605 |
|  |  | 83637 |
|  |  | 23476 |
|  |  | 54939 |
|  |  | 29970 |
|  |  | 6722 |
|  |  | 83746 |
|  |  | 81602 |
|  |  | 9950 |
|  |  | 60528 |
|  |  | 8672 |
|  |  | 84678 |
|  |  | 51168 |
|  |  | 4013 |
|  |  | 55850 |
|  |  | 7226 |
|  |  | 64708 |
|  |  | 9047 |
|  |  | 3760 |
|  |  | 87178 |
|  |  | 8882 |
|  |  | 10081 |
|  |  | 3635 |
|  |  | 22907 |
|  |  | 54952 |
|  |  | 56647 |
|  |  | 10056 |
|  |  | 256380 |
|  |  | 8893 |
|  |  | 9183 |
|  |  | 8567 |
|  |  | 8816 |
|  |  | 730249 |
|  |  | 54663 |
|  |  | 51493 |
|  |  | 1731 |
|  |  | 60678 |
|  |  | 65985 |
|  |  | 10051 |
|  |  | 220042 |
|  |  | 51104 |
|  |  | 51010 |
| GSE104121M GSE10640H | 175 | 9333 |
|  |  | 9736 |
|  |  | 4066 |
|  |  | 573 |
|  |  | 51147 |
|  |  | 23247 |
|  |  | 57144 |
|  |  | 143662 |
|  |  | 56751 |
|  |  | 148170 |
|  |  | 1718 |
|  |  | 55092 |
|  |  | 23432 |
|  |  | 54812 |
|  |  | 4191 |
|  |  | 84336 |
|  |  | 60482 |
|  |  | 200150 |
|  |  | 56902 |
|  |  | 83982 |
|  |  | 51701 |
|  |  | 23386 |
|  |  | 84330 |
|  |  | 51341 |
|  |  | 1272 |
|  |  | 55611 |
|  |  | 137695 |
|  |  | 10100 |
|  |  | 6611 |
|  |  | 114780 |
|  |  | 1788 |
|  |  | 896 |
|  |  | 23678 |
|  |  | 246175 |
|  |  | 65264 |
|  |  | 65975 |
|  |  | 57617 |
|  |  | 25963 |
|  |  | 553115 |
|  |  | 339122 |
|  |  | 6164 |
|  |  | 3420 |
|  |  | 9144 |
|  |  | 285231 |
|  |  | 7045 |
|  |  | 23505 |
|  |  | 8417 |
|  |  | 22985 |
|  |  | 25873 |
|  |  | 5436 |
|  |  | 9726 |
|  |  | 5573 |
|  |  | 10692 |
|  |  | 29980 |
|  |  | 30844 |
|  |  | 136853 |
|  |  | 254173 |
|  |  | 64090 |
|  |  | 3301 |
|  |  | 55757 |
|  |  | 10055 |
|  |  | 83696 |
|  |  | 80759 |
|  |  | 254427 |
|  |  | 114908 |
|  |  | 11016 |
|  |  | 3338 |
|  |  | 56917 |
|  |  | 10169 |
|  |  | 51614 |
|  |  | 10298 |
|  |  | 1915 |
|  |  | 84991 |
|  |  | 50944 |
|  |  | 11188 |
|  |  | 1051 |
|  |  | 2155 |
|  |  | 23291 |
|  |  | 54776 |
|  |  | 26127 |
|  |  | 7743 |
|  |  | 79719 |
|  |  | 6193 |
|  |  | 1828 |
|  |  | 166 |
|  |  | 10618 |
|  |  | 5048 |
|  |  | 8581 |
|  |  | 9990 |
|  |  | 54934 |
|  |  | 84875 |
|  |  | 29907 |
|  |  | 27091 |
|  |  | 148137 |
|  |  | 1662 |
|  |  | 2623 |
|  |  | 257397 |
|  |  | 682 |
|  |  | 9913 |
|  |  | 890 |
|  |  | 3669 |
|  |  | 222865 |
|  |  | 1340 |
|  |  | 401548 |
|  |  | 2207 |
|  |  | 6203 |
|  |  | 57185 |
|  |  | 840 |
|  |  | 32 |
|  |  | 11070 |
|  |  | 51411 |
|  |  | 7450 |
|  |  | 367 |
|  |  | 56947 |
|  |  | 1012 |
|  |  | 2931 |
|  |  | 151636 |
|  |  | 81542 |
|  |  | 10197 |
|  |  | 4915 |
|  |  | 57127 |
|  |  | 9446 |
|  |  | 2246 |
|  |  | 54815 |
|  |  | 83606 |
|  |  | 26000 |
|  |  | 23608 |
|  |  | 10067 |
|  |  | 8604 |
|  |  | 2051 |
|  |  | 339 |
|  |  | 51123 |
|  |  | 5936 |
|  |  | 9413 |
|  |  | 26119 |
|  |  | 9978 |
|  |  | 56961 |
|  |  | 255426 |
|  |  | 23229 |
|  |  | 23523 |
|  |  | 8721 |
|  |  | 84465 |
|  |  | 3094 |
|  |  | 4069 |
|  |  | 8832 |
|  |  | 55324 |
|  |  | 2661 |
|  |  | 83475 |
|  |  | 8869 |
|  |  | 91137 |
|  |  | 7280 |
|  |  | 57515 |
|  |  | 5319 |
|  |  | 11142 |
|  |  | 144453 |
|  |  | 7253 |
|  |  | 131616 |
|  |  | 84545 |
|  |  | 26137 |
|  |  | 7804 |
|  |  | 7167 |
|  |  | 9919 |
|  |  | 64080 |
|  |  | 57404 |
|  |  | 390061 |
|  |  | 2773 |
|  |  | 283635 |
|  |  | 23586 |
|  |  | 4125 |
|  |  | 79144 |
|  |  | 10459 |
|  |  | 8720 |
|  |  | 54407 |
|  |  | 57693 |
|  |  | 8792 |
| GSE10640M GSE6874H | 106 | 51474 |
|  |  | 2274 |
|  |  | 54865 |
|  |  | 53343 |
|  |  | 54534 |
|  |  | 28981 |
|  |  | 55668 |
|  |  | 201254 |
|  |  | 4998 |
|  |  | 85458 |
|  |  | 6398 |
|  |  | 5973 |
|  |  | 84665 |
|  |  | 90 |
|  |  | 7991 |
|  |  | 5984 |
|  |  | 55227 |
|  |  | 5286 |
|  |  | 3948 |
|  |  | 3140 |
|  |  | 5372 |
|  |  | 64434 |
|  |  | 140461 |
|  |  | 10127 |
|  |  | 5871 |
|  |  | 3680 |
|  |  | 130507 |
|  |  | 79736 |
|  |  | 701 |
|  |  | 55789 |
|  |  | 7525 |
|  |  | 79774 |
|  |  | 11132 |
|  |  | 58533 |
|  |  | 51134 |
|  |  | 57326 |
|  |  | 23176 |
|  |  | 10897 |
|  |  | 9510 |
|  |  | 10036 |
|  |  | 7079 |
|  |  | 8609 |
|  |  | 10963 |
|  |  | 55669 |
|  |  | 4025 |
|  |  | 10371 |
|  |  | 124222 |
|  |  | 3820 |
|  |  | 6240 |
|  |  | 8603 |
|  |  | 4313 |
|  |  | 54841 |
|  |  | 2059 |
|  |  | 56034 |
|  |  | 10205 |
|  |  | 5074 |
|  |  | 2038 |
|  |  | 29851 |
|  |  | 10184 |
|  |  | 5049 |
|  |  | 10602 |
|  |  | 260429 |
|  |  | 5921 |
|  |  | 23181 |
|  |  | 5052 |
|  |  | 55703 |
|  |  | 56341 |
|  |  | 1544 |
|  |  | 5996 |
|  |  | 8728 |
|  |  | 4099 |
|  |  | 6198 |
|  |  | 64359 |
|  |  | 56985 |
|  |  | 51435 |
|  |  | 1525 |
|  |  | 125488 |
|  |  | 815 |
|  |  | 3034 |
|  |  | 3516 |
|  |  | 414328 |
|  |  | 51264 |
|  |  | 3547 |
|  |  | 10099 |
|  |  | 11044 |
|  |  | 146956 |
|  |  | 653784 |
|  |  | 83714 |
|  |  | 3329 |
|  |  | 7283 |
|  |  | 3762 |
|  |  | 5357 |
|  |  | 57480 |
|  |  | 765 |
|  |  | 222484 |
|  |  | 10538 |
|  |  | 5187 |
|  |  | 91782 |
|  |  | 51231 |
|  |  | 719 |
|  |  | 203260 |
|  |  | 7043 |
|  |  | 283358 |
|  |  | 10605 |
|  |  | 53405 |
|  |  | 4678 |
| GSE10640M GSE6874M | 100 | 55854 |
|  |  | 114836 |
|  |  | 7398 |
|  |  | 55102 |
|  |  | 386617 |
|  |  | 64761 |
|  |  | 100532731 |
|  |  | 4609 |
|  |  | 9088 |
|  |  | 2919 |
|  |  | 7549 |
|  |  | 3489 |
|  |  | 11184 |
|  |  | 25962 |
|  |  | 55795 |
|  |  | 11011 |
|  |  | 28984 |
|  |  | 55719 |
|  |  | 4208 |
|  |  | 254042 |
|  |  | 84433 |
|  |  | 1663 |
|  |  | 81691 |
|  |  | 2531 |
|  |  | 10200 |
|  |  | 6948 |
|  |  | 8061 |
|  |  | 2805 |
|  |  | 1488 |
|  |  | 54980 |
|  |  | 93594 |
|  |  | 8454 |
|  |  | 55911 |
|  |  | 3105 |
|  |  | 151887 |
|  |  | 3718 |
|  |  | 2475 |
|  |  | 51706 |
|  |  | 54749 |
|  |  | 4068 |
|  |  | 57047 |
|  |  | 55340 |
|  |  | 4317 |
|  |  | 9575 |
|  |  | 10672 |
|  |  | 85459 |
|  |  | 3480 |
|  |  | 375033 |
|  |  | 5360 |
|  |  | 84549 |
|  |  | 127066 |
|  |  | 23498 |
|  |  | 9760 |
|  |  | 53829 |
|  |  | 4001 |
|  |  | 148523 |
|  |  | 4938 |
|  |  | 91584 |
|  |  | 4033 |
|  |  | 79754 |
|  |  | 1493 |
|  |  | 53347 |
|  |  | 388569 |
|  |  | 163351 |
|  |  | 3250 |
|  |  | 1840 |
|  |  | 49854 |
|  |  | 6275 |
|  |  | 55922 |
|  |  | 10915 |
|  |  | 80195 |
|  |  | 9275 |
|  |  | 11232 |
|  |  | 8942 |
|  |  | 100529257 |
|  |  | 2872 |
|  |  | 83732 |
|  |  | 7127 |
|  |  | 54957 |
|  |  | 3908 |
|  |  | 80833 |
|  |  | 5297 |
|  |  | 123904 |
|  |  | 27436 |
|  |  | 2017 |
|  |  | 284366 |
|  |  | 27000 |
|  |  | 6843 |
|  |  | 3872 |
|  |  | 83716 |
|  |  | 9696 |
|  |  | 7779 |
|  |  | 1434 |
|  |  | 137964 |
|  |  | 10683 |
|  |  | 10750 |
|  |  | 3214 |
|  |  | 121268 |
|  |  | 56252 |
|  |  | 26276 |
| GSE104121M GSE10640M | 231 | 1785 |
|  |  | 152926 |
|  |  | 401409 |
|  |  | 203074 |
|  |  | 91373 |
|  |  | 51596 |
|  |  | 8993 |
|  |  | 652 |
|  |  | 27044 |
|  |  | 23295 |
|  |  | 25996 |
|  |  | 10787 |
|  |  | 55437 |
|  |  | 130162 |
|  |  | 6997 |
|  |  | 54708 |
|  |  | 7054 |
|  |  | 8848 |
|  |  | 5720 |
|  |  | 7454 |
|  |  | 5527 |
|  |  | 2791 |
|  |  | 222255 |
|  |  | 79887 |
|  |  | 54674 |
|  |  | 1535 |
|  |  | 8106 |
|  |  | 11258 |
|  |  | 344 |
|  |  | 91543 |
|  |  | 5354 |
|  |  | 85027 |
|  |  | 24145 |
|  |  | 2926 |
|  |  | 26001 |
|  |  | 2313 |
|  |  | 7784 |
|  |  | 60 |
|  |  | 51379 |
|  |  | 64598 |
|  |  | 57579 |
|  |  | 4698 |
|  |  | 3553 |
|  |  | 1088 |
|  |  | 23585 |
|  |  | 4288 |
|  |  | 5789 |
|  |  | 63922 |
|  |  | 65983 |
|  |  | 80019 |
|  |  | 1747 |
|  |  | 5019 |
|  |  | 10924 |
|  |  | 80790 |
|  |  | 6205 |
|  |  | 130075 |
|  |  | 10987 |
|  |  | 4050 |
|  |  | 163882 |
|  |  | 90952 |
|  |  | 6881 |
|  |  | 79930 |
|  |  | 9380 |
|  |  | 2752 |
|  |  | 6913 |
|  |  | 1874 |
|  |  | 147949 |
|  |  | 969 |
|  |  | 2804 |
|  |  | 7511 |
|  |  | 811 |
|  |  | 858 |
|  |  | 9457 |
|  |  | 5110 |
|  |  | 130074 |
|  |  | 26959 |
|  |  | 1512 |
|  |  | 51654 |
|  |  | 6504 |
|  |  | 397 |
|  |  | 1191 |
|  |  | 9672 |
|  |  | 132884 |
|  |  | 6001 |
|  |  | 9991 |
|  |  | 3067 |
|  |  | 9555 |
|  |  | 5837 |
|  |  | 1870 |
|  |  | 10347 |
|  |  | 151188 |
|  |  | 2617 |
|  |  | 55813 |
|  |  | 56956 |
|  |  | 29127 |
|  |  | 10379 |
|  |  | 81790 |
|  |  | 51060 |
|  |  | 5493 |
|  |  | 10223 |
|  |  | 9610 |
|  |  | 6643 |
|  |  | 121457 |
|  |  | 3055 |
|  |  | 489 |
|  |  | 56829 |
|  |  | 2534 |
|  |  | 51374 |
|  |  | 22933 |
|  |  | 51343 |
|  |  | 3001 |
|  |  | 9262 |
|  |  | 83641 |
|  |  | 10634 |
|  |  | 11344 |
|  |  | 3006 |
|  |  | 54910 |
|  |  | 5552 |
|  |  | 4352 |
|  |  | 1455 |
|  |  | 5638 |
|  |  | 51246 |
|  |  | 219972 |
|  |  | 153339 |
|  |  | 3683 |
|  |  | 131544 |
|  |  | 4109 |
|  |  | 8932 |
|  |  | 90678 |
|  |  | 187 |
|  |  | 212 |
|  |  | 6390 |
|  |  | 84302 |
|  |  | 5770 |
|  |  | 2944 |
|  |  | 112840 |
|  |  | 54344 |
|  |  | 2815 |
|  |  | 1316 |
|  |  | 84272 |
|  |  | 11255 |
|  |  | 2119 |
|  |  | 5997 |
|  |  | 5584 |
|  |  | 2629 |
|  |  | 348093 |
|  |  | 94121 |
|  |  | 1795 |
|  |  | 11266 |
|  |  | 4051 |
|  |  | 3268 |
|  |  | 135 |
|  |  | 26095 |
|  |  | 8622 |
|  |  | 93663 |
|  |  | 79772 |
|  |  | 64105 |
|  |  | 128869 |
|  |  | 5790 |
|  |  | 219402 |
|  |  | 9289 |
|  |  | 1013 |
|  |  | 3765 |
|  |  | 10959 |
|  |  | 89857 |
|  |  | 5868 |
|  |  | 121665 |
|  |  | 92241 |
|  |  | 51690 |
|  |  | 9182 |
|  |  | 2778 |
|  |  | 60343 |
|  |  | 51258 |
|  |  | 59344 |
|  |  | 2040 |
|  |  | 127544 |
|  |  | 51639 |
|  |  | 6865 |
|  |  | 27238 |
|  |  | 3796 |
|  |  | 10267 |
|  |  | 6585 |
|  |  | 10616 |
|  |  | 287 |
|  |  | 80325 |
|  |  | 11182 |
|  |  | 5154 |
|  |  | 51194 |
|  |  | 57224 |
|  |  | 56882 |
|  |  | 83699 |
|  |  | 347 |
|  |  | 865 |
|  |  | 1537 |
|  |  | 894 |
|  |  | 22913 |
|  |  | 10797 |
|  |  | 239 |
|  |  | 55203 |
|  |  | 56904 |
|  |  | 27297 |
|  |  | 1495 |
|  |  | 91304 |
|  |  | 8887 |
|  |  | 64805 |
|  |  | 25792 |
|  |  | 27159 |
|  |  | 63933 |
|  |  | 348 |
|  |  | 201931 |
|  |  | 3028 |
|  |  | 7408 |
|  |  | 54529 |
|  |  | 5244 |
|  |  | 6402 |
|  |  | 255394 |
|  |  | 9045 |
|  |  | 5054 |
|  |  | 25828 |
|  |  | 2049 |
|  |  | 89876 |
|  |  | 5210 |
|  |  | 7358 |
|  |  | 140685 |
|  |  | 80273 |
|  |  | 5076 |
|  |  | 924 |
|  |  | 55657 |
|  |  | 6916 |
|  |  | 5175 |
|  |  | 25939 |
| GSE6874H GSE6874M | 82 | 84939 |
|  |  | 55720 |
|  |  | 25909 |
|  |  | 83860 |
|  |  | 5097 |
|  |  | 285315 |
|  |  | 129138 |
|  |  | 64216 |
|  |  | 11073 |
|  |  | 7073 |
|  |  | 6632 |
|  |  | 55721 |
|  |  | 153830 |
|  |  | 6908 |
|  |  | 23310 |
|  |  | 9984 |
|  |  | 121601 |
|  |  | 57534 |
|  |  | 2022 |
|  |  | 9493 |
|  |  | 29078 |
|  |  | 10594 |
|  |  | 30011 |
|  |  | 4939 |
|  |  | 1230 |
|  |  | 79656 |
|  |  | 57466 |
|  |  | 1153 |
|  |  | 4005 |
|  |  | 92591 |
|  |  | 51071 |
|  |  | 64801 |
|  |  | 25896 |
|  |  | 57231 |
|  |  | 1607 |
|  |  | 9702 |
|  |  | 2687 |
|  |  | 79066 |
|  |  | 80017 |
|  |  | 23612 |
|  |  | 5356 |
|  |  | 91433 |
|  |  | 23438 |
|  |  | 9654 |
|  |  | 11047 |
|  |  | 117246 |
|  |  | 2308 |
|  |  | 8870 |
|  |  | 117143 |
|  |  | 4548 |
|  |  | 10155 |
|  |  | 10252 |
|  |  | 79589 |
|  |  | 54210 |
|  |  | 79713 |
|  |  | 3383 |
|  |  | 9404 |
|  |  | 399 |
|  |  | 2651 |
|  |  | 58516 |
|  |  | 79627 |
|  |  | 8443 |
|  |  | 7372 |
|  |  | 23512 |
|  |  | 55743 |
|  |  | 22984 |
|  |  | 8573 |
|  |  | 80267 |
|  |  | 3557 |
|  |  | 10480 |
|  |  | 203100 |
|  |  | 56913 |
|  |  | 8916 |
|  |  | 51154 |
|  |  | 7006 |
|  |  | 253959 |
|  |  | 54505 |
|  |  | 58986 |
|  |  | 80010 |
|  |  | 327 |
|  |  | 84861 |
|  |  | 200845 |
| GSE104121M GSE6874H | 158 | 3936 |
|  |  | 145781 |
|  |  | 9600 |
|  |  | 84266 |
|  |  | 85315 |
|  |  | 23603 |
|  |  | 9859 |
|  |  | 142940 |
|  |  | 126003 |
|  |  | 5935 |
|  |  | 2590 |
|  |  | 140886 |
|  |  | 6181 |
|  |  | 10574 |
|  |  | 1318 |
|  |  | 142891 |
|  |  | 114798 |
|  |  | 5756 |
|  |  | 64858 |
|  |  | 217 |
|  |  | 2180 |
|  |  | 51006 |
|  |  | 6741 |
|  |  | 9450 |
|  |  | 23625 |
|  |  | 375444 |
|  |  | 6271 |
|  |  | 3687 |
|  |  | 54472 |
|  |  | 83706 |
|  |  | 92799 |
|  |  | 79006 |
|  |  | 51267 |
|  |  | 2014 |
|  |  | 3459 |
|  |  | 11001 |
|  |  | 10971 |
|  |  | 6217 |
|  |  | 84101 |
|  |  | 6397 |
|  |  | 9411 |
|  |  | 123920 |
|  |  | 23118 |
|  |  | 8747 |
|  |  | 78988 |
|  |  | 7965 |
|  |  | 8636 |
|  |  | 114987 |
|  |  | 54790 |
|  |  | 84231 |
|  |  | 9441 |
|  |  | 549 |
|  |  | 84792 |
|  |  | 58506 |
|  |  | 55004 |
|  |  | 6144 |
|  |  | 83695 |
|  |  | 6652 |
|  |  | 27090 |
|  |  | 83719 |
|  |  | 3785 |
|  |  | 9381 |
|  |  | 783 |
|  |  | 4232 |
|  |  | 79748 |
|  |  | 51283 |
|  |  | 219453 |
|  |  | 10060 |
|  |  | 9789 |
|  |  | 94241 |
|  |  | 4724 |
|  |  | 11010 |
|  |  | 154661 |
|  |  | 5641 |
|  |  | 79768 |
|  |  | 143 |
|  |  | 8638 |
|  |  | 60526 |
|  |  | 3017 |
|  |  | 127933 |
|  |  | 11170 |
|  |  | 23164 |
|  |  | 3655 |
|  |  | 3939 |
|  |  | 51 |
|  |  | 3727 |
|  |  | 9958 |
|  |  | 51504 |
|  |  | 284422 |
|  |  | 25879 |
|  |  | 80023 |
|  |  | 3021 |
|  |  | 5908 |
|  |  | 1642 |
|  |  | 100529209 |
|  |  | 3954 |
|  |  | 25813 |
|  |  | 11031 |
|  |  | 81027 |
|  |  | 51251 |
|  |  | 339834 |
|  |  | 6834 |
|  |  | 128506 |
|  |  | 51768 |
|  |  | 50808 |
|  |  | 165918 |
|  |  | 372 |
|  |  | 9107 |
|  |  | 85452 |
|  |  | 94081 |
|  |  | 1317 |
|  |  | 80308 |
|  |  | 55622 |
|  |  | 64841 |
|  |  | 51131 |
|  |  | 713 |
|  |  | 396 |
|  |  | 4713 |
|  |  | 51102 |
|  |  | 3752 |
|  |  | 5689 |
|  |  | 3732 |
|  |  | 1471 |
|  |  | 55840 |
|  |  | 254251 |
|  |  | 54187 |
|  |  | 1725 |
|  |  | 84313 |
|  |  | 1072 |
|  |  | 4725 |
|  |  | 55034 |
|  |  | 6138 |
|  |  | 93974 |
|  |  | 79001 |
|  |  | 23645 |
|  |  | 8536 |
|  |  | 51172 |
|  |  | 90338 |
|  |  | 10166 |
|  |  | 6176 |
|  |  | 7381 |
|  |  | 4922 |
|  |  | 79899 |
|  |  | 23594 |
|  |  | 55630 |
|  |  | 2637 |
|  |  | 10289 |
|  |  | 126374 |
|  |  | 56674 |
|  |  | 5897 |
|  |  | 8664 |
|  |  | 51377 |
|  |  | 26122 |
|  |  | 2027 |
|  |  | 5431 |
|  |  | 79036 |
|  |  | 27173 |
|  |  | 222236 |
| GSE104121M GSE6874M | 158 | 55226 |
|  |  | 80727 |
|  |  | 339324 |
|  |  | 4200 |
|  |  | 6257 |
|  |  | 83541 |
|  |  | 6510 |
|  |  | 7189 |
|  |  | 2936 |
|  |  | 51100 |
|  |  | 973 |
|  |  | 90268 |
|  |  | 11261 |
|  |  | 4798 |
|  |  | 9972 |
|  |  | 56006 |
|  |  | 338442 |
|  |  | 79058 |
|  |  | 115024 |
|  |  | 375790 |
|  |  | 83590 |
|  |  | 139818 |
|  |  | 79598 |
|  |  | 54778 |
|  |  | 6747 |
|  |  | 1509 |
|  |  | 25842 |
|  |  | 160518 |
|  |  | 4790 |
|  |  | 55005 |
|  |  | 7919 |
|  |  | 972 |
|  |  | 29880 |
|  |  | 11034 |
|  |  | 64132 |
|  |  | 51399 |
|  |  | 23383 |
|  |  | 5533 |
|  |  | 10621 |
|  |  | 8861 |
|  |  | 389541 |
|  |  | 1397 |
|  |  | 8673 |
|  |  | 7071 |
|  |  | 26167 |
|  |  | 5899 |
|  |  | 6251 |
|  |  | 1756 |
|  |  | 23367 |
|  |  | 6811 |
|  |  | 2904 |
|  |  | 27243 |
|  |  | 6228 |
|  |  | 284361 |
|  |  | 2957 |
|  |  | 8161 |
|  |  | 3654 |
|  |  | 23309 |
|  |  | 6314 |
|  |  | 4193 |
|  |  | 4318 |
|  |  | 25874 |
|  |  | 1523 |
|  |  | 7763 |
|  |  | 622 |
|  |  | 64108 |
|  |  | 4528 |
|  |  | 5411 |
|  |  | 23495 |
|  |  | 1192 |
|  |  | 4616 |
|  |  | 30834 |
|  |  | 55700 |
|  |  | 3836 |
|  |  | 9908 |
|  |  | 1396 |
|  |  | 2907 |
|  |  | 23097 |
|  |  | 12 |
|  |  | 65991 |
|  |  | 6431 |
|  |  | 7314 |
|  |  | 1161 |
|  |  | 1380 |
|  |  | 7329 |
|  |  | 126364 |
|  |  | 51237 |
|  |  | 221037 |
|  |  | 974 |
|  |  | 29802 |
|  |  | 1647 |
|  |  | 54520 |
|  |  | 26994 |
|  |  | 65987 |
|  |  | 10077 |
|  |  | 10419 |
|  |  | 7266 |
|  |  | 11277 |
|  |  | 2113 |
|  |  | 84304 |
|  |  | 23258 |
|  |  | 8698 |
|  |  | 8439 |
|  |  | 7390 |
|  |  | 1781 |
|  |  | 143686 |
|  |  | 5437 |
|  |  | 84896 |
|  |  | 6248 |
|  |  | 302 |
|  |  | 83759 |
|  |  | 7343 |
|  |  | 55568 |
|  |  | 53918 |
|  |  | 23589 |
|  |  | 220972 |
|  |  | 256949 |
|  |  | 29916 |
|  |  | 1778 |
|  |  | 7706 |
|  |  | 50486 |
|  |  | 51762 |
|  |  | 65061 |
|  |  | 84309 |
|  |  | 3958 |
|  |  | 1327 |
|  |  | 64421 |
|  |  | 11341 |
|  |  | 57805 |
|  |  | 10577 |
|  |  | 55904 |
|  |  | 54780 |
|  |  | 79922 |
|  |  | 284207 |
|  |  | 94059 |
|  |  | 4170 |
|  |  | 5601 |
|  |  | 54881 |
|  |  | 10286 |
|  |  | 9448 |
|  |  | 90480 |
|  |  | 6277 |
|  |  | 718 |
|  |  | 55278 |
|  |  | 79760 |
|  |  | 23759 |
|  |  | 6932 |
|  |  | 5704 |
|  |  | 54984 |
|  |  | 51433 |
|  |  | 1603 |
|  |  | 54498 |
|  |  | 27166 |
|  |  | 1912 |
|  |  | 57142 |
|  |  | 4615 |
|  |  | 9135 |
|  |  | 29957 |

Supplementary Data 4

| GO ID | term description | observed gene count | background gene count | strength |
| --- | --- | --- | --- | --- |
| GO:0009987 | cellular process | 494 | 14652 | 0.06 |
| GO:1901564 | organonitrogen compound metabolic process | 220 | 5281 | 0.15 |
| GO:0048518 | positive regulation of biological process | 222 | 5459 | 0.14 |
| GO:0016043 | cellular component organization | 210 | 5163 | 0.14 |
| GO:0008152 | metabolic process | 344 | 9569 | 0.09 |
| GO:0044238 | primary metabolic process | 321 | 8808 | 0.09 |
| GO:0071704 | organic substance metabolic process | 331 | 9135 | 0.09 |
| GO:0048522 | positive regulation of cellular process | 199 | 4898 | 0.14 |
| GO:0050896 | response to stimulus | 289 | 7824 | 0.1 |
| GO:0071840 | cellular component organization or biogenesis | 212 | 5342 | 0.13 |
| GO:0051240 | positive regulation of multicellular organismal process | 80 | 1551 | 0.24 |
| GO:0044237 | cellular metabolic process | 316 | 8797 | 0.09 |
| GO:0044281 | small molecule metabolic process | 88 | 1779 | 0.23 |
| GO:0006950 | response to stress | 140 | 3267 | 0.16 |
| GO:0043062 | extracellular structure organization | 27 | 339 | 0.43 |
| GO:0044267 | cellular protein metabolic process | 149 | 3603 | 0.15 |
| GO:0051094 | positive regulation of developmental process | 67 | 1286 | 0.25 |
| GO:0006793 | phosphorus metabolic process | 96 | 2086 | 0.19 |
| GO:0016192 | vesicle-mediated transport | 82 | 1699 | 0.22 |
| GO:0019538 | protein metabolic process | 168 | 4194 | 0.13 |
| GO:0051128 | regulation of cellular component organization | 104 | 2306 | 0.19 |
| GO:0065008 | regulation of biological quality | 147 | 3559 | 0.15 |
| GO:0045597 | positive regulation of cell differentiation | 51 | 908 | 0.28 |
| GO:0002376 | immune system process | 105 | 2370 | 0.18 |
| GO:0032984 | protein-containing complex disassembly | 20 | 220 | 0.49 |
| GO:0043624 | cellular protein complex disassembly | 15 | 131 | 0.59 |
| GO:0051179 | localization | 200 | 5233 | 0.11 |
| GO:0007010 | cytoskeleton organization | 52 | 953 | 0.27 |
| GO:0009611 | response to wounding | 35 | 547 | 0.34 |
| GO:0006796 | phosphate-containing compound metabolic process | 93 | 2065 | 0.19 |
| GO:0042060 | wound healing | 31 | 461 | 0.36 |
| GO:0002521 | leukocyte differentiation | 24 | 313 | 0.42 |
| GO:0006807 | nitrogen compound metabolic process | 293 | 8349 | 0.08 |
| GO:0048513 | animal organ development | 122 | 2926 | 0.15 |
| GO:0032940 | secretion by cell | 51 | 959 | 0.26 |
| GO:0071702 | organic substance transport | 90 | 2040 | 0.18 |
| GO:1901135 | carbohydrate derivative metabolic process | 55 | 1083 | 0.24 |
| GO:0043085 | positive regulation of catalytic activity | 66 | 1381 | 0.21 |
| GO:0022603 | regulation of anatomical structure morphogenesis | 50 | 961 | 0.25 |
| GO:0051716 | cellular response to stimulus | 225 | 6212 | 0.09 |
| GO:0022411 | cellular component disassembly | 25 | 364 | 0.37 |
| GO:0050793 | regulation of developmental process | 102 | 2416 | 0.16 |
| GO:0048584 | positive regulation of response to stimulus | 89 | 2054 | 0.17 |
| GO:0051347 | positive regulation of transferase activity | 36 | 630 | 0.29 |
| GO:0070527 | platelet aggregation | 6 | 25 | 0.91 |
| GO:0002576 | platelet degranulation | 13 | 129 | 0.54 |
| GO:0031344 | regulation of cell projection organization | 35 | 608 | 0.29 |
| GO:0007596 | blood coagulation | 21 | 288 | 0.39 |
| GO:0010720 | positive regulation of cell development | 30 | 491 | 0.32 |
| GO:0046903 | secretion | 53 | 1070 | 0.23 |
| GO:1901566 | organonitrogen compound biosynthetic process | 64 | 1370 | 0.2 |
| GO:0001775 | cell activation | 51 | 1024 | 0.23 |
| GO:0016310 | phosphorylation | 59 | 1236 | 0.21 |
| GO:0034109 | homotypic cell-cell adhesion | 7 | 38 | 0.8 |
| GO:0006810 | transport | 157 | 4130 | 0.11 |
| GO:0006898 | receptor-mediated endocytosis | 17 | 209 | 0.44 |
| GO:0010646 | regulation of cell communication | 131 | 3327 | 0.13 |
| GO:0043933 | protein-containing complex subunit organization | 78 | 1770 | 0.18 |
| GO:0044093 | positive regulation of molecular function | 76 | 1713 | 0.18 |
| GO:0048583 | regulation of response to stimulus | 149 | 3882 | 0.12 |
| GO:0051234 | establishment of localization | 161 | 4248 | 0.11 |
| GO:0051239 | regulation of multicellular organismal process | 113 | 2788 | 0.14 |
| GO:0006996 | organelle organization | 124 | 3131 | 0.13 |
| GO:0030198 | extracellular matrix organization | 21 | 296 | 0.38 |
| GO:0032270 | positive regulation of cellular protein metabolic process | 68 | 1496 | 0.19 |
| GO:0048731 | system development | 157 | 4144 | 0.11 |
| GO:0051641 | cellular localization | 92 | 2180 | 0.16 |
| GO:0120035 | regulation of plasma membrane bounded cell projection organization | 34 | 600 | 0.29 |
| GO:0019752 | carboxylic acid metabolic process | 44 | 854 | 0.24 |
| GO:0051247 | positive regulation of protein metabolic process | 71 | 1587 | 0.18 |
| GO:0033574 | response to testosterone | 7 | 41 | 0.76 |
| GO:0023051 | regulation of signaling | 131 | 3360 | 0.12 |
| GO:0030168 | platelet activation | 12 | 120 | 0.53 |
| GO:0097006 | regulation of plasma lipoprotein particle levels | 9 | 70 | 0.64 |
| GO:2000727 | positive regulation of cardiac muscle cell differentiation | 5 | 18 | 0.98 |
| GO:0001934 | positive regulation of protein phosphorylation | 47 | 941 | 0.23 |
| GO:0006897 | endocytosis | 30 | 510 | 0.3 |
| GO:0008104 | protein localization | 84 | 1966 | 0.16 |
| GO:0022402 | cell cycle process | 45 | 890 | 0.24 |
| GO:0030036 | actin cytoskeleton organization | 26 | 418 | 0.33 |
| GO:0032501 | multicellular organismal process | 230 | 6507 | 0.08 |
| GO:0045937 | positive regulation of phosphate metabolic process | 51 | 1052 | 0.22 |
| GO:0033036 | macromolecule localization | 94 | 2268 | 0.15 |
| GO:0071705 | nitrogen compound transport | 74 | 1690 | 0.17 |
| GO:0080134 | regulation of response to stress | 60 | 1299 | 0.2 |
| GO:0097062 | dendritic spine maintenance | 4 | 10 | 1.13 |
| GO:0030029 | actin filament-based process | 29 | 493 | 0.3 |
| GO:0033554 | cellular response to stress | 69 | 1553 | 0.18 |
| GO:0010543 | regulation of platelet activation | 6 | 31 | 0.82 |
| GO:0007162 | negative regulation of cell adhesion | 18 | 245 | 0.4 |
| GO:0002682 | regulation of immune system process | 63 | 1391 | 0.19 |
| GO:0042327 | positive regulation of phosphorylation | 48 | 984 | 0.22 |
| GO:0030098 | lymphocyte differentiation | 17 | 226 | 0.41 |
| GO:0002684 | positive regulation of immune system process | 44 | 882 | 0.23 |
| GO:0007154 | cell communication | 189 | 5219 | 0.09 |
| GO:0009966 | regulation of signal transduction | 119 | 3033 | 0.13 |
| GO:0040012 | regulation of locomotion | 44 | 881 | 0.23 |
| GO:0070887 | cellular response to chemical stimulus | 107 | 2672 | 0.13 |
| GO:1902531 | regulation of intracellular signal transduction | 76 | 1764 | 0.17 |
| GO:0009888 | tissue development | 71 | 1626 | 0.17 |
| GO:0000278 | mitotic cell cycle | 34 | 628 | 0.27 |
| GO:0051130 | positive regulation of cellular component organization | 53 | 1128 | 0.2 |
| GO:0043603 | cellular amide metabolic process | 38 | 732 | 0.25 |
| GO:0050865 | regulation of cell activation | 29 | 506 | 0.29 |
| GO:0050878 | regulation of body fluid levels | 28 | 483 | 0.3 |
| GO:0031346 | positive regulation of cell projection organization | 22 | 343 | 0.34 |
| GO:0007339 | binding of sperm to zona pellucida | 6 | 34 | 0.78 |
| GO:0010033 | response to organic substance | 111 | 2815 | 0.13 |
| GO:0098657 | import into cell | 33 | 609 | 0.27 |
| GO:0006464 | cellular protein modification process | 117 | 2999 | 0.12 |
| GO:0035556 | intracellular signal transduction | 67 | 1528 | 0.17 |
| GO:0043408 | regulation of MAPK cascade | 37 | 712 | 0.25 |
| GO:0055086 | nucleobase-containing small molecule metabolic process | 35 | 662 | 0.26 |
| GO:0060252 | positive regulation of glial cell proliferation | 4 | 12 | 1.05 |
| GO:0098609 | cell-cell adhesion | 25 | 416 | 0.31 |
| GO:0002790 | peptide secretion | 13 | 153 | 0.46 |
| GO:0007166 | cell surface receptor signaling pathway | 90 | 2198 | 0.14 |
| GO:0009306 | protein secretion | 12 | 134 | 0.48 |
| GO:0022604 | regulation of cell morphogenesis | 26 | 442 | 0.3 |
| GO:0032879 | regulation of localization | 101 | 2524 | 0.13 |
| GO:1903047 | mitotic cell cycle process | 31 | 564 | 0.27 |
| GO:2000026 | regulation of multicellular organismal development | 79 | 1876 | 0.16 |
| GO:0048738 | cardiac muscle tissue development | 13 | 154 | 0.46 |
| GO:1901565 | organonitrogen compound catabolic process | 46 | 958 | 0.21 |
| GO:0006082 | organic acid metabolic process | 46 | 959 | 0.21 |
| GO:0140014 | mitotic nuclear division | 12 | 136 | 0.48 |
| GO:1902533 | positive regulation of intracellular signal transduction | 46 | 959 | 0.21 |

Supplementary Data 5

| Query | 10Gy24Blk34k | 10Gy24Blk34k | 10Gy6hrGSE6874 | 10Gy6Blk34k | 10Gy6Balb | 10Gy636k | 2Gy2434k | 2Gy2436k | 2Gy66874 | 2Gy6BLK34k | 2Gy6Balbc34k | 0.5Gy7Dblk36k | 0.5Gy24blk34k | 0.5Gy66874 | 0.5Gy6blk34k | 0.5Gy6balbc34k | 0.5Gy636k | Human6874 | Human 10640 | 2Gy7Dblk36k | Mo24hr2Gy | Mo24hr4Gy | Mo24hr8Gy | Mo48hr2Gy | Mo48hr4Gy | Mo48hr8Gy | Mo6hr4Gy | Mo16hr1Gy | Mo16hr2Gy | Mo16hr4Gy | Mo16hr8Gy | Mo16hr12Gy |
| --- | --- | --- | --- | --- | --- | --- | --- | --- | --- | --- | --- | --- | --- | --- | --- | --- | --- | --- | --- | --- | --- | --- | --- | --- | --- | --- | --- | --- | --- | --- | --- | --- |
| IL1B | -1.982573473 | -0.9825734726 | 0.0174265274 | 1.017426527 | -0.5153695211 | 1.121622768 | -1.87950169 | 0.403812195 | 1.834175629 | 2.665721148 | 0.3037111357 | 2.016430148 | -1.623704227 | 0.2727594343 | 2.942196447 | 0.2796273742 | 1.010905501 | -0.3313019958 | -0.5421258275 | 1.844232691 | 3.690403958 | 3.396469331 | 2.816656546 | 3.364731547 | 3.165262104 | 3.805225094 | -1.927284173 | -1.869860702 | -1.76951993 | -1.858232561 | -1.857611355 | -1.873466453 |
| APOE | -1.47922923 | -0.4792292301 | 0.5207707699 | 1.52077077 | 0.0257627828 | 1.186756715 | -1.904106188 | 0.4368822342 | 0.2063842994 | 1.411088116 | -0.1704010725 | -5.189957142 | -1.665033431 | -0.0131606981 | 2.469837219 | -0.1776205839 | 1.107669854 | 0.1132401819 | 0.406264192 | 2.047443524 | 3.293090217 | 3.302081251 | 3.978801066 | 4.705812471 | 3.935496786 | 2.86863573 | -3.516346038 | -3.443962327 | -3.50302374 | -3.518482632 | -3.403096736 | -3.520467936 |
| F10 | -1.168045867 | 1.475559354 | 1.939731438 | 4.307239982 | 0.0064601783 | 0.4694237458 | -1.23924836 | 0.5397248502 | 1.683320565 | 1.414333535 | 0.6967596124 | -3.581910563 | -1.048363795 | 0.6672241749 | 1.571689693 | 0.6699013484 | 0.5724094876 | 0.1119847154 | 0.1665572168 | 0.5615716667 | 4.502460813 | 4.632706236 | 5.173802255 | 5.164838341 | 5.737230262 | 5.502895017 | -2.098778739 | -1.210145601 | -1.63063456 | -1.65335973 | -1.420825527 | -1.588408072 |
| CSF3R | -1.699789217 | -0.6997892172 | 0.3002107828 | 1.300210783 | -0.0342510209 | 2.390226003 | -1.225371274 | 0.0451254426 | 2.082464632 | 1.94363555 | -0.1286472163 | -4.151902782 | -0.6318337876 | 0.3810392038 | 1.096291846 | 0.0713831742 | 2.080614896 | -0.08828331205 | -0.0548983908 | 0.8788795128 | 3.328122842 | 3.466009907 | 3.44906507 | 2.804354925 | 2.443284874 | 2.520874168 | -3.424395604 | -3.390885514 | -2.98142626 | -3.51763318 | -3.232500876 | -3.181356633 |
| HDC | -1.678907272 | -0.6789072719 | 0.3210927281 | 1.321092728 | 0.2418585714 | 1.72094279 | -1.46406422 | -0.0671533618 | 2.228594667 | 2.60233757 | 0.0505354796 | -1.827822908 | -1.281504984 | 0.3187685653 | 2.314741324 | 0.0244378423 | 0.9898233817 | 0.01847853903 | -0.0454598589 | 1.648646046 | 4.679508566 | 4.643952102 | 4.911482884 | 3.237140929 | 4.114129192 | 4.011341765 | -1.494463224 | -1.207476526 | -1.389549806 | -1.580772011 | -0.9955264391 | -1.040288663 |
| SYTL4 | -0.7710901718 | 1.119617224 | 2.054686901 | 3.002483246 | -0.0300965785 | 0.3325252822 | -0.7895214829 | -0.0200715278 | 1.308460096 | 1.788200808 | 0.1924373647 | -2.636928859 | -1.107921882 | 0.8077060902 | 2.263266092 | 0.3518272156 | 0.264702447 | 0.4695164615 | 0.3091289467 | -1.315267055 | 4.474098437 | 4.420755589 | 3.681030408 | 4.726476358 | 4.70742175 | 5.710387222 | -2.831736223 | -2.803976634 | -2.771781802 | -2.80224127 | -3.00360888 | -2.499216234 |
| APOC2 | 2.356685076 | 2.114303411 |  |  | -0.0580816526 | 1.521257141 | 1.823580423 | 0.6140170486 | 1.309827407 | 1.760643138 | 0.0457062402 | -1.772786278 | 0.1673963259 | 0.629873095 | 0.8677975987 | 0.2372298569 | 1.381346298 | 0.07775925081 | 0.1483606871 | 2.150664416 | -1.633847964 | -1.72774309 | -1.051303311 | -1.141668507 | -1.857726494 | -3.053918101 | 2.119988624 | 2.331129309 | 2.298907468 | 2.299092513 | 2.161289439 | 1.974069725 |
| VWF | 2.853153985 | 3.853153985 | 4.853153985 | 5.853153985 | 0.0561723747 | 0.6322031716 | 2.020818085 | 0.6593317538 | 1.888917926 | 0.8830148548 | 0.1562541008 | 1.001334359 | 0.6581151994 | 1.397052855 | 0.2648007788 | 0.157525884 | 0.5434003501 | -0.05101233291 | 0.0420498047 | 0.7044930424 | 5.065011139 | 5.128410128 | 4.537911764 | 4.830900228 | 5.224552267 | 6.06095517 | -3.685490199 | -2.87491873 | -3.530290664 | -3.547050544 | -2.96026745 | -2.816791536 |
| HCK | -1.297943511 | -0.2979435112 | 0.7020564888 | 1.702056489 | 0.0007394032 | 1.070716491 | -1.170523339 | 0.1380973382 | 1.131064325 | 1.826044884 | -0.3189354364 | 0.7323069739 | -1.445311922 | 0.0272257679 | 1.888696523 | -0.0263979816 | 0.6913807684 | 0.2121343616 | 0.0342953804 | 0.471673672 | 2.481821575 | 1.904962338 | 1.956148732 | 1.191290942 | 2.142914796 | 1.508916214 | -1.72777585 | -1.306509697 | -2.223503559 | -1.88774406 | -2.135992512 | -1.771254865 |
| SMOX | -0.4321184698 | 0.5678815302 | 1.56788153 | 2.56788153 | -0.0207507447 | -2.45892691 | -0.2203121099 | 0.1651333241 | 1.589244263 | 1.360039732 | 0.2522219544 | -0.7721692517 | -0.6138238196 | 0.3713399893 | 1.036011587 | 0.3283971231 | -1.023945639 |  |  | 1.569887303 | 3.939302852 | 4.100341821 | 3.903104113 | 3.155591624 | 2.557069716 | 3.8856084 | -3.929644327 | -3.802532201 | -3.696320728 | -3.665068303 | -3.753787972 | -3.536619871 |
| PRKCI | 0.9084009083 | 1.908400908 | 2.908400908 | 3.908400908 | 0.0123581229 | 0.0706680525 | 1.071064569 | -0.5898624276 | -0.1696574838 | -1.022786819 | 0.2003513832 | 2.157154029 | 1.1548523 | 0.2194266718 | -1.406161446 | 0.2006970119 | -0.1249744261 | 0.1710494619 | 0.10899092 | -0.2679213654 | -1.879993346 | -2.147106122 | -2.34787911 | -1.945854401 | -1.713795909 | -1.918785316 | 2.270944849 | 1.638971436 | 1.53389133 | 1.526526678 | 2.127708996 | 1.471080053 |
| FECH | -1.405488038 | -0.4054880379 | 0.5945119621 | 1.594511962 | 0.3165060697 | -1.033742508 | -1.610181462 | -3.487184315 | -0.82369808 | 0.8391792595 | 0.9113692301 | -4.931483725 | -1.369522834 | -0.9784797594 | 1.249808531 | 0.2520017739 | -0.6771851526 | -0.07954141846 | -0.3171641563 |  | 4.36889859 | 4.456899696 | 4.463640382 | 3.463641008 | 1.630063345 | 2.23762977 | -5.209320362 | -4.666482008 | -4.837674076 | -4.963012345 | -5.131437557 | -5.170776426 |
| NFKBIA | -0.6443854861 | 0.3556145139 | 1.355614514 | 2.355614514 | -0.0150420344 | 1.839802168 | -0.8016980912 | -0.6767901888 | 1.874188961 | 2.046130608 | -0.0061659028 | -0.5299804051 | -0.9159542773 | 0.0889478614 | 1.489169743 | -0.0937711681 | 1.494163616 | -0.4402425849 | -0.6245060535 | 1.092624219 | 2.943255701 | 2.253504331 | 2.43971877 | 2.600800514 | 2.803777277 | 1.853592126 | -3.695366413 | -3.481170495 | -3.581358611 | -3.428331555 | -2.920071325 | -3.58726909 |
| ALAS2 | -1.743043132 | 1.575481812 |  |  | 0.100507966 | -0.1913465812 | -1.869376644 | -1.286288005 | 0.095358479 | 1.671787305 | -0.177935595 | -2.614964221 | -1.741314085 | -0.2948540469 | 2.601352665 | 0.0891672939 | 0.027032046 | 0.08491302653 | 0.0225238473 |  | 6.184595816 | 6.097393957 | 6.112199409 | 5.927634986 | 4.307530663 | 5.412907824 | -6.272516223 | -6.580760844 | -6.525541721 | -6.126148117 | -6.526570438 | -6.267228718 |
| IFNAR2 | -1.329741277 | 1.38068958 |  |  | 0.007268786 | -0.3332283757 | -1.523918622 | -3.162360376 | -0.0907998481 | 1.467832474 | 0.1451515111 | -2.073365065 | -1.63365901 | -0.6437201055 | 1.946741938 | -0.064755972 | -0.1748101138 | -0.1156632556 | -0.3387108549 | 1.561873432 | 2.644622912 | 2.551229812 | 2.801280167 | 2.699464887 | 3.096177749 | 2.456060344 | 0.440525274 | -2.416415114 | -2.20093556 | -2.139968823 | -1.361868146 | -2.30608326 |
| TBXAS1 | -1.140946058 |  |  |  | 0.2789435912 | -0.5326206966 | -1.326832428 | -0.2473396739 | 1.609444106 | 2.062191789 | 0.221612435 | -2.663694851 | -1.175269209 | 0.745642222 | 1.936470859 | 0.1346204122 | 0.0027055933 | -0.1340030922 | -0.2008664156 | 0.4083724249 | 4.423330977 | 4.602482732 | 4.515952969 | 4.631015888 | 4.980451255 | 5.456906493 | -2.567320477 | -2.716063004 | -2.654201232 | -2.824291781 | -2.582766608 | -2.562866813 |
| RAMP1 | -1.050407305 |  |  |  | -0.009900452 | -0.1722572915 | -1.038468135 | 0.4654593007 | 1.129780054 | 1.56446289 | 0.0338961775 | -1.226411457 | -1.516328791 | 0.9373843451 | 2.040323286 | -0.3740235806 | 0.0433881987 | -0.04108000314 | -0.1410145908 |  | 3.642268218 | 3.785368703 | 2.865169276 | 3.277672578 | 2.989466043 | 4.299718515 | -2.137132177 | -1.66184335 | -1.976671465 | -1.943676628 | -2.234735996 | -2.029345511 |
| GP9 | -1.580232855 |  |  |  | -0.0078517104 | -0.1763959866 | -1.958330988 | 0.4352491353 | 1.992049807 | 2.648673137 | -0.2848835462 | -0.2438147839 | -1.813169735 | 1.312425895 | 3.6618277 | -0.1039165649 | 0.3386377901 | 0.02634635355 | -0.0645275949 |  | 6.049391796 | 5.841212887 | 4.895410182 | 6.601317853 | 5.934527581 | 7.163370257 | -3.476870699 | -4.29544232 | -3.814409321 | -4.04522548 | -4.181664097 | -4.494326652 |
| C1QB | -1.806130341 | -0.8061303406 | 0.1938696594 | 1.193869659 | -0.006097754 | 2.298357468 | 1.391061096 | 0.4475738098 | 0.5006859463 | -1.303296964 | 0.0641598123 | -0.5212846776 | 1.307059208 | -0.057033949 | -2.411939138 | 0.0035882301 | -0.1550056894 | -0.2094445522 | -0.1621729766 | 0.0433998925 | 2.60057464 | 3.244878105 | 4.884201542 | 3.495852769 | 4.77968888 | 2.557930868 | -1.529055479 | -1.665270729 | -1.751001453 | -1.36273329 | -1.37429604 | -1.716894286 |
| SELL | -2.701300643 | -1.701300643 | -0.7013006433 | 0.2986993567 | -0.1116252254 | -0.1239028931 | -1.579838626 | -0.4188969842 | -0.0252605756 | 1.945842031 | -0.342071114 | 1.04270444 | -1.720837588 | 0.0413252082 | 3.109197829 | 0.9626414129 | -0.0065532126 | -0.2347704621 | -0.3080450436 |  | 3.346792598 | 3.041074569 | 2.987008739 | 2.826105687 | 2.704700908 | 3.146209182 | -3.954247328 | -3.582088451 | -3.631258702 | -2.96958777 | -3.480659101 | -3.653540985 |
| CSTB | 1.026317817 | 2.026317817 | 3.026317817 | 4.026317817 | -0.0071254885 | 0.3947625395 | 1.109469159 | -0.2090443071 | 0.7814384836 | -0.3879727814 | -0.0664159008 | -0.1041650425 | 1.160242993 | 0.1501668242 | -1.40784101 | 0.1056126729 | -0.1341753992 | 0.1853096698 | 0.1107822728 | 0.1565353729 | 2.033187662 | 1.657622741 | 2.408297485 | 3.033236512 | 3.12793974 | 2.472511469 | -1.621231183 | -2.20262357 | -2.14167733 | -2.337818805 | -1.779737811 | -2.468199747 |
| LRG1 | -0.1856867109 |  |  |  | -0.0318129756 | 4.162189617 | -0.2851718269 | 1.05545884 | 3.738944018 | 3.433099041 | 0.3101386517 | -1.290096684 | -0.9095645117 | -0.0841804055 | 1.194848068 | 0.2573434363 | 2.124154597 |  |  | 1.640916702 | 4.935285398 | 5.274935157 | 6.125481118 | 3.535142767 | 4.732201839 | 4.676018832 | -1.220957094 | -1.396187321 | -1.251543876 | -0.874321524 | -1.448886344 | -1.374128581 |
| CTSH | -0.7874386411 | 0.2125613589 | 1.212561359 | 2.212561359 | 0.007564474 | -0.1728209786 | -1.196336829 | -0.6591309551 | -0.4744814855 | 1.058041899 | -0.3900090087 | -3.931788772 | -1.728609293 | -0.2038588463 | 2.244601096 | 0.2284720797 | 0.7260971001 | 0.08523416478 | -0.030493936 | 0.2475956201 | 2.333328012 | 1.881123188 | 2.388204135 | 2.704742999 | 3.011220172 | 1.403163978 | -3.060546556 | -3.451800262 | -3.085493254 | -3.237110849 | -3.361811727 | -3.050013106 |
| P2RY12 | -0.4464493879 |  | -0.1761118567 |  | 0.555830684 | 0.7266507536 | -1.087226657 | 0.6386370033 | 1.631438722 | 1.563742284 | 0.1025635895 | -4.681815835 | -1.035689641 | 1.129536539 | 2.304053955 | -0.0454939553 | 0.677007232 | -0.02237994368 | -0.1469377253 | 0.7836540322 | 4.280236603 | 4.326757503 | 3.002944721 | 4.505748016 | 4.114579949 | 5.469170248 | -2.360954898 | -2.05247554 | -2.13803412 | -2.36587327 | -1.908193164 | -2.202497716 |
| DSTN | 2.033130984 | 3.033130984 | 4.033130984 | 5.033130984 | 0.0164128558 | 0.8492412866 | 0.8574569092 | 0.2543566368 | 1.69865821 | 1.982037912 | 0.3572320388 | -0.2290548377 | -0.5203768731 | 0.6555262877 | 1.552341507 | 0.2264525047 | 0.0382651578 | -0.0326142565 | 0.209008713 |  | 2.845332424 | 2.727202289 | 2.87377117 | 3.215868914 | 3.112569381 | 3.643470885 | -1.581733704 | -1.206432165 | -1.575606471 | -1.459870296 | -1.725894468 | -1.595665971 |
| CKS1B | 1.163275543 | 2.163275543 | 3.163275543 | 4.163275543 | -0.0080702834 | 0.179262919 | 1.220271191 | -0.4082063921 | 0.53216581 | -0.6752271808 | 0.405745359 | 0.4465440782 | 1.154781379 | 0.3343808339 | -1.639723792 | -0.0064210622 | -0.1760310197 | 0.1500891999 | 0.2802424285 |  | 1.514957282 | 1.278448838 | 1.666442904 | 2.107136252 | 1.436726389 | 1.714685967 | -1.082938603 | -1.57043879 | -1.216487657 | -1.297947684 | -1.529396793 | -1.668690776 |
| TF | -0.6921571161 | 0.3078428839 | 1.307842884 | 2.307842884 | 0.0175887978 | 1.861621866 | -0.5568941405 | -0.0955889197 | 1.448683179 | 1.377502332 | -0.2417957443 | -0.7682310388 | -0.4413210029 | 0.1774633577 | 1.058782538 | 0.0720493708 | 1.681073087 | 0.3170077289 | 0.3883000298 | 1.440913147 | 2.307472507 | 2.707251219 | 3.610250039 | 4.387873582 | 4.197148056 | 2.702262756 | -1.429230343 | -1.352644426 | -1.484990284 | -1.581882196 | -1.495750677 | -0.9537857443 |
| ALOX12 | 2.833391902 |  |  |  | -0.0162144099 | -0.6646194227 | 2.127214412 | -0.356041099 | 1.957401967 | 1.287760245 | 0.3253982945 | -2.927803527 | 0.5743320896 | 1.307974556 | 0.1391413204 | 0.0896951886 | -0.2997390206 | 0.4856529353 | 0.8131797699 | 1.258291864 | 3.228274344 | 3.550577813 | 2.807193254 | 2.956254946 | 2.638872166 | 3.763547422 | -1.514652267 | -1.432296297 | -1.674233453 | -1.832977339 | -1.292161177 | -1.531240115 |
| ALOX5 | 0.8059617436 |  |  |  | 0.1749561711 | 3.716686724 | 1.001029554 | 0.9846515392 | 1.856832139 | 1.156982438 | 0.0895760681 | -1.382623115 | 0.2599635485 | 0.107729516 | -0.4007916423 | 0.096838957 | 2.455676579 | -0.360152838 | -0.6606009381 |  | 3.239423347 | 3.026220882 | 3.965209292 | 2.390700244 | 3.906455404 | 3.347572352 | -1.514844231 | -1.346823789 | -1.267316967 | -1.221068447 | -1.346034505 | -1.206388443 |
| PFKFB4 | -0.0489595651 | 0.9818553973 | 2.58002018 | 3.825724681 | 0.2400909207 | 1.28911341 | 0.0607440312 | 0.3108434232 | 1.442558718 | 1.000580277 | 0.4142871325 | -1.132434272 | 0.2336396118 | 0.3232933335 | 0.7321239697 | 0.057913476 | 0.588757581 | -0.2707157585 | -0.1555742495 |  | 4.083650928 | 4.003315108 | 3.476973588 | 3.94091451 | 3.747825764 | 4.836463077 | -2.128114195 | -2.084612711 | -2.005940736 | -1.896867132 | -2.223385652 | -1.661491235 |
| TSPO | 0.2446994471 | 1.244699447 | 2.244699447 | 3.244699447 | -0.0043734149 | 0.2444088746 | 0.3823418013 | -0.0353081596 | 1.740651349 | 1.111381618 | 0.2811377577 | -1.723075521 | 0.1499091838 | 0.6019816826 | 0.2812472447 | -0.1358469844 | -0.5742335339 |  |  |  | 5.199079884 | 5.472243076 | 5.087423604 | 5.4718646 | 5.447541614 | 6.179390169 | -3.64263923 | -3.942320345 | -3.906752147 | -4.068389037 | -3.901841156 | -3.894686272 |
| APOBR | 0.1495711714 | 1.149571171 | 2.149571171 | 3.149571171 | -0.1275582868 | 3.061921931 | -0.2795281643 | 1.287262337 | 1.618622874 | 1.163691614 | -0.1306594361 | -0.0066202297 | -0.2331127803 | -0.1175667522 | 0.1420027385 | 0.0425482733 | 1.556902667 |  |  |  | 3.15437788 | 2.983879977 | 3.220010886 | 1.919610899 | 2.901659698 | 3.093952977 | -1.285255419 | -0.8675733632 | -1.260425317 | -1.086657457 | -1.081104999 | -0.9637376006 |
| CLU | -1.005346138 | -0.0053461382 | 0.9946538618 | 1.994653862 | 0.0947078931 | 0.1912616297 | -0.8376439982 | 0.1169484601 | 2.306938654 | 2.099834258 | 0.5519038929 | -0.7441778501 | -0.671268019 | 1.581703932 | 2.284946468 | 0.1153528645 | 0.2423116115 | 0.2718666074 | 0.2343027228 | -0.2257718528 | 5.794769837 | 5.826971443 | 5.12224264 | 6.119741446 | 5.521158174 | 6.779055264 | -4.201193116 | -3.742056021 | -3.849155458 | -4.061294218 | -4.536220432 | -4.124068203 |
| TUBB1 | 2.342643806 |  |  |  | 0.0482163344 | -0.0515443915 | 1.797731868 | -0.0436481475 | 1.384259529 | 1.308121472 | 0.7448065204 | -5.400105699 | 0.452646726 | 1.039036744 | 0.4688408717 | 0.3488597799 | -0.0453929155 | 0.3445592929 | -0.172481796 |  | 4.543695822 | 5.037057518 | 4.432839019 | 4.53508901 | 4.468480357 | 5.671583783 | -3.378418243 | -3.051457662 | -3.386960794 | -3.374973851 | -3.353090632 | -3.107680962 |
| S100A6 | 0.8621356641 | 0.5075133496 | 3.197901561 | 3.106793435 | 0.024263895 | 1.337839397 | 0.7526020832 | -1.222648933 | 2.720990122 | 0.9821005999 | 0.2033773492 | -0.469208912 | 0.9130984967 | 0.2235113238 | -1.26611171 | 0.0754066756 | -0.2845839182 | 0.1247864953 | 0.0669290915 | -0.2320599148 | 4.109513806 | 4.086278068 | 4.57056655 | 4.447841459 | 5.347349998 | 4.979215591 | -3.47427457 | -3.189985059 | -3.270637244 | -3.76422995 | -3.447008672 | -3.468297739 |
| GSR | -0.2857578682 | 0.7142421318 | 1.714242132 | 2.714242132 | 0.0550952322 | 1.729889379 | -0.1822904765 | -0.374939236 | 2.377720511 | 1.767404632 | 0.5896070362 | -1.496298709 | -0.3100702392 | 0.2656928356 | 0.469740787 | -0.0687528657 | 0.2486215802 | 0.3194698918 | 0.1900392007 | -0.0665140214 | 4.243616548 | 4.221209678 | 4.136586343 | 3.848766406 | 4.060172234 | 4.724753324 | -3.438222159 | -2.79571149 | -3.309974352 | -3.163325086 | -3.124546026 | -2.762782514 |
| DNMT3A | 2.148471908 | 3.148471908 | 4.148471908 | 5.148471908 | 0.4852378469 | 0.5374684641 | 1.68140976 | 1.116424362 | 0.3409341392 | 0.2735945778 | 0.2291532454 | 0.0537892215 | 0.9111902233 | 0.1968334125 | 0.6359935049 | 0.2320787243 | 0.1650514556 | -0.01883405913 | 0.1299381073 | -0.9274586507 | -2.353417361 | -2.397678634 | -2.845678508 | -2.080720608 | -1.892700969 | -1.396285485 | 2.186900537 | 2.744961417 | 2.460868017 | 2.845068613 | 2.414739352 | 2.741335643 |
| STK17B | -2.074569127 | -1.074569127 | -0.0745691268 | 0.9254308732 | 0.0136801184 | -0.2038496093 | -1.859662129 | -0.9936077667 | 0.1988640701 | 0.9773403264 | -0.0915861625 | -1.057485781 | -1.423124838 | -0.0391383657 | 1.690641387 | 0.0094532862 | 0.1674372602 | 0.01426292353 | -0.2599512706 | -0.9621719223 | 2.107299198 | 1.948463119 | 1.74880151 | 1.734081995 | 1.784099359 | 2.034253081 | -2.82919536 | -3.668683843 | -2.622212853 | -3.159058294 | -3.378438109 | -3.672018371 |
| PTGER4 | -0.654577805 | 1.843391298 | 1.638043832 | 3.254917478 | 0.0119860079 | -0.8019414973 | -0.6932995121 | -0.0463042674 | -0.3689269901 | 1.483678054 | -0.1458255427 | -0.444942774 | -1.841754838 | 0.0615676944 | 2.916894204 | -0.1575780514 | 0.249250324 | -0.4159102407 | -0.6264436746 | -0.5565413002 | -5.686182659 | -6.00321954 | -5.527022081 | -5.648614899 | -5.358780231 | -5.587951925 | 5.388352538 | 5.744973518 | 5.594685893 | 5.569064115 | 5.756540218 | 5.735097196 |
| MCL1 | 1.872246224 |  |  |  | 0.5557110319 | 1.412213946 | 1.355120192 | -0.0663467774 | 1.491357842 | 2.329331882 | 0.2667662533 | -0.593769089 | -0.2455907521 | 0.059929063 | 1.122854402 | 0.1250966248 | 0.9087530565 | 0.06432341043 | -0.2638692118 | -0.2064106562 | 3.285245731 | 3.220013271 | 3.317271871 | 2.811766389 | 3.400421053 | 2.944157202 | -2.420990939 | -3.177980661 | -3.396082078 | -3.080259507 | -3.435285173 | -3.068622454 |
| SORD | -0.1265315546 | 0.8734684454 | 1.873468445 | 2.873468445 | 0.1777774832 | 0.8022808973 | 0.0328966602 | 0.619597229 | 1.954769891 | 1.264122391 | 0.2060888785 | -3.914784576 | -0.0311011187 | 1.15085006 | 0.9990623671 | -0.0986900666 | 0.4027868838 | -0.02585164866 | -0.0391231493 |  | 4.739676257 | 4.799816374 | 4.676846132 | 5.469718095 | 5.339428586 | 6.264549778 | -3.331236117 | -3.862700565 | -3.517381189 | -3.509351266 | -3.900739181 | -3.690963678 |
| WAS | -1.168014973 | -0.1680149727 | 0.8319850273 | 1.831985027 | 0.2117740631 | 0.4767766306 | -1.656243135 | 0.4759255104 | 0.5282516075 | 1.290211047 | -0.220321708 | -0.9806195233 | -1.427154602 | 0.4338002417 | 2.065966045 | 0.024786173 | 0.172494003 | -0.07187901625 | -0.1318052172 |  | 3.093775513 | 3.310263451 | 3.134799727 | 2.825740207 | 3.37314328 | 3.837370113 | -1.938989236 | -1.823531392 | -1.920810832 | -1.959441504 | -2.232016086 | -2.173053468 |
| BMP4 | 3.816518599 | 1.012193106 | 0.4877118432 | 3.065681576 | 0.0124930759 | 0.3247823356 | 2.546957446 | 0.0219313725 | 0.3465845484 | -0.5234327705 | 0.4845773893 | 1.066722904 | 2.157664024 | 0.129693231 | -1.386066744 | 0.1433188836 | -0.2286575501 | 0.1304929178 | 0.10282857 | -0.5872344444 | -1.799533785 | -1.962482343 | -2.323466344 | -1.878811847 | -0.8571279568 | -1.711249552 | 2.480571056 | 2.208236386 | 2.249979279 | 2.132571142 | 2.691074143 | 2.491444605 |
| CA14 | -1.45411842 | 1.084505088 | 2.529258046 | 2.865070544 | 0.3525981178 | -0.1961586346 | -0.9563637323 | -0.6916319589 | -0.1778381194 | 0.5153140016 | -0.1044430326 | 0.5412687807 | -1.137518936 | -0.2487843479 | 1.188581692 | 0.0027303991 | -0.126272374 | 1.096020778 | 1.200222589 | 0.2866060479 | -2.086667549 | -2.338224363 | -2.458047978 | -1.317623898 | -1.574103516 | -1.582764039 | 0.5670239723 | 1.320461397 | 1.173173916 | 1.243753175 | -0.2992947214 | 1.099447061 |
| RAC2 | -0.7656249435 | 1.062724316 |  |  | 0.0025955765 | 0.7401191751 | -0.5084326605 | -0.057690984 | 1.000583994 | 1.881103965 | -0.0674803221 | -1.800949031 | -1.64259723 | 0.355454531 | 2.384895192 | -0.5101147332 | 0.5974012597 | -0.2321381952 | -0.4199442967 | -0.0708361059 | 3.186493832 | 2.890465897 | 2.978160989 | 3.136395634 | 3.543641273 | 3.394092494 | -3.312460767 | -3.256411825 | -3.195995588 | -3.296498245 | -3.313324046 | -3.23428233 |
| CYBB | -0.7069883169 | 0.2930116831 | 1.293011683 | 2.293011683 | 0.0661160724 | 1.215243363 | -0.9190023499 | 0.5217289982 | -0.0911481508 | 0.8180855323 | -0.0896992712 | -2.172030669 | -0.9518386382 | -0.0733649149 | 1.389996972 | 0.0712002548 | 1.572704392 | -0.1050884133 | -0.2018882728 | 1.281194359 | 2.211734999 | 1.678090177 | 2.457929692 | 1.065779022 | 2.33351212 | 0.7664217477 | -1.349921759 | -1.425183165 | -1.627302348 | -1.454096205 | -2.216423848 | -1.648627528 |
| CYBA | -0.0832293511 | 0.9167706489 | 1.916770649 | 2.916770649 | 0.0871072151 | 0.7406708513 | -0.3069942967 | 0.1782968899 | 1.083290855 | 1.041247378 | 0.0564382086 | -1.246476457 | -0.3954438422 | 0.4729292387 | 1.046142041 | -0.0362500411 | 0.7179558826 | 0.005460014211 | -0.1506518936 | 0.7227163962 | 3.636087673 | 3.692152574 | 4.197881226 | 3.663813561 | 4.314955011 | 4.085324316 | -3.602847419 | -3.927932069 | -3.379624937 | -2.935790137 | -4.215978038 | -3.660894952 |
| NDUFB9 | 2.88837909 | 3.497048934 | 2.137010349 |  | 0.564207709 | 0.5603637001 | 1.865138981 | -0.0941886206 | 0.7310729231 | -0.5920811303 | 0.2070224087 | 0.1465216353 | 1.166410729 | 0.3796846151 | -1.556961124 | 0.1095110912 | 0.0563429529 | 0.08430686695 | 0.206198854 | -0.0062813793 | 3.373601302 | 3.336099101 | 3.498097786 | 3.670545307 | 3.675129743 | 3.636185531 | -3.63486564 | -3.325518463 | -3.504105442 | -3.590479864 | -3.520776926 | -3.193003285 |
| PTPN6 | -0.7406728978 | 0.8628653111 | 1.602387713 | 2.923435994 | -0.003594649 | 1.104584841 | -1.015180029 | 0.0512979443 | 0.1771785474 | 0.7901635106 | -0.3663526372 | -4.126052149 | -0.8515498596 | -0.1885800617 | 1.104515141 | -0.5086320151 | 0.9858261715 | 0.04757481969 | -0.1693610868 |  | 2.229241116 | 1.876553066 | 2.328168958 | 1.496738099 | 2.187931496 | 1.705102027 | -3.599895241 | -3.301847616 | -3.250281875 | -3.172560215 | -3.896955358 | -3.427351144 |
| CD79B | -0.784578699 | 0.215421301 | 1.215421301 | 2.215421301 | 0.0024719071 | -5.38279405 | -0.8981891021 | -0.1657923033 | -2.19544923 | 0.056932619 | -1.058183369 | -0.8590296564 | -1.052279699 | -0.3562438783 | 1.811546692 | -0.193643021 | 0.0642812953 | -0.330970062 | -0.4326926781 | -1.420103022 | 1.690159133 | 0.4700660344 | -0.02800580899 | 1.503203853 | 0.534764593 | -1.251367142 | -3.166199756 | -3.588903086 | -3.371817032 | -3.088659382 | -4.385441076 | -4.244859869 |
| G6PD | -1.056388465 | -0.0563884647 | 0.9436115353 | 1.943611535 | 0.0592299775 | 1.494109869 | -0.27464498 | 0.4910321714 | 1.516527216 | 0.4920751417 | 0.1830852878 | -1.008269458 | 0.5522715168 | 0.1330240789 | -0.4191916037 | -0.0289148446 | 0.6192255025 | 0.06150942304 | 0.4122418675 | 0.0925407031 | 3.935520197 | 3.957327123 | 3.989181 | 3.892925872 | 4.302455221 | 4.419273613 | -2.828514414 | -3.438027388 | -3.071160204 | -3.142550718 | -3.64646655 | -3.520841093 |
| IFNGR1 | -0.9109463007 |  |  |  | 0.0016917327 | 0.8497235298 | -1.01649601 | 0.0037561191 | 0.2010477709 | 1.183003521 | 0.1227197759 | -1.361652414 | -1.008994864 | 0.084353079 | 1.63820103 | -0.1381013694 | 0.4279397477 | 0.2867511332 | 0.0514747843 |  | 2.370078323 | 2.171154206 | 2.350808593 | 2.52497767 | 2.885689987 | 2.304465432 | -2.339235435 | -2.57478645 | -2.737577066 | -2.91516224 | -2.650047189 | -2.489215749 |
| RDH12 | 0.5495275737 |  | 1.92581192 |  | -0.0903537072 | 1.4350536 | -0.2897819061 | -0.8280913374 | 1.067121075 | -0.2389768218 | 0.1831549287 | -1.776125566 | 0.0022353347 | -0.3675641523 | -1.050088835 | 0.7241244861 | 0.154360126 | 0.2404022962 | 0.3190192785 |  | 2.584991338 | 2.792986028 | 2.47311009 | 2.193478413 | 2.230246223 | 2.337389202 | -3.204013489 | -2.84930396 | -3.100520208 | -2.97739658 | -2.591221525 | -2.826392223 |
| INPP5D | -0.2490637433 | 0.7509362567 | 1.750936257 | 2.750936257 | 0.2630727967 | -0.1769649548 | -0.4562452416 | 0.1576332047 | -0.0985473425 | 0.64452294 | 0.3076272742 | -1.813386046 | -0.7144037441 | 0.1456898986 | 1.188438887 | 0.2099209481 | 0.2055550429 | -0.2630926607 | -0.2414211828 | -1.497722732 | 2.876836079 | 2.971428502 | 2.190686275 | 2.93695373 | 3.342286311 | 3.952675508 | -1.840794054 | -1.609765667 | -1.989213412 | -1.676119439 | -1.789431017 | -1.51949771 |
| TEC | 1.144944604 | 2.144944604 | 3.144944604 | 4.144944604 | 0.4484432515 | -1.383577815 | -0.256334398 | -0.2183513728 | -1.154689268 | 0.2107697697 | -0.0074865877 | -2.892308482 | 0.2463395737 | -0.118476277 | 1.49384028 | -0.1132190428 | -0.1488023159 | -0.005257090662 | -0.1188311326 | -1.379834516 | 0.5073420254 | -0.2851525915 | -0.001331336381 | 0.8498652217 | 1.121202358 | 1.429242894 | -1.380580989 | -1.661941909 | -1.513859807 | -1.608884015 | -1.580445947 | -1.630473109 |
| EPHB6 | 0.7918197448 |  | 4.538952402 | 3.469019969 | 0.29348936 | -1.238151478 | 0.3557320894 | -2.251892224 | 0.2782433484 | 0.458258966 | 0.2089694777 | -2.489585473 | 0.2974837298 | 0.0859629523 | 0.2264214856 | 0.2089227312 | -0.706847545 | -0.04802033461 | 0.266298206 | -0.0833562914 | 3.448099715 | 3.735922649 | 3.448309444 | 3.691220545 | 1.800578853 | 2.70409699 | -4.337497014 | -4.219776265 | -4.04348872 | -3.472036111 | -4.504459448 | -4.267140449 |
| RPS9 | -0.1342057497 | 1.140039913 | 2.136067423 | 3.878173912 | -0.1083688946 | 0.185058798 | -0.4165814665 | -1.167756786 | 0.4411198504 | 0.4511578684 | 0.0211627169 | -2.11635203 | -0.1742794532 | 0.3076198398 | 0.5640915851 | 0.1880882238 | 0.7132351938 | -0.02910134254 | -0.2080430621 |  | 3.21396546 | 3.183544759 | 3.371915221 | 2.543603213 | 2.234848955 | 1.868554804 | -3.282328313 | -2.87029536 | -2.951505461 | -2.90492147 | -2.831502004 | -3.136561401 |
| SNCA | -1.642344093 | -0.6423440927 | 0.3576559073 | 1.357655907 | 0.0521219653 | -0.5290244283 | -0.7753267279 | -1.174143476 | 0.1726520405 | 2.355705652 | 0.6125400722 | 0.1077959673 | -0.1701043947 | 0.0637932074 | 3.848122716 | 0.2536610118 | -0.1805226047 | 0.77837962 | 0.524402023 | 0.6086154575 | 5.675657948 | 5.504586026 | 5.528377924 | 4.5561472 | 2.678489121 | 3.968608221 | -6.05985367 | -6.166981688 | -6.120490116 | -6.114810108 | -5.869420074 | -6.063489824 |
| HMMR | 0.9006868018 | 1.900686802 | 2.900686802 | 3.900686802 | -0.038688564 | 0.4222641786 | 0.5292022909 | 0.6183538424 | 0.0894729611 | -0.5912049979 | 0.2849803331 | 1.06050038 | 0.7036866322 | 0.1374973375 | -1.026812851 | 0.222930068 | -0.2012900037 | 0.355078233 | 0.4148830025 | -0.5325173854 | -2.248825573 | -2.152324817 | -1.562211227 | -1.819710873 | -1.687066699 | -1.192558741 | 2.456140756 | 1.983803717 | 2.134679876 | 2.162303864 | 2.343391502 | 2.267620605 |
| SYK | 0.406662289 | 1.406662289 | 2.406662289 | 3.406662289 | 0.0940631615 | 0.5473405552 | -0.2504433605 | -0.6010279372 | -0.1398080387 | 0.4310697427 | 0.0032046963 | -2.057005148 | -0.0776048905 | -0.2477999271 | 0.6720688495 | 0.3959131348 | 0.3145933541 | 0.02396488919 | -0.2128506812 | -1.079965261 | 3.288261024 | 3.479836669 | 3.44537473 | 3.323388171 | 3.709017264 | 3.980847307 | -3.439849084 | -2.914124055 | -3.150590935 | -3.205614746 | -3.140861222 | -3.027778092 |
| RAB8B | 0.0951293902 | 1.09512939 | 2.09512939 | 3.09512939 | 0.2770689199 | 0.9147146454 | -0.3139459607 | 0.5611603083 | 1.38966063 | 0.5076457659 | 0.2037771826 | 0.3314250665 | 0.2008135798 | -0.0981014035 | -1.09213681 | 0.3442263499 | 0.0028730468 |  |  | 0.1913357151 | 3.822608115 | 3.811333572 | 3.73795473 | 3.515060723 | 3.990597653 | 4.50088489 | -2.264165467 | -2.88403728 | -2.357802664 | -2.600289628 | -2.559768432 | -2.779723832 |
| CRABP1 | 1.661593417 |  |  |  | -0.0221682048 | -0.648033583 | 1.072106864 | -0.9868681421 | 0.3504976261 | -1.153423401 | 0.3754018582 | 0.0454837469 | 1.17561098 | 0.348426195 | -2.126495672 | 0.1044476589 | -0.7857514613 | 0.3362785597 | 0.6390018678 | 0.191506343 | -3.120577167 | -3.30042792 | -3.245563179 | -3.371742308 | -3.490727786 | -3.405442752 | 2.83375098 | 3.120726853 | 2.896846234 | 2.9440437 | 2.460885067 | 3.028301725 |
| RRM2 | 1.136250428 | 2.601957802 |  |  | -0.0004516315 | -0.1487560299 | 1.791125506 | -0.9861456443 | 0.1068289172 | -0.5656293892 | 0.3288478753 | 0.4958969482 | 1.110419779 | 0.2271312881 | -1.246118068 | 0.0586073784 | -0.5855853364 | 0.1152756453 | 0.4321424756 | -0.1774379465 | 2.700245744 | 2.84475014 | 3.033150147 | 2.843430067 | 2.009253022 | 3.071325899 | -1.57540603 | -1.642060124 | -1.777055115 | -1.822633358 | -1.043663507 | -1.317619734 |
| ABCC9 | 3.843243962 | 3.620000522 |  | 2.539476897 | 0.3628117243 | -0.321563143 | 2.864575855 | 0.065002852 | 0.2663992018 | 0.6825101979 | 0.0016819811 | -0.3654160903 | 1.712771303 | 0.4583718977 | 0.5093938145 | 0.047320862 | -0.1140013009 | 0.08151311909 | 0.009460814 | -0.0430356674 | -2.98434869 | -2.895370364 | -2.756892659 | -2.859251378 | -2.929133694 | -2.807612306 | 3.927142852 | 2.756950328 | 3.381247489 | 3.288003323 | 3.546773491 | 3.507167032 |
| NDUFS4 | -1.054088552 | 1.031032811 | 2.275560172 | 3.499184103 | 0.0978740507 | 0.6723381938 | -0.433535842 | 0.4720239935 | 0.7187409333 | 0.2697938825 | 0.3317566823 | -2.370613197 | -0.1970567695 | 0.5914016043 | 0.2059534894 | 0.1915093286 | 0.6130287157 | -0.00682882697 | 0.014592962 | -0.4923583434 | 4.192000668 | 4.25368937 | 3.72676794 | 4.399764632 | 4.436770498 | 4.720445515 | -3.097556564 | -3.332864445 | -3.174370096 | -3.183681582 | -3.580884184 | -3.578126684 |
| FARSB | 1.250906953 | 2.250906953 | 3.250906953 | 4.250906953 | -0.0202865099 | -1.254297105 | 0.574141621 | -1.098299952 | -1.763386766 | -1.017602759 | -0.4561119694 | -2.142753675 | 0.3246321052 | 0.1025504181 | -0.1125071621 | 0.2097434632 | -0.3446519807 |  |  |  | 0.6558271661 | 0.1777370983 | 0.2520383479 | 1.043707164 | 1.01508404 | 0.03678614416 | -2.118259428 | -2.0581637 | -1.756797517 | -2.018938775 | -2.030363295 | -2.082404137 |
| SERPINE1 | 2.109079049 |  |  |  | -0.0785096599 | 0.5301115991 | 1.144729386 | 0.30823034 | 0.2111923186 | -1.888371692 | 0.5642386707 | 0.094485371 | 1.891119076 | 0.1051379411 | -3.341094578 | 0.2690454886 | -0.2252954895 | 0.1734153806 | 0.4720379463 |  | -2.850369504 | -2.97783272 | -1.932589579 | -2.439043417 | -2.149637974 | -1.983216111 | 2.082048888 | 2.558551127 | 2.540253449 | 2.346617888 | 2.297075532 | 2.532718755 |
| RALB | -0.2633462243 | 0.7366537757 | 1.736653776 | 2.736653776 | -0.2608079651 | 0.6772883135 | -0.2754295333 | -0.2489126521 | 1.008983509 | 1.034079985 | 0.1671504756 | 2.25420551 | -0.5861752266 | 0.1152926041 | 0.572563808 | -0.0643547582 | 0.3283479484 | 0.02826951616 | 0.0154781467 | -0.1598080731 | 3.223005241 | 3.271557247 | 3.667149777 | 3.48918317 | 4.226565152 | 3.870228788 | -1.830852854 | -1.494610847 | -1.97557693 | -1.834068883 | -1.669647497 | -2.111785076 |
| MPL | -0.5808449379 | 0.4191550621 | 1.419155062 | 2.419155062 | -0.0200145063 | -0.0295065372 | -0.3626372561 | 0.2796137071 | 2.185667493 | 2.064359185 | 0.662086472 | 1.95422419 | -0.7146728709 | 1.419631128 | 2.43825377 | 0.7155489587 | 0.2723113415 | 0.7441511602 | 0.6151169386 | -0.4487327335 | 2.098436638 | 2.595514428 | 1.447679623 | 0.5822205657 | 0.5198792806 | 2.075077636 | 2.072035006 | 1.625717479 | 1.557209441 | 1.672442468 | 2.348038942 | 1.835034137 |
| ANXA2 | 1.262696929 | 0.7619834548 |  |  | -0.0010418871 | 0.8319229859 | 1.390694848 | -0.8177473453 | 1.814299694 | 0.3025461575 | 0.0525029578 | -0.8161906655 | 1.291702152 | -0.0760456261 | -1.899746739 | -0.120093072 | -0.2911749794 | 0.1142934455 | 0.0875600663 | -0.1272590524 | 1.243074511 | 1.216675242 | 1.477123061 | 1.371809434 | 2.000634666 | 1.820446917 | -2.262669581 | -1.188853498 | -1.0890295 | -0.9583949373 | -2.838653598 | -2.162045341 |
| UNG | 1.442903866 | 2.442903866 | 3.442903866 | 4.442903866 | 0.1676702721 | -0.3218276216 | 1.144343279 | 0.1479148086 | -0.6061114899 | -0.7279653878 | -0.061548958 | 1.91267214 | 0.8219948166 | 0.0158909014 | -1.000406529 | -0.0407287069 | -0.2235908003 | -0.006416345663 | 0.0001923422 |  | 2.861440504 | 3.274179738 | 3.298010288 | -0.541830355 | -0.3491800319 | -0.3357720018 | -3.793467027 | -3.578094106 | -3.652962073 | -3.94633933 | -3.754811567 | -3.776804973 |
| LGALS3 | 0.9372113747 | 0.4501499155 |  |  | 1.180898279 | 1.409409477 | 0.7929513641 | -0.0085491455 | 1.838122619 | 1.035283298 | 0.0420131827 | 0.9833173073 | 0.8531229613 | 0.0343858227 | -0.4072179255 | 0.0288257403 | 0.3237333891 | 0.02970628467 | -0.0237684158 | 0.9503549626 | 3.38704246 | 3.08500963 | 3.47703891 | 3.446544672 | 3.815728303 | 3.251804904 | -2.510318341 | -2.467060258 | -2.911458684 | -2.937198487 | -2.459292192 | -3.009913294 |
| NAPEPLD | 0.4095866586 | 1.409586659 | 2.409586659 | 3.409586659 | -0.2248745975 | 0.03743511 | 0.1516165343 | 0.5234192731 | 1.164091787 | -0.0737190968 | 0.1987276685 | 0.3203911879 | 0.1000580704 | 0.4134232056 | -0.2594197482 | -0.0476680837 | 0.3120614084 |  |  |  | -2.441263439 | -2.991926259 | -2.111648965 | -2.128760935 | -1.996344958 | -2.00992157 | 1.774930024 | 2.123955983 | 2.001575793 | 1.988262555 | 1.607810689 | 1.709503018 |
| CACNG5 | -1.360759355 | 0.5687372317 |  | 2.7895903 | 0.0721735029 | -1.135157866 | -0.2931218939 | -1.067358932 | 0.0940957394 | -0.209981626 | -0.1138605956 | -0.6671237458 | 0.2507509161 | -0.3263296568 | -0.344454584 | 0.1549078942 | -0.7286624733 | 0.01983331062 | -0.1757075199 |  | -3.415520315 | -3.495973712 | -3.637145169 | -3.688160722 | -3.424655014 | -3.507105936 | 2.827131343 | 3.036006295 | 2.915080872 | 3.082905264 | 2.817548183 | 3.040967796 |
| ACTB | -0.5327793218 | 0.4672206782 | 1.467220678 | 2.467220678 | -0.0002737216 | 1.344886836 | -0.6630561935 | 0.323992106 | 0.2568327641 | 0.7026741137 | -0.0930935396 | -1.830996197 | -0.5015969177 | -0.0330567975 | 0.9115310002 | 0.0771230445 | 0.8209977464 | 0.09866861991 | 0.0391853802 | -0.6315657839 | 4.716638898 | 4.554708394 | 4.895499235 | 5.349828703 | 4.83619725 | 5.574862873 | -2.031587254 | -3.701033965 | -3.407849284 | -3.194365814 | -2.536882161 | -2.93070574 |
| TAGLN2 | 0.6219019458 |  |  |  | -0.0906827669 | 1.60786493 | 0.4692127864 | 2.321789626 | 1.049243926 | 1.03135145 | 0.4245645391 | -0.4883477305 | 0.0947853744 | 0.3780441694 | 0.3640429484 | -0.1238050092 | 0.9990889205 | -0.04846331083 | 0.0127001476 |  | 5.484606564 | 5.510730922 | 5.016696022 | 6.509154373 | 6.961379701 | 7.182516354 | -3.850612812 | -4.127627815 | -3.709418758 | -3.943976996 | -3.83191175 | -3.912160151 |
| PPP2R5C | 0.0603240849 | 1.060324085 | 2.060324085 | 3.060324085 | 0.1558211293 | -0.006323208 | -0.0720521541 | 0.1599344719 | 0.0446555296 | 0.0449930853 | 0.2295448897 | -1.092903195 | -0.0790340954 | -0.0995455204 | 0.1211674279 | -0.1506123295 | 0.2528646926 | 0.1957788899 | 0.2716708576 | -0.1128802222 | 2.423948414 | 2.577666839 | 2.469033789 | 2.270554199 | 2.789806557 | 3.117618903 | -2.254835868 | -2.524773005 | -2.208760333 | -2.145713512 | -2.284970041 | -2.298772059 |
| RAB5A | 0.1831027032 | 1.183102703 | 2.183102703 | 3.183102703 | 0.0118051107 | 0.612151902 | -0.076811879 | 0.4743536106 | -0.2090037523 | -0.1314063113 | -0.0582192601 | 1.560903267 | -0.3149863205 | -0.2511465427 | -0.3032574757 | 0.1983865297 | 0.6930085897 | -0.04948774671 | -0.1593865638 | -0.2072351287 | 2.205142212 | 2.566846616 | 2.434951337 | 1.82907066 | 2.21388087 | 2.806686209 | -1.422857233 | -1.494689565 | -1.49816766 | -1.353380972 | -1.420408867 | -1.379606505 |
| RAN | 0.7170138236 | 1.717013824 | 2.717013824 | 3.717013824 | 0.0375013219 | -0.1224279114 | 1.103902691 | -0.4177106114 | -0.1834688971 | 0.0624414294 | 0.0613668326 | -0.2309170414 | 0.6420032932 | 0.0465940003 | 0.3517770865 | 0.0245590691 | -0.0563524562 | 0.2410403184 | 0.3519259634 | -0.7481059474 | -4.045723415 | -3.82299401 | -3.611154449 | -4.046919696 | -3.947195625 | -4.017735389 | 2.4365532 | 3.708113675 | 3.315666176 | 3.490941367 | 1.61444139 | 2.497275082 |
| LSM6 | 1.187376744 |  | 3.93476121 | 3.719718438 | 0.2415533321 | -0.1128708841 | 0.5200668923 | -0.0389500018 | -0.1741936795 | -0.1952543566 | -0.1453504617 | 0.2047634329 | 0.288980578 | 0.0547016167 | -0.2784091199 | -0.0759260194 | 0.1700273416 |  |  | -1.119192199 | 3.181275088 | 3.174565736 | 2.813573413 | 2.722234727 | 2.301734964 | 1.969785041 | -3.251236448 | -3.382826705 | -3.062933688 | -3.375738393 | -3.404244238 | -3.549118182 |
| SDHB | 0.6969103749 | 1.696910375 | 2.696910375 | 3.696910375 | -0.0139472192 | 0.088481433 | 0.0039443586 | -0.7167411653 | 0.1191388513 | 0.5267177956 | -0.1131040791 | -1.232406901 | -0.3913781536 | 0.2290879856 | 0.5549823688 | 0.0325760705 | 0.182492889 | 0.3394985305 | 0.2977904419 | -0.1066760088 | 2.784481179 | 2.627792845 | 2.583853122 | 3.440887964 | 3.381692997 | 2.958321582 | -2.717052642 | -2.936306781 | -3.078990131 | -3.049370826 | -3.02104374 | -3.402824108 |
| PML | -0.5065427755 | 0.4934572245 | 1.493457225 | 2.493457225 | 0.0330820442 | -1.422074022 | -0.6411750485 | -0.3290178105 | -1.186076233 | -0.4859775061 | 0.0308197657 | -2.109539287 | -0.3114957566 | -0.06156884 | 0.4222016759 | -0.2112287808 | -0.2283759292 | 0.1095653483 | 0.2045422536 | -1.535710514 | 1.690098103 | 1.473159706 | 2.048726374 | 0.918064104 | 1.394141197 | 0.850016387 | -2.005857409 | -2.388841469 | -2.074103179 | -2.099789981 | -2.142867748 | -2.246158817 |
| SLC12A6 | 0.9982994329 | 0.6881851049 | 2.557989481 | 3.685684971 | 0.0206216739 | -0.29314811 | 0.7308044078 | -0.2195952647 | -0.2237292016 | 0.1115920779 | 0.023953999 | 1.675608341 | 0.0346743245 | 0.1763239504 | 0.3142806476 | -0.0245115618 | 0.0704224728 | 0.1808745858 | 0.2429581734 | -1.092057257 | 1.744454583 | 1.729634389 | 1.534054978 | 1.625737761 | 2.279788382 | 2.842148915 | -1.066665157 | -1.568666378 | -1.533085844 | -1.555568708 | -2.115076287 | -2.151743975 |
| CDK15 | 1.487898944 | 1.651369436 | 1.890636781 | 3.186181353 | 0.1244105527 | 0.240527497 | 0.5886350348 | 0.313292314 | 0.0479056476 | 0.0628409395 | 0.008718743 | 0.110885107 | 0.5287378587 | -0.3008232635 | 0.1231917388 | 0.1184583488 | 0.3113395134 |  |  |  | -3.374321982 | -2.88094433 | -3.150296663 | -3.079958315 | -3.386030008 | -3.182108304 | 2.42379615 | 3.181926897 | 3.391573947 | 3.210343285 | 2.776812776 | 2.901119807 |
| TCN2 | 0.9395508399 |  |  |  | 0.1234428793 | 1.52757514 | 0.1757521121 | -0.3793707905 | 1.178517411 | 1.290528647 | 0.3839652831 | -1.341524424 | 0.0628670458 | 0.0839530132 | 0.7519277491 | 0.2600763775 | 0.6540371818 | -0.1745462846 | -0.0829217136 | 0.571911618 | 2.395874686 | 2.04801464 | 3.346503232 | 2.6659574 | 4.086449464 | 2.753196609 | -1.83529008 | -1.044461284 | -1.436602724 | -1.181855687 | -1.783853147 | -2.250875692 |
| MOCOS | 1.659146956 | 2.659146956 | 3.659146956 | 4.659146956 | 0.1033287723 | 2.334555977 | 0.4652209454 | 0.6646976612 | 0.416126572 | -0.2285933715 | 0.1017582445 | 0.3149250526 | 0.1696643003 | 0.0012862324 | -0.9371299803 | 0.2548616664 | 0.4093467185 |  |  |  | -1.743642172 | -1.453739541 | -1.389700835 | -1.811007176 | -0.9895763671 | -1.299806852 | 3.33747597 | 3.080823015 | 3.230566263 | 3.393675385 | 3.397786556 | 3.332660357 |
| CALR | 0.7660567074 | 1.766056707 | 2.766056707 | 3.766056707 | -0.0014289541 | -0.6695835821 | 0.4726469165 | -0.4079396746 | -0.0235352334 | -0.3401968484 | -0.0606075494 | -1.149325923 | 0.5625200384 | 0.2448784654 | -0.7228186334 | -0.1361148768 | -0.8424249694 | 0.1947943288 | 0.1875583254 | -0.0133050635 | 3.449574861 | 3.430569023 | 4.011150791 | 3.362146777 | 3.793997265 | 3.410758858 | -2.678447704 | -3.249194429 | -2.863085657 | -3.197412916 | -3.077451389 | -3.182264328 |
| CANX | 0.317219177 | 1.317219177 | 2.317219177 | 3.317219177 | -0.0390048586 | 0.48122607 | 0.256414367 | 0.2136448977 | -0.5020870098 | -0.5739556309 | 0.0207348693 | 2.311909461 | 0.4017454411 | -0.4719661621 | -0.9839732004 | -0.0656733031 | 0.0733401276 | 0.1951290112 | 0.321029069 | -0.1641237753 | 1.923437363 | 2.033118982 | 2.673110857 | 2.392914192 | 2.946967319 | 2.251424706 | -2.835315229 | -2.595605444 | -2.514783876 | -2.515233168 | -2.492702897 | -2.424909322 |
| TSHR | 1.56838334 | 2.56838334 | 3.56838334 | 4.56838334 | 0.1359655982 | 0.9086266807 | 0.7864258067 | 0.3291895607 | -0.2767155607 | 0.0248933669 | 0.1089293408 | 0.3806626553 | 0.1877076033 | 0.008328037 | 0.0751599133 | 0.1610155713 | 0.25644185 | 0.1730664909 | 0.2336725305 | 0.1021548983 | -2.677006672 | -3.041124816 | -3.107991914 | -3.013351462 | -3.372472165 | -3.157285318 | 1.931018907 | 2.74846065 | 2.493481385 | 2.545091522 | 1.429614925 | 2.31117236 |
| PI4KA | 0.470360054 | 1.470360054 | 2.470360054 | 3.470360054 | 0.6773529717 | -0.4444611249 | 0.8167494444 | 0.1588291702 | -0.1058973603 | 0.4982219165 | 0.2046116235 | -1.252486209 | 0.4612072484 | -0.0072781545 | 0.9414970057 | -0.0311457676 | 0.0628968606 |  |  |  | 1.969345166 | 2.201244012 | 2.096910171 | 2.018306504 | 2.634731415 | 2.681985967 | -2.079420857 | -1.575498261 | -2.269119259 | -2.14127047 | -1.955549681 | -2.296240321 |
| CBFB | 0.1284999578 | 1.128499958 | 2.128499958 | 3.128499958 | -0.0032162885 | -0.1377782853 | 0.1571968072 | -0.1636311636 | -0.3562011598 | -0.2842521018 | -0.244125382 | 1.659997297 | 0.0585970189 | -0.0833383655 | -0.1338246476 | -0.2560276081 | 0.2881057084 | -0.04472108731 | -0.0574770025 | -0.7228196783 | 1.667625691 | 1.007834331 | 1.541804198 | 1.890133542 | 2.121074494 | 1.140451629 | -3.017966942 | -2.289843417 | -2.774042218 | -3.163021954 | -2.329523425 | -1.873512633 |
| CD3G | -0.9282424028 | 0.0717575972 | 1.071757597 | 2.071757597 | 0.0100788385 | -0.3705653926 | -0.9895660421 | 0.3505575043 | -0.4456877868 | 0.7544839422 | -0.1495409696 | -2.982563768 | -1.234364103 | 0.0073191086 | 1.868375443 | -0.07557062 | 1.321457386 | -0.2607222242 | -0.3978101798 | 0.0205124647 | 1.299068921 | -0.3971397776 | -1.877764383 | 2.695829904 | 0.5917132176 | 0.04582087642 | -1.891842072 | -1.664116504 | -1.862311124 | -1.853803965 | -1.485949091 | -1.68384768 |
| S100A8 |  |  | 2.862135664 | 2.50751335 |  |  |  |  |  |  |  |  |  |  |  |  |  | -0.2543083259 | -0.6819075882 | 2.189946739 | 5.396608795 | 5.074279011 | 4.904470248 | 5.511045542 | 5.457784242 | 6.080726029 | -4.198664916 | -3.844807556 | -4.065337109 | -4.07331435 | -3.601303083 | -3.818118352 |
| FPR1 | 0.3398009239 |  |  |  | -0.0288056512 | 2.039826536 | -0.2061196982 | -0.7088684907 | 1.900709287 | 1.545871682 | -0.2800784023 | 0.7148006503 | -0.2536589575 | -0.2170710102 | 0.2826066513 | 0.1716047831 | 0.4109851457 | -0.2222844229 | -0.4225555679 | -0.1912466098 | 3.437663146 | 3.519008387 | 3.665593098 | 2.92978462 | 3.408889936 | 3.660416429 | -1.263602945 | -1.250633563 | -1.295875719 | -1.265475873 | -1.345438742 | -1.121289873 |
| NCF1 | -0.2149835936 |  |  |  | -0.0644449647 | 0.5413247492 | -0.0579936692 | 0.161217568 | 1.208130984 | 1.964945687 | 0.178742353 | 0.3098714332 | -0.1465653574 | -0.1498586804 | 1.329754696 | 0.2086795173 | 0.0349090225 | -0.2759858777 | -0.6123876649 |  | 2.879259854 | 3.091892466 | 3.333001434 | 2.162628937 | 3.166443539 | 2.8304786 | -2.884576919 | -2.49262374 | -2.615271046 | -2.439797632 | -2.144379144 | -2.413594053 |
| MAPK12 | 0.4071314848 |  | 1.72770177 | 3.316670481 | 0.3418303418 | -0.8447805898 | -0.2435102354 | -0.0799036204 | -0.0051761932 | 0.4140187047 | -0.0395614355 | -0.7601781054 | -0.3198890659 | 0.0223301299 | 0.6936760423 | 0.1414517767 | 0.0296547246 | 0.04199296957 | 0.0045525642 | -1.190107315 | -2.980411346 | -3.112298983 | -2.783470256 | -2.689190657 | -1.788744282 | -2.474859741 | 2.893110513 | 2.794500414 | 2.96174354 | 2.911841877 | 3.276253677 | 2.63768043 |
| PCMT1 | 0.1445304654 |  |  |  | 0.0324058359 | 0.4123644523 | -0.4021663813 | 0.9734774151 | 1.476154617 | 1.352873178 | 0.5214899531 | 0.3876880479 | 0.1079581382 | 0.8080206406 | 1.458272738 | 0.2543885186 | 0.1883921526 | 0.3258563313 | 0.433171922 | -0.6736471941 | 3.741525797 | 3.860398457 | 3.244159134 | 3.875956966 | 3.946018027 | 4.620685488 | -3.06046203 | -2.807179583 | -2.822565342 | -2.714446997 | -3.526098865 | -2.839892582 |
| GMPS | 0.622540982 | 1.622540982 | 2.622540982 | 3.622540982 | 0.1834378449 | -0.1245750209 | 0.8005323268 | 0.0244384693 | 0.0599691556 | -0.2271434094 | 0.1545812261 | 0.5047051327 | 0.7045349372 | 0.4287603348 | -0.3978875347 | 0.1042740084 | 0.0490857614 | 0.02821227469 | 0.0112131475 |  | -4.19348829 | -4.485866808 | -3.477405982 | -3.474726524 | -3.420820815 | -3.639293769 | 2.917625942 | 3.13915103 | 2.993458639 | 3.004230062 | 3.052455913 | 3.109467832 |
| GSTO1 | 1.088382071 |  |  |  | 0.000305762 | 1.996129511 | 0.5331481362 | 0.4700808673 | 0.420215812 | 0.1461014008 | 0.3245713971 | -1.35549228 | 0.2263809451 | -0.0436132781 | -0.2041670648 | -0.039058102 | 0.8981338311 |  |  | -0.0545090611 | 2.405054542 | 2.567297167 | 2.285691789 | 2.539204837 | 2.980483378 | 2.709949182 | -1.176653483 | -1.155635964 | -1.112023645 | -1.163347299 | -1.733932269 | -1.485272893 |
| MELK | 1.057056125 | 2.057056125 | 3.057056125 | 4.057056125 | -0.014357687 | 0.0809236469 | 1.038447425 | -0.0227077955 | 0.1144731952 | -0.7879887441 | 0.3350191068 | 1.208026907 | 0.8766738865 | 0.0792334463 | -1.604972225 | 0.067071425 | -0.2352772199 |  |  | 0.9646627577 | -1.265069859 | -1.119804501 | -0.9238456978 | -0.6772305277 | -0.5958404936 | -0.462838012 | 1.672776072 | 1.846721255 | 2.105684807 | 2.397422894 | 2.915340665 | 2.645659881 |
| VIM | 0.8506994147 | 1.850699415 | 2.850699415 | 3.850699415 | -0.00002235119797 | 0.3428418445 | 0.8077809742 | -0.6288138311 | 0.5803870669 | -0.0244306882 | 0.1137916866 | 0.062033375 | 0.9073207129 | -0.0883605328 | -0.9762803626 | -0.0507707695 | -0.1220183963 | 0.1146262577 | 0.0419004768 | -0.1618487546 | 3.021830574 | 2.929185155 | 2.535631798 | 3.22990378 | 3.55561793 | 2.640019014 | -3.270971796 | -3.085138725 | -3.070481019 | -2.74271646 | -2.666215715 | -3.14771821 |
| GLUL | -0.2731389382 | 0.7268610618 | 1.726861062 | 2.726861062 | 0.1317819207 | 0.955530258 | -0.5383945795 | 0.2836597065 | 0.2377826769 | 0.9734281537 | 0.1980725344 | -1.428254192 | -0.5933314263 | 0.1967038072 | 1.561158339 | 0.2750964508 | 1.688679487 | 0.2074783341 | 0.017825929 |  | 1.826951285 | 2.035235407 | 2.336042192 | 1.799806104 | 2.844416451 | 2.770232168 | -1.267463497 | -1.144712916 | -0.7020626435 | -0.912835455 | -1.197254164 | -1.144283899 |
| KRT9 | 0.3199452914 | 1.344855558 | 2.499671755 |  | 1.747477658 | 0.065717837 | 0.1458814253 | 0.3260209646 | 0.0684087783 | 0.0137359963 | 0.0897554058 | -0.4510370044 | -0.0340892107 | 0.1947413552 | 0.1134170812 | 0.076479579 | -0.087033013 | -0.05081889925 | -0.083643461 |  | -1.993148017 | -1.815580378 | -1.605881165 | -1.648082827 | -1.728475795 | -1.703964255 | 2.660188547 | 2.674616324 | 2.402426446 | 2.707019068 | 3.303674994 | 2.587318622 |
| NISCH | 0.5743139494 | 1.574313949 | 2.574313949 | 3.574313949 | 0.0417939897 | 0.0442767954 | 0.4365980982 | -0.2253657226 | 0.1547469011 | -0.2067050283 | 0.113455195 | -0.1434011483 | 0.5214656018 | 0.1690275584 | -0.2538373791 | 0.0605627675 | -0.2771652087 | 0.008077540786 | -0.0373976574 | -0.552766699 | 2.301304651 | 2.861161703 | 3.1239494 | 1.699378773 | 2.710041675 | 2.12468372 | -1.600293545 | -1.674284052 | -2.062757915 | -1.929082071 | -2.07952223 | -2.088940586 |
| PSMB7 | -0.3267889805 | 1.052315227 | 2.737230222 | 3.579859662 | -0.0132966432 | 0.1300653045 | -0.5058587839 | -0.2170667944 | 0.1492254335 | 0.603956599 | -0.0101960015 | -0.3841173178 | -0.4460187032 | 0.1432594378 | 0.6606604675 | 0.0918291093 | 0.215866263 | 0.1087868918 | 0.2257259912 | -0.149556885 | 2.398038215 | 2.129258842 | 2.373533705 | 2.769127421 | 2.896098932 | 2.786635142 | -2.080078727 | -2.45162168 | -2.324902391 | -2.666448985 | -1.647893524 | -2.431466964 |
| CCNA2 | 1.340070696 | 1.315523826 |  |  | 0.3879579587 | -0.4988700276 | 1.297940649 | -0.8215813084 | -0.1615313503 | -1.24987781 | 0.5039013042 | 0.6673906597 | 1.302395186 | -0.1142356463 | -2.279632748 | -0.0941200129 | -0.7171190858 | 0.2047886533 | 0.4307079515 | 0.6710958215 | 3.028219482 | 2.952150338 | 2.660023669 | 0.4577575394 | -0.730532194 | 0.1700888276 | -3.064740612 | -3.205199366 | -3.156865227 | -3.097459713 | -2.992677202 | -2.69290746 |
| TRAPPC5 | 0.1466998718 | 2.061695791 | 3.589825227 |  | 0.0505027659 | 0.0326840147 | 0.368772517 | 0.2655038878 | 0.4408389812 | -0.0295201527 | -0.2388198307 | -1.080499071 | 0.2158859635 | 0.4701196636 | -0.0260722096 | -0.0025794398 | 0.3239741908 |  |  | 0.3774144743 | 2.264537749 | 2.085925237 | 2.120704776 | 2.625465429 | 2.766963667 | 2.336265532 | -2.660106891 | -2.781186231 | -2.548086593 | -2.641058247 | -2.658990439 | -2.572193094 |
| GBA | 0.2858007698 | 1.28580077 | 2.28580077 | 3.28580077 | -0.089676206 | 0.5360314142 | 0.3094777658 | 0.0664801267 | 0.1469173206 | 0.3008330751 | 0.1446664743 | 0.1092546245 | 0.3529532823 | 0.3179592055 | 0.6986086718 | 0.0315982109 | 0.7932957311 | -0.2194213861 | -0.1505349386 | -0.7614837084 | 2.556171898 | 2.63220245 | 2.972024159 | 2.299052992 | 3.148324496 | 3.16772215 | -1.098878256 | -1.590335092 | -1.462456457 | -1.359615888 | -1.417363717 | -1.080385378 |
| ACACB | 0.4883612558 | 1.488361256 | 2.488361256 | 3.488361256 | 0.9745965869 |  | 0.6523080372 |  | 0.6460871018 | -0.4043378135 | -0.0919717985 |  | 0.7739508209 | 0.3994039263 | -0.8719493167 | 0.0232897992 |  | -0.0436301976 | 0.30814994 | 0.0035715701 | -2.934171111 | -3.171844187 | -3.232515343 | -1.908453821 | -2.047718453 | -2.32677673 | 3.3010267 | 3.19762556 | 3.261285334 | 3.283257029 | 3.734590832 | 3.598114443 |
| AR | 0.0437793023 | 1.043779302 | 2.043779302 | 3.043779302 | -0.6103409717 | 0.5209651758 | 0.0651734042 | -0.1802355346 | 0.3031316342 | 0.6096932493 | -0.1461081341 | 0.2899449727 | 0.160271219 | 0.0594005253 | 0.0993732005 | -0.256435414 | 0.2528842751 | 0.09044230415 | -0.0800075468 | -0.0935308681 | -1.932223701 | -2.990563613 | -2.470327023 | -2.756070818 | -3.092091249 | -2.978893675 | 3.57808102 | 2.747043677 | 2.797201348 | 2.564450162 | 3.535725788 | 2.917109181 |
| TNFRSF11A | 0.8161709971 | 1.816170997 | 2.816170997 | 3.816170997 | 0.0072874373 | -0.609693968 | 0.7047312489 | -1.094020453 | 0.0303228087 | 0.049295553 | -0.1360238111 | -0.6343416546 | -0.0687853065 | -0.3754219457 | -0.2525728972 | -0.0613108501 | -0.2822794594 | 0.4861543861 | 0.2986447089 | 0.3664644244 | -1.59743879 | -1.499831461 | -1.862339003 | -1.872190903 | -2.100410154 | -1.727976899 | 1.275125783 | 1.785713542 | 1.639693078 | 1.762257621 | 0.6283422608 | 1.18273397 |
| ST3GAL5 |  | 1.118745507 | 1.946733521 | 3.570280419 |  |  |  |  |  |  |  |  |  |  |  |  |  |  |  |  | 3.607848347 | 3.688710441 | 4.055733824 | 3.118145041 | 3.085191376 | 2.94956566 | -4.091877922 | -4.279999836 | -4.055994972 | -4.085845344 | -3.949748866 | -4.469393704 |
| CASP7 | 0.1838812725 | 2.470690615 |  | 3.324122629 | 0.3709423104 | 0.2540444465 | -0.1233984919 | 1.038085204 | 0.1295021127 | -0.0334138483 | 0.3083484093 | -0.5924783496 | 0.0174038025 | 0.3006296567 | 0.0812747323 | -0.0763436883 | -0.1033528145 | 0.115092728 | 0.0631476356 | -0.535480345 | -1.213065009 | -1.9821741 | -1.89649643 | -1.45597758 | -1.099784987 | -1.49495633 | 1.538107493 | 1.496030253 | 1.663237864 | 1.624269659 | 1.656973053 | 1.519291555 |
| ADAM9 | 0.7627540116 | 0.2410110045 | 1.960788245 |  | -0.0275317553 | 0.5260450226 | 0.7277514019 | 1.333706483 | 0.8797817431 | 0.0027520195 | 0.4759439214 | 3.593676348 | 0.7912061414 | 0.5274021506 | -0.6361807602 | 0.8475146267 | -0.110356636 | 0.3513114989 | 0.3889753094 | -0.6026532873 | 3.844154062 | 4.333259982 | 3.769475886 | 3.658147299 | 3.977346475 | 4.914498282 | -1.585838111 | -1.221052703 | -1.602545884 | -1.586793142 | -1.808073042 | -1.640387504 |
| ITGAL | -0.6843413142 | 0.3156586858 | 1.315658686 | 2.315658686 | 0.0013287815 | 0.6274345246 | -0.6385344938 | 0.4334412528 | 0.6593496379 | 0.9760740012 | 0.1080192397 | -2.931962016 | -0.5570739505 | 0.1946182749 | 1.190731998 | -0.3964929454 | 0.579108191 | 0.259440662 | 0.0631119925 | -0.4986631739 | 2.859480978 | 3.160279995 | 2.595519512 | 1.640497679 | 1.75916036 | 2.929230215 | -1.317335457 | -1.90995377 | -1.383187111 | -0.8586621005 | -2.30651153 | -2.270675988 |
| ISG20 | 1.19724266 |  |  |  | 0.2494059593 | -0.1492338468 | -0.3459249865 | -1.147852606 | 0.199009796 | 0.5503420824 | 0.7158567152 | -3.238446835 | -0.0280494623 | -0.307216039 | 0.4987554559 | 0.1373119358 | 0.0011188043 | -0.405417686 | -0.6589687667 |  | 5.122179991 | 5.196381385 | 5.166937647 | 5.154838078 | 4.09767012 | 4.910061479 | -6.046316429 | -5.122362713 | -5.40881528 | -5.30466122 | -6.240256026 | -5.493832506 |
| TH | -0.1074782786 | 0.8925217214 | 1.892521721 | 2.892521721 | -0.0047703033 | 0.2883036783 | -0.0152648149 | 1.12116128 | 0.737277782 | 0.4860700545 | 0.0061854282 | -0.1007331151 | 0.0265635903 | 0.1733225194 | 0.315119546 | -0.0483385148 | 0.1913325488 | -0.003182671453 | -0.0035806053 |  | -2.208006421 | -2.653276769 | -1.875982973 | -2.436989711 | -2.825987064 | -2.730220411 | 2.819885949 | 1.558413413 | 1.476327223 | 1.290541619 | 2.721039773 | 2.297812484 |
| RPL15 | 0.4522796627 | 0.8176847609 | 2.033710191 | 2.991377918 | 0.2573198293 | -0.1462105196 | -0.3980911856 | -0.8261923121 | 0.0949170393 | -0.3875568824 | 0.2094229371 | -1.261463124 | 0.1972902965 | 0.231740148 | -0.2636135839 | 0.064046515 | 0.2956548976 | 0.1413028848 | 0.1168450876 |  | 3.571672732 | 3.246241268 | 3.491629116 | 4.193691084 | 4.048067374 | 3.408763772 | -4.300759542 | -4.498808693 | -4.348260558 | -4.333671913 | -4.750172513 | -4.535397804 |
| DNTT | 0.5764394531 | 1.576439453 | 2.576439453 | 3.576439453 | 0.3749286294 | 0.7594749463 | 0.148009576 | 0.2359744199 | -0.235531145 | -0.0419214621 | 0.2095560204 | -0.265084109 | 0.5151404036 | 0.1068021661 | -0.1031897369 | 0.0038738595 | 0.2123541195 | 0.2248548431 | 0.3118805717 | -0.5307056414 | -3.954202226 | -4.064149275 | -3.621548559 | -3.663488434 | -3.952196382 | -3.980961797 | 3.465026268 | 3.305048098 | 3.583999394 | 3.404571467 | 3.392890169 | 3.342529637 |
| PANX1 | -0.1594885093 |  |  |  | 0.5536456997 | 0.6392251134 | -0.3041099959 | 1.23866698 | 0.8059350863 | 0.7807101721 | 0.3656948936 | -1.401068269 | -0.3028956843 | 0.5435326366 | 1.197463146 | 0.3055178006 | 0.3281287244 | 0.04212079307 | -0.0249682509 | -0.4546631461 | 3.473218899 | 3.60474069 | 2.899726735 | 3.490959015 | 3.152335349 | 4.008290432 | -3.075057982 | -2.007902196 | -2.389106391 | -2.394868307 | -3.164361611 | -2.951131687 |
| GRIN2B | 0.5626813648 | 1.562681365 | 2.562681365 | 3.562681365 | 0.1327193538 | -0.475706248 | 0.3161775323 | 0.1103786961 | -0.3825338583 | -0.4158956522 | -0.0684597386 | 0.033393204 | 0.211705045 | -0.0645835137 | -0.253611933 | 0.0469067534 | -0.1634097255 | -0.2330157823 | -0.1142688019 | -0.2243453415 | -2.279914642 | -2.109561028 | -2.973816459 | -2.382141844 | -2.536131363 | -2.338377183 | 4.871120984 | 4.759228341 | 4.195222528 | 4.444247676 | 5.260011704 | 4.587853044 |
| PLTP | 1.856216989 | 2.856216989 | 3.856216989 | 4.856216989 | -0.0501736089 | -0.2052267133 | 1.572632433 | -0.4193342528 | 0.6366634085 | -0.2325745606 | 0.0221161683 | 0.9987231356 | 0.741766274 | 0.0934687312 | -0.8128044699 | 0.0535430658 | -0.1929858415 | 0.2647952731 | 0.2878485533 | 0.2488318784 | 1.425388811 | 1.196634655 | 2.67420842 | 2.446626532 | 3.27017286 | 1.818205696 | -0.9787079223 | -1.081389591 | -1.343399018 | -1.002907668 | -0.9972330336 | -1.097727873 |
| NDUFB7 | 0.1370103494 |  | 2.21053064 | 6.114832813 | 0.0481475324 | -0.0612945958 | 0.192366532 | -0.0823694053 | 0.2654520882 | 0.19973622 | -0.1280731682 | -1.40923275 | 0.5167926351 | 0.0629572043 | 0.0752998246 | -0.1997110537 | -0.0885417357 | 0.2263092607 | 0.1718076618 | -0.4115543559 | 3.114027403 | 3.003345159 | 3.169234547 | 3.564853192 | 3.787255784 | 3.619816303 | -3.327349762 | -3.057340973 | -3.061237769 | -3.17170599 | -2.424888708 | -2.661669532 |
| TGM5 |  | 1.363157469 | 2.106705471 | 3.08827015 |  |  |  |  |  |  |  |  |  |  |  |  |  | -0.006654427568 | 0.1315194663 |  | -4.240360003 | -4.53105839 | -4.450451879 | -4.458347179 | -4.631731144 | -4.522260531 | 4.950211192 | 4.879050125 | 4.674581138 | 4.817953784 | 4.96350351 | 4.757824688 |
| SLC25A15 | -0.1019901575 | 1.610472773 | 1.931842566 | 3.187402963 | 0.0250998874 | -0.6749661349 | 0.0771059568 | -0.641983148 | -0.4010806406 | -0.1978454682 | -0.0265280478 | -0.6838290813 | 0.0718962773 | -0.3481258554 | -0.2312371203 | -0.0733934843 | -0.3349146091 | 0.004124146246 | -0.1273794971 | -0.3130935091 | -6.375546588 | -6.023020839 | -6.000888942 | -5.772522046 | -5.85623321 | -6.085697712 | 6.078863563 | 6.17827121 | 6.163671845 | 6.17827121 | 6.169606815 | 6.168625036 |
| SLC5A7 | -0.8100317089 | 0.9157243588 | 1.292506129 | 4.107136251 | -0.0390839515 | -0.2834698073 | -0.5440568807 | 0.4096357525 | 0.1372667529 | 0.2019098829 | -0.0722716154 | 1.691820668 | -0.2569341635 | -0.1177910712 | 0.2096946314 | -0.0352041738 | 0.0838961504 | -0.03624589985 | 0.1414424218 | 0.4551613285 | -3.945819423 | -3.86789945 | -3.873279587 | -4.083705782 | -4.067615501 | -4.031956662 | 2.961575157 | 3.677230226 | 3.532953084 | 3.300534462 | 2.754330007 | 3.157450547 |
| FLI1 | -0.600572171 |  |  |  | 0.238196994 | -0.852819866 | -0.3938406813 | -0.2131972383 | -0.1146690504 | 0.5840022538 | -0.1302482387 | -1.696832393 | -0.5400649576 | 0.099269331 | 1.086786484 | -0.3558153201 | -0.2606538967 | -0.1400845307 | -0.3652128056 | -1.118473484 | 3.631978267 | 3.706929703 | 3.203807558 | 3.693007404 | 4.068899936 | 4.550687064 | -2.521532172 | -2.534514821 | -2.121825852 | -2.722137714 | -3.104948463 | -3.101379804 |
| RASSF9 | 0.2472932881 | 2.317596392 | 2.538051324 | 3.724461411 | 0.27513417 | -0.326607698 | 0.0478468987 | 0.0265214574 | 0.5142702868 | 0.1246034283 | 0.0260922218 | -0.0295569874 | -0.0479256677 | 0.8648045265 | 0.0813183311 | 0.0037271684 | -0.0119610928 |  |  |  | -3.843281039 | -3.455723538 | -3.429452386 | -3.697748106 | -3.384460467 | -3.404047959 | 3.118349093 | 3.423223259 | 3.382890062 | 3.426577443 | 2.770284237 | 3.227903296 |
| OAS2 | 0.7065415375 | 2.992641511 |  |  | 0.185658553 | 2.840687391 | 0.6295573155 | 1.87451172 | 2.542742297 | 1.253463578 | 0.3317786501 | -1.263535559 | 0.9999241078 | 0.447265491 | -0.2605763824 | 0.4906616252 | 0.1430292402 | -0.2880762446 | -0.2162167298 | -0.989854466 | 3.736131034 | 3.909393007 | 4.793933315 | 0.8617705167 | 1.98806166 | 1.440491455 | -0.9401934708 | -1.342244153 | -1.267497206 | -1.014916733 | -0.7249671261 | -1.383666455 |
| RPL14 | -0.1823152391 | 1.033710191 | 1.991377918 |  | 0.0777797933 | -0.4052407811 | -0.5570949678 | -0.9162999885 | -0.4207371764 | -0.2675470372 | 0.2383238324 | -0.5348048101 | -0.0211838395 | 0.0348304925 | -0.0953488667 | 0.11990731 | -0.1537623467 | 0.01829224976 | 0.049236169 | -0.1404447649 | 3.037058832 | 2.587736661 | 2.827941573 | 4.10650467 | 4.556550758 | 3.069233649 | -4.042635448 | -4.314079902 | -4.33693979 | -4.411210581 | -4.612238952 | -4.093287792 |
| ACSL1 | 0.1263165483 |  |  |  | -0.0421050171 | -1.457609797 | -0.2813395236 | -2.588567933 | -0.5554661122 | -0.0170267788 | 0.4396807642 | -2.506677606 | -0.4413211765 | -0.593960336 | 0.0053516815 | 0.1122067211 | -0.9745873903 |  |  | 1.45916647 | 2.075565169 | 1.895029356 | 1.714856984 | 1.354771137 | 0.4111869072 | 0.5301738497 | -3.531684079 | -2.896320558 | -3.460320888 | -3.217716729 | -3.391072818 | -2.978563192 |
| DMD | -0.4127037207 | 0.5872962793 | 1.587296279 | 2.587296279 | 0.5049229073 |  | -0.3609828522 |  | -0.401380887 | 0.0179436811 | -0.2116240777 |  | -0.3570544871 | 0.1805562089 | 0.6268798413 | 0.1633838018 |  | 0.1135671179 | 0.1401006353 | 0.0646665589 | -2.009888573 | -1.747367411 | -1.740751304 | -1.819826997 | -1.9304419 | -1.882305372 | 1.156248224 | 1.593283502 | 1.59809842 | 1.602999593 | 1.154923342 | 1.610540041 |
| BAD | -0.093519318 | 0.906480682 | 1.906480682 | 2.906480682 | -0.0049487519 | -0.1039317189 | 0.303838563 | 0.2053277971 | 0.4818286954 | -0.1579349398 | 0.0509429765 | 0.1907499512 | 0.2310650996 | 0.1320850984 | -1.074252048 | -0.0136720595 | -0.2640959376 | -0.0278593185 | 0.0781184536 | 0.3945315425 | 2.434658383 | 2.449061788 | 2.695915127 | 2.796493712 | 3.12063426 | 2.627067257 | -1.356682662 | -1.71890487 | -1.559301252 | -1.712382369 | -0.980671782 | -1.129470603 |
| PAK5 |  |  | 3.193098368 | 2.061793958 |  |  |  |  |  |  |  |  |  |  |  |  |  |  |  | -0.0019822815 | -2.879850217 | -2.589065733 | -3.897928053 | -3.125855278 | -3.832458241 | -3.665480693 | 2.218694513 | 2.708373071 | 2.487671265 | 2.454270944 | 2.242370983 | 2.391984302 |
| MAP4K2 | -0.6014692737 | 1.240798018 |  |  | -0.2165884091 | -0.2219028321 | -1.020961923 | -0.4868629499 | -1.110787277 | 0.2105508966 | -0.1727948353 | -2.613894379 | -1.098106234 | -0.2102918825 | 1.447403545 | -0.092110967 | -0.2985712101 | -0.1263913642 | -0.1387217754 |  | 1.310641742 | 1.970503641 | 2.131522178 | 0.07418838702 | 0.4001501813 | 0.5930723635 | -1.187331654 | -0.9411578849 | -1.192982041 | -1.634749778 | -1.636526131 | -1.830781249 |
| MAP4K4 | -0.2446033398 | 1.618550471 | 1.398530726 | 3.240798018 | -0.0007719577 | 0.3312281269 | -0.4988299594 | 0.0349503871 | -0.1155558812 | 0.2012984636 | 0.2474781239 | -0.7653670302 | -0.3147051128 | -0.0638209825 | 0.4715980639 | 0.010801924 | 0.2891103329 | 0.1179162593 | 0.1605309226 | -0.5136308651 | -2.325953877 | -2.547753741 | -3.21045136 | -2.111866817 | -1.907813769 | -0.9142775537 | 2.055836207 | 1.608454511 | 1.819032223 | 1.938314922 | 2.04691474 | 1.926394075 |
| RPS5 | -0.182771794 |  | 0.6641221596 |  | 0.004964948 | -0.5432018987 | 0.0879166234 | -1.045388753 | -0.1488889857 | -0.3927406551 | 0.041689955 | -2.041426268 | -0.1781801685 | -0.0933554008 | -0.6774193764 | 0.0533951218 | 0.7000095474 | -0.1129431597 | -0.0712983974 | -0.4609240284 | 2.88783618 | 2.651355225 | 2.964179099 | 3.634227888 | 3.566839403 | 2.564251973 | -2.929250487 | -3.151205213 | -3.047346402 | -3.029137728 | -2.45384479 | -2.863341306 |
| ELOVL5 | -0.3526348785 | 1.16205575 | 2.18392708 | 5.542961998 | 0.007712523 | -0.6440135137 | 0.2559969687 | -0.146720717 | -0.4396992208 | 0.3763171985 | -0.3003838589 | 1.447115362 | -0.6388063324 | -0.3579616981 | 0.6657842423 | -0.4336563923 | 0.0028802534 |  |  | -0.1075537258 | 1.526651992 | 0.9388083695 | 1.208913788 | 2.371622175 | 2.305836484 | 0.9263576241 | -2.230042701 | -2.66907179 | -2.398886499 | -2.118256374 | -1.968996708 | -2.315783423 |
| PEF1 | -0.510549924 |  |  |  | 0.0531432847 | 0.5446704927 | -0.0284247733 | 0.3817040579 | 1.009176449 | 0.7226499723 | 0.3395453664 | -2.00971784 | -0.0155428406 | 0.7080100641 | 0.8262923059 | -0.0049495741 | 0.1474751168 |  |  |  | 3.616423735 | 3.65083103 | 3.040779664 | 4.091641963 | 3.965117651 | 4.401970103 | -2.507330689 | -2.83689794 | -2.500794337 | -3.099774137 | -2.980112919 | -3.112130355 |
| APOD | -0.095088414 | 0.904911586 | 1.904911586 | 2.904911586 | -0.0164072933 | 0.6897648633 | -0.2262866373 | 0.4381701762 | 0.310892172 | 0.3926467806 | 0.218529754 | -0.1937100513 | -0.2981598215 | 0.0344396306 | 0.2544771904 | 0.1821854364 | 0.5123344435 | 0.02365523827 | -0.0456905024 | -0.4319827775 | -2.824682726 | -2.661150603 | -2.791139092 | -2.638816497 | -2.518721085 | -2.519886197 | 3.373552915 | 3.452038645 | 3.642644685 | 3.213186205 | 3.441926515 | 3.391673212 |
| PSMB1 | 1.080054732 |  |  |  | -0.1033315436 | 0.7235170736 | 1.096349954 | 0.3501417571 | -0.1673366755 | -0.3399003797 | -0.1105682385 | 0.0679819505 | 0.7440788597 | 0.0331679869 | -0.3716025418 | 0.1042124542 | 0.7265024672 | -0.2070203727 | 0.3008751229 | -0.0242412057 | 2.584634109 | 2.454782327 | 2.48947981 | 3.10506534 | 3.1164181 | 2.819219909 | -3.341787072 | -2.790429899 | -3.573493575 | -3.669920446 | -2.828310542 | -3.297647124 |
| ALB | 0.1181049901 | 1.11810499 | 2.11810499 | 3.11810499 | 0.1206807841 | 0.2514121589 | 0.4977246498 | 0.2124013618 | 0.3119724944 | 0.7285680301 | 0.1804679213 | -0.090244529 | 0.5337301256 | 0.2156302264 | 0.8476344333 | 0.1327730952 | 0.7739865357 | 0.4107009523 | 0.6531235844 |  | -2.216615689 | -2.662895864 | -2.447365191 | 0.7901117144 | -2.686737941 | -2.412993156 | 3.53099697 | 1.926649817 | 1.721797795 | 1.967227998 | 2.645474224 | 2.669151213 |
| ABCB4 | -0.1104778837 |  |  | 3.683447713 | 0.2954406523 | -0.4114999119 | -0.0371535111 | -0.1984934382 | 0.1251901419 | 0.6053994047 | 0.4475059554 | -2.17692601 | -0.1411916503 | 0.1364015946 | 0.9968988409 | 0.1812907685 | -0.0794769859 | -0.01601018268 | 0.3339145929 | 0.6125823291 | 2.599887029 | 3.008474532 | 2.737275583 | 2.873873764 | 1.779559108 | 3.097952252 | -2.117477674 | -2.155000448 | -2.396230956 | -2.251475585 | -2.323827228 | -1.171123901 |
| JAK1 | -0.6327011898 |  |  |  | 0.1698414235 | 0.1775087225 | -0.6958544989 | 0.4314345625 | -0.1583258108 | 0.6252646648 | 0.0671719471 | 1.397673174 | -0.614595549 | -0.1852352982 | 1.039861821 | 0.1043771509 | 0.117676465 | -0.1209637163 | -0.0857762163 | -0.95686812 | 3.732308676 | 3.764407902 | 3.583382758 | 3.548409138 | 3.75897265 | 4.473265386 | -3.160976856 | -3.504525926 | -3.225370738 | -3.263607531 | -3.086450271 | -3.196128393 |
| CD1D | -1.205394677 | -0.2053946773 | 0.7946053227 | 1.794605323 | 0.8034851784 | 0.0529221595 | -1.118931773 | -0.3560101496 | -0.2747108813 | 1.222027213 | 0.2619142188 | -0.2689948888 | -0.6652988658 | 0.324210947 | 2.770658685 | 0.2048949997 | 0.717456313 | -0.1913303545 | -0.2398825199 | -1.488948952 | 0.3197835883 | -0.02727830907 | 0.386585221 | 2.294488746 | 1.199551417 | 0.7773374009 | -1.369671349 | -1.584021834 | -1.488983283 | -1.748795751 | -1.476115477 | -1.844556262 |
| TPI1 | 0.2690591323 |  |  |  | -0.0420693986 | -0.4694319759 | 0.6129634567 | 0.0857747451 | 0.9379326612 | -0.62406342 | 0.4379883345 | -0.1632282748 | 1.195851226 | 0.5384528269 | -1.53336738 | 0.061235637 | -0.6469841692 | 0.1954292961 | 0.4525448348 |  | 2.879334478 | 2.998367109 | 2.58405633 | 3.008982673 | 3.12225206 | 3.406163594 | -1.25827564 | -1.660165277 | -1.561594875 | -1.512184124 | -1.401268152 | -1.453835934 |
| PRKCD | -0.5790970219 | 0.4209029781 | 1.420902978 | 2.420902978 | -0.0020679953 | 0.337574864 | -0.5081426863 | 0.1370081804 | 0.3608330703 | 0.6615456908 | -0.1312317575 | -0.9699416385 | -0.2794508092 | 0.0996432835 | 0.6152623356 | -0.0304655393 | 0.6853446637 | -0.01308598881 | -0.1096166957 | -0.2726397635 | 2.463495203 | 2.57536751 | 2.430329759 | 1.851364442 | 2.252182715 | 2.877425866 | -1.994362232 | -1.568793644 | -1.710773976 | -1.676040659 | -3.173766937 | -2.169290883 |
| MDH2 | 1.066105345 | 2.389175184 |  |  | 0.0615943986 | 0.2082275732 | 0.8103347982 | -0.7207145582 | -0.1085593547 | -0.2705727877 | 0.0684896387 | -0.4571569549 | 0.3174659979 | -0.2295798751 | -0.6523138915 | 0.0132586526 | 0.1817869744 | 0.04576431835 | 0.1398774688 | -0.2292070766 | 3.11034503 | 3.135008037 | 3.330900706 | 3.329367961 | 3.633079621 | 3.402933599 | -3.594525025 | -3.56696758 | -3.703868537 | -3.556754558 | -3.365102424 | -3.426155213 |
| PAH | -0.2638417353 | 0.7361582647 | 1.736158265 | 2.736158265 | 0.0594898754 | -0.3617156562 | -1.376393738 | -1.476848427 | 0.1826069509 | 1.020814692 | -0.0277961264 | -0.884805604 | -0.8774389425 | -0.1466706619 | 1.276549349 | -0.0843277107 | -0.0335835173 | 0.1369566251 | 0.1754594589 | -0.4667018932 | -0.6870190406 | -1.2508137 | -1.232366116 | 1.188746374 | -0.9642468804 | -0.615624066 | 1.768451021 | 0.6255471958 | 3.233945137 | 2.957345324 | 2.118594238 | 4.412863212 |
| HINT1 | 0.7090720207 |  |  |  | 0.0003874868 | -0.033280903 | 0.3423619467 | -1.152777608 | 0.0549903209 | -0.2526692449 | 0.0922170989 | -1.497017458 | 0.3679092825 | 0.2404447977 | -0.4627933082 | -0.0198083771 | 0.6684545364 | -0.01694410274 | -0.0026771263 | 0.0525440781 | 2.869303656 | 2.536929939 | 3.386301733 | 3.261375066 | 3.266256715 | 2.631481319 | -3.665378162 | -3.468303823 | -3.744863428 | -3.641551845 | -3.305238673 | -3.593321052 |
| MMP27 |  |  | 2.502800229 | 3.691322185 |  |  |  |  |  |  |  |  |  |  |  |  |  | -0.2092057966 | -0.1483938356 |  | -4.235722911 | -4.258105983 | -4.266909817 | -4.129880259 | -3.924443822 | -3.976806321 | 3.993233341 | 4.260453364 | 3.763347553 | 4.014913194 | 3.932554759 | 4.119942936 |
| KCNQ2 | -0.8377922559 | 0.9724531467 |  |  | 0.3168129566 | 0.0796973264 | -0.4370095107 | -0.0012615032 | 0.0848733479 | 0.8079774406 | 0.0040968758 | 3.701989759 | -0.1782681765 | -0.1481139209 | 1.025175042 | 0.2057193534 | 0.4536627109 | 0.04724308959 | 0.1434213366 | -0.1385864644 | -1.083990422 | -1.330949016 | -1.359833804 | -1.428895831 | -1.469639919 | -1.673238517 | 1.870409233 | 2.12501125 | 1.798164688 | 1.84963082 | 2.307885165 | 1.974788117 |
| NDUFS6 | -0.0096485681 | 1.221255784 | 0.945911448 | 3.031032811 | -0.1382104884 |  | 0.1205912251 |  | 0.2182084403 | 0.1552797153 | -0.1142295756 |  | -0.0464684263 | 0.3330222946 | 0.2284412383 | 0.0060743489 |  | 0.1231720499 | 0.2524851213 | -0.3540697217 | 2.671840055 | 2.620150664 | 2.59777734 | 3.395098631 | 3.372948127 | 2.933362721 | -3.094607774 | -3.129001713 | -3.056361995 | -2.928928932 | -2.692371533 | -2.940218958 |
| NDUFA5 | 0.5483766782 | 0.9299429918 | 1.584142273 | 4.900408242 | 0.017190476 | -0.2782198747 | 0.5653562626 | -0.198802733 | -0.1366437604 | -0.5438671808 | -0.327730605 | 0.3230959354 | 0.5342902633 | 0.1704510603 | -0.6686593819 | -0.1302449054 | 0.209034488 | -0.1435724667 | -0.0221280231 | -0.5041470444 | 1.857990603 | 1.618828909 | 2.09414144 | 3.033982224 | 2.776768082 | 1.857144102 | -3.128004061 | -2.496235715 | -2.940053471 | -2.579759289 | -2.68973568 | -2.480580468 |
| NAGPA | -0.1650415169 | 0.8349584831 | 1.834958483 | 2.834958483 | -0.0635070968 | 0.3210081869 | -0.0845917774 | -0.2659238621 | 0.5486850841 | 0.3215973876 | 0.1036376672 | -0.4462183675 | 0.0430515242 | 0.3883654052 | 0.3635369949 | -0.029017643 | -0.0342679813 |  |  | 0.0475410186 | -2.516312422 | -2.544764215 | -2.247580163 | -2.3067246 | -1.796977097 | -2.13357751 | 2.836953031 | 2.796211553 | 3.111182964 | 2.900316484 | 3.040719906 | 2.922511294 |
| PORCN | 0.3058706597 | 1.30587066 | 2.30587066 | 3.30587066 | 0.1257983685 | 0.409516426 | 0.4343818205 | -0.0114808789 | 0.5266877079 | 0.0469790683 | -0.0161592812 | -0.080931821 | 0.261259202 | 0.1295019733 | -0.3490321818 | -0.1186727663 | -0.0184755496 |  |  | -0.2307462143 | -1.511979489 | -0.9589598484 | -1.339956155 | -1.418946313 | -1.241876145 | -1.365876574 | 1.333799217 | 1.109633084 | 1.130403551 | 1.144707853 | 1.52092897 | 1.123898967 |
| MALT1 | -0.7270127449 |  |  |  | 0.5110345373 | -1.360991267 | -0.8931963019 | -0.8037421101 | -0.5041412272 | -0.0554521209 | -0.4224423123 | -0.2466004132 | 0.1189564733 | 0.0883504352 | 0.1750605841 | -0.0143143906 | 0.1152302875 | -0.02162375948 | 0.0990808466 | -1.172038502 | -3.877444885 | -4.10134554 | -4.241766145 | -3.894980642 | -3.724170375 | -4.170601223 | 2.96105048 | 2.524892466 | 2.155139194 | 2.293278263 | 2.837671447 | 2.597892312 |
| LPAR2 | -0.7551014887 | 1.516237926 |  |  | 0.5080899232 | -0.6282606167 | -0.3711970061 | -0.5972211221 | 0.413511674 | -0.4031258209 | 0.0769677495 | -1.465441428 | 0.5114699712 | 0.4224284405 | -0.302652934 | 0.2316585585 | -0.5961263022 |  |  |  | -2.063269223 | -1.941812401 | -1.894671873 | -1.778579724 | -1.836631627 | -1.868411861 | 1.418426634 | 1.837027815 | 2.09925922 | 2.008385718 | 1.502367185 | 1.715607296 |
| CDKN1A | 0.3105122811 | 1.310512281 | 2.310512281 | 3.310512281 | -0.0633979843 | 1.720771844 | 0.3894492884 | 0.1896534247 | 2.500364586 | 1.407581028 | 1.344660957 | 0.9679111399 | 0.3867908572 | 1.203256134 | 0.3572833218 | 0.4400810066 | 0.3864971998 | 0.4831826763 | 0.2850447511 | 0.6859652326 | 3.050724641 | 3.339287911 | 5.018648994 | 2.219767672 | 4.151354071 | 3.396472034 | -0.02107087072 | 0.02545981076 | -0.006539147419 | -0.05830364618 | -0.1396302121 | -0.02538760502 |
| PLA2G7 | -0.3299314496 | 1.396679806 | 2.033926213 |  | 0.0292830313 | 3.927868884 | -0.1860647923 | 1.08735326 | 2.072562716 | 1.621113319 | 0.0087442432 | -0.6380598401 | -0.5883410968 | -0.0134563501 | 0.4794738618 | -0.0142081808 | 2.166080836 | 0.04785695664 | 0.0746057736 | 1.911772024 | 2.510397905 | 3.062846369 | 3.194855273 | 1.379682278 | 1.534479688 | 2.618719568 | -0.1975745689 | -0.5031576422 | -0.767433798 | -0.8388482111 | 0.3372225204 | -0.1423606824 |
| ITK | 0.0222128735 | 1.022212874 | 2.022212874 | 3.022212874 | 0.2212599955 | -0.7589307759 | -0.1886526927 | -0.2598352345 | 0.179166101 | 0.4174290654 | 0.1708738692 | -1.578017006 | -0.2985398938 | 0.4260277604 | 1.308797591 | 0.6333968941 | 0.1888991952 | 0.1219624019 | -0.049416984 | -1.706612521 | -2.199724568 | -2.505930218 | -3.958798528 | -1.908227405 | -3.292016611 | -3.310762633 | -0.6484743413 | 0.03601964263 | -0.1183421858 | 0.160026567 | -1.742890613 | -0.9953796633 |
| IL1RN | 0.1328429043 | 1.132842904 | 2.132842904 | 3.132842904 | 0.0513642717 | 3.724403777 | 0.297960581 | -0.3922879667 | 3.263578865 | 2.999798955 | -0.2776109861 | -1.355650723 | -0.4669853727 | -0.2178665071 | 1.207123803 | -0.4773216812 | 1.051191067 | -0.180554676 | -0.3292746706 | 0.4074689729 | 1.831187855 | 1.668927919 | 1.803433309 | 0.8441519028 | 0.9814256294 | 1.635330585 | -0.6536302768 | -0.6716671049 | -0.4812421515 | -0.6803063153 | -0.2425675456 | -0.6581996172 |
| SIRT2 | 0.4164823409 | 0.0279346941 |  |  | 0.2007910956 | -0.1121374124 | 0.4368853196 | -0.3884599982 | 0.0519845485 | 0.0313304711 | 0.1737638447 | -1.711972729 | -0.1412975236 | 0.1206375322 | -0.1468873745 | 0.057707487 | -0.049179604 | 0.02492867226 | -0.1203105481 | -0.1870159611 | 2.267453309 | 2.41159416 | 2.391388521 | 1.972589159 | 2.25109754 | 2.322406848 | -2.6258557 | -2.089305727 | -2.604501179 | -2.240244232 | -3.143532281 | -2.818043555 |
| STK33 | -0.959950577 |  | 2.713629161 |  | -0.0798221027 | 0.1690718614 | -0.564904855 | -0.2504921311 | 0.1654564074 | -0.0787563648 | -0.0671751213 | -0.1837574824 | -0.240629509 | -0.0608761976 | -0.0780487251 | -0.0443271592 | 0.0207587613 | 0.09714142659 | 0.0931360076 |  | -1.758804765 | -2.242126085 | -1.973717461 | -2.050122221 | -2.051106232 | -2.175185108 | 2.480313492 | 1.707958438 | 1.287137068 | 1.342933071 | 2.488977886 | 1.953541093 |
| CLIC1 | -0.2406671274 |  |  |  | -0.0179976035 | 1.065816475 | -0.1327433963 | 0.1532807263 | 0.4678252852 | 0.9705113429 | 0.0096630123 | 0.4291984101 | -0.5534636813 | -0.0591249697 | 0.765410607 | -0.0132475492 | 0.8140725438 | 0.273166852 | 0.1228731067 | -0.0107140063 | 3.584638282 | 3.38545928 | 3.726036589 | 3.516437837 | 3.660560078 | 3.387175734 | -3.075378808 | -3.501399789 | -3.45164037 | -3.516277766 | -3.32663742 | -3.399784883 |
| CACNB2 |  | 1.217626788 |  |  |  |  |  |  |  |  |  |  |  |  |  |  |  | 0.1827264958 | 0.2340295627 | -0.1268800547 | -2.564451603 | -2.763591121 | -2.938762194 | -3.060355878 | -3.23878095 | -3.109818637 | 4.22422266 | 3.515446547 | 3.573855466 | 4.090000413 | 4.905166293 | 4.349571957 |
| C3 | 0.9412854254 | 0.8758413236 |  |  | 0.0337404696 | 1.23786755 | 0.5915009674 | -0.3134059759 | 1.746813148 | 0.524606203 | 0.1768348782 | -0.1300657993 | 0.6710748271 | -0.1351280335 | -1.165408559 | 0.1079235189 | -0.1676722419 | 0.2437453519 | 0.7039306097 | 0.6168290707 | 1.343715282 | 1.329308461 | 2.287461494 | 3.530924946 | 3.670553253 | 2.098206308 | -1.144395299 | -0.8727175326 | -0.9740163846 | -1.064522757 | -1.267483812 | -1.098462636 |
| SERPINF1 | 2.505446004 |  |  |  | 0.1909112939 | 0.2171225728 | 2.192511972 | -0.8109419286 | 0.7772545025 | 0.3705947625 | 0.4390683125 | -0.8019128218 | 1.771198063 | 0.4715547744 | 0.427979251 | 0.1950454714 | -0.1344984116 | 0.06654147682 | 0.2858699273 | -0.690251839 | -1.177679055 | -1.241147817 | -0.805269162 | -0.6470643469 | -1.095276676 | -1.169220019 | 1.595555749 | 1.742267711 | 1.670075581 | 1.805789983 | 1.744417354 | 1.659750964 |
| PSME1 | -0.5086912584 |  |  |  | 0.1892111539 | 0.2331507474 | 0.0982904316 | 0.3303246278 | 0.332222168 | 0.1373087682 | -0.0751843213 | -1.210744485 | 0.7347862622 | 0.3720807686 | 0.4858832649 | -0.067076326 | 0.1802055274 | -0.03361524455 | -0.0098556768 |  | 2.633764724 | 2.430954501 | 2.626936805 | 2.653189832 | 2.83246874 | 2.67384001 | -2.639271117 | -2.912778326 | -2.707808244 | -2.57185964 | -2.933723001 | -2.880970627 |
| ARPC2 | 0.0201482761 |  |  |  | -0.0468682898 | 0.4989910587 | -0.0320268694 | 0.6438781727 | 0.6658946066 | 0.6481953744 | 0.0814710225 | -1.468019076 | -0.0292490198 | 0.0942142458 | 0.2572570816 | 0.0305736846 | 0.1377989164 | 0.05359288678 | -0.0025556066 | -0.1662441904 | 5.757677535 | 5.39320439 | 5.589440333 | 5.911776124 | 6.587188539 | 6.834737948 | -4.171883039 | -4.381730647 | -4.553860023 | -4.361281219 | -4.44408943 | -4.149737246 |
| AKT2 | -0.166794051 | 0.4626461559 |  |  | 0.0351263164 | 0.1166383636 | -0.5433498141 | -0.0348022138 | 0.775783859 | 0.9160876206 | 0.3326472791 | -1.701351609 | -0.6685496787 | 0.3173478876 | 0.9561739551 | -0.0849000997 | -0.1436400264 | 0.08729217475 | -0.0785314109 | -0.8566537008 | 3.622423739 | 3.478847592 | 3.269350117 | 3.459992878 | 3.28215837 | 4.037125441 | -2.936461789 | -2.937811417 | -3.022804053 | -2.383984255 | -3.018911985 | -2.755662568 |
| PFN1 | -0.401512903 |  |  |  | -0.0725315831 | -0.7335046256 | -0.1467312277 | -0.618030795 | 0.6622573826 | 0.5195021426 | -0.1520025743 | -1.896740902 | -0.2962056929 | 0.466416756 | 0.5108360468 | -0.1672319479 | -0.8607603686 | -0.10835373 | -0.045818939 | -0.1677694413 | 4.593496145 | 4.505330694 | 4.341871346 | 4.808329336 | 4.888099724 | 5.454721527 | -3.739127456 | -3.859887871 | -3.719376022 | -3.61406732 | -4.523754449 | -4.119327664 |
| CASP1 | 0.0502368413 |  |  |  | -0.0028293779 | 1.343711303 | -0.0006470964 | 0.606257206 | 0.6010132329 | 0.416241005 | -0.0122523919 | -1.379630191 | -0.2342174272 | -0.1080018296 | -0.1197793384 | 0.0710311166 | 0.7881584519 | -0.3185480888 | -0.4984933559 | 0.6809793672 | 1.671014778 | 1.216806565 | 1.95594794 | 1.372606783 | 1.878649919 | 0.764477377 | -1.560450866 | -1.218345759 | -1.576581094 | -1.628361465 | -1.005142605 | -1.641453986 |
| KCND3 | 0.3912389051 |  | 2.223925541 |  | 0.3819363187 | -0.196789698 | 0.1643686293 | -0.5117033026 | 0.2143468496 | 0.0959395117 | -0.023236052 | -0.3382191112 | -0.0266772646 | 0.1924579229 | -0.0235324596 | 0.101182246 | 0.1059108666 | 0.009412731589 | -0.0173569302 | 0.0265369141 | -3.282075945 | -2.912390658 | -3.482033246 | -3.325778053 | -3.562795526 | -3.34379105 | 2.571748876 | 2.983227304 | 2.990950571 | 3.065254883 | 2.06485616 | 2.729215579 |
| GLIPR1 |  |  |  |  |  |  |  |  |  |  |  |  |  |  |  |  |  | -0.2197502903 | -0.0643823896 | 1.483975324 | 3.004576619 | 2.788287393 | 2.910911124 | 2.73622819 | 3.105724825 | 3.593702568 | -2.080368017 | -1.777615232 | -1.834669795 | -1.992597003 | -1.636785908 | -1.827742411 |
| F7 | 1.307239982 |  | 0.3736425241 |  | -0.019593574 | 0.5218335088 | 0.1117175715 | 0.1888882199 | 0.3167294692 | 0.7649043058 | -0.0320258776 | 0.2654907713 | -0.9189507067 | 0.1221659375 | 0.9752950936 | -0.0408518876 | -0.011924766 | 0.1327632569 | 0.2044860761 | 0.2954026488 | -3.052049304 | -2.893974215 | -2.902157848 | -2.756527738 | -2.608648173 | -2.893508449 | 3.507041132 | 3.535616698 | 3.678105485 | 3.338319268 | 3.528717436 | 3.618480834 |
| CDC42 | -0.4826202755 |  | 2.163821095 |  | -0.00094753 | 0.7112744767 | -0.2172586099 | 0.0692247693 | 0.4758821679 | 0.5777871295 | 0.026834371 | -0.8111381489 | 0.2413404762 | 0.2636602184 | 0.5598364055 | 0.0535151842 | 0.6563147686 | 0.07076707509 | 0.1057185017 |  | 3.697672037 | 3.809146989 | 3.712146108 | 3.430015198 | 3.647830776 | 4.05465857 | -3.609246162 | -2.763007684 | -3.393960532 | -3.093178346 | -2.783822572 | -2.621110226 |
| NPR2 | 0.6058432536 | 1.291532627 |  |  | 0.1117774026 | 0.3695545051 | 0.0098053604 | -0.5126511616 | -0.0252416018 | -0.2081922516 | 0.3565321345 | 0.5745957354 | 0.5931935699 | 0.0997711108 | -0.6396763662 | 0.3975222488 | -0.3683949265 | 0.2139310957 | 0.1055305686 | -0.6483671561 | 1.456064229 | 1.662851975 | 1.715266491 | 1.584002305 | 1.476178977 | 1.3375134 | -1.628978861 | -1.884684503 | -1.295538243 | -1.895462968 | -2.331752422 | -2.009636775 |
| KCNJ9 | 0.1997966974 | 0.6662924029 |  | 3.158477241 | 0.3183377518 | -0.002343851 | 0.041983473 | 0.0444555916 | 0.3795185441 | 0.1560990073 | -0.1275399693 | -0.0244927904 | -0.0490197943 | 0.6151795941 | 0.531169827 | -0.0543966263 | 0.1905379886 | -0.007383023899 | -0.0766871246 | 0.0271363244 | -4.930200665 | -4.225067489 | -3.672299658 | -5.288707076 | -5.141249588 | -5.111751593 | 4.869260465 | 5.26425847 | 5.211747556 | 5.127399431 | 5.225339279 | 5.236088056 |
| CKB | 0.5086395137 | 1.508639514 | 2.508639514 | 3.508639514 | 0.2221782421 | 0.8664534752 | -0.1932431338 | 1.130026432 | 1.280045086 | 0.9164897603 | 0.1606380046 | -1.351021917 | -0.0164402944 | 0.2862203842 | 0.3988216104 | 0.2524955237 | 0.1881102621 | 0.1589392263 | 0.358754916 |  | -1.169472549 | -0.9877089169 | -0.0005931977354 | -0.8467630351 | 0.04067867805 | 0.08223077829 | 2.946164619 | 3.109882501 | 2.999949145 | 2.972350816 | 3.095002053 | 2.891050218 |
| S100A1 | -0.0627150729 |  |  |  | 0.0235802579 | 0.0900274753 | -0.0919578112 | 0.7629279401 | 0.2709966852 | 0.3246223719 | -0.0284436443 | -2.52120569 | -0.2142741493 | 0.6274573738 | 0.7144168732 | 0.0666633016 | -0.2066060471 | -0.1199391416 | 0.0338072786 | 0.0426418284 | 4.415422448 | 4.540298459 | 4.114316661 | 4.203699344 | 4.112294766 | 4.531961346 | -1.213087167 | -3.234243515 | -2.686333859 | -2.669373328 | -3.16706262 | -2.48289019 |
| PTDSS1 | -1.131852878 |  |  |  | 0.1414806877 | 0.111121775 | -0.4960690416 | 0.030849318 | -0.0535036365 | 0.1015741224 | -0.0168035658 | -0.6762091766 | -0.2413246622 | 0.2748544665 | 0.5770902141 | -0.1308160546 | -0.0593290012 | 0.2515247304 | 0.3184848481 | -0.170391497 | 2.277552412 | 2.601101412 | 2.251436474 | 2.929471652 | 3.166473908 | 3.185610162 | -1.830245903 | -1.965689965 | -1.794617262 | -2.153096115 | -1.807097388 | -1.782305758 |
| MTR | 1.152353758 | 2.152353758 | 3.152353758 | 4.152353758 | 0.3142705854 | 0.0673682403 | 0.5182972205 | -0.2514571981 | -0.2219166548 | 0.1013335339 | 0.0302925991 | -0.3984829088 | 0.2714675743 | 0.1640772494 | 0.4212097511 | 0.1304996291 | 0.5263809638 | -0.1054242234 | 0.1901570976 | -0.5763845987 | 1.545510301 | 1.924357716 | 1.64872601 | 0.4400434873 | -0.03302747447 | 0.1039165429 | -1.57307024 | -1.453509782 | -1.623206112 | -1.698113356 | -1.702845585 | -1.774611042 |
| CD52 |  |  |  |  |  |  |  |  |  |  |  |  |  |  |  |  |  |  |  | 1.138030874 | 3.959711121 | 3.51982376 | 3.597784722 | 4.676470447 | 4.624616255 | 4.21766776 | -4.232508756 | -4.290072713 | -4.194563049 | -4.210703925 | -3.921618259 | -3.820032916 |
| CD2 | -1.718773686 |  |  |  | -0.031710018 | -1.315455601 | -1.982140258 | -0.474983425 | 0.1186999437 | 1.638926564 | -0.0311717845 | -3.436004602 | -2.014462971 | 0.1494742507 | 2.792668675 | 0.0738852915 | 0.2658066627 | -0.2788204976 | -0.540179925 | 0.6090695037 | -1.705937065 | -3.057831201 | -3.042552892 | -0.530603269 | -0.7713948721 | -2.253034281 | -0.7672657872 | -0.3550422925 | -0.342495097 | -0.6099277676 | -1.352141945 | -0.9271716187 |
| CEBPB |  |  |  |  |  |  |  |  |  |  |  |  |  |  |  |  |  | 0.09803238502 | -0.1012365763 | 1.221232257 | 1.788711251 | 1.914159032 | 2.821008147 | 1.079876244 | 2.233521932 | 1.375899928 | -1.891721981 | -0.9754555923 | -1.541281504 | -1.213491955 | -2.661413885 | -1.724562978 |
| HCAR2 |  | 3.594402714 |  |  |  |  |  |  |  |  |  |  |  |  |  |  |  |  |  |  | -1.548235077 | -2.534271186 | -1.909133619 | -2.147699944 | -1.361629155 | -1.361819751 | 1.318908728 | 2.517600482 | 2.072391778 | 2.636746928 | 0.5149359449 | 1.513248806 |
| LDHA |  |  |  |  |  |  |  |  |  |  |  |  |  |  |  |  |  | 0.2914990241 | 0.2458325391 |  | 3.278792338 | 3.275475805 | 3.558712327 | 3.637575179 | 4.157814106 | 3.728815218 | -2.498189001 | -2.65768577 | -2.759989199 | -2.967842702 | -2.324889982 | -2.682953168 |
| SLC20A1 | -0.1121010162 |  |  |  | -0.0093983257 | -0.3891103082 | -0.0719674842 | -0.2777630772 | -0.3234976142 | 0.2626038766 | -0.0859061866 | -0.6684825659 | -0.0470052931 | 0.0400633176 | 0.590453832 | -0.2031885263 | -0.0427930324 | -0.0245571302 | 0.0624898263 | 0.1129578119 | -2.646793241 | -2.509365817 | -2.641244064 | -2.334803672 | -2.283107361 | -2.09169432 | 2.882088166 | 2.360282265 | 2.568726632 | 2.653843376 | 2.672964583 | 2.348222853 |
| CTSD |  |  |  |  |  |  |  |  |  |  |  |  |  |  |  |  |  | 0.1653929782 | 0.0717770734 |  | 4.872627652 | 5.167601019 | 5.164634679 | 5.099639634 | 5.629670997 | 5.815733592 | -4.097017431 | -3.767289749 | -3.424806426 | -3.665891307 | -4.366231835 | -3.802300772 |
| SLC31A2 |  |  |  |  |  |  |  |  |  |  |  |  |  |  |  |  |  | 0.2394063048 | 0.2320191011 | -0.0893565488 | -2.984672304 | -3.04617738 | -2.165776458 | -2.762427425 | -2.198268471 | -2.663333505 | 2.059592551 | 2.468955588 | 2.304765861 | 2.372147456 | 1.32493588 | 1.945977386 |
| FCER1G |  |  |  |  |  |  |  |  |  |  |  |  |  |  |  |  |  | -0.07101037568 | -0.234453442 | 0.8307044459 | 4.823614037 | 4.522962923 | 4.258835008 | 5.407800883 | 5.250471175 | 5.89000766 | -2.692279214 | -2.936784095 | -3.160419786 | -3.38950983 | -2.386562988 | -2.744064219 |
| ANXA1 | -0.2380165452 |  |  |  | -0.0127589083 | 1.442127049 | 0.2472571824 | -0.9175470249 | 2.244689218 | 0.7089416579 | -0.0776767714 | -0.5477594064 | 0.5446767209 | -0.2090839326 | -1.414994878 | -0.1497548988 | -0.2164953111 | -0.1559947991 | -0.2639989206 | 0.108273036 | 1.68742649 | 1.629814549 | 1.996910173 | 1.568339767 | 2.262318545 | 1.953820167 | -1.376289112 | -1.513974059 | -1.925642623 | -0.6629002984 | -0.4490701463 | -0.3074893667 |
| PAK4 |  | 2.193098368 | 1.061793958 | 8.63297373 |  |  |  |  |  |  |  |  |  |  |  |  |  | -0.2331407114 | -0.3647755397 | 0.6901778466 | 2.979035798 | 3.395218698 | 3.582774665 | -0.1065016001 | 0.2331406303 | 0.07786057772 | -3.70536704 | -3.533214883 | -3.681699554 | -3.934125373 | -3.995202413 | -3.720892091 |
| GSK3A |  |  |  |  |  |  |  |  |  |  |  |  |  |  |  |  |  | 0.007276688504 | 0.0866158129 |  | 2.808078881 | 3.07643704 | 3.174328603 | 2.067880265 | 2.554564128 | 2.6181631 | -2.660433074 | -2.818503381 | -2.463365163 | -3.018890437 | -2.746643434 | -2.855634779 |
| EEF1A1 | 0.1746221902 |  |  |  | 0.3832021158 |  | 0.2462516655 |  | -0.3631697818 | -0.4131459559 | 0.0317967644 |  | 0.3718580474 | -0.2556276369 | -0.5188112576 | -0.0383548077 |  | 0.1295445733 | 0.097551515 |  | 2.539356031 | 2.338274209 | 2.487021777 | 3.519856412 | 3.727958866 | 3.064304645 | -3.437987838 | -4.442983578 | -3.325451947 | -3.886499149 | -5.017047449 | -4.304460994 |
| CFL1 |  |  |  |  |  |  |  |  |  |  |  |  |  |  |  |  |  | -0.02869266556 | 0.1246624004 | 0.3375065761 | 5.16961055 | 5.148198354 | 4.554342961 | 5.670125922 | 5.628195791 | 6.276405614 | -4.198027541 | -3.851138748 | -4.242606505 | -4.262609336 | -4.181667883 | -3.909909318 |
| CHP1 | -0.1082992786 |  |  |  | -0.0108849231 | -0.0440613882 | -0.799882655 | -0.7179436281 | 0.3259135914 | 0.6032400964 | 0.1967568428 | -0.6017834549 | -0.7878278072 | -0.1742693209 | 0.4202613621 | -0.0720672875 | -0.4677836751 |  |  |  | 3.310533301 | 3.273778704 | 3.320301183 | 2.828095934 | 2.702832936 | 3.181106343 | -4.020908948 | -4.04860816 | -4.08722747 | -3.497072689 | -3.670850894 | -4.084714279 |
| APOA2 | 0.4836347565 | 0.9853468745 |  |  | 0.0436953063 | 0.2818819849 | 0.0827992518 | 0.5430557382 | 0.1918278694 | -0.0814934718 | 0.1222206456 | 0.3260476078 | -0.2375365244 | 0.0239319948 | -0.2923322438 | 0.0442515483 | 0.3786160875 | 0.4125064758 | 0.5859074739 |  | -1.57041025 | -1.184561142 | -1.853880224 | 2.079712916 | -1.214505382 | -1.1178199 | 1.999025781 | 1.592360257 | 1.741204721 | 1.823266355 | 2.178754686 | 2.034389739 |
| STIP1 | 0.1099106845 |  |  |  | -0.0136318937 | -0.6701039572 | 0.3469694557 | 0.0964265497 | -1.099802674 | -0.3099641223 | 0.0116927938 | 0.001191958 | -0.3011850065 | -0.821208158 | -0.2794316527 | -0.3700736066 | -0.4894456107 | 0.06331227396 | 0.1977789381 | -0.2719207458 | 1.468829448 | 0.7335590511 | 1.862662938 | 1.114919033 | 1.235967373 | 1.009827428 | -1.919232767 | -1.41159347 | -1.669210475 | -1.683569284 | -1.186704281 | -1.812430404 |
| PTPN1 | -0.5425981377 |  |  |  | 0.0036665322 | 0.3348307669 | -0.9692684762 | 0.5057783269 | 1.16682115 | 1.5279311 | 0.2387085907 | -0.1394067514 | -0.9293237308 | 0.1911161969 | 1.42888697 | 0.0659048763 | -0.2431392135 | 0.1203255999 | 0.1815646379 | -0.5840927259 | 2.16833748 | 2.834197323 | 2.237506061 | 1.861093988 | 2.285619825 | 3.422049219 | -1.275982222 | -0.2183644308 | -0.7121990385 | -0.603673452 | -1.863629997 | -1.29683193 |
| FGF1 | 0.1081819628 |  |  |  | -0.0220691609 |  | 0.196460585 |  | 0.6112581038 | 0.4410914701 | -0.1094734899 |  | -0.0861069697 | 0.2402729493 | 0.2831279605 | -0.0663800957 |  | 0.193146731 | -0.1343464181 | 0.355747961 | -3.015227974 | -3.528522301 | -3.012311683 | -3.648951542 | -3.648231582 | -3.665770832 | 4.371380431 | 4.093989516 | 3.886859598 | 3.60442817 | 4.656716183 | 4.200192463 |
| BAG1 | -0.7718462416 |  |  |  | -0.0005216669 | 0.4042674096 | -0.1221577188 | 0.227989188 | -0.016352159 | -0.1876158372 | 0.1007954459 | 0.1606039689 | 0.0560442876 | -0.123975332 | -0.3655177714 | 0.0718045226 | 0.3121790649 | 0.02468728839 | -0.0831718001 | 0.3341632056 | 4.845631274 | 4.81630369 | 4.86794452 | 3.545243048 | 3.349630956 | 3.628715442 | -3.650391907 | -4.482484854 | -4.137276665 | -4.081944123 | -4.222639594 | -4.291784662 |
| AKR1B1 | 0.0851813756 |  |  |  | 0.116891968 | -0.188350274 | -0.0397307426 | -0.1453603297 | -0.6298947909 | 0.6100904889 | -0.0938554094 | -0.0603934308 | 0.1821245667 | -0.0663065888 | 1.343618619 | -0.1808105897 | -0.1662571697 | -0.3303701058 | -0.3702008971 | -0.3162665985 | 1.655799413 | 0.8806100978 | 1.400077898 | 2.125120494 | 2.142668421 | 1.320154534 | -3.131087267 | -2.733189131 | -2.959004862 | -3.11221296 | -2.920584953 | -2.80859138 |
| NFKB1 | -0.3795841946 |  |  |  | 0.0036435406 | 0.0726277077 | -0.3684332129 | -0.2224196693 | -0.356962439 | -0.2566100888 | -0.0526390833 | -0.0801886744 | -0.4052744941 | 0.0341503014 | 0.0506092196 | -0.0543321974 | 0.1781228157 | -0.4656922387 | -0.4341635786 | -0.0731256519 | -3.357532095 | -2.77730209 | -2.692986872 | -3.729105511 | -3.556043669 | -3.510698069 | 2.044945521 | 2.651177782 | 2.45003866 | 2.528605464 | 1.80425222 | 2.280635173 |
| CXCR4 | -0.8791958588 | 0.6638160464 | 1.816848739 |  | 0.3230243012 | -0.8644575377 | -0.9766845349 | -0.8230639258 | -0.2836421267 | 0.7146285259 | 0.1088761464 | 0.3004979896 | -0.8995040624 | -0.6529670656 | 0.9398689068 | 0.4800190416 | -0.3790262835 | -0.1620420339 | -0.4551341557 |  | 2.305773754 | 1.224912597 | 0.1066453332 | 1.156124429 | 1.358554341 | 1.383019513 | -2.183742907 | -2.240180131 | -2.53151514 | -2.363439336 | -2.135601899 | -2.236277025 |
| TUBB2A |  |  |  |  |  |  |  |  |  |  |  |  |  |  |  |  |  |  |  |  | 4.696611375 | 4.621307361 | 4.00979696 | 5.311142728 | 5.448747171 | 6.868808509 | -3.817462358 | -3.555798108 | -3.493004043 | -3.308926632 | -3.968124631 | -3.499366536 |
| CSNK1G2 | 1.581459541 |  |  |  | 0.059130514 | -0.6922812719 | 0.8618216732 | -0.2298578114 | -0.0076472585 | 0.4121879333 | 0.2533855724 | -1.741197446 | 0.708272731 | 0.3539851603 | 1.011049071 | -0.1218383831 | -0.4567586707 | -0.06473726706 | -0.0905505555 | -0.4182635984 | -1.926463666 | -1.495557985 | -1.931978085 | -1.628445267 | -1.304267067 | -1.368424144 | 0.7250117656 | 1.76722043 | 1.295846107 | 1.525982495 | -0.1159606446 | 0.4843583091 |
| DHPS |  |  |  |  |  |  |  |  |  |  |  |  |  |  |  |  |  | -0.02150488072 | 0.100212344 | -0.3695953668 | -2.509256823 | -2.986555302 | -2.470807059 | -2.21969049 | -2.409587555 | -2.608298147 | 2.341458061 | 2.417342755 | 2.592136314 | 2.459155704 | 2.549173756 | 2.244197978 |
| ME2 | -0.1795540244 | 2.472137732 |  |  | 0.0656301156 | -0.3468973004 | -1.205754876 | -0.451830749 | -0.0886251732 | 0.5198298146 | -0.1196568037 | -0.0632341922 | -0.8818177911 | 0.5505702984 | 1.151126065 | -0.0967483508 | 0.4617597649 | 0.2786423285 | 0.4777991244 | 0.0076711786 | -2.788748645 | -3.569236087 | -2.715323483 | -2.090573774 | -2.040023438 | -2.806711718 | 0.6656995307 | 1.433880632 | 1.215479195 | 1.71316174 | 0.2878178168 | 0.9976581857 |
| AIFM1 | -0.4520292059 |  |  |  | -0.0758061094 | -0.0723112103 | 0.2199681031 | 0.3088458872 | -0.4958939901 | -1.253124718 | -0.0693651874 | 1.356945448 | 1.369391791 | -0.1721725835 | -2.092582869 | 0.1852832176 | -0.0165677941 |  |  |  | 0.3335052607 | 0.4048813207 | 1.280654784 | 1.126979615 | 1.150714691 | 0.7931114843 | -1.348311075 | -1.069472308 | -0.8403910868 | -1.126083072 | -1.751147527 | -1.725303193 |
| CYB5R1 | 1.188576508 |  |  |  | 0.1082812477 | 0.6851788111 | 0.9057093977 | -0.5881983945 | 1.046850893 | 0.2973975172 | 0.2456935149 | 0.1686442457 | 0.9129727931 | 0.2827188673 | -0.189435075 | -0.0099975953 | 0.2674471225 |  |  |  | 1.49863013 | 2.093597303 | 2.633606569 | 0.9702997757 | 1.855769579 | 1.457101702 | -1.409200726 | -1.505536942 | -1.297590176 | -1.583090407 | 0.9666887412 | -1.760947155 |
| COX6B1 | 0.6380582919 |  |  |  | 0.003416271 | 0.4385719819 | -0.008963284 | 0.4563183787 | 0.8025659989 | 0.5065246186 | 0.1285644674 | -0.5254966499 | -0.1516145021 | 0.4857619002 | 0.234538469 | 0.0391281919 | -0.0256369061 |  |  |  | 3.712791707 | 3.357149904 | 3.184424019 | 3.933667099 | 4.005836655 | 4.223987155 | -2.601210497 | -3.423645627 | -2.286244766 | -2.446581414 | -3.256780215 | -2.903198609 |
| COX4I1 | -0.4945618287 |  |  |  | 0.006527575 | 0.125015977 | -0.310184985 | -0.9752815038 | 0.393457611 | 0.0870074447 | 0.2913301414 | -0.8658230343 | 0.1950383207 | 0.092589301 | -0.5587752206 | 0.1680490253 | 0.0452912743 | -0.08805179922 | -0.0844162098 | 0.2514924286 | 3.918371389 | 4.011087307 | 4.149790681 | 4.239243112 | 4.42309319 | 4.402147784 | -3.841488329 | -3.832120384 | -3.811408784 | -3.862620911 | -3.945378953 | -3.944388123 |
| UBB |  |  |  |  |  |  |  |  |  |  |  |  |  |  |  |  |  | -0.072265223 | -0.0710441589 | -0.0180295166 | 5.642284091 | 5.873423153 | 5.883862642 | 5.595098771 | 5.02874537 | 5.32321296 | -5.459453985 | -5.781040536 | -5.489122662 | -5.783514168 | -5.45239549 | -5.544043451 |
| LGALS2 | -0.5498500845 |  |  |  | 0.6999749999 | -0.3068253079 | -0.1353687752 | -1.121321718 | 1.031271484 | -0.7445813232 | 0.3495922351 | 0.1576894271 | 0.6316970456 | 0.7334946956 | -1.310502663 | 0.2991612421 | -0.5208215121 | -0.3271305836 | -0.4729640865 | -0.274686991 | -1.23555031 | -1.342156801 | -0.7467349311 | -1.0537654 | -1.066429945 | -1.126291299 | 1.01673259 | 1.237449435 | 0.9259753598 | 1.208643291 | 1.215447756 | 0.875527947 |
| ALDH2 |  |  |  |  |  |  |  |  |  |  |  |  |  |  |  |  |  | 0.005098950968 | -0.1058396085 | -0.5421713953 | 4.244787454 | 4.466404582 | 4.425197979 | 4.054535531 | 4.080793325 | 5.025910797 | -2.76116037 | -2.745876777 | -2.991195733 | -2.856736755 | -2.95379664 | -2.96242285 |
| VKORC1 |  |  |  |  |  |  |  |  |  |  |  |  |  |  |  |  |  |  |  | -0.0686807373 | 2.741533253 | 2.617360043 | 3.207435257 | 3.733921435 | 4.014225463 | 3.26551952 | -2.068754226 | -1.950394364 | -2.017782676 | -2.26684175 | -2.108562597 | -2.21869382 |
| HRH3 | 0.0788583745 | 0.23737903 |  |  | 0.3048737624 | -0.0793636428 | 0.5863383824 | 0.004353217 | 0.5528807212 | 0.186057653 | 0.0475094627 | 0.0803481356 | 0.7318281167 | 0.2752018626 | 0.1150671911 | 0.0602533356 | 0.0455748734 | 0.03974717297 | -0.0323800467 | -0.5743779223 | -4.475591128 | -3.603605177 | -3.311651648 | -3.891824056 | -4.118399086 | -3.868755369 | 2.926564659 | 3.820726374 | 3.741442088 | 3.938239714 | 2.798396625 | 3.669863005 |
| SLC31A1 |  |  |  |  |  |  |  |  |  |  |  |  |  |  |  |  |  | 0.1464387611 | 0.2572013651 |  | -2.557376238 | -2.668893625 | -1.777863213 | -1.047551511 | -1.339469382 | -1.930770676 | 1.65423391 | 1.234812286 | 1.567372165 | 1.63733927 | 1.120084926 | 1.506372164 |
| SLC10A1 | -0.2603256977 |  |  |  | -0.0520324166 | 0.1722285587 | -0.4706574878 | -0.0524773528 | 0.3673662407 | -0.1578258017 | -0.0524691922 | 0.6695891559 | -0.3521252132 | 0.3331435381 | -0.3994853652 | 0.0067311391 | 0.0928209372 | -0.2671666894 | -0.1979261558 | -0.1311978605 | 4.915237989 | 5.048134799 | 5.265847576 | 3.926557894 | 3.885619999 | 3.432986774 | -4.693018761 | -4.545465583 | -4.826717261 | -4.654752987 | -5.35347876 | -4.741500708 |
| SLCO1C1 | -0.7857889856 |  |  |  | -0.0531364339 | 0.0635491447 | 0.0969167119 | -0.0573022788 | 0.2375653615 | -0.1717903658 | -0.0081679862 | -0.5141947898 | 0.3523960516 | -0.1673846328 | -0.2598791291 | -0.0470737765 | 0.174791194 |  |  | -0.2131159287 | -1.899690626 | -1.815441227 | -1.891436389 | -1.660591721 | -1.964457151 | -1.798242294 | 3.133730899 | 3.247234875 | 2.837687245 | 3.081441292 | 3.932312447 | 2.873781284 |
| SLC1A5 | -1.372204883 | 2.611225973 | 1.989896017 | 3.608107535 | -0.0018305644 | -1.69993604 | -0.9125039709 | -0.7239551121 | -0.8741183791 | -0.7603883471 | -0.2137637319 | -0.7311397759 | -0.2598390257 | 0.0890889723 | -0.1807241599 | -0.3388977797 | -0.7479114126 | 0.1815217271 | 0.3291112244 | -0.372069489 | 0.6786792768 | 0.5380326127 | 1.058983617 | 0.0226724803 | 0.1659980256 | -0.3057545456 | -1.267713369 | -1.312144115 | -1.411220497 | -1.799118841 | -1.098972299 | -1.526850188 |
| NDUFS5 | 0.2212557837 | -0.054088552 | 2.031032811 | 3.275560172 | -0.0694593901 | 0.2065782553 | -0.1142868109 | 0.1990511628 | 0.3053837219 | -0.5015243637 | -0.0563633696 | 0.225507918 | 0.8110310942 | 0.1981124556 | -1.012863284 | -0.1260489218 | -0.1488831692 | 0.1381499169 | 0.1912777553 |  | -1.503175357 | -2.16260602 | -2.415543219 | -0.4834124646 | -0.8471881686 | -0.846778671 | 1.526610025 | 1.720838346 | 1.532418151 | 1.365462921 | 1.400830961 | 1.424988624 |
| SMS |  |  |  |  |  |  |  |  |  |  |  |  |  |  |  |  |  | -0.03754338455 | -0.0466500106 | -0.3926513442 | -2.549398914 | -3.626448324 | -3.429244002 | -2.61553258 | -2.674119716 | -2.773721426 | 2.187328333 | 1.997080791 | 2.032880209 | 1.91022061 | 2.182924905 | 1.828173564 |
| MAT2A |  |  |  |  |  |  |  |  |  |  |  |  |  |  |  |  |  | 0.8435057351 | 0.6368813948 | 0.0714818239 | 1.472090999 | 1.207576766 | 1.405650104 | 1.55406617 | 2.170746058 | 1.76166021 | -2.703687334 | -2.879745266 | -3.069532093 | -2.783675782 | -2.947898184 | -2.925692289 |
| HLA-DQB1 |  |  |  |  |  |  |  |  |  |  |  |  |  |  |  |  |  | -0.1832386985 | -0.3486599617 | 1.602685861 | 2.77152722 | 1.92287673 | 1.493675884 | 2.803110146 | 2.608620138 | 0.827056458 | -3.81287947 | -4.350393704 | -4.215692056 | -4.26900588 | -4.369985187 | -4.204351021 |
| COL1A2 | 1.559969284 | 1.726386561 |  |  | -0.0110130409 | -0.4601928063 | 1.556730414 | -1.012127587 | 0.2292962066 | -1.059158202 | 0.4307646036 | 0.3513656298 | 1.644837556 | 0.2612008324 | -2.108245562 | 0.2040782541 | -0.7583366806 | 0.1773358749 | 0.7596888381 |  | -0.06031204312 | 1.956104825 | 1.233628505 | 1.912080792 | 2.289470517 | 1.898776645 | -0.19519293 | -0.1367583106 | 0.04372498601 | 0.4603489022 | -0.7129743565 | 0.07898452781 |
| UCP2 | -0.6257557804 | 1.094346508 |  |  | 0.1660780025 | -0.3229676265 | -0.647144761 | -0.6041698577 | 0.0367599552 | 0.3878390624 | -0.0628042344 | -0.4685107452 | -0.3554977651 | -0.1501161504 | 0.5830540161 | -0.0099233663 | -0.5049214757 | -0.1643225053 | -0.2042269776 | 1.198055185 | 4.412376525 | 4.492772338 | 4.423338891 | 1.332386591 | 0.9240818699 | 0.8806612099 | -2.465185182 | -2.597462001 | -2.733719852 | -2.668417517 | -2.216828825 | -2.819462061 |
| SCD | 1.521461892 | 2.521461892 | 3.521461892 | 4.521461892 | 1.449647328 | 0.8168570328 | 0.8513255693 | 0.0520943572 | 0.6456131348 | 0.3328011249 | 1.777480916 | 2.951686001 | 1.072302219 | 0.7958944901 | 1.091679902 | 1.008824297 | 0.5373779245 | -0.05512650825 | 0.2522471097 | 0.0652597182 | 1.01375021 | 0.9192503741 | 0.04741867764 | 0.4850541559 | 0.772063293 | 0.157105861 | -0.8351697406 | -1.087319798 | -0.9530674132 | -0.8947814499 | -0.9812068375 | -0.4761160964 |
| POLD1 | 1.484517393 |  |  |  | -0.086163325 | -1.821097654 | 0.7644666761 | -0.3577632045 | -1.689104504 | -1.430850608 | -0.5366221655 | -1.061190975 | -0.0264193427 | -0.4871745513 | -1.752959244 | -0.191807839 | -0.4181490035 | 0.3001316249 | 0.3301116324 | -0.9630652153 | 0.4824860231 | -0.7018201844 | -0.9097925891 | 0.3027501152 | 0.4118190121 | -0.1346849974 | -1.313967249 | -1.587403086 | -1.416025831 | -1.570684989 | -1.397243228 | -1.220379091 |
| TFPI | 1.652080989 | 2.652080989 | 3.652080989 | 4.652080989 | 0.2811620441 | 0.9354162571 | 1.663688457 | 0.356227872 | 0.1688453218 | -0.3727709581 | 0.2980597317 | 0.7001196843 | 1.347544268 | 0.6577308207 | -0.6559461503 | 0.1665332608 | 0.3639554328 | 0.3834243673 | 0.7111893716 | -0.4062535682 | -0.9074982784 | -0.8468717421 | -0.3502444683 | -0.4077669511 | -0.1227441959 | -0.4196546072 | 1.229688384 | 1.341177341 | 1.249140174 | 1.592773184 | 1.595943528 | 1.110715332 |
| ZAP70 | -0.2266088903 |  |  |  | 0.0089095034 | -0.4796757446 | -0.565135033 | 0.0891919982 | 0.1717990427 | 0.8758242339 | -0.0239493061 | -2.924646382 | -0.5972899766 | 0.511193072 | 1.689629016 | -0.0310898035 | 0.341216827 | -0.499350047 | -0.4241744532 | -0.3570100182 | 1.14675987 | 0.819906115 | -1.09503362 | 1.852355698 | 0.5566705688 | 0.5845976432 | -1.759865683 | -1.947206234 | -1.747369916 | -1.972680477 | -1.847155581 | -1.864830068 |
| FYN | -0.4904664457 | 0.5095335543 | 1.509533554 | 2.509533554 | 0.0837262604 | 0.0858495936 | -0.5962418741 | 0.560213657 | 0.050211871 | 0.8641490239 | 0.0624119448 | 2.816250669 | -0.5475881416 | 0.3894840433 | 1.777235531 | -0.0034102494 | 0.4317683627 | -0.5521275943 | -0.358667064 | -0.4550247406 | -0.5786175816 | -0.392029676 | -1.761820144 | -0.8684732035 | -0.3870477328 | 0.8414913511 | 3.261744199 | 1.666055 | 1.811979446 | 1.677653278 | 2.369761089 | 2.288084016 |
| MT2A |  |  |  |  |  |  |  |  |  |  |  |  |  |  |  |  |  | 0.1081452168 | 0.1705069941 |  | -2.215724227 | -2.128865475 | -1.442648742 | -0.06941893157 | -1.217160178 | -1.479091065 | 3.661220278 | 2.838973173 | 2.625521843 | 2.678091059 | 3.394238823 | 2.986645607 |
| LUM | 0.6311066578 | 1.631106658 | 2.631106658 | 3.631106658 | 0.0122073035 | 0.0023015982 | 0.2048262743 | 0.0484829773 | -0.3135159969 | -0.2424464434 | 0.1032902967 | 0.6517508975 | 0.1640364516 | -0.671807899 | -0.6762336113 | -0.1472460538 | -0.4178522864 | 0.2178842983 | 0.2619403275 | -0.2945933456 | -1.352245465 | -0.4603386375 | -1.409225066 | -0.5487965727 | 0.3670553278 | -0.1729355069 | 2.847053614 | 2.828265592 | 2.368598103 | 2.692009423 | 3.341820582 | 2.554190613 |
| ADORA2A |  |  |  |  |  |  |  |  |  |  |  |  |  |  |  |  |  | -0.04946114257 | -0.2436229877 | -0.4295932694 | -2.278083961 | -2.066328017 | -2.159954681 | -1.140190637 | -1.172490863 | -0.8950588084 | 2.35677227 | 1.747485583 | 1.635831516 | 1.871417527 | 2.077794391 | 2.138711956 |
| TRAPPC4 | 1.061695791 | 2.589825227 |  | 3.670172943 | 0.0864203519 | 0.7681926601 | 0.4443153609 | 0.6246598649 | 0.6661666014 | 0.4551639381 | 0.0169373465 | -0.0058696611 | 0.3822860047 | 0.2884507962 | 0.4711812207 | 0.1118531553 | 0.2781178293 |  |  | -0.2772013075 | 1.332761486 | 1.04792182 | 0.7501373026 | 1.74886608 | 1.626660581 | 0.8561949913 | -1.230536479 | -1.221709424 | -0.9654563301 | -0.8656694954 | -2.448213367 | -1.480874925 |
| CYC1 | -0.1927913754 |  |  |  | -0.0170115809 | -0.7037794016 | 0.0022796189 | -0.3738289507 | 0.115150641 | 0.2259829271 | -0.063106132 | -0.7677279904 | -0.0583071062 | 0.4740010811 | 0.0455407485 | -0.0532660928 | -0.5800672507 | 0.1639761363 | 0.3180143326 | -0.3850472756 | 0.8904711573 | 1.084542093 | 1.46321802 | 1.769190096 | 1.732155897 | 1.55274448 | -2.092231745 | -2.516965581 | -1.881692482 | -2.367390044 | -2.618899139 | -2.593419881 |
| UQCRB | 0.7294410087 | 1.729441009 | 2.729441009 | 3.729441009 | -0.078713032 | -0.0988577225 | 0.661298152 | 0.0083429338 | -0.1695278189 | -0.2829968925 | -0.1302212137 | 1.425986745 | 0.3973685824 | 0.062620628 | -0.4743852743 | 0.1476003474 | -0.006308941 | -0.07772427597 | 0.010094165 |  | -1.068627432 | -0.7812601176 | -0.6244497048 | 1.21966271 | 1.315517645 | 1.094593821 | -0.9517804235 | -1.409181591 | -0.7822783725 | -0.823837203 | -1.734227866 | -1.295024395 |
| TACR2 | 0.3407954701 | 1.037602883 |  |  | -0.0142755904 | -0.0283017654 | -0.8282274225 | 0.1195805886 | -0.505488819 | 0.5732800479 | 0.0307922658 | -0.0087449759 | -1.076992935 | -0.2034128724 | 0.7973036614 | 0.0204265367 | 0.1017244323 | 0.2456658916 | 0.0518550807 | -0.3530660608 | 3.058068789 | 2.612815774 | 2.17622255 | 5.101275443 | 3.562948426 | 4.345873121 | -1.290026629 | -1.660027561 | -0.8125888963 | -0.2228483199 | -1.263866031 | -0.7704226188 |
| RTCB | 0.140908879 | 1.140908879 | 2.140908879 | 3.140908879 | -0.018816316 | -0.5961527785 | 0.5939078482 | -0.9686910334 | -0.7308705261 | -0.0733749775 | -0.1410535305 | -1.022110552 | 0.2256115697 | -0.0594197719 | 0.0042202104 | -0.0286076098 | 0.4351126501 |  |  |  | -0.7328211664 | -1.497978313 | -1.345616119 | -0.07749719681 | -0.1778199634 | -1.030475474 | -1.165973543 | -0.03406563157 | -1.127960546 | -1.346336669 | -0.77116395 | -1.693736398 |
| LYZ |  |  |  |  |  |  |  |  |  |  |  |  |  |  |  |  |  | -0.2728087352 | -0.7400834281 |  | 3.666884854 | 3.865775378 | 4.473128595 | 3.735057116 | 4.386815379 | 4.390293492 | -0.8362543731 | -3.286490576 | -2.523143839 | -2.469392282 | -1.853294378 | -1.979848573 |
| SLC2A6 | -0.7219534917 | 3.659357391 | 2.145454012 |  | -0.1145154914 | 2.32259055 | -0.5258155348 | 0.2588344381 | 2.803882282 | 1.388524034 | -0.305676339 | -0.7230615275 | 0.2875953358 | 0.4316499133 | 0.3780287031 | 0.1087635993 | 0.0563182012 | -0.1877384088 | -0.4651607546 | 0.5524337758 | 1.818515085 | 2.368397584 | 2.303215492 | 0.7560012755 | 1.644235736 | 1.560632195 | -0.6254749232 | -1.139540066 | -0.8541489912 | -0.8272252886 | -0.985855504 | -0.8324643351 |
| IDH3B | 0.3608688689 | 1.360868869 | 2.360868869 | 3.360868869 | 0.0309373095 | -0.3616955658 | 0.5609022327 | -0.5232498593 | -0.2514452671 | 0.2206204452 | 0.0871010265 | -1.245471108 | 0.1142516797 | 0.1065247686 | 0.1817376535 | -0.1172184929 | 0.319211545 | 0.07499895956 | 0.1934366003 | -0.3350161451 | -1.316228598 | -1.601016508 | -1.206491128 | -0.9558815342 | -0.9006010179 | -1.052065994 | -1.444071121 | -0.3516391703 | -0.6629145463 | -0.5360198595 | -2.216497091 | -1.444598932 |
| BDH1 | 0.4192748935 |  |  |  | 0.0712367898 | -0.8894677603 | 0.0106746969 | 0.001804907 | -0.7836300021 | -0.2662403434 | -0.1171751186 | -0.1855443375 | 0.0375845356 | -0.0810582349 | -0.1014629805 | -0.1218743298 | -0.183283607 |  |  |  | -1.409459981 | -1.344578727 | -1.735117027 | -0.07698812848 | -1.231047657 | -1.407266092 | 1.944880228 | 2.050498566 | 1.492653805 | 1.678136243 | 2.251442095 | 1.484363507 |
| GATB | -0.1924349833 | 0.8075650167 | 1.807565017 | 2.807565017 | 0.054010326 | -0.1915386762 | -0.1141087704 | -0.0564279594 | -0.5579458373 | -0.074682786 | 0.3789587174 | -0.5182049167 | -0.3767750298 | -0.0885760109 | 0.1781826721 | 0.2330122154 | -0.1001742722 |  |  |  | -1.384733982 | -1.655072399 | -1.398194107 | -0.8029438189 | -0.670275865 | -1.123585883 | 1.298540638 | 1.016704007 | 0.9747171566 | 1.155949365 | 1.417833658 | 1.46821626 |
| DNM2 | -0.0713102377 | 1.109829795 |  |  | -0.1594057729 | 0.1951776614 | 0.0998652392 | 0.1939148215 | 0.7879490915 | 1.023878645 | 0.4764200439 | -1.783457678 | -0.1671450075 | 0.380446026 | 1.078945463 | 0.1011388488 | 0.1328929755 | 0.07522472526 | -0.1122504731 | 0.6377608911 | -0.844600222 | -0.4984388468 | -0.2851643954 | -1.223837801 | -0.6444139315 | -0.3974394075 | 1.766460455 | 1.566792147 | 1.492224684 | 1.415001849 | 1.679076372 | 1.750795397 |
| TREM1 | -0.6736402255 |  |  |  | -0.1183862775 | 3.49742061 | -0.569228374 | 1.28171512 | 3.099726087 | 2.755492615 | -0.2070891549 | -0.663117373 | -0.6501000335 | 0.4132934944 | 1.369824876 | 0.2511605307 | 2.521642147 | 0.05226401223 | -0.3241267568 | 1.408232631 | 1.27138913 | 1.395920894 | 0.865816731 | 0.5161135437 | 1.381221235 | 1.390970993 | -0.3607239272 | -0.2266613464 | -0.310437445 | -0.1840615834 | -0.4067107355 | -0.6890170747 |
| SLC7A7 | 0.7272792295 | 2.196397512 | 2.632108809 | 3.055207768 | 0.0327619501 | 0.4650842909 | 0.3524670739 | 0.3556795286 | -0.4434799267 | 0.1700688707 | -0.0378952781 | 0.8827894851 | 0.0836966952 | -0.319588967 | -0.0083460134 | -0.0151956254 | -0.2529800379 | -0.4896664561 | -0.5522827215 |  | 1.075392276 | 1.154533308 | 1.532815933 | 1.392871192 | 2.223680916 | 1.292871321 | -0.5711890395 | -0.6190737207 | -0.763472248 | -0.3883516517 | -1.021093348 | -0.8269454454 |
| TLR2 | -0.6982334598 | 0.292340536 |  |  | 0.0406027268 | 2.136800614 | -0.7857490916 | 0.1053816513 | 2.503969161 | 2.652439454 | -0.2113790339 | 0.2621919257 | -1.092865939 | 0.0012327085 | 1.794267244 | 0.0642906241 | 0.7735604426 | 0.03067759365 | -0.0302437291 | 1.202057453 | 1.228094423 | 0.842886152 | 1.323702776 | 0.7182665006 | 1.497197856 | 1.112262434 | -0.1649028216 | -0.1572998567 | -0.1762009021 | -0.2127644293 | -0.09235111141 | -0.2150223423 |
| HPR | -1.099453814 | -0.0994538141 | 0.9005461859 | 1.900546186 | 0.0173084102 | 2.19679941 | -1.441651976 | 0.0714013891 | 2.439211241 | 2.38065271 | 0.324448802 | -2.02746599 | -1.085340828 | 0.3648768052 | 1.754496873 | 0.1460134326 | 1.443543804 | 0.1335513664 | 0.2196673748 |  |  |  |  |  |  |  |  |  |  |  |  |  |
| IL6R | 0.7510024519 | 1.751002452 | 2.751002452 | 3.751002452 | 0.3178129295 |  | 0.3762915511 |  | 0.5530072479 | 0.5947213458 | -0.0820159543 |  | 0.0044552854 | 0.1526373774 | 0.3373694858 | -0.1539585556 |  | -0.1033038444 | -0.1515495228 | -0.0091957679 | 0.2387821246 | 0.6445018415 | 1.631053465 | -0.3704561906 | 1.105000791 | -0.07451799011 | -1.548345783 | -1.225180557 | -0.8985137955 | -1.027853018 | -1.574375004 | -1.03946022 |
| SLC25A12 | 0.1874029634 | 1.969682325 | 4.029383587 |  | -0.0190787704 | -0.3826809611 | 0.4841406179 | 0.1759135839 | 0.3318242912 | 0.2426342507 | -0.0652626525 | 0.0021783051 | -0.1094155144 | 0.4514015899 | 0.4741751982 | -0.0694632076 | -0.1143099418 | 0.1846247325 | 0.5544659527 | -0.2097800951 | -0.8834445993 | -1.170207985 | -1.331936059 | -0.7467724942 | -0.4550392717 | 0.1697171688 | 1.805764239 | 1.356686839 | 1.755643868 | 1.737310409 | 1.93742733 | 1.572185899 |
| CCR9 | 2.220004726 | 1.439968151 | 0.3616152128 | 6.309188884 | 0.0122821201 | 0.2970392173 | 1.766751772 | 0.4867691212 | -0.0201390316 | -0.0795795341 | 0.3041994678 | 2.351465528 | 0.7768716055 | 0.3627669833 | -0.0145270713 | 0.3449671869 | 0.3201315681 | 0.1419441679 | 0.1393027665 | 0.4128139342 | -0.3774416004 | -0.9644415568 | -0.5956874821 | 0.2157617312 | -0.2810591091 | -0.5310696957 | -1.31532482 | -1.140125466 | -1.221662536 | -1.099438391 | -1.126915456 | -0.979933032 |
| CDK4 | 0.8653566383 |  | 1.884730535 | 3.976867794 | 0.0109510594 | -0.7143874586 | 0.7853228567 | -0.4800364954 | -0.2414944364 | -0.5943184469 | 0.03490287 | -0.1328579685 | 0.7433909897 | 0.4211294927 | -0.8496785236 | -0.1690515764 | -0.6126741745 | -0.01161691215 | 0.2254929753 | -0.0489475929 | -2.107489462 | -2.861932202 | -2.617178892 | -0.622140371 | -0.5500502201 | -1.387435985 | 1.204017242 | 1.055861913 | 1.100100955 | 0.9614243983 | 1.356461496 | 0.8520507956 |
| DDX39B | 0.0925009578 | 1.092500958 | 2.092500958 | 3.092500958 | 0.0995325007 | -0.545313113 | 0.3476456314 | -0.3682402664 | -0.6545237524 | -0.6430520604 | -0.0391976381 | -0.1664243121 | 0.1939539522 | 0.0579882787 | -0.5272712603 | -0.1226503785 | -0.2119826879 |  |  |  | 1.082411578 | 0.6059643154 | 0.005478453762 | 0.1626246778 | 0.01675657276 | 0.08888635407 | -1.793911116 | -1.545759486 | -1.457604946 | -1.545486044 | -1.338667044 | -1.420048402 |
| CDK8 | -0.2451355332 |  |  | 3.155163495 | 0.3545912935 | -0.1524033979 | 0.0562930976 | -0.3750974345 | -0.5849369753 | -0.9225809307 | 0.3158508909 | 0.1848215407 | 0.6379241541 | -0.2959522955 | -1.100352838 | 0.0482239703 | -0.0241102829 | 0.09420970671 | 0.1143656078 | 0.3072101609 | -1.432912935 | -1.226174843 | -0.620030066 | -1.105662234 | -0.9832873863 | -0.9686896007 | 1.924178167 | 0.9664721401 | 1.196350138 | 1.866849097 | 1.107814727 | 1.906579683 |
| IGFBP6 | 0.7110030062 | 1.494313328 | 2.261348565 | 2.951529025 | -0.075850594 | 0.6288554063 | 0.5827313406 | -0.5083329767 | 0.4600610583 | -0.405012554 | 0.0702594396 | 0.4950030271 | 0.7051111791 | -0.3064516558 | -1.410653008 | -0.1631850803 | -0.5105667971 | 0.04485892479 | 0.3746603702 | -0.2231191925 | 3.004125517 | 3.247912708 | 3.606881531 | 2.977827064 | 3.931236145 | 3.483167165 | -0.3648622816 | -0.2658780771 | -0.331011119 | -0.2054041693 | -0.2470242049 | -0.370253348 |
| NNMT | 2.159723472 | 3.159723472 | 4.159723472 | 5.159723472 | -0.3340644624 | 0.1511995284 | 1.40918172 | 0.1561833999 | 0.3129421893 | -1.354526628 | 0.5622476845 | 1.14557469 | 1.079854529 | 0.823006181 | -2.151424015 | 0.292370435 | -0.5035285173 | 0.2938305413 | 0.3859576491 | -0.5248278487 | -0.1360440741 | -0.5071522522 | -0.5309897517 | -0.2986336289 | -0.5671994644 | -0.4097944192 | 0.8475446185 | 0.5046187467 | 0.3897720977 | 0.5966998538 | 1.286705799 | -0.08896623243 |
| NDUFS1 | 0.4991841034 |  |  |  | 0.0305841162 | 0.1301145823 | 0.6012020655 | -0.2494157196 | -0.2681788104 | -0.7522777993 | 0.0770371341 | 0.3804417319 | 0.7847399464 | 0.0818379109 | -0.9267518545 | 0.0122991376 | 0.1767029823 | 0.148552062 | 0.1510709735 | -0.09885259 | 0.8835972522 | 0.8226927169 | 1.312202466 | 1.673894067 | 1.972708007 | 1.687911111 | -2.135300506 | -2.418963098 | -1.936157467 | -2.421249256 | -2.355997313 | -2.176355474 |
| COL3A1 | 3.863909144 |  |  |  | -0.1735042725 | -0.6620240088 | 2.73886767 | -0.8009441113 | -0.004099373 | -2.032492673 | 0.486310939 | 0.8679983611 | 2.960507744 | 0.217215326 | -3.645327964 | 0.1532268997 | -0.9172858425 | 0.01128527493 | 0.1310616684 | -0.4088705315 | -0.02228439454 | 2.049600969 | 2.023076347 | 2.319675178 | 2.786795175 | 2.242251516 | -0.57193908 | -0.1167757692 | -0.2823566815 | -0.2219132329 | -0.3477409996 | -0.01515253885 |
| TRPM2 | 0.8458910825 | 1.732868043 |  |  | 0.2497875189 | 0.7408342873 | 0.3370599226 | 0.0143132391 | 3.003745298 | 2.092033692 | 0.319387401 | 0.0116137408 | 0.3678954277 | 0.1886280418 | 0.5934971756 | 0.3954394812 | 0.2694207536 | -0.1082886951 | -0.0807595398 | -0.1412591846 | -1.214407524 | -1.084940865 | -0.9814490712 | -1.692301258 | -1.110371526 | -1.494737329 | 1.560313239 | 0.8169646795 | 0.9026361914 | 0.4375866437 | 1.207491586 | 0.794859416 |
| MYD88 | 0.2554712574 |  |  |  | 0.1225352155 | 2.235953791 | -0.1277617127 | -0.0474708091 | 2.038686527 | 1.095273295 | 0.3048372838 | -1.610402212 | 0.0974132965 | 0.5196189613 | 0.1453507331 | 0.0835532928 | 0.402012313 | -0.1248850868 | -0.0252840464 | -0.111614719 | 2.057273449 | 1.741535926 | 1.984462418 | 1.620661101 | 2.002955167 | 2.375705041 | -0.8837370704 | -0.7593590916 | -0.7924938108 | -0.6598748317 | -0.7790879046 | -0.8442712529 |
| P2RY13 | 1.106900311 | 0.5535506121 |  | 0.8238881433 | -0.0781445983 | 2.863827926 | 0.4044137167 | 0.0790201983 | 1.767510062 | 1.393743165 | -0.0298771207 | -0.1803995032 | -0.0649424947 | -0.1054561878 | 0.1157025928 | 0.1054019859 | 0.4580170431 |  |  | -0.7155517204 | 2.68680462 | 2.829979658 | 2.364262877 | 2.47010435 | 2.598997119 | 3.487196671 | -0.492950382 | -0.7694014275 | -0.5435090342 | -0.5148669727 | -0.4092325255 | -0.4666741196 |
| MMP9 | -0.0433494451 | 0.1334105496 | 2.21782889 |  | -0.0322155043 | 2.742935538 | -0.566199039 | -0.071570771 | 2.686386566 | 2.084016965 | 0.5740850022 | -0.2100771876 | -0.4836552325 | -0.0070132199 | 0.7288937379 | 0.1542724927 | 0.8610944383 | -0.2690429872 | -0.378875837 | 0.893597029 | 2.51386849 | 3.14878887 | 3.219660094 | 1.443083965 | 1.742463902 | 3.005488043 | -0.4223694455 | -0.2076466305 | -0.2680468815 | -0.174503357 | -0.184859332 | -0.2490286248 |
| DTYMK | -0.2774958895 | 0.7225041105 | 1.722504111 | 2.722504111 | 0.24184183 | -0.3521535674 | -0.4300174419 | -0.6729335338 | -0.6971205137 | -0.2691969399 | 0.0363853706 | 0.1761193509 | -0.3145073069 | -0.0900279717 | 0.0354290699 | 0.067722827 | 0.3003942862 | 0.03403674744 | 0.1053653731 | -0.6483664089 | 1.759877609 | 1.388875772 | 1.878628244 | 2.564041992 | 2.635849277 | 2.49916843 | -0.761960455 | -0.684112871 | -0.8507896001 | -0.8926077505 | -1.102512642 | -0.9760512031 |
| CLK4 | -1.142880588 | 0.7766015733 |  | 3.069650711 | 0.0090730688 | -0.149356072 | -1.217583061 | -0.4012728677 | -0.3531709689 | -0.2850539389 | -0.0683923451 | 1.519341713 | -0.4574402726 | -0.0399002862 | 0.0907502128 | 0.2052182026 | 0.6000580861 | -0.07590451777 | -0.0661648387 | -0.9017401344 | -0.8952466848 | -0.9962887141 | -1.283671651 | -1.74383559 | -1.606112884 | -1.462134692 | -1.202720524 | -0.1940419091 | -0.6613924291 | -0.3292661786 | -0.8777465106 | -0.9451565145 |
| LOX | 1.917389374 | 2.917389374 | 3.917389374 | 4.917389374 | -0.0007313562 | 0.1242491908 | 1.772262446 | -0.0500162786 | 0.0118306228 | -1.587375369 | 0.2634807952 | 1.23896701 | 1.914557311 | -0.2168618566 | -3.228565139 | -0.0178615711 | -0.5155580514 | 0.1808186804 | 0.1374061236 |  | -0.1855754236 | 0.1193348341 | 0.107196096 | -0.1540333734 | 0.07219594956 | -0.04963551754 | 0.0250422705 | -0.1768408243 | -0.1080765311 | 0.1899409498 | 0.01472899025 | -0.2495186335 |
| CCS | -0.0750449894 | 0.9249550106 | 1.924955011 | 2.924955011 | 0.0107009629 | -0.2897376773 | -1.072920276 | -1.687587732 | 0.6339522065 | 1.17926565 | 0.1298255295 | -1.630801048 | -1.256981326 | 0.0014302086 | 1.609681352 | -0.1184432122 | -0.3784116951 | 0.1595647406 | 0.1873813864 | -0.7436511522 | 0.2760581157 | 0.2579072539 | 0.5857992567 | 0.8320640173 | 0.04305318567 | -0.4532663407 | -0.3179005351 | -0.8351977716 | -1.07443944 | -0.6773272711 | -0.2359461068 | -0.6709523698 |
| NAAA | -3.008346859 | -2.008346859 | -1.008346859 | -0.0083468593 | 0.0254373751 | 1.294470761 | 0.2656616021 | -0.0346028411 | 1.476894067 | -0.6481281519 | 0.0271502527 | -0.2033163439 | 1.507811787 | 0.1411440653 | -2.739248236 | -0.1295746823 | 0.5552635251 |  |  |  | 0.6916662901 | 0.8034698139 | 1.035551178 | 0.4348443152 | 1.179098074 | 0.5153800607 | -0.5627247649 | 0.105085216 | -0.3362389173 | -0.3096591366 | -0.2086333588 | -0.3446732573 |
| RXRB | -0.0975855426 | 0.9024144574 | 1.902414457 | 2.902414457 | 0.0018621224 | -0.4192459511 | -0.0060165485 | -0.253420984 | -0.098909144 | 0.0118638958 | 0.1900264135 | -1.554690622 | -0.148453462 | 0.2275144606 | 0.3226075931 | -0.1088135143 | -0.3494735414 | 0.09860439957 | -0.0215979035 |  | -1.716055937 | -1.828316573 | -1.456684469 | -1.874429088 | -1.685954685 | -1.833592386 | 0.9048338167 | 0.8889273815 | 0.9976537497 | 0.9105229902 | 0.8469850654 | 0.9182265438 |
| MMP8 | -0.8665894504 | 1.21782889 |  |  | 0.0057841834 | 3.410070902 | -1.665081883 | 0.2019762879 | 2.896242679 | 3.279521611 | -0.127682006 | 0.989255009 | -1.300964009 | -0.5369610901 | 1.457288896 | 0.0954626553 | 2.882526325 | -0.1748470683 | -0.2092849837 | 1.224970998 |  |  |  |  |  |  |  |  |  |  |  |  |
| HSD17B10 | 0.2033438648 |  |  | 2.015272017 | 0.0166020143 | -0.3028386388 | 0.2369309133 | -0.770347468 | 0.0516961979 | 0.0056334176 | -0.0266588951 | -0.4672649599 | 0.272064797 | 0.1496882708 | -0.3692514663 | -0.0218847448 | 0.0101008026 |  |  |  | 0.7037085598 | 0.5403937392 | 0.5005492287 | 1.832482707 | 1.464583023 | 0.9555918307 | -1.437790929 | -2.833331543 | -1.443747805 | -1.619747113 | -2.751802443 | -2.046974572 |
| GSTM1 |  |  |  |  |  |  |  |  |  |  |  |  |  |  |  |  |  | 0.3307762325 | 0.1950901643 | 0.2415965483 | -2.831949519 | -2.588554114 | -2.686986189 | -0.6441060676 | -0.4310790905 | -0.8008317684 | 1.981519807 | 2.55670652 | 2.521294968 | 2.664108344 | 1.208968057 | 2.061920981 |
| FGR | -2.154371626 | -1.154371626 | -0.1543716256 | 0.8456283744 | 0.0012163353 | 2.672882663 | -1.351770607 | 0.4305924218 | 2.519745463 | 2.13768938 | -0.1050173222 | -1.385853156 | -0.6245795468 | 0.5435599247 | 1.113844353 | 0.1135304582 | 1.483656501 | -0.3286954006 | -0.4682911895 | 0.7653869861 |  |  |  |  |  |  |  |  |  |  |  |  |
| NT5E | -0.0085629682 | 0.9914370318 | 1.991437032 | 2.991437032 | 0.0368841785 | 0.6696510825 | 0.1784887386 | 0.3471214263 | 0.0247245435 | -0.2228407015 | -0.0584655989 | -1.227784077 | 0.303456502 | 0.0996685255 | -0.1457895959 | -0.2677588153 | 0.6525337611 | 0.2058281036 | 0.6053324395 | -0.8777911223 | 1.628993746 | 1.440431679 | 2.664634021 | 1.829434491 | 3.421915843 | 2.794081421 | -0.4707140868 | -0.2056661935 | -0.696815872 | -0.536857509 | -0.3970048876 | -0.5163373807 |
| PRKAA1 | 0.4845129246 |  |  |  | 0.9321062427 | 0.1920781344 | 0.3795876475 | -0.1486062881 | 0.2623289553 | -0.2029001581 | -0.0703317442 | 2.198483025 | 0.4612955414 | 0.2302230074 | -0.4007747118 | 0.0515100995 | -0.3291831841 | -0.1213815169 | -0.1769066991 |  | 1.308199292 | 1.454622351 | 1.431555068 | 0.9809490582 | 1.52704956 | 1.61250149 | -0.4444264602 | -1.318918545 | -0.8711156691 | -0.9755378645 | -1.080142039 | -1.319320492 |
| ACSS1 | -0.4402899228 |  |  |  | 0.0211786768 | -2.276692871 | -0.3712304017 | -0.8738922501 | -0.8871129747 | 0.61289805 | -0.1941257591 | -1.500463262 | -0.8930619189 | 0.1281846044 | 1.747725817 | -0.118662483 | -0.5086762219 |  |  |  | 0.3674901093 | -0.06172027756 | -0.6868285984 | 0.1871817017 | 0.8709364371 | 0.09254992706 | -1.340993487 | -1.559739514 | -1.517190855 | -1.405820572 | -2.257172419 | -1.186215314 |
| EGLN3 | 1.728033413 |  | 2.333500584 |  | -0.1571700073 | 0.1671780584 | 1.275862143 | -0.3744388038 | 0.8993381228 | -1.243681833 | 0.3127382993 | 0.3128998625 | 1.51331042 | 0.6477928492 | -2.353075014 | 0.2661671527 | -0.1205786615 | -0.06097167661 | 0.2900702614 | -0.227505665 | 0.788948251 | 0.8425186594 | 0.5630387033 | 1.138419947 | 1.438363599 | 1.643018779 | -0.6767828795 | -0.1988775991 | -0.5369216591 | -0.5168197 | -0.1269502849 | -0.4372831758 |
| CDH2 | 2.085076778 | -0.2646427803 |  |  | 0.025976316 | 0.5424226735 | 1.679974683 | -0.0747191873 | 0.6665962314 | -0.9642681514 | 0.3452865439 | 1.326752687 | 1.49031324 | -0.053913086 | -2.52839434 | 0.1738460461 | 0.25839776 | 0.4481560615 | 0.6282113437 | 0.2685051868 | -0.7089065898 | 0.571740917 | -0.1657789904 | 1.510144399 | 1.154414343 | 0.7698129559 | 1.187743952 | 0.9444207381 | 0.9642956968 | 0.6144008445 | 1.302287268 | 0.8456975797 |
| BRD4 | -0.19350414 | 2.056398551 | 1.764512703 | 4.988220892 | 0.0663774874 | -0.5761841274 | -0.3721510419 | -1.269797023 | -0.3871147338 | -0.052607069 | 0.171317981 | -0.8552506885 | -0.092431734 | -0.0242710618 | 0.2751212964 | -0.0613015812 | 0.0142676622 | 0.01622394437 | -0.0228800966 | 0.0605300135 | 1.030359066 | 1.269946623 | 0.8277214259 | 1.305148435 | 1.836578393 | 1.317589361 | -0.07985008842 | -0.1058401857 | 0.3094823402 | -0.1590234548 | 0.451750791 | 0.1968500447 |
| IGF1R | 3.814378085 | 4.814378085 | 5.814378085 | 6.814378085 | 0.0549302555 | 1.633398907 | 3.071257308 | -0.0838785851 | 1.446683386 | 1.483146195 | 0.1868502865 | -0.2703464177 | 2.260557212 | 0.2378155579 | 0.8157477786 | 0.1169481051 | 0.3071074401 | -0.0001810525943 | -0.0712989605 | -0.2174799953 |  |  |  |  |  |  |  |  |  |  |  |  |
| AURKA | 1.153401183 | 2.153401183 | 3.153401183 | 4.153401183 | -0.2105098401 | 0.1768592897 | 1.16907155 | -0.4481803933 | 0.5569667966 | -0.5927046976 | 0.0589464493 | 1.001420373 | 1.000847813 | 0.0165909423 | -1.542107187 | -0.0332772766 | -0.3247710012 |  |  |  |  |  |  |  |  |  |  |  |  |  |  |  |
| GSTZ1 | -0.1754330312 |  |  |  | -0.0869943701 | 0.2134023661 | 0.0643467923 | 0.2623994694 | 0.175135298 | 0.1821482101 | 0.3720000338 | -0.2482509211 | 0.4339822903 | 0.2934098829 | 0.465628585 | 0.160511332 | 0.1472467056 | 0.3348319592 | 0.6147047987 | -0.4672099945 | 2.227984849 | 2.343443473 | 1.959049582 | 3.395785903 | 2.68217073 | 3.03108462 | -0.4911845801 | -0.5693904064 | -1.060115303 | -0.9680678385 | -0.8688352068 | -1.047054405 |
| KIF2C | 3.113421034 | 4.113421034 | 5.113421034 | 6.113421034 | 0.2143659407 | -0.3016366168 | 2.604268922 | 0.0084388371 | 0.4580671566 | -1.703489869 | -0.1312124506 | 0.9297699554 | 2.257160553 | 0.3627574916 | -3.006927833 | -0.5936679551 | -0.5601247191 | 0.3504247353 | 0.4244403088 | 0.0431963223 | -0.2544958396 | 0.06683853153 | 0.5174885406 | 0.2368001504 | 0.1979817358 | 0.4662222339 | 0.01086882959 | -0.2010737268 | -0.228199132 | -0.02797332042 | -0.2465947239 | -0.3455476722 |
| CBR1 | -1.078615525 |  |  |  | -0.8637027166 | 0.491518827 | -1.514863495 | 0.2387171072 | -0.041481332 | -3.815825834 | 0.5748060354 | 0.1995591516 | 2.399157199 | 0.0532724308 | -4.134361192 | 0.1688309625 | -0.1003891688 | 0.2538476691 | 0.32398961 |  | -0.1139026356 | -0.3122541032 | -0.1130502801 | -1.1683089 | -1.657543586 | -1.741971994 | 0.07219912864 | -0.3870985109 | -0.5243454113 | -0.613567521 | 0.1737664896 | -0.2664153533 |
| GCSH | 0.8778102037 | 1.877810204 | 2.877810204 | 3.877810204 | -0.0958581028 | 0.1232963739 | 0.9223589321 | 0.0104018352 | 0.2654254404 | -0.4496797444 | 0.0264318218 | 1.980796197 | 0.5862951627 | 0.3763199434 | -0.8488819745 | 0.0872822962 | -0.3883454027 | 0.3050209698 | 0.5299730757 |  | 0.2930039449 | -0.4818692845 | 0.6392141249 | 1.918107859 | 1.89428457 | 1.49330339 | -0.6508967191 | -0.7487330829 | -0.5090357865 | -0.6118203606 | -1.002587975 | -0.8611616211 |
| CTSV | 2.223072041 | 3.223072041 | 4.223072041 | 5.223072041 | -0.0778357942 | 0.775844267 | 1.720734721 | -0.6336616679 | 0.7690554687 | -1.068669361 | 0.2158658629 | 0.6449178158 | 1.780825069 | 0.0082629408 | -2.879470515 | 0.0171489042 | -0.4281651596 |  |  |  |  |  |  |  |  |  |  |  |  |  |  |  |
| GOT1 | 0.2341910764 |  |  |  | 0.1242097783 | -0.408322593 | -0.7748438509 | -0.4927578978 | -0.8226366196 | 0.1416282788 | -0.468818635 | 0.6458855571 | -1.169784782 | -0.1049858752 | 0.8811124888 | -0.022596941 | 0.1305281904 | 0.2190334191 | 0.354409216 | 0.1926785245 | 0.4154275275 | 0.6722352439 | 0.05748468867 | 2.644452669 | 2.199880541 | 1.587951925 | -1.510371184 | -0.9397679569 | -0.9815830452 | -1.508278857 | -1.689086499 | -1.015262396 |
| PDXDC1 | 0.2499439108 |  |  |  | 0.0708065794 | 0.3459859737 | 0.5800736148 | 0.2117337198 | 0.0287303929 | -0.8885063729 | 0.3837519976 | 0.183017854 | 0.6132608299 | 0.2064015873 | -1.282787759 | 0.0698862353 | 0.0527055828 |  |  |  | -1.122620097 | -1.011417742 | -1.124696897 | -1.287858476 | -1.113132531 | -1.043513844 | 0.6886731929 | 0.9723234163 | 0.9821773208 | 0.9387772779 | -0.5331837404 | 1.214291774 |
| PYGM | -0.6349218609 | 0.3650781391 | 1.365078139 | 2.365078139 | -0.06844842 | -0.6042140424 | -0.4495156807 | -0.6181537628 | 0.3047505647 | 0.7151175965 | 0.0918831676 | 1.359479565 | -0.2828472181 | -0.1253243958 | 0.3975514483 | -0.0414978345 | -0.588958957 | 0.2875511258 | 0.2114508704 | -0.5613880623 | 1.046839409 | 1.868425264 | 0.9879426417 | 1.761111987 | 1.767876032 | 2.689316007 | 0.5153134122 | 0.1943069256 | 0.7035649418 | 0.308175144 | 0.4198062449 | 0.5288069117 |
| KCNJ5 | -0.3996970798 | 0.8692119727 | 1.94043059 | 3.095892398 | 0.0286940272 | 0.0353996589 | -0.3899379751 | -0.1882202584 | 0.1558938574 | 0.1409328379 | -0.0569971131 | -0.6390164405 | -0.2786351478 | 0.1118106171 | 0.1340952954 | -0.0755394795 | 0.1208916776 | -0.1249457017 | -0.2243160262 | -0.4217598102 | 2.810457171 | 3.425602197 | 2.432192268 | 2.067604553 | 2.027720779 | 2.770450851 | -0.4482419004 | -0.5017386634 | -0.4918798845 | -0.6782336883 | -0.1863018199 | -0.8148693638 |
| MDM2 | 0.2394936079 | 0.8553545665 |  |  | 0.0099028541 | 0.6841028794 | 0.092041683 | 0.6020280697 | 0.5330818354 | 0.435557125 | 0.8731274706 | 0.4967559426 | -0.1611769994 | 0.0501246361 | -0.1277557239 | 0.5064105285 | -0.0095669843 | 0.2671644183 | 0.2427474298 | 0.1078258668 | -0.9791353461 | -0.7460538497 | -0.2984673053 | -1.486909782 | -1.073456356 | -0.5810205422 | 1.165491107 | 1.593881378 | 1.512148259 | 1.438589874 | 0.7825826167 | 1.12543381 |
| PEBP1 | 1.018536829 |  |  |  | 0.7742949007 | -0.3119178507 | 0.4925034019 | 0.1519111378 | -0.1771666126 | -0.4004276223 | 0.1208610488 | -0.0572329861 | 0.7255663992 | 0.3291727728 | -0.6949244888 | 0.04224349 | -0.3528035649 |  |  |  | -1.261198497 | -1.450201949 | -1.389241216 | -1.860171602 | -1.577710645 | -1.36236787 | -0.04382434521 | 0.6095582672 | 0.4192356437 | 0.7823905582 | -0.8197777817 | 0.3238444221 |
| HK2 | 1.66968811 | 1.431761869 |  |  | -0.003950362 | 0.5037079846 | -0.4118541314 | -0.0687626893 | 0.4435925467 | 0.2595001116 | 0.1346484214 | 2.236767637 | -0.0699415417 | 0.0563446998 | -0.3017177271 | 0.0735790918 | 0.3231347692 | 0.189923875 | 0.1343106878 | -0.1352948147 | 1.622559582 | 0.2376817475 | 0.6756610331 | 0.828037662 | 1.451527184 | 1.390802076 | -0.5193552826 | -0.8515121898 | -1.049624776 | -0.5169560878 | -0.6779690041 | -0.7610161616 |
| SMARCE1 | 0.5496945546 |  |  |  | 0.4792466037 | -0.384804567 | 0.147945259 | -0.6066964674 | -0.2165902015 | 0.1063316297 | 0.0798759311 | -0.8587277021 | 0.050403665 | 0.1376361326 | 0.3314903471 | -0.0790773936 | 0.4796940817 | 0.1036827105 | -0.0086662555 | -0.325747821 | 0.7836401967 | 0.3973814286 | 0.6227157408 | 0.9506976498 | 1.304153777 | 0.7116857901 | -1.386251986 | -2.452213097 | -1.925543596 | -1.828754836 | -1.982217598 | -1.758724351 |
| CCR1 | -0.5986901596 |  |  |  | 0.054405296 | 3.297357831 | -1.356067622 | 0.1624826068 | 3.128316233 | 2.751033556 | 0.2481239563 | -0.9882053907 | -0.9751989222 | 0.27649043 | 1.515928484 | 0.320099341 | 1.937140477 | -0.1498679189 | -0.3684471569 | 1.604396471 | 0.1083154317 | 0.02730622418 | 0.8041273385 | 0.1387843496 | 0.2600321973 | 0.3535028683 | -0.084478828 | -0.2071244518 | -0.2493703256 | -0.1995394056 | -0.06083758171 | -0.2795484742 |
| TAB1 | 0.0720019389 |  |  |  | -0.0627670957 | -0.4240505752 | 0.0470808227 | -1.568730183 | 0.2684618825 | 0.0993537331 | 0.0385864933 | -1.861029636 | -0.1479900746 | -0.2728227303 | -0.2467937958 | -0.2225411931 | -0.3438228221 |  |  |  | 0.5990976591 | 1.025584661 | 0.5178782003 | 0.8873589307 | 1.536964876 | 0.8475476743 | -0.6994191015 | -1.252435014 | -0.8841355489 | -0.93133064 | -1.003852125 | -1.081013206 |
| AURKB | 2.997168415 | 3.997168415 | 4.997168415 | 5.997168415 | 0.0586329969 | -0.1524002965 | 1.391614559 | -0.7472340396 | 0.1622557475 | -0.4626005661 | 0.0824424975 | 0.953137424 | 1.328544952 | -0.6012985194 | -1.74249163 | -0.1232110333 | -0.5463690976 |  |  | 0.1532784076 |  |  |  |  |  |  |  |  |  |  |  |  |
| IMPDH2 |  | 0.2575617404 |  |  |  |  |  |  |  |  |  |  |  |  |  |  |  | 0.01279135433 | 0.1645065296 |  | 0.9896306387 | 0.6002131457 | 0.8085678942 | 1.532233962 | 1.910926433 | 0.7964594314 | -1.517286815 | -0.981425611 | -1.685764392 | -1.409705103 | -1.147095135 | -1.073364255 |
| PRKAR1A | 3.651335888 | 4.651335888 | 5.651335888 | 6.651335888 | 0.1953511559 | 0.2956113016 | 2.416063022 | 0.3157806031 | 0.149194891 | 0.3506832316 | 0.4048095451 | -2.268415037 | 2.041921089 | 0.2310182387 | 0.5658556945 | 0.188991328 | -0.1567006198 | 0.3687051585 | 0.4207195802 | -0.8298482108 | 0.247171186 | 0.09303522237 | -0.03604473374 | -0.2027200039 | -0.2088118877 | -0.2900347033 | -0.2790790708 | -0.02337340844 | 0.07014383196 | 0.3116933331 | 0.05239790393 | -0.08958075411 |
| ME1 | 1.472137732 |  |  |  | 0.372829888 | 0.334306008 | 1.326640321 | -0.9248244484 | -0.1981765596 | -1.37005381 | -0.1990598767 | 0.5599075888 | 1.29977189 | 0.2062498009 | -2.306882816 | -0.519182197 | -0.3620397544 | 0.02579425388 | 0.2002396188 |  | -0.01225113347 | -0.1642067304 | 0.3153545291 | 0.9130488922 | 1.496147261 | 0.7159837841 | 1.432942724 | 0.5126218916 | 0.9698752506 | 0.6145826434 | 0.5516335958 | 0.7116874928 |
| SLC16A5 | -0.7354091281 | 1.738310639 | 1.674450146 | 2.928961988 | 0.6113382339 | -0.4743119081 | -0.3590249267 | 0.1648173817 | 0.1333820948 | 0.0552781863 | 0.0581333214 | -0.1217910752 | 0.1028054497 | -0.0970589922 | -0.1302382298 | -0.0748999719 | -0.1926524749 | 0.2151568477 | 0.2745943159 |  | -1.093642992 | -1.014831285 | -0.9354256399 | -0.0112521635 | -1.033886845 | -1.055690139 | 0.5122553712 | 0.4486742343 | 0.5599350075 | -0.004391758215 | -0.4078019072 | -0.2546258836 |
| HTR4 | 1.82265702 |  |  |  | 0.1162591046 | 1.254231321 | 1.023545347 | 1.035811167 | 1.189138459 | 0.441001859 | 0.1297837425 | 0.0350299958 | 1.004889176 | 1.019251108 | 0.2601459064 | 0.0478197261 | 0.8865529222 | 0.07944102855 | -0.1201773192 | -0.6568706473 | -0.5022768188 | -0.6725041176 | 0.06498990208 | -0.4489412425 | -0.6708895709 | -0.3027884827 | 0.1172236277 | 0.2797678457 | 0.04681005516 | 0.2048933464 | -0.3944003014 | -0.06648528374 |
| PRODH | 0.9472118379 | 1.947211838 | 2.947211838 | 3.947211838 | -0.0479034982 | 0.344275993 | 0.5053836559 | 0.5960802228 | 0.2633719952 | 0.1638865332 | 0.0424884179 | 0.0853993668 | -0.0613554675 | -0.2288071238 | -0.3638472095 | 0.0657150059 | 0.2159361951 | -0.06401902349 | -0.0652553803 |  | 0.5524596199 | 0.8353286188 | 1.30275891 | 2.387635541 | 1.81964382 | 1.727872263 | -0.06737072571 | 0.05509397825 | 0.01291242218 | -0.2346776571 | -0.2566397564 | -0.2082835434 |
| HNF4A | 2.912220877 | 3.912220877 | 4.912220877 | 5.912220877 | 0.1136176158 | 0.0405281743 | 2.260824641 | 0.1990448563 | 0.0535282605 | -0.0038259775 | 0.0510053501 | 0.1987667311 | 1.845356124 | 0.0117762695 | -0.0497386944 | 0.1108145905 | 0.1151450435 | -0.08523139163 | -0.1000449424 |  | 0.2644017985 | 0.5492817233 | 0.05203290748 | 1.613840384 | 0.2148323099 | 0.1856852305 | -0.3483157105 | -0.0321258134 | -0.218542172 | -0.3463118751 | -0.2372842195 | -0.4042547151 |
| CASK | 1.358021222 | 2.358021222 | 3.358021222 | 4.358021222 | 0.2178598436 | 1.497032774 | 0.7831438712 | 0.1762278107 | 1.530075078 | 0.3119458038 | 0.0900926345 | 3.330094501 | 0.7195887186 | 0.0830238717 | -0.2622161013 | 0.2406089466 | 0.3872877961 | 0.1040657201 | 0.2465244814 | 0.0234671788 | -0.0773684594 | 0.2970783134 | -0.05248720247 | 0.1165419992 | 0.1695800341 | 0.5567384543 | -0.162254373 | -0.3089231747 | -0.01537163223 | 0.08122261719 | -0.5320242768 | -0.4555636716 |
| HSPG2 | 0.5847447812 |  |  |  | 0.3796110713 | -0.102753673 | 0.3536055302 | -0.4307165545 | 0.5311644656 | 1.255154411 | 0.5187774188 | -0.033965494 | 0.1404700172 | 0.7114146964 | 1.804637969 | 0.277558474 | -0.0062445857 | 0.1251890414 | 0.2564122567 | -0.2008019798 | 0.5006129287 | 1.674400109 | 1.298474649 | 1.157393147 | 1.737037713 | 1.429780884 | -0.6362116324 | -0.4211783503 | -0.4746759935 | -0.7176982203 | -0.611019902 | -0.6408977002 |
| CA6 | 2.464367068 |  | 1.620440792 | 3.252423534 | -0.0079381882 | 0.2154361072 | 0.978666184 | -0.0304552765 | -0.1679987892 | -1.454954988 | 0.5787194575 | 0.0928190988 | 1.35317586 | -0.1761875879 | -2.637080273 | 0.3244945535 | -0.0603681899 | 0.06771761532 | 0.0344304435 | -0.3458921391 |  |  |  |  |  |  |  |  |  |  |  |  |
| TNFRSF1B | -0.9787171067 | 0.0212828933 | 1.021282893 | 2.021282893 | -0.0105052385 | 0.7450928249 | -1.344983492 | -0.2229405913 | 0.6591972165 | 1.676092754 | 0.3086778748 | -0.8818274305 | -1.413654572 | 0.2479234851 | 1.983659617 | 0.2965425036 | 0.3324356342 | 0.05484597856 | -0.0709565427 | 0.2367127228 |  |  |  |  |  |  |  |  |  |  |  |  |
| VLDLR | 0.8733913279 | 1.873391328 | 2.873391328 | 3.873391328 | -0.0037448302 | 0.1375572416 | 0.9538138506 | 0.136144054 | 1.423483895 | -0.7520913437 | 0.4385262279 | 0.3208025434 | 1.24080634 | 0.8529610109 | -1.405326545 | 0.1376951566 | -0.2982180954 | 0.0109034724 | 0.1968364737 | -0.4821972552 |  |  |  |  |  |  |  |  |  |  |  |  |
| PGLYRP2 |  | 0.5518392036 |  |  |  |  |  |  |  |  |  |  |  |  |  |  |  |  |  |  | -1.276985021 | -1.394045034 | -1.52345418 | -0.5503646422 | -0.9280039072 | -0.7925371664 | 0.672832154 | 1.016842924 | 1.187764644 | 0.8765720437 | 1.497041052 | 0.8552038927 |
| CTLA4 | -1.873191366 |  |  |  | 0.1157427915 | -0.303343414 | -1.941383982 | -0.0906598519 | -0.02017447 | 1.131392036 | -0.02340563 | 2.657683226 | -1.953103839 | 0.2404881183 | 2.18321864 | 0.3439462786 | 0.7159003181 | -0.3046280335 | -0.5190208267 | -0.1798715939 | -0.1294492985 | 0.01546428656 | -0.02907917397 | 0.1504957887 | 0.2389584706 | -0.171690669 | -0.2774103079 | -0.1259649954 | -0.4384580707 | -0.1981600285 | -0.1127193564 | -0.1418189419 |
| TXNDC12 | 0.7875360605 | 0.9124001662 |  |  | 0.1207805183 | -0.2047385513 | 0.8470930415 | -0.8652979858 | 0.1540636496 | -0.2739554904 | 0.0586509962 | -0.3717013531 | 0.5487429419 | 0.2148082333 | -0.7220012581 | 0.0262066345 | -0.1283292472 |  |  |  | -1.2021417 | -1.839197211 | -1.567338242 | -1.367347827 | -1.055842072 | -1.657291934 | 0.7728713183 | 0.7587180726 | 0.548630043 | 0.5984765427 | 0.5308031895 | 0.6834362503 |
| EPHB3 | 0.4690199689 | 1.723810189 | 1.926030882 |  | -0.0113366424 | 0.2625255041 | 0.4991952532 | -0.4319131579 | 0.3337209013 | 0.2028073445 | 0.1263446591 | 1.219258341 | 0.1158154017 | 0.1227791886 | -0.0958659284 | 0.0490184499 | 0.172147599 |  |  |  | 0.2317443548 | 0.7215375454 | 0.6261562906 | -3.337002426 | -3.149582834 | -3.203347622 | -0.2945196496 | -0.1237053964 | -0.3646573859 | -0.09136688426 | 0.644577831 | -0.1870446977 |
| CAMK1 | -0.1559990373 |  |  |  | 0.2711113177 | 0.3038271393 | 0.3514812128 | 0.5799887897 | 0.6301596997 | 0.3919430948 | 0.104520285 | -0.6925066492 | 0.0550806863 | 0.1295294568 | -0.0167667762 | -0.0571416322 | -0.2047502925 | -0.1446403366 | -0.2846003823 | 0.1091113827 | 2.802615012 | 3.05381531 | 2.649248783 | 3.021177516 | 3.364603447 | 3.9047284 | -0.5193390373 | -0.6451534227 | -0.5432332574 | -0.6940478064 | -0.5659189934 | -0.5733250931 |
| TRPV5 | 0.2713683027 | 2.327194595 | 1.877810919 | 2.518475964 | -0.1787666415 | 0.7338184098 | 0.0633106006 | 0.6874036865 | 1.339205849 | 0.5825377968 | 0.1411435583 | -0.1070989845 | 0.0085602379 | 0.2245590058 | -0.0056463249 | 0.2182438688 | 1.575084197 | 0.2861918359 | 0.2152343258 |  | -0.06078620428 | 0.02921914929 | -0.03299802679 | -0.2065894508 | -0.01872210921 | -0.2727802978 | 1.160779453 | 0.6710616196 | 0.3772681931 | 0.1493736931 | 0.9049632675 | 0.1070519636 |
| ENG | 0.1564444889 | 1.156444489 | 2.156444489 | 3.156444489 | -0.0040579408 | 0.2184691106 | 0.2034423569 | -0.2924285721 | 1.227873461 | 0.5467053494 | 1.045675954 | -0.5186560975 | 0.0122157994 | 0.8635097118 | 0.3891043817 | 0.6903549254 | 0.2115640771 | 0.1133468233 | -0.1111692762 | -0.7893245617 | 0.1939844856 | 0.8864373752 | 0.1801686219 | 0.6762279741 | 1.883097993 | 0.6836289085 | -0.5132222696 | -0.7135297057 | -0.3249981328 | -0.5621973813 | -0.7094988568 | -0.7287562225 |
| GALE | 0.6566864778 | 1.656686478 | 2.656686478 | 3.656686478 | 0.0081255898 | 0.472754586 | 0.5456236668 | -0.3918424007 | 0.4738964458 | 0.5961470719 | 0.1859698418 | -0.2227629646 | 0.896922861 | 0.156834356 | 0.2144740075 | 0.1293438378 | 0.2538587156 | 0.008869320928 | 0.1706579738 | -0.152590341 | -0.4187720514 | -0.532131419 | -0.1884539582 | -0.0709935832 | 0.2697699258 | -0.008724045374 | 1.067227053 | 0.6515268839 | 1.158182086 | 1.049120235 | 0.9176757773 | 0.8656457938 |
| ACADS | 3.30578999 | 4.30578999 | 5.30578999 | 6.30578999 | 0.0088426186 | -0.4704221042 | 2.308880066 | -0.2767940375 | 0.2624094794 | 0.0641291626 | 0.1020316209 | 0.0270817706 | 1.027329173 | 0.4472648672 | -0.4486262687 | 0.0490815501 | -0.3141034049 | -0.3688486498 | -0.1934495805 | -0.0336115658 |  |  |  |  |  |  |  |  |  |  |  |  |
| MTOR | 4.009339977 | 5.009339977 | 6.009339977 | 7.009339977 | 0.4120215469 | 0.2612185648 | 2.414066497 | 0.016497875 | 0.1727364245 | 0.2768428199 | 0.1160422621 | 0.0616436818 | 1.319767506 | 0.1563356763 | 0.285752561 | 0.2027121131 | 0.09051239 |  |  |  | -0.9166974912 | -0.8226825891 | -0.7148050273 | -0.8190251049 | -0.6434367947 | -0.7459346358 | 0.03115699277 | 0.6442048903 | 0.5810581841 | 0.5662650635 | -0.3850047709 | 0.3778985857 |
| PCDH19 | 0.7076079075 | 1.590328336 | 3.10817373 |  | 1.144462282 | 0.3762651268 | 0.9877886522 | 0.2517165549 | 0.7357140838 | 1.754203346 | 0.4762908822 | 0.3027962017 | 1.103742743 | 0.9959762091 | 2.6724249 | 0.2141597694 | -0.0329097976 |  |  |  | 0.2597200077 | -0.04850791043 | 0.641823464 | 0.2096141991 | 0.3554858818 | 0.1050254735 | -0.02641575033 | 0.05679969517 | -0.1697838211 | 0.1475154497 | 0.143323162 | -0.02578026663 |
| FABP7 | 0.3404409758 | 0.0615732541 | 2.337480582 |  | 0.0053108562 | 0.5382873914 | 0.2549885237 | 1.954324472 | 0.6778993211 | -0.1973843655 | 0.1936966124 | -0.1154673266 | 0.3785619255 | 0.1373224838 | -0.4240589076 | -0.0553331275 | -0.1319190177 | 0.2037457962 | 0.2669786587 |  | 0.9750855773 | 0.8217180669 | 2.469503552 | 1.95484213 | 3.572254192 | 1.625189238 | -0.06066777412 | 0.1629295616 | -0.2328673149 | -0.3395097445 | -0.09920163579 | 0.04838131197 |
| ITGB6 | 1.410995838 | 1.128748784 | 1.640435764 | 7.203020251 | 0.0235411987 | -0.355965687 | 0.8400341658 | -0.0179385131 | 0.448204708 | 1.313185964 | -0.0736983461 | -0.3538044293 | 0.7314755785 | -0.1038011819 | 1.51253342 | -0.066263318 | -0.148668048 | 0.2656940604 | 0.1500979035 | 0.5152901939 | -0.12148559 | -0.05752839134 | -0.1758063118 | -0.3323188936 | -0.3101403285 | -0.2185083636 | 0.07142874485 | -0.0719068063 | 0.09038642766 | 0.1497166998 | 0.8353779343 | 0.4447481834 |
| PAK1 | 5.63297373 |  |  |  | 0.2136610753 | 0.3863286949 | 4.649471838 | -0.3521784174 | 0.3617235628 | -1.801259094 | 0.0740154761 | 0.5121737796 | 3.282577003 | -0.0826671391 | -4.668919333 | 0.017564093 | -0.0417530421 | 0.003719355572 | 0.407034701 | -0.1074019634 | -0.09387764506 | 0.5232614362 | 0.1859159194 | 0.3266931229 | 0.8286556929 | 0.4774878236 | 0.077811864 | -0.04334343648 | 0.2257369484 | -0.1946383312 | -0.2474622033 | -0.3290594172 |
| YWHAQ |  |  |  |  |  |  |  |  |  |  |  |  |  |  |  |  |  | 0.1761581561 | 0.2622616018 |  | -0.5312497493 | -0.6477956241 | -0.9287675431 | -0.7482172405 | -0.6781665042 | -0.650598006 | -1.887656784 | -1.271518747 | -1.249380034 | -0.9391248026 | -2.192407362 | -1.719047424 |
| CRYZ | 0.9571111905 | 1.957111191 | 2.957111191 | 3.957111191 | -0.0206797008 | -0.2414912133 | -0.4506250432 | -1.024216212 | 0.0593707711 | -0.0527569175 | 0.2024483971 | -0.1493726517 | 0.0426786951 | 0.1405410071 | -0.3781167327 | 0.161180388 | -0.2704653097 | -0.03515444266 | 0.1682716411 | -0.058739714 | -0.4218814086 | -0.1893437006 | 0.8166017585 | 1.207835376 | 0.9063788867 | 0.3700096807 | -0.1196570388 | -0.2828692112 | -0.08711662524 | 0.284591748 | -0.110188653 | -0.1664500191 |
| RRM1 | 1.601957802 |  |  |  | 0.0008984255 | -0.0258528358 | 1.070498074 | -0.7662138026 | -0.5030550191 | -1.147266128 | 0.2667376715 | -0.4019264906 | 1.127046299 | -0.0460157161 | -1.502951881 | 0.1400417802 | 0.5034271576 | 0.065370108 | 0.3441430121 | -0.4929112318 | -0.6537062125 | -0.4561340231 | -0.7667584787 | -0.3105362628 | -0.2107753847 | -0.4249554571 | 0.09548101604 | 0.007480743247 | -0.1484665031 | 0.27824578 | -0.07013344067 | 0.2231677021 |
| PIK3C2G | 0.011752731 | 1.011752731 | 2.011752731 | 3.011752731 | 0.2614203823 | -0.6120003734 | 0.1206123144 | -1.89336119 | -0.21076336 | -0.0483693816 | -0.0438689851 | -1.530716215 | 0.0685617772 | -0.7522340624 | -0.2385548477 | -0.020086736 | -0.5363505752 | -0.4023973942 | -0.2670100974 |  | -0.4204032424 | -0.4538926222 | -0.4382487595 | -0.3739051108 | -0.2378577754 | -0.5641911705 | 0.1676017044 | 0.1536140353 | -0.09424453762 | -0.1608482525 | -0.1735318516 | -0.09751423606 |
| TAOK1 | -0.3986212787 |  |  |  | -0.0397395592 | 0.8005650597 | -1.158763153 | -0.0067016397 | 0.3014730773 | 0.8595359253 | 0.1175570842 | -0.0261364076 | -0.8956792524 | -0.0666167107 | 1.039597418 | -0.0635935022 | -0.9852979804 |  |  |  | 1.216634895 | 0.8909564718 | 0.5616970565 | 0.5960051063 | 0.7159339196 | 1.041882468 | -0.6710958776 | -0.9984331651 | -0.9173736849 | -0.8396247923 | -0.9378189005 | -1.170767582 |
| IRAK1 | 0.5254063971 |  |  |  | 0.1811791938 | -0.2302016082 | 0.0116226087 | -0.0136096426 | -0.23296927 | 0.343521223 | -0.0743526945 | 0.1805809962 | 0.1330855936 | -0.0253181439 | 0.521289701 | 0.0663808903 | 0.2008173371 |  |  | -0.2606454635 | -0.9348514945 | -0.5532674118 | -0.4915391412 | -2.183115828 | -1.999073941 | -1.516089047 | 1.714736121 | 0.8699463079 | 0.7697681266 | 0.490333485 | 1.668798782 | 0.9904550929 |
| IDH1 | -0.0404253598 |  |  |  | 0.0097555878 | 0.6193554299 | -0.2029806238 | -0.5599426345 | 0.9012649364 | 0.4052805331 | 0.1330994185 | -0.7307277568 | 0.1587096491 | -0.0381926292 | -0.2694757554 | -0.072982521 | 0.3092668313 | 0.4704286364 | 0.5007828996 | 0.0233505241 | -1.091320637 | -1.270255839 | -0.031254722 | -0.32175123 | 0.2668869616 | 0.1746070221 | 0.5966345095 | 0.9739984848 | 1.044325548 | 1.665519782 | 1.08531082 | 0.547519645 |
| S100A4 | 1.197901561 | 1.106793435 |  | 2.937284927 | 0.040190313 | 0.5660477766 | 0.8815121379 | -0.5862687943 | 0.8429854002 | -0.2693528529 | -0.2379044575 | 0.2425815802 | 1.087207061 | -0.1559377331 | -1.727933514 | -0.1582907048 | 0.0729030843 | -0.09025152883 | -0.2172255216 | 0.268084768 |  |  |  |  |  |  |  |  |  |  |  |  |
| UGDH | 1.237716933 | 2.237716933 | 3.237716933 | 4.237716933 | 0.0103086605 | -0.3367219419 | 0.8632336602 | -0.710075448 | -0.1255807812 | -0.8018202816 | -0.0429714089 | -0.0345019022 | 0.7987291492 | -0.2001940666 | -1.549227602 | -0.2890525446 | -0.2380139107 | 0.1089524412 | 0.2561246961 | -0.0023611436 | -0.6790608004 | -0.9054561185 | -0.4134670313 | -0.4132943639 | -0.3030179487 | -0.7340837848 | 0.4039330379 | 0.12214115 | -0.5835069202 | -0.5974379678 | 0.2690809304 | -0.1502094269 |
| KIF11 | 1.0032958 |  | 2.083554252 |  | -0.2810134862 | 0.2766204793 | 1.034848579 | -0.1705708818 | 0.8704387557 | -0.5008930242 | 0.3284053104 | 0.8838088135 | 1.172839451 | 0.1083308935 | -1.590983528 | 0.0749630726 | -0.005130165 | 0.3166497094 | 0.3305819082 | 0.6061019468 | -0.06614742898 | 0.05070625935 | 0.260028323 | 0.1558101984 | 0.2034106928 | 0.2009381081 | -0.1449048113 | 0.1402267354 | 0.01153035683 | -0.009400167156 | -0.03793188372 | 0.02460626588 |
| NEK2 | 1.675739365 | 1.300241122 |  |  | 0.0099408626 | -0.1116899799 | 1.375185977 | -0.4211769059 | 0.2204903738 | -0.4653702386 | 0.2809483041 | 0.8148682029 | 1.328251547 | 0.2035964781 | -1.463582975 | 0.1919964346 | -0.189802457 | 0.1824889548 | 0.3152687787 | -0.2846411863 | 0.189206994 | -0.02631593325 | -0.2279703454 | 0.03878726279 | 0.3099145173 | 0.1947579818 | -0.123432776 | 0.07285977354 | -0.3297938893 | -0.1339770856 | -0.1705078888 | -0.0770158319 |
| ALDOA | 1.08172307 | 2.08172307 | 3.08172307 | 4.08172307 | 0.6366284942 | -0.4647136188 | 0.7295144636 | 0.013745596 | 0.7764184727 | -0.4335056459 | 0.2085041178 | -0.6283725724 | 0.8463119383 | 0.1933333905 | -1.400075657 | -0.0797235033 | -0.7468052501 | 0.3698445879 | 0.4376176744 | 0.0117750916 | -0.1770707536 | 0.09198273181 | 0.1015797612 | 0.1470912277 | -0.3312486255 | -0.02180377427 | 0.008331212367 | 0.333024514 | -0.06833560004 | 0.1623691424 | 0.04369898522 | 0.04143430568 |
| IMPA1 | -0.4197709879 |  |  |  | -0.0287534515 | 0.189780028 | -0.6375852213 | 0.0303882219 | -0.2549772973 | 0.0316215204 | -0.016966674 | 0.0512689936 | -0.6847188648 | -0.1039058796 | 0.2778681875 | -0.1122460568 | 0.0041738765 | -0.3058520679 | 0.3567492765 | 0.334731363 | -0.1544656255 | -0.000998677816 | -0.1402689875 | 1.298941964 | 1.301413037 | 1.015802534 | -1.149967851 | -0.994618604 | -1.101522117 | -0.9203830161 | -0.7525221106 | -0.7768513504 |
| ENO3 |  | 1.812311857 |  |  |  |  |  |  |  |  |  |  |  |  |  |  |  | 0.1043899922 | 0.2657034005 | 0.1768686458 | 1.017580136 | 1.001165625 | 0.5205087673 | 0.761860102 | 0.6328777754 | 0.7092650443 | -1.07544886 | -0.3573993708 | -0.6017403467 | -0.5750688641 | -1.59471737 | -0.6534962589 |
| IGFBP4 | 0.2613485652 | 0.9515290249 | 3.204078622 | 1.932944369 | -0.0290248775 | 0.3347613008 | -0.2504742522 | 0.2091170924 | 1.114900393 | 0.5540427493 | -0.1377962489 | -0.469560668 | -0.1411700258 | 0.5029665612 | 0.2070775893 | -0.4669136913 | 0.2139696606 | 0.07951443383 | 0.2479141 | -0.3759916702 | 0.7048122024 | 0.343508742 | 0.2137526304 | 1.732257594 | 0.7092590569 | 0.4610897707 | -0.9335234821 | -1.088911461 | -0.8858178773 | -0.7178663536 | -0.8030610486 | -0.8639390868 |
| YES1 | 3.010449018 |  |  |  | 0.2196193921 | 0.7103986666 | 1.698249912 | 0.2114370423 | 0.2254256887 | -0.2355166081 | 0.1254189931 | 1.512363893 | 1.256010322 | -0.0406299547 | -1.179770384 | 0.1597206735 | 0.5402393401 | 0.09732962961 | 0.0283628713 |  |  |  |  |  |  |  |  |  |  |  |  |  |
| SMO | 0.831344743 | 1.831344743 | 2.831344743 | 3.831344743 | -0.0276239395 | 0.2865065153 | 0.6418772221 | 0.0737338054 | 0.6978691961 | -0.6863638307 | 0.1835865028 | 0.8215131539 | 1.152827095 | 0.6307762651 | -1.335486985 | 0.1443458474 | -0.1277772574 | 0.09945753953 | 0.1307041669 |  | -0.4612255108 | -0.1153531443 | 0.3418259032 | 0.6110127993 | 0.9663895849 | 0.2620118308 | 0.1779206105 | -0.05885119678 | 0.2196978282 | 0.1104229158 | -0.3082155445 | 0.1442930442 |
| PPIH |  |  |  |  |  |  |  |  |  |  |  |  |  |  |  |  |  | -0.001533526782 | 0.0445573899 |  | 0.4584986092 | 0.2637655213 | -0.2003987618 | 1.165344809 | 1.286679424 | 1.342462895 | -0.9300334712 | -0.6791280058 | -1.146008041 | -1.049112124 | -0.7961352183 | -0.8793999341 |
| PSAT1 | 1.269063612 |  |  |  | 0.0554709123 | 0.0192894905 | 0.465368488 | -0.5196849493 | -0.1761314341 | -0.7643393063 | -0.0242517616 | 0.6762269413 | 0.5421768676 | -0.2636837901 | -1.725121686 | -0.2227590694 | -0.1729441722 |  |  | -0.2157881567 | 0.2214751422 | 0.03824903909 | 0.01638361913 | 1.159655353 | 0.9686216545 | 0.3813941325 | -0.9674974957 | -0.9884566812 | -1.07337179 | -0.8352759055 | -0.9499578913 | -1.003548599 |
| VAMP1 | 0.3200861412 |  |  |  | 0.1452579225 | -0.9077395024 | 0.0710485144 | -0.4808922922 | 0.3578828977 | 0.4424361851 | 0.0840025518 | -0.4192393048 | 0.0730114857 | 0.3423410197 | 0.5734922873 | 0.0970998446 | 0.8581051642 | 0.06626207107 | 0.0036357789 | -1.127830695 | 1.001468482 | -0.001702059225 | -0.1303665935 | -0.0743731757 | -0.2674562368 | -0.2052770535 | -1.337978758 | -0.8145468733 | -1.280257762 | -1.071318881 | -0.6142326287 | -0.8340617706 |
| CCNB1 | 1.50610666 |  |  |  | 0.8105059472 | 0.2079864871 | 1.333077397 | 0.0003757768 | 0.1443939714 | -0.5353901466 | 0.2331258182 | 1.299482577 | 0.9264731341 | -0.0220137997 | -1.45258504 | 0.1366523289 | -0.2616505746 | 0.1844876243 | 0.2359846821 | -0.1649181761 | 0.4638837355 | 0.3681788591 | 0.6830133004 | 0.6520596378 | 0.7140244702 | 1.178700602 | 0.05169178863 | 0.3521308111 | 0.09114549553 | -0.141629038 | 0.292325929 | 0.1878327043 |
| PRMT5 | 0.437538575 |  | 1.721495323 | 3.717234603 | 0.0558093815 | 0.168231154 | 0.3932984582 | 0.5631918326 | -0.66212966 | -0.5626513494 | 0.0415068858 | 0.2561449885 | 0.3965915393 | 0.1191253739 | -0.4614969118 | 0.0038052493 | 0.3069320455 |  |  |  | -0.6274055589 | -1.202745126 | -1.422707725 | -0.6263222924 | -0.3768960411 | -1.140137974 | -0.1240123014 | 0.4362503102 | 0.4512567422 | 0.710651552 | -0.03888394936 | -0.5580117475 |
| OAS1 | 1.992641511 |  |  |  | 0.1302038845 | 2.166398686 | 1.450188488 | 1.935237111 | 1.266771399 | 0.3830403093 | 0.2638788669 | -0.0493144207 | 0.6370150719 | 0.082608007 | 0.5575468683 | 0.1445702513 | 0.6636976422 | -0.3021872997 | -0.1130856717 | 0.4425276936 |  |  |  |  |  |  |  |  |  |  |  |  |
| HAL | 0.1728006991 | 1.172800699 | 2.172800699 | 3.172800699 | 0.1597556259 | 0.7945203674 | 0.6486155854 | -0.1594178621 | 0.6187418128 | 0.4094436038 | 0.2385124849 | -0.1350098195 | 0.1120807918 | -0.019642856 | 0.2365353641 | 0.3884443963 | -0.2462259229 | -0.1638362316 | -0.3539389648 | -0.256904137 | 0.2292599593 | 0.1408682838 | 0.5181735118 | 1.651971962 | 1.120781232 | -0.1655352939 | 0.0645538261 | -0.1097275479 | 0.01221806488 | 0.08635472129 | -0.4426412799 | -0.4944965167 |
| PF4 | -0.373407047 |  |  |  | 0.0125416362 | 0.0675987807 | -0.4126445717 | -0.0440387887 | 2.366657124 | 2.062572456 | 0.2890646441 | -0.044554076 | -0.6765301014 | 1.448975029 | 2.186226 | -0.0057083719 | 2.175264094 | 0.08382084524 | -0.4633818288 |  |  |  |  |  |  |  |  |  |  |  |  |  |
| AHSG | 0.2942269704 | 1.29422697 | 2.29422697 | 3.29422697 | -0.1198100612 | -0.3423528474 | 0.876146957 | -0.2973332974 | 0.1031240537 | -0.4310938077 | -0.0052382237 | 0.3105035931 | 0.825328923 | -0.0624937835 | -0.7570491243 | 0.119197569 | -0.2900125152 | 0.3366443487 | 0.5534557317 | -0.0743954363 | 0.3832214175 | 1.494690814 | 0.4052322926 | 3.662085598 | 0.501318454 | 0.1554255619 | -0.2579699971 | -0.1080615456 | -0.2175576731 | -0.3720669083 | -0.4275375392 | -0.3471282346 |
| GNAS | 1.139165254 | 2.139165254 | 3.139165254 | 4.139165254 | 0.3710653103 | 0.1034510622 | 0.310522368 | 1.14592557 | 0.9287329959 | 0.3734917669 | 0.6708189474 | 0.7809126759 | 0.5020220958 | 0.4004549515 | 0.3770197785 | 0.1255448377 | -0.2425728068 | 0.1021094758 | 0.1248026796 | -0.1151505634 | -0.7360999461 | -0.5698302681 | -0.9700064729 | -0.7365152555 | -0.837448107 | -0.6217958193 | 0.5843835189 | 0.6784507327 | 0.9331658033 | 0.5884131062 | 0.8051564996 | 0.8589511794 |
| MTTP | 0.583439212 | 1.583439212 | 2.583439212 | 3.583439212 | -0.0074277696 | 0.0286799758 | 0.3722279307 | 0.0205816594 | 0.1983860538 | -0.2292400105 | 0.1727566504 | -0.1052530972 | 0.284467325 | 0.2168187018 | -0.5289908181 | -0.0147447622 | 0.3181232586 |  |  | -0.7256606794 | -0.6420662702 | -0.5628477531 | -0.4566494941 | -0.001519333911 | -0.2833429344 | -0.6879360241 | 0.9537936 | 1.076058823 | 0.8051828012 | 0.2939216245 | 1.347690426 | 0.4781568404 |
| NFATC1 | -0.4903403267 |  |  |  | 0.0200981313 | 0.1081366401 | -0.1700693616 | 0.7280498658 | 0.1565292543 | 0.4536701382 | 0.3103664047 | 0.08580582 | -0.4855345163 | 0.1846276647 | 0.7934484236 | 0.2128389562 | 0.0929637287 | 0.07642359437 | -0.0773293491 | -0.1683398419 | 1.021878814 | 1.343553455 | 0.7886911661 | 1.25131672 | 1.559398321 | 2.098208051 | -0.5275233529 | -0.277692881 | -0.7594621025 | -0.499102275 | -0.3800389238 | -0.3825469517 |
| NDUFV3 | -0.5797915501 | 2.773330169 | 1.57366932 |  | 0.1193529386 | 0.3753427593 | -0.5017904266 | -0.1336225464 | 0.0476971901 | 0.6489568043 | -0.0764051485 | 0.0117615439 | -0.4718993904 | 0.3733427798 | 1.356997036 | -0.0690535034 | 0.6637205638 | 0.309449705 | 0.46086606 |  | 0.6221870895 | -0.1195258542 | -0.1480943162 | 0.8868694367 | 1.125638827 | 1.300782435 | 0.2172541864 | -0.2954658781 | -0.1619626457 | -0.4751820172 | -0.5162654896 | -0.4451829441 |
| NAE1 | -0.3406605328 |  |  |  | 0.0121089021 | -0.030580304 | -1.607979475 | -0.2918727444 | -0.1835734438 | 0.606672774 | 0.052377073 | 1.443658568 | -1.772409935 | 0.2293426661 | 1.471315933 | 0.3548056114 | 0.5038692369 |  |  |  |  |  |  |  |  |  |  |  |  |  |  |  |
| HNRNPF | -1.289929869 | -0.289929869 | 0.710070131 | 1.710070131 | -0.4709994236 | -0.5582767747 | -0.9522087998 | -0.1581567288 | -0.3587440777 | -0.2875152695 | -0.1297413503 | 0.3273094737 | -0.46145132 | -0.1898378934 | -0.102490398 | -0.1506252049 | -0.1323086744 |  |  |  | -0.5106452796 | -0.2670394514 | -0.2950568842 | -0.7410577727 | -0.5520392456 | -0.05647695307 | -1.211278937 | -0.4312057436 | -1.307642178 | -0.6916696657 | -0.4786521049 | -0.8991842503 |
| MKNK2 | 0.5181413541 | 1.014436902 |  |  | 0.1275695008 | -0.6825202465 | 0.8699702108 | -1.3877355 | 0.0404150103 | -0.8166718286 | 0.3718177524 | -0.9525512302 | 1.480785681 | 0.2603810465 | -1.283478702 | 0.1028432917 | -0.4767788951 | 0.4295956447 | 0.2445946898 | -0.2097567096 | -0.497160419 | -0.01410366864 | -0.3615875273 | -0.5293641629 | -0.6837696181 | -0.4345238439 | -0.3134514095 | 0.09029949221 | 0.00822427665 | 0.03374990187 | -0.8975193175 | 0.0605145561 |
| AZGP1 | -1.119495872 |  |  |  | -0.0175368381 | -0.4605134841 | -0.7772732357 | -0.7765056881 | 0.7406721511 | 0.5726875629 | -0.1617531755 | -1.478316337 | -0.6657430033 | 0.2709735909 | 0.6902349167 | 0.1223651629 | -0.362211625 | -0.0884360208 | 0.2645394088 | -1.099982463 | 0.06391418802 | -0.105921533 | 0.1099316912 | 2.427094331 | -0.04889462308 | -0.06967097557 | -0.1357503997 | -0.2590621817 | -0.4403122988 | -0.5352365849 | -0.1909060464 | -0.3419999259 |
| HPN | -0.4530857026 | 0.5469142974 | 1.546914297 | 2.546914297 | 0.0399245964 | -0.9285235453 | 0.1691840392 | -2.395358404 | -0.5197763772 | 0.1570156798 | -0.1145882763 | -2.596548505 | -0.35692705 | -0.8564056202 | 0.2012647941 | -0.170460075 | -0.395711483 | 0.1371139016 | 0.5987180759 | -0.3897142579 |  |  |  |  |  |  |  |  |  |  |  |  |
| ATP1A3 | -0.1030249467 | 1.799449384 | 2.514167277 |  | 0.2536726589 | -1.138122267 | 0.3408988488 | -1.671894046 | 0.9352821803 | 0.7086009514 | -0.0574480246 | 0.3377127555 | 0.7480608726 | 0.1580486654 | 0.1768391033 | 0.2443176445 | -0.5076853172 | -0.1385981417 | -0.1916659514 |  | 0.1290044265 | -0.1884777505 | -0.01704591269 | 0.3400456253 | 0.2481657207 | 0.3521645124 | -0.2404595411 | -0.1768256074 | 0.0007301330111 | -0.3548178551 | -0.05114664902 | -0.3149702747 |
| LDHC | 0.6194718263 | 1.619471826 | 2.619471826 | 3.619471826 | 0.0118113762 | -0.3010165353 | 0.3860415294 | -1.156124872 | 0.5448005465 | 0.3334052535 | -0.1363350392 | -0.974606461 | 0.1533838162 | 0.482684354 | 0.1521574953 | -0.1106672412 | -0.3476034264 | 0.0103631515 | 0.3287268714 |  | -0.04581890819 | -0.1827837335 | 0.1355274708 | 0.03156217171 | 0.0775981419 | -0.1263493581 | 0.5053125088 | -0.2352036669 | -0.209260007 | 0.1367481705 | 0.08279921575 | -0.4801613891 |
| TOX | 0.0908576356 | 1.090857636 | 2.090857636 | 3.090857636 | -0.1012621481 | -0.6620762191 | 0.0616165573 | 0.2539389184 | 0.1851603614 | 0.2128715056 | -0.0284313216 | 2.030660274 | 0.0815516285 | 0.4274237672 | 0.3666333618 | -0.1264098346 | 0.0618862705 |  |  | -0.0618926296 | 0.01935569144 | -0.4300894476 | -0.29696179 | -0.172738893 | 0.07702715228 | -0.2056976732 | -0.1282461209 | -0.02765830322 | -0.1648097145 | 0.3751089935 | -0.331390455 | -0.2461988745 |
| JAK3 | -0.1184503853 | 0.2740971167 | 1.36729881 |  | 0.7369164158 | 1.397040688 | -0.009965687 | 0.1223644567 | 1.301419685 | 1.019068626 | 0.4951932215 | -0.5685944051 | 0.4885293306 | 0.4844470152 | 0.7103418581 | 0.2713094443 | 0.3124427136 | 0.241946058 | 0.1079993827 |  | -0.4041514742 | 0.1125034992 | 0.01231657139 | 0.08473039289 | 0.3322203103 | 0.274878226 | -0.5099778816 | -0.3590921173 | -0.5080287039 | -0.2351672199 | 0.09105090012 | -0.0681297955 |
| VCAN | 0.325435181 | 1.325435181 | 2.325435181 | 3.325435181 | 0.1719505267 | -0.0659076362 | 0.1861233719 | -0.4683028341 | 0.4202107606 | 0.431162699 | 0.0013135995 | -0.0080061649 | 0.2034494856 | -0.0096297967 | 0.1664272039 | 0.0566143124 | -0.2079078523 |  |  |  | -0.5253967803 | -0.1323030593 | -0.1096792305 | -0.5058861742 | -0.7271185182 | -0.6305163834 | 0.8019692335 | 0.3840304165 | 0.2184408171 | -0.02557671579 | 1.024189904 | 0.2823430855 |
| KYNU | -0.1965171646 | 0.8034828354 | 1.803482835 | 2.803482835 | 0.4700018908 | 0.0949110474 | 0.1330969341 | -0.1136145076 | -0.6208768391 | 0.8655553246 | 0.5199150863 | 0.3473277334 | -0.703251755 | 0.3135958228 | 1.572256889 | 1.407885248 | 0.9376385476 | 0.3584779784 | 0.3740280583 | -0.6242998898 | -0.04884678992 | -0.3073124985 | -0.3138716535 | 0.01811965295 | -0.04931914151 | -0.238014337 | -0.5807889877 | 0.3081277272 | -0.6768318744 | -0.153656166 | -0.2024562335 | -0.2060254406 |
| FGG | 0.2443671516 | 1.244367152 | 2.244367152 | 3.244367152 | -0.0114871407 | -0.1038936199 | 0.0153292237 | -0.0894414723 | -0.5838215671 | -0.0735834211 | 0.0807289017 | -0.3857440564 | 0.28898535 | -0.4781890009 | -0.1309895505 | 0.0244758866 | 0.5118120928 | 0.387045898 | 0.9160837388 | -0.2168780526 | -0.2033443088 | -0.0712508276 | -0.2087613766 | 2.602293575 | -0.3722820147 | -0.4011888045 | -0.1886078201 | -0.2686449293 | 0.321334492 | 0.1346753187 | 0.1566507695 | -0.3472653404 |
| ACVR1 | 1.23961433 |  |  |  | -0.0006377921 | 0.5356228283 | 0.7590945172 | 0.8118951828 | 1.152451463 | -0.1631287874 | 0.4653433207 | -0.0259393706 | 1.306069569 | 1.059228502 | -0.3886985307 | 0.1405428713 | 0.5466643299 | 0.1758468167 | 0.258869038 | -0.4523212664 | -0.3322143941 | -0.3083738747 | -0.1739326517 | -0.1588304776 | 0.2017750465 | 0.07315802275 | -0.0112172168 | -0.4909034241 | -0.1138443321 | -0.2062412548 | -0.2492455039 | 0.2322471639 |
| ACOX1 | 0.6568705292 |  |  |  | -0.0947872636 | 0.0586634854 | 0.1153619708 | -0.6597707309 | -0.0539738844 | 0.5448365359 | -0.0701295498 | -0.6144200186 | -0.325441251 | -0.3409200506 | 0.4766032492 | -0.3633550622 | -0.4134127533 | 0.2335531434 | 0.2270279351 | 0.382494949 | 0.7667901717 | 1.145611959 | 0.7283577701 | 1.746209537 | 1.460717042 | 2.110259023 | 0.8099934273 | 0.9273463826 | 0.7816905514 | 0.7774344093 | 0.8425851693 | 0.7702863055 |
| PLA2G1B | -0.0136307466 | 0.9863692534 | 1.986369253 | 2.986369253 | 0.0335052666 | 0.4666997091 | -0.2590501761 | 0.2326692571 | 0.3699266444 | 1.048164404 | 0.0455723202 | 0.4745200551 | -0.1008318896 | 0.1559550464 | 1.236585887 | 0.0257507297 | 0.5650663124 | 0.1650176181 | 0.1603635832 | 0.2687911329 | -0.2151331942 | -0.07641941029 | -0.2084606728 | -0.4592943854 | -0.2386963091 | -0.2099507256 | -0.08173141628 | 0.09035627283 | 0.114296789 | 0.3381238002 | -0.1347375403 | 0.2239665804 |
| FGA | 0.2189502389 | 1.218950239 | 2.218950239 | 3.218950239 | -0.1974406655 | -0.0517044227 | 0.1479804907 | 0.2648493517 | 0.4831732013 | -0.2281655014 | 0.0007458038 | 0.199883983 | 0.2119115204 | 0.036693757 | -0.6109456824 | -0.0090819923 | -0.2325437658 | 0.1085662731 | 0.2328412182 |  | -0.2833055369 | -0.4738517954 | -0.4329476003 | 1.775731652 | -0.5606957645 | -0.4888578138 | -0.2083281442 | -0.01041009617 | 0.2897062974 | -0.1290460886 | -0.2674386094 | -0.07782940487 |
| KCNJ3 | -0.0595694105 | 1.095892398 | 3.455581242 |  | -0.0099432371 | -0.4011930095 | -0.0217626652 | -0.4007228919 | 1.377822422 | 1.403191209 | -0.0555887223 | -0.0971864638 | -0.577894167 | -0.438718536 | 0.4570892991 | 0.0148102082 | -0.2368436123 | -0.1624934129 | 0.1019349974 | 0.1102115741 | -0.4015894273 | -0.2876670925 | 0.02037633128 | -0.09206696741 | -0.07013393214 | -0.2404950608 | 0.1069134509 | 0.1911109898 | 0.3116044093 | 0.2441709051 | 0.7980105227 | 0.3132661766 |
| SI | 0.6813726783 | 1.681372678 | 2.681372678 | 3.681372678 | 0.1043752768 | 0.5014570063 | 0.5694625941 | 1.097865533 | 0.1804023845 | 0.6245380711 | 0.0079745168 | -0.0621880633 | 0.1772721686 | 0.1653544254 | 0.4595476139 | 0.0498411944 | 0.0700742637 | -0.1433751473 | -0.2836382178 |  | 0.735301811 | 0.01471019372 | 0.2411796701 | 0.07082362737 | 0.09419311691 | -0.04963687916 | -0.4170440049 | -0.4220204922 | -0.03646124817 | -0.4945129252 | -0.4886136194 | -0.5144342406 |
| CSNK1G3 |  | 2.581459541 |  |  |  |  |  |  |  |  |  |  |  |  |  |  |  | -0.01154996311 | -0.0105363552 | -1.163126946 | -0.2175719069 | -0.7948448799 | -0.5758868591 | 0.01847625582 | 0.487897594 | 0.1223471083 | -0.3623938029 | -1.085933579 | -0.4937417807 | -0.2217233347 | 0.2442218138 | 0.7986319301 |
| OXCT1 | 0.5122838371 |  |  |  | 0.0838481772 | 0.2522560851 | 0.7892860847 | 0.3286734105 | -0.5415170833 | -0.9019556617 | -0.1569825997 | 0.2587307609 | 0.8317522166 | -0.0767321211 | -1.13311062 | -0.1958329409 | -0.1199215506 |  |  |  | 0.03710688492 | -0.3253313085 | -0.735872916 | 0.8418028572 | 1.073701371 | 1.512889855 | 0.09402563271 | -0.09426697593 | -0.1388569433 | 0.1698374374 | -0.2494091664 | 0.3608688567 |
| HADH | -0.0008723298 | 0.9991276702 | 1.99912767 | 2.99912767 | 0.0020464492 | -0.7305593409 | -0.4121692104 | -1.108097091 | -0.5306977884 | -0.4105483989 | 0.2747024454 | -0.1664065966 | 0.3919383251 | 0.2536368939 | -0.1283629131 | -0.0952367104 | -0.2055749413 |  |  |  | -0.2479785804 | -0.5027979174 | -0.4198399505 | 0.619933349 | -0.02926543171 | -0.08829667183 | 0.03580604583 | 0.2382599359 | -0.1419937252 | -0.4208822135 | 0.4685709914 | 0.266730305 |
| CYP19A1 | 1.795320287 |  |  |  | -0.1504905365 | 0.1378583984 | 0.5063551293 | -0.3194403174 | -1.600193778 | 0.1069076065 | -0.0853151756 | -0.2463605238 | 0.5758215928 | -1.118905007 | -0.1726942043 | -0.054080613 | -0.0650703267 |  |  |  | 0.9550807115 | -0.0758936827 | -0.07245134291 | -0.1326091908 | -0.4368826828 | -0.2430378961 | -0.6205165666 | -0.784585247 | -0.6777534618 | -0.7753466939 | -0.612569198 | -0.9866084108 |
| CA8 | -0.3956249936 | 1.262673436 | 4.464367068 |  | 0.0210191383 | -0.1740522513 | 0.2165965769 | 0.1252270233 | -0.8228153166 | -0.7565823202 | -0.0184304154 | 0.1855994167 | 0.58048965 | -0.0025255239 | -0.6952989967 | -0.0475178724 | -0.0611981427 | 0.3509859005 | 0.4841189982 |  | -0.7760823918 | -0.9853346521 | -1.277077435 | -0.7997076914 | -0.6962932966 | -0.8732088504 | 0.05036868603 | 0.574399456 | 0.4855141766 | 0.9251106651 | -0.2055232874 | 0.4295350195 |
| NTRK2 |  | 1.102400156 |  |  |  |  |  |  |  |  |  |  |  |  |  |  |  | 0.124468667 | 0.1175773721 | -0.4820315203 | -0.9531361222 | -0.9069967691 | -1.088821048 | -0.8970238814 | -1.003418457 | -0.9774570853 | 0.6051835729 | 0.8252259217 | 0.7804204509 | 0.7877317103 | 0.6434065872 | 0.7521798311 |
| MTHFD2 | 0.7525252159 | 1.08660035 |  |  | -0.0004144875 | -0.3168265184 | 0.7530966737 | -1.254780364 | -0.8475549608 | -0.4366115293 | 0.039859179 | -0.2448834552 | 0.592105748 | -0.5836159057 | -0.8472858084 | -0.1570126234 | 0.1117943244 | 0.1422745577 | 0.1126495052 | 0.8323918836 | 0.02623063286 | -0.01119173989 | 0.5350571544 | -0.3809641161 | -1.156812629 | -0.3436688254 | 0.1486859368 | 0.208428473 | 0.02370899553 | 0.1864477935 | 0.181334644 | 0.2777009871 |
| USP1 | 2.365886106 |  |  |  | -0.0149735928 | -0.3127550635 | 1.740711199 | -0.5008654408 | -0.7318313892 | -0.7048075627 | -0.0558525557 | -0.961177782 | 0.9285230274 | -0.0996951551 | -1.154926161 | 0.0144586007 | 0.4407040504 | -0.2089157992 | -0.187959825 | -0.7735163851 |  |  |  |  |  |  |  |  |  |  |  |  |
| FASN | 0.3725033248 | 1.372503325 | 2.372503325 | 3.372503325 | 0.0037663327 | -0.6001440838 | 0.3550549045 | -0.7087735244 | -0.2514408622 | -0.3328399218 | 0.0625500794 | -0.5869288306 | 0.1359057868 | 0.139807446 | -0.3546088546 | -0.3975610951 | -0.2122954576 | 0.05530653635 | 0.3691831567 | -0.4256533037 |  |  |  |  |  |  |  |  |  |  |  |  |
| RENBP | 0.0995687923 | 1.099568792 | 2.099568792 | 3.099568792 | -0.044872449 | -0.0915959279 | -0.2700801538 | -0.8576069171 | -0.2344120241 | -0.3120347573 | 0.0808145258 | -0.229776725 | -0.2861686917 | -0.1048886578 | -0.3816877068 | -0.0108644784 | -0.1017693737 | -0.09056709144 | 0.1900877024 | 0.3737153978 | -0.009684599829 | -0.09651258898 | 0.4246594534 | 0.2134231974 | 0.6351401833 | 0.3525739728 | -0.4564476523 | -0.3867988487 | -0.1922543445 | -0.1103639538 | 0.06466290684 | -0.2555148953 |
| ULK3 | 0.1984811975 | 1.15652971 | 1.445726695 |  | 0.1951672708 | -0.5594142399 | 0.038529118 | -0.1181991881 | -0.1547795568 | -0.280081364 | -0.1452604397 | 3.221199003 | 0.0135282172 | 0.0313539785 | 0.1365542597 | 0.0642041667 | 0.1279222722 |  |  |  | 0.3198685134 | 0.2924969123 | -0.229582379 | 0.03211415979 | 0.2751346168 | 0.5402213268 | -0.5438444315 | -0.2519547868 | -0.02764270362 | 0.336451185 | -0.4690625846 | 0.2115547864 |
| HEPH | 0.3493205757 | 1.349320576 | 2.349320576 | 3.349320576 | -0.041438022 | -0.0554492336 | 0.2710656473 | 0.1603358857 | -0.0819007368 | -0.1742390115 | -0.1602760108 | 0.2091642458 | -0.0501522842 | 0.1920180175 | -0.0892600259 | -0.1838136986 | 0.1574900145 | 0.1667838658 | 0.0948085147 | -0.0558305733 | 0.09316469905 | 0.4806053058 | -0.1649310247 | -0.1029846446 | -0.2151052645 | -0.1036191748 | 0.7292773759 | 0.159152787 | 0.4585929704 | 0.1627969602 | 0.3253404248 | 0.03759991002 |
| ICAM1 | 2.41853749 |  |  |  | 0.3178909157 | 0.7617200231 | 1.52781663 | 0.7043595075 | 1.26460241 | 0.6206439003 | 0.3760322282 | -0.4043556768 | 0.4117020753 | 0.1732900505 | -0.438469037 | 0.0524050814 | 0.3814320662 | -0.3887053546 | -0.6195636135 |  | -0.918727456 | -0.8296530346 | -0.3042755257 | -0.9921794736 | -0.7260658125 | -0.7768052029 | -0.4505303178 | -0.1486014889 | -0.05699492433 | 0.1271596691 | -0.8812173392 | -0.2582544256 |
| ARPC1B | -0.0244928758 | 0.9755071242 | 1.975507124 | 2.975507124 | -0.0747947845 | 1.471189872 | -0.0722291926 | 0.571555024 | 0.7551533495 | 0.4618456232 | -0.3626189185 | -0.3469655883 | -0.1569584467 | -0.2354612256 | -0.157444232 | -0.1377792725 | 0.7536398809 | 0.03131920691 | -0.0974054153 | 0.013337421 |  |  |  |  |  |  |  |  |  |  |  |  |
| RBP4 | 0.5268867472 | 1.147829654 | 2.100757709 | 4.274195709 | 0.3368095916 | 0.2925453925 | 0.6673563223 | 0.1567851536 | 0.9348179862 | -0.1268501407 | 0.4228816594 | 0.8036657096 | -0.1053115906 | -0.0925621436 | -0.6258915634 | -0.2438216644 | 0.207854907 | 0.2134202227 | 0.6158855618 | -0.185198185 |  |  |  |  |  |  |  |  |  |  |  |  |
| SLC7A11 | 0.9124098737 | 1.109021464 | 1.77133546 | 2.342572093 | 0.0400272359 | 0.1126433312 | 0.0949880063 | -0.3486793677 | 0.2523121087 | 0.6263835544 | 0.1133321061 | 0.1248010438 | -0.3767883899 | -0.4573351174 | -0.1373129899 | 0.4074939405 | 0.2950613176 | -0.05277020388 | -0.1428361366 | 0.2548080783 | 0.04081256133 | -0.4459340513 | -0.3491554792 | -0.02906003412 | 0.1640929377 | 0.385652562 | 0.06847815713 | -0.2180379601 | 0.2443771941 | -0.002570813145 | 0.04290027568 | 0.02183989876 |
| PTH2R | 0.0012119807 | 1.001211981 | 2.001211981 | 3.001211981 | -0.0652098901 | 0.211648928 | 0.1256493718 | 0.1157965428 | 0.8773095242 | 0.1733040214 | 0.0297962824 | 0.0416738435 | 0.0801497552 | 0.6297319907 | 0.1106480264 | 0.0722976106 | 0.0835740869 |  |  |  | 0.1209524493 | 0.1048042469 | 0.2993793136 | -0.07889007294 | -0.06223943429 | 0.1243421252 | -0.3918741468 | 0.2558836082 | -0.1467059035 | -0.04490044816 | 0.09688292172 | 0.9159629619 |
| MMP2 | 2.113964618 | 1.176738913 |  |  | 0.0386009201 | -0.0638168865 | 1.132203449 | -0.4001290348 | 0.6822573262 | -0.3585034524 | 0.243822244 | 0.7188298399 | 0.6240273643 | 0.4261479038 | -0.9962042854 | 0.1206642762 | -0.4097049537 | 0.02701028412 | -0.011486891 | -0.1943009139 | 0.03295502546 | 0.06877441768 | -0.1846167374 | -0.1028676153 | 0.5195703422 | 0.550815353 | -0.01982973081 | -0.06593064904 | -0.2612083413 | 0.2702461245 | -0.3240817023 | 0.03858955974 |
| DLAT | -0.116090953 | 0.883909047 | 1.883909047 | 2.883909047 | -0.0728023668 | -0.1673458931 | 0.3805046931 | -0.1794740166 | -0.2235391903 | -0.6475727076 | -0.1089141144 | 0.5345315729 | 0.5229908249 | 0.0968946385 | -0.7158345121 | -0.1053340789 | 0.0796332884 | -0.05603820254 | 0.1628289427 | -0.43842967 | -0.1660169621 | 0.0393536811 | 0.04813689954 | 0.7682846171 | 1.155587398 | 0.8147707616 | -0.09856230812 | 0.00008837339402 | 0.03120965301 | -0.08945749259 | -0.03565796609 | -0.3616850324 |
| ITPKA | 0.5326912531 | 1.532691253 | 2.532691253 | 3.532691253 | -0.1371542148 | -0.0984967234 | 0.7770475333 | 0.0764840068 | 0.3416622877 | -0.6085548546 | -0.0115914782 | 0.9474707106 | 0.7969793741 | 0.3699022594 | -0.8968715131 | -0.0112969325 | 0.0280533542 | 0.1081333024 | 0.1112676624 | -0.5934351573 | 0.0905155913 | -0.001930821727 | 0.1346525239 | 0.1534987955 | 0.4341774942 | -0.02574619121 | -0.1562080655 | 0.0669439407 | -0.1263402487 | 0.06786167706 | -0.04055967628 | 0.09857616081 |
| UMPS | 0.8244605028 | 1.824460503 | 2.824460503 | 3.824460503 | -0.137821447 | 0.0371099045 | 0.1383174567 | -0.0732244144 | -0.3930628654 | -0.7434981924 | -0.2213347992 | 0.2236795797 | 0.5177593355 | 0.1279933565 | -0.9567582218 | -0.1366245061 | 0.2087551477 | 0.2898363413 | 0.493125768 | -0.4216037857 | 0.04443344516 | -0.06234129234 | 0.6591510734 | 0.4044561141 | 0.4102684676 | 0.2191124686 | -0.1492210783 | -0.1457456279 | -0.1520060076 | -0.1911319218 | -0.08664781786 | 0.03200370389 |
| NUDT9 | 0.3938101596 | 4.779434426 | 1.703964808 | 3.604222826 | 0.4787302151 | -0.2411485392 | 0.5425368921 | -0.1323680029 | -0.2111463706 | -0.1509481817 | -0.0229495207 | -0.4698359482 | 0.1032996042 | 0.1665116228 | -0.238424906 | 0.0300753819 | -0.0007530151 | 0.01714970254 | 0.0623890262 | -0.0481870435 | -0.16930843 | -0.3796577937 | 0.122376972 | 0.3337572608 | 0.5297655322 | -0.2061559602 | -0.810401672 | -0.6245155295 | -0.7676690449 | -0.7924729802 | -0.8119891256 | -0.6910553864 |
| FST | 0.3658428251 | 1.365842825 | 2.365842825 | 3.365842825 | -0.0046727317 | 0.0095223199 | 0.9722745185 | -0.0945097506 | 0.6637041683 | 0.3848406985 | 0.2635507871 | 0.4495437434 | -0.1353463908 | 0.308068604 | 0.3074640267 | 0.11549007 | -0.0310893328 | 0.185434243 | 0.3941144878 |  |  |  |  |  |  |  |  |  |  |  |  |  |
| SLC4A8 | 0.320945306 | 0.2880658551 |  |  | 0.0735599585 | 0.180519809 | 0.1865913264 | -0.0439285846 | 0.474824855 | 0.2801973813 | 0.1780968192 | -0.7006260293 | 0.0228673109 | 0.2879269382 | 0.1333467199 | 0.2339673351 | 0.0267775093 | 0.2660485839 | 0.193797315 | -0.9231271361 | -0.4036546531 | -0.3687166056 | -0.3991804717 | -0.4340004302 | -0.4108110127 | -0.3765855124 | 1.189575607 | 1.109421789 | 0.8579822615 | 0.4573457161 | 1.709903078 | 0.8304701612 |
| MYC | 0.5914537262 | 1.591453726 | 2.591453726 | 3.591453726 | -0.0019669239 | -0.9393886215 | -0.1332024003 | -0.3656715335 | -0.8980848731 | -0.1579015184 | -0.2310173935 | 0.3406585978 | 0.1244752563 | 0.2244157896 | 0.8526994263 | 0.1313405935 | 0.2823714377 | -0.2309799826 | -0.0316468363 | -0.1038104025 | -0.2303966761 | -0.2611364786 | 0.07200602525 | 0.2051799578 | 0.6195843912 | -0.1051863236 | -0.7327921719 | -0.5033904312 | -0.684359944 | -0.5289265825 | -0.8162299204 | -0.6357898667 |
| ATF7 |  | 0.7255288228 | 3.19469105 | 3.918233442 |  |  |  |  |  |  |  |  |  |  |  |  |  | 0.1719928159 | 0.0701102041 | 1.495059462 | 0.04817733679 | 0.5380874487 | 0.2340886692 | 0.2183038698 | 0.07027652972 | 0.1531901488 | 0.05083120343 | -0.02476158721 | -0.1040894087 | -0.0392193162 | 0.02721138585 | -0.1023390613 |
| HTR2B | 0.1766676308 | 1.176667631 | 2.176667631 | 3.176667631 | 0.0776460354 | 0.5078065828 | -0.2799830293 | -0.0535441484 | -0.3367708833 | 0.4657356796 | -0.0451203878 | 0.8356371868 | -0.0776708233 | -0.1231661993 | 0.4867528109 | 0.0589210138 | -0.1816817446 | -0.0320178635 | 0.005337923 | 0.2309762931 | 0.1842799109 | -0.01487395843 | 0.6681281619 | 0.03666202823 | 0.02636663168 | 0.1004051525 | 0.1380789038 | -0.01591203486 | 0.2155163743 | -0.03478364191 | -0.1486235663 | 0.2003562188 |
| SERPINA6 |  | 1.103308929 |  | 3.738552282 |  |  |  |  |  |  |  |  |  |  |  |  |  | 0.242744115 | 0.2569694373 | 0.4586540622 | 0.8716114094 | 0.1413675398 | -0.2336132784 | 1.698822304 | -0.3251904343 | -0.0009524402144 | -0.5582229038 | -0.366530822 | -0.2720318982 | 0.1984524599 | 0.02159222312 | -0.1795644836 |
| ALDH3B1 | 0.9470352456 |  |  |  | 0.2258794176 | 1.330044896 | 0.3598671775 | -0.3667456946 | 1.842997028 | 1.085946912 | 0.0652365104 | -0.7428180831 | 0.2673701962 | 0.3207073925 | 0.1749781273 | 0.2012258082 | 0.3403032473 | -0.02972320676 | -0.0427949886 | 0.101306676 |  |  |  |  |  |  |  |  |  |  |  |  |
| PIPOX | -0.0683646674 | 0.9316353326 | 1.931635333 | 2.931635333 | -0.006916489 | -0.2384662043 | -0.1109078772 | -0.076899467 | 0.1161934425 | 0.2856460187 | -0.0549791944 | -0.0661624879 | -0.2819658779 | -0.0721443004 | 0.3740436763 | 0.036732304 | 0.0359343492 | 0.1491880794 | 0.0765460934 | -0.1694631943 | 0.02435408643 | 0.1780995194 | 0.1057493263 | 1.172721277 | -0.2258298076 | -0.1004406735 | -0.5016751571 | -0.5573807368 | -0.2801857982 | -0.4142652415 | -0.7912088649 | -0.3498185981 |
| CBS | 0.0343207355 | 1.034320736 | 2.034320736 | 3.034320736 | 0.0356344348 | 0.0604969109 | -0.1598688001 | 0.1782770805 | -0.2073000752 | 0.362703389 | -0.0711925863 | -0.1351844112 | -0.2870728796 | -0.5887829621 | 0.3803396918 | -0.0417970718 | -0.1519331558 | 0.1551823175 | 0.3383994285 |  |  |  |  |  |  |  |  |  |  |  |  |  |
| CUBN | 0.8923576352 | 1.892357635 | 2.892357635 | 3.892357635 | 0.005492893 | 0.2228478627 | 0.1522505121 | -0.0937637456 | 0.0474352901 | 0.2124471947 | 0.0728848752 | -0.2748794742 | -0.1919141858 | -0.1565018354 | 0.0219907249 | -0.0131859046 | 0.3522166852 | -0.007844745541 | 0.1224868437 | -0.4575380005 | 0.3404903836 | 0.1443317635 | 0.1710658033 | -0.08042983891 | 0.5728687158 | 0.0251221719 | -0.2232910464 | 0.4087682114 | -0.08280981299 | 0.1817404707 | 0.1017469991 | 0.1737305042 |
| FSHR | 0.6628103014 | 1.662810301 | 2.662810301 | 3.662810301 | 0.0300607698 | -0.0444383161 | -0.3082519878 | 0.0664613347 | 0.2687638932 | 0.4458942996 | -0.0280144077 | 0.0368006715 | -0.5565613132 | 0.2033305306 | 0.5305599485 | 0.0282756625 | 0.1731767673 | -0.01415025467 | 0.0305707666 | -0.0513953906 |  |  |  |  |  |  |  |  |  |  |  |  |
| MAP3K8 | -0.5696498502 | 1.525488725 | 2.038309867 |  | 0.0184972472 | 1.506747286 | -0.3080488162 | -0.0819744946 | 0.6938376279 | 0.8113214374 | 0.465049118 | -0.6043115945 | -0.6140451158 | -0.2207013481 | 0.6886486601 | 0.3721542588 | 0.1144128038 | -0.2813417759 | -0.5114674791 | -0.3170780125 | 0.3359733155 | 0.05286887959 | 0.1036600484 | 0.3130714058 | 0.7462161694 | 0.7025343586 | -0.80935451 | -0.5851497074 | -0.6166684699 | -0.6301183533 | -0.383743672 | -0.443959003 |
| ATG2B | 0.6477872714 | 1.647787271 | 2.647787271 | 3.647787271 | 0.218360005 | 0.0171765951 | 0.3486445311 | 0.1872691597 | 0.2213315476 | 0.6112210334 | 0.2128043407 | 0.2354345383 | 0.1915645082 | 0.1402692832 | 0.9577747702 | 0.0630311366 | -0.0554020212 |  |  |  | 0.2565925062 | 0.2498815673 | 0.9182225428 | 0.1478416247 | 0.2303176471 | 0.2293637214 | -0.2802868694 | 0.1583009996 | -0.3219128746 | -0.2868337391 | 0.05805835728 | -0.3744770915 |
| KCTD8 | -0.4412530774 | 1.393685364 | 2.468689004 | 3.116617469 | 0.1728719719 |  | -0.2338242401 |  | 0.0370197875 | 0.4459347871 | -0.0695876672 |  | -0.3569335496 | 0.18009971 | 0.304758455 | 0.0483121731 |  |  |  | 0.5423867144 | 0.2187593829 | 0.1546564731 | 0.5907398422 | 0.3162857557 | -0.1096365354 | 0.1487535224 | -0.2668250168 | -0.2653289256 | 0.1388121521 | -0.1215848635 | -0.2948689408 | -0.2158200948 |
| F2 | 0.2879230535 |  |  |  | -0.1297207434 | 0.229279976 | 0.23348981 | 0.2880681591 | -1.021466914 | -0.291222868 | 0.0827436447 | 0.1283760685 | 0.0239405002 | -1.498599164 | -0.3685248189 | -0.0003826309 | 0.100969619 | 0.1709978914 | 0.317681903 | 0.3127141807 |  |  |  |  |  |  |  |  |  |  |  |  |
| HSPD1 | 0.9316355573 |  |  |  | 0.3660387946 | -0.1348936569 | 0.9430898043 | -0.0078709406 | -0.6052722607 | -0.7578154625 | -0.1562652973 | 0.0256260532 | 1.091731388 | -0.2336971273 | -1.349151585 | -0.2725489381 | 0.4246649188 | -0.008540392191 | 0.3157818674 |  |  |  |  |  |  |  |  |  |  |  |  |  |
| SMPD3 | -0.0183962802 | 0.1579225788 | 1.796255686 |  | 0.0676550322 | 0.1160819354 | -0.6107771203 | 0.1818675801 | -0.78495313 | -0.292250409 | 0.1095337984 | 0.6106718017 | -0.0815049486 | -1.147678307 | -0.245100661 | -0.0631791421 | 0.547459315 | 0.2275587345 | 0.1116905638 | -0.0038068359 | -0.3871361419 | -0.5330359661 | 0.03828158877 | -0.09044336222 | 0.3179995464 | -0.1131651251 | 0.5448289785 | -0.1320251724 | 0.2649687833 | 0.2163450746 | -0.1591587593 | -0.1407684759 |
| NQO1 | 0.9539708415 |  |  |  | 0.003055309 | 0.4086257577 | 0.1015536645 | -0.2153059581 | -0.2712639829 | -0.5848001688 | 0.2911608603 | 0.6417111129 | 0.446011164 | -0.4731059893 | -1.534727789 | 0.205172656 | 0.3383240703 | 0.3821857387 | 0.4623130612 | -0.0974386587 | 0.3663922413 | 0.3793463418 | 0.7871525135 | 0.7522253115 | 0.6263645172 | 1.196177798 | -0.6679509984 | -0.5393573496 | -0.5638225438 | -0.8948844776 | -0.8852681659 | -0.7312916731 |
| CD33 |  |  |  |  |  |  |  |  |  |  |  |  |  |  |  |  |  | -0.02709470696 | -0.0889807749 | 0.3286023909 | -1.144970136 | -0.5386106362 | -1.121308955 | -0.7830990796 | -0.918531944 | -0.6083448732 | 0.4326650143 | 0.5577372526 | 0.5517180842 | 0.7195400633 | -0.409645569 | -0.3425693009 |
| PAPSS1 | 0.0214542 |  |  |  | 0.0396793852 | -0.6270420176 | -0.5180745336 | -1.337034236 | 0.0544779468 | 0.1832284161 | 0.0254811431 | -0.4082140318 | -0.2756286731 | 0.0398184152 | 0.1155812863 | -0.1118593466 | -0.4699186838 | 0.1489026292 | 0.1816219721 | -0.0236989045 | 0.6698115503 | 0.1508364853 | 1.041233991 | 0.3489782855 | 0.4803059119 | 0.3710018327 | -0.2954138548 | 0.3215406022 | -0.0450683653 | -0.09292884098 | 0.5272374892 | 0.4402574261 |
| TGFB3 | -0.0868728981 | 0.8068996841 | 2.064313932 |  | -0.0063529619 | -0.3398527487 | -0.2852304882 | -0.6773247917 | -1.084011741 | -0.5549595074 | 0.2055081241 | -0.2737391104 | -0.0600621017 | -0.0955499054 | -0.4244268629 | 0.3374834771 | -0.238232991 | -0.2022604133 | -0.1562564038 | -0.2789469125 | -0.5350337203 | -0.01554410952 | -0.2054295996 | 0.01751127157 | 0.1657585284 | -0.05607723226 | 0.787066673 | 0.6627064601 | 0.6873802031 | 0.6411969184 | 0.8008706953 | 0.7288656876 |
| IL12B | -1.018085589 | -0.0180855891 | 0.9819144109 | 1.981914411 | 0.0076743559 | -0.1711599383 | -0.7820993639 | 0.0205302945 | 0.0960394304 | 0.1791533225 | -0.0947412265 | -0.5793656315 | -0.5574635771 | 0.0452893237 | 0.2051366508 | 0.0913636613 | 0.0228471414 | -0.3754296332 | -0.2579640847 | -0.2397941122 |  |  |  |  |  |  |  |  |  |  |  |  |
| GPT2 | 0.8575180274 |  |  |  | -0.1857646817 | 0.3764376906 | 0.7357180566 | -0.1204730442 | -0.484643554 | -0.8458234593 | 0.4422590587 | 0.3704864911 | 0.8811283365 | -0.1785850067 | -1.303127372 | 0.0565423279 | -0.0413012804 | 0.1806930788 | 0.2527425193 | -0.016648577 | -0.6208914063 | 0.003129180532 | 0.4405111772 | 1.078316529 | 0.3657301822 | 0.3285161875 | 0.151473061 | -0.1268712944 | 0.156546693 | 0.4672935049 | -0.08437173161 | 0.5492277402 |
| TSTA3 | 0.4342120575 |  |  |  | -0.0295785726 | 0.0081896072 | 0.1284991555 | -0.3085361561 | -0.1524420786 | -0.2748388432 | 0.0625866519 | -0.7594764166 | 0.199575464 | 0.1763080699 | -0.0530378678 | 0.018827459 | 0.1519961114 | 0.02365667518 | 0.0045033019 | -0.1100322516 | 0.4712153318 | 0.338688053 | 0.04465399792 | 0.8543304753 | 0.9268606228 | 0.5272469853 | -1.133803336 | -1.440299528 | -0.4857000831 | -0.4379263889 | -0.8396941848 | -0.4082966406 |
| CTPS1 | 0.7016727424 |  |  |  | 0.0015366502 | -0.009733492 | 1.345637721 | -0.3413205797 | -0.571521824 | -0.6774637054 | 0.0966666127 | 0.1450202183 | 0.4290096983 | 0.2137854157 | -0.6201443252 | -0.0720590149 | -0.3120139579 |  |  | -0.6394845228 | -0.7696569504 | -1.00715607 | -0.8967172215 | -0.1045489288 | -0.08239015426 | -0.3210822146 | 0.5294046575 | 0.4297096584 | 0.146021787 | -0.06526595744 | 0.5007405884 | 0.02797532606 |
| GJB1 | -0.398713232 |  |  |  | -0.0139452148 | -0.2324510251 | -0.6623303231 | -0.6336112557 | 0.2930538561 | -0.0187707424 | -0.2471464475 | 0.2404805371 | 0.0642247134 | 0.275645272 | -0.7645591778 | -0.0619306256 | 0.0629928498 | 0.1334527316 | 0.098359489 | 0.404053524 | 0.353927746 | 0.4548701426 | -0.4883582678 | 2.245485088 | 0.2763285247 | 0.335784799 | -0.9972278863 | -0.7518235657 | -1.026622458 | -0.7539163587 | -0.9425873629 | -0.7798163955 |
| SFRP1 | -0.2148580015 |  |  |  | 0.3615869622 | -0.5193668345 | -0.6799456031 | -1.132724194 | -0.1484240068 | -0.9880400164 | 0.0156684745 | -0.1401546326 | 0.2866874596 | -0.3789426235 | -1.796612266 | -0.1738979002 | -0.4658014233 | 0.2757621053 | 0.3561893432 | 0.9217654924 | -0.5070935273 | -0.4059710384 | -0.6220139481 | -0.3652006047 | -0.2649278553 | -0.6531038293 | 0.2816585445 | 0.2479270816 | 0.1647546434 | 0.1012442428 | 0.1976625124 | 0.2037342905 |
| SRMS | -0.0720312315 | 0.9279687685 | 1.927968769 | 2.927968769 | -0.0053433814 | -0.0675150675 | 0.049556124 | -0.1103521344 | -0.0720718192 | 0.0203424283 | 0.0110291906 | -0.1106520098 | -0.2280496506 | -0.1782804365 | 0.0330452183 | 0.1143804856 | 0.1734436667 | -0.0671642908 | -0.0636009109 | -0.2437632897 | -0.6390645189 | -0.3743996881 | -0.258728371 | -0.05209969257 | 0.06882190178 | -0.1938952927 | 0.466882728 | -0.1733928089 | 0.447661836 | 0.1894106191 | 0.417311684 | 0.2563609158 |
| ADORA2B | -0.0505978255 | 0.9494021745 | 1.949402175 | 2.949402175 | -0.1288230354 | 0.0449478624 | -0.3474870981 | -0.6918848264 | 0.0489794081 | 0.3469482413 | -0.1976887602 | -0.4164368601 | -0.4108346985 | -0.8491442033 | 0.1105212255 | -0.1773052228 | -0.0435945522 | 0.3119634919 | 0.2875053148 | 0.0174743467 |  |  |  |  |  |  |  |  |  |  |  |  |
| MAPK9 | 0.3166704809 | -0.4700860331 | 2.018378179 | 3.199277695 | 0.141639858 | 0.2226870982 | 0.1743448946 | -0.085778093 | -0.3751051232 | 0.1092874204 | 0.503038559 | 0.5178445185 | 0.0001264025 | 0.0230537467 | 0.1509939795 | 0.4273517378 | 0.6083443177 | -0.07269453439 | 0.007112627 | -0.4984338267 | -0.2701734024 | -0.386724192 | 0.2084407794 | 0.1278414067 | 0.1893700932 | 0.1779934651 | -0.4303591443 | -0.2473059182 | -0.4928278766 | -0.2357081076 | -0.3628090996 | -0.1524351128 |
| SGK3 |  | 1.041501156 | 1.848885523 |  |  |  |  |  |  |  |  |  |  |  |  |  |  |  |  | 0.2217380037 | 0.2971728652 | 0.6139995829 | 0.8626894501 | 0.2119942529 | 0.1800789748 | 0.4007685353 | 0.314948746 | 0.5798822106 | 0.3382214657 | 0.4860863996 | 0.7257797671 | 0.5201747077 |
| PRDX1 | 1.537940041 |  |  |  | 0.1466841054 | -0.5712902343 | 0.61139237 | -0.6354453703 | 0.3310993979 | -0.1162472448 | -0.0258893844 | 1.677058262 | 0.4198939342 | 0.2608098955 | -0.5945475907 | 0.0249880563 | -0.5267715178 | 0.08629684395 | 0.4449523647 | -0.3487650121 |  |  |  |  |  |  |  |  |  |  |  |  |
| ALDH7A1 |  |  |  |  |  |  |  |  |  |  |  |  |  |  |  |  |  | 0.4118947462 | 0.6053747391 | -0.1842218253 | 0.3216594774 | 0.3648261 | 0.3403593429 | 2.169864452 | 1.295301835 | 0.9972101585 | -0.8363731718 | -0.4395380524 | -0.5954267602 | -0.7566447019 | -0.5982701209 | -0.6372856982 |
| AGT | -0.3323966551 | 0.6676033449 | 1.667603345 | 2.667603345 | 0.0879156505 | 0.0538956911 | 0.0951337809 | -0.0438665764 | -0.7683334787 | -0.2804949177 | 0.0250098425 | -0.0079161938 | 0.343011161 | -0.9968878887 | -0.2244315809 | -0.1130156403 | 0.029407338 | 0.3821639617 | 0.6729741579 | -0.1299264568 |  |  |  |  |  |  |  |  |  |  |  |  |
| ITIH2 | 1.053385025 | 0.8915335633 |  |  | 0.2427903826 | -0.0130798647 | 0.5127806173 | -0.0361015431 | 0.2518169492 | 0.0665485292 | 0.1258745023 | -0.13719137 | 0.6914550121 | 0.1340313373 | -0.4447751518 | -0.0088812877 | 0.2627754871 | 0.7208157146 | 0.7938005929 | -0.3954388856 | 0.1525994411 | 0.0423782062 | 0.2150591358 | 1.593809741 | 0.3650462381 | 0.1897432519 | 0.08152395388 | -0.28164286 | -0.1611116922 | 0.05249094444 | -0.09947139572 | -0.1434309363 |
| GABBR2 | 0.9194162345 | 3.555857119 |  |  | 1.115024945 | -0.8688415005 | 0.2049869393 | -0.7074296302 | 0.2148455507 | 0.7244990353 | 0.0886942645 | 0.0634154294 | -0.0834199669 | -0.105620034 | 0.4709482629 | 0.2700051264 | -0.7958114115 |  |  |  | 0.1312893357 | 0.2414323338 | 0.4183629757 | -0.2634913277 | -0.06039447732 | 0.1406159305 | 0.3053708333 | -0.1841768166 | -0.05589630772 | 0.03172781994 | 0.09115871178 | 0.02346622458 |
| CAMK1G | -0.8328457612 | 0.1671542388 | 1.167154239 | 2.167154239 | -0.0453917989 | 0.1974354924 | -0.543355098 | 0.0983936489 | -0.3274378579 | 0.0500559397 | 0.1637734175 | -0.3286934947 | 0.0811146121 | -0.1584311585 | 0.3455182492 | -0.063705814 | 0.2858822505 | -0.06417423331 | -0.0730829552 | -0.100068769 | -0.699844737 | 0.01239670156 | -0.1318696355 | -0.6947143896 | -0.6419336097 | -0.466229427 | 0.07563465609 | 0.7101605906 | 0.5196769969 | 0.6718019936 | 0.4901386534 | 0.4143061084 |
| CAMK2A | -0.0593701179 | 0.9406298821 | 1.940629882 | 2.940629882 | 0.087888311 | -0.0420197599 | 0.2705757713 | -0.0182210504 | 0.8410023304 | 0.5077472742 | 0.1450710976 | 0.0010277403 | 0.4357526702 | 0.1831573898 | 0.6217902114 | -0.018087963 | 0.0103287497 | 0.03497276306 | 0.0546886319 | -0.3673347073 | 0.147097141 | 0.4195678464 | 0.1711842989 | 0.3675180833 | -0.06494143943 | 0.07987780689 | -0.6964110225 | -0.4560568558 | -0.7918677142 | -0.702233257 | -0.4984364362 | -0.121535881 |
| MAPKAPK2 | 0.0114922086 |  |  |  | -0.0157219633 | 1.153969709 | 0.0851663408 | -0.0185463102 | 1.404943459 | 0.7105345408 | -0.078124062 | -0.6887648747 | -0.0134420283 | 0.4918783177 | 0.2492419229 | -0.1583685765 | 0.6742612009 | -0.1157607737 | -0.1440413518 | 0.3783275055 |  |  |  |  |  |  |  |  |  |  |  |  |
| UCK2 | 0.401767378 | 0.9303991995 |  |  | -0.0324221595 | -0.029983529 | 0.6813763436 | -0.2720826734 | 0.186323816 | -0.0694040787 | -0.0300457231 | 0.2159943674 | 0.3734694484 | 0.3207215946 | -0.2622241588 | -0.1338312137 | -0.1185750694 |  |  |  | 0.8844782295 | 0.09700444511 | 0.8215328043 | 1.464567096 | 1.194363381 | 0.9455000635 | -0.2084653939 | -0.8901746411 | -0.9885691053 | -0.652204718 | -0.5230534177 | -0.653251502 |
| DMC1 | 1.979021239 |  |  |  | 0.0538969257 | 0.1533046541 | 0.9251014028 | 0.091641551 | 0.5465949129 | 0.9856024996 | 0.0260388248 | -0.2915384692 | -0.3467930335 | 0.1383732835 | 1.44860751 | 0.0214565652 | 0.2708618292 | -0.1086357045 | -0.0221735689 |  | 0.1732224197 | 0.2789150407 | -0.215850568 | -0.1424356304 | -0.06619197437 | -0.2779999322 | -0.05293615951 | -0.1781497554 | -0.1427654086 | 0.1335015643 | 0.637032191 | 0.1111939624 |
| SLC16A1 | 1.094514607 |  |  |  | -0.0337412002 | -0.4073877359 | 0.7886722862 | -0.7130162647 | -0.0969858024 | -0.1795600212 | -0.1372183065 | 2.411386166 | 0.2508125256 | 0.0241248092 | -0.4089239138 | -0.2248986022 | 0.0357164287 | 0.9040126272 | 0.7677058391 | 0.2681491759 |  |  |  |  |  |  |  |  |  |  |  |  |
| ACAD8 | -0.0920402819 |  |  |  | -0.081213134 | 1.449462241 | 0.0317676198 | 0.4213660927 | 0.5476458027 | 0.6820875527 | 1.134781757 | 0.1496234085 | -0.3015792861 | 0.2819160966 | 0.7418669989 | 0.385837312 | 0.6092948089 | 0.3957262606 | 0.3062298626 | -0.0308917843 |  |  |  |  |  |  |  |  |  |  |  |  |
| ATOX1 | 0.3780109189 |  |  |  | -0.1446428263 | 0.8292301237 | 0.0114546681 | 0.411191934 | 1.383619472 | 1.068330012 | 0.3067079711 | 0.0344874425 | -0.5030678018 | 0.4854247922 | 0.7641277627 | 0.1534065593 | 0.0381459557 | 0.5949261403 | 0.5393489723 | 0.0671756142 |  |  |  |  |  |  |  |  |  |  |  |  |
| RCAN1 | 1.565607423 |  |  |  | 0.1881983545 | 0.6612306969 | 0.7303980542 | 0.4320660508 | 0.4229914608 | -0.1448502225 | 0.060812122 | 1.04225414 | 0.8884831713 | 0.1248700257 | -0.9122398401 | 0.1104212822 | 0.0329184477 |  |  |  |  |  |  |  |  |  |  |  |  |  |  |  |
| MMP26 |  | 1.502800229 | 2.691322185 |  |  |  |  |  |  |  |  |  |  |  |  |  |  | -0.1239814014 | 0.0117076214 |  |  |  |  |  |  |  |  |  |  |  |  |  |
| ESRRB | -0.7412549285 | 0.2587450715 | 1.258745072 | 2.258745072 | 0.141401833 | -0.0801253316 | -0.3003475908 | -0.2024387127 | -0.2883110149 | -0.0382815139 | -0.0951420871 | -0.015279788 | -0.0112864292 | -0.3957087094 | 0.0498592233 | -0.0015323311 | 0.0842338999 | 0.08911386875 | 0.0971990941 | 0.0460009773 | 0.1214552187 | -0.1460580551 | 0.07532071627 | 0.1040654985 | 0.2687243815 | 0.04305336018 | 0.4433490679 | -0.05975884753 | -0.02317133823 | -0.04606890066 | 0.3406421684 | -0.0167702165 |
| PDE6C | -0.0963729686 | 0.9036270314 | 1.903627031 | 2.903627031 | 0.0984526422 | 0.0578939909 | -0.1455798963 | -0.0801459998 | -0.1529070374 | 0.4047614085 | 0.0357405405 | 0.0300182117 | -0.1511909581 | 0.2925373841 | 0.5213303875 | 0.0176497554 | 0.0649568017 | -0.06719996374 | -0.0825205455 | -0.4802101255 | 0.3699942502 | 0.3957022806 | 0.07027970285 | 0.02110379353 | -0.1618660329 | 0.02463128666 | -0.03742127467 | 0.07441906699 | 0.09009847746 | -0.07275067177 | 0.2224204863 | 0.04207285599 |
| PDE8B | -0.1479182441 | 0.8520817559 | 1.852081756 | 2.852081756 | 0.1160834167 | -0.1939809252 | 0.2499146144 | -0.168782884 | -0.109629637 | 0.2257242616 | -0.1105544757 | -0.1788346012 | -0.5026243729 | -0.0882762947 | 0.3326249783 | -0.0960069395 | -0.567307668 | 0.125875335 | 0.15627407 |  | -0.08690502776 | -0.2114580642 | 0.3817128378 | 0.1645755739 | 0.1634439122 | 0.2334933044 | -0.1053707034 | -0.2887528487 | -0.2208650812 | 0.01919732431 | -0.4007478486 | -0.1024514047 |
| PCCA | -0.1471479029 | 0.8528520971 | 1.852852097 | 2.852852097 | 0.0339197935 | -0.0771410684 | 0.3665854545 | -0.3292776624 | -0.6436243703 | -0.2887098842 | 0.1694755987 | -0.7830175606 | -0.1628865842 | -0.1540471451 | 0.031128593 | 0.1660103865 | 0.4747914064 | 0.3344745769 | 0.4038055467 | -0.5401218272 | 0.08889504069 | -0.3675446115 | 0.2374948938 | 0.7641745379 | 0.6406050666 | 0.3024144441 | -0.5850038475 | -0.3122312608 | -0.319287093 | -0.2262107427 | -0.07834316575 | -0.582503179 |
| RPS6KB1 | 0.4639301024 |  |  |  | 0.0384903441 |  | 0.5802485218 |  | -0.1748859303 | -0.5012661524 | 0.2541833769 |  | 0.5242155339 | 0.0227176295 | -0.7075981074 | 0.1206170489 |  | 0.3316901476 | 0.797949386 | 0.1449585811 | -0.659485402 | -0.586932483 | -0.3816601676 | -0.4549259178 | -0.5004178613 | -0.7991554007 | 1.153800232 | 0.5703598661 | 0.8223668753 | 0.3460463606 | 1.182549856 | 0.4883756232 |
| MAP2K7 | -0.4455287223 | 1.903208951 |  | 3.06722644 | 0.0800903917 | -0.5586859602 | -0.3933734773 | -0.8971941046 | 0.8694260339 | 0.6874394334 | 0.036234257 | 0.4497226631 | 0.0913351271 | 0.4654054857 | 0.9429544317 | 0.0461146986 | -0.3310721771 | -0.1162154887 | -0.1994835425 | 0.886304885 | -0.1394294173 | -0.006438856716 | 0.09322605703 | 0.1429128685 | 0.2111572666 | 0.2263897403 | 0.08384419138 | 0.4350709042 | -0.07585357027 | 0.1292461981 | 0.08097803588 | 0.3742646271 |
| PIKFYVE | -0.3083638147 | 0.6916361853 | 1.691636185 | 2.691636185 | 0.2201270508 | -0.1070315244 | -0.0377765283 | -0.1984465112 | -0.3081499272 | -0.0544189634 | 0.0169189625 | -0.7993538494 | 0.7886127585 | 0.3897521437 | 0.1191716675 | -0.0520819857 | 0.0454807685 |  |  |  |  |  |  |  |  |  |  |  |  |  |  |  |
| MAP4K1 | 0.2407980179 |  |  |  | 0.9402257058 | -1.088862263 | -0.6461158552 | -0.4020949272 | -0.9009160581 | -0.5829832194 | -0.1386631177 | -0.0639061369 | -0.4806146198 | 0.1099068254 | 0.1191078275 | -0.0009318329 | -0.2789123854 | -0.4733381655 | -0.2365075973 | -0.7647714642 |  |  |  |  |  |  |  |  |  |  |  |  |
| PLK1 | 0.4244202692 |  |  |  | -0.0330722591 | -0.0362249251 | 0.7031918586 | -0.733449645 | 0.751958558 | -0.3738603794 | 0.4295975646 | 0.1424233106 | 0.7978645488 | 0.3046432255 | -1.059893665 | 0.2314304222 | -0.0517448917 |  |  | -0.7284268887 | 0.1015741607 | 0.06391213437 | 0.5789671838 | 0.3224232648 | 0.3489909702 | 0.5043616645 | -0.2194912782 | -0.02567600366 | -0.1471066326 | -0.05990742745 | -0.2398693211 | 0.3431926547 |
| CPB1 | -1.22873584 |  |  |  | 0.0186479805 | -0.0030028574 | -0.5588868104 | -0.2494171447 | -0.4245262771 | -0.1062970971 | -0.0153280091 | -0.3010909386 | -0.3222191367 | -0.4212287065 | -0.2011938792 | -0.0078662693 | -0.128457533 | -0.1116880828 | -0.0514269436 |  | -0.07763896719 | 0.1148415103 | 0.1001159876 | 0.1223104942 | 0.2239719816 | 0.192926016 | 0.1920944409 | -0.1602240897 | -0.02945328581 | -0.1132326112 | 0.002239805282 | -0.1548334883 |
| CDK1 | 0.9768677938 |  |  |  | -0.0545543492 | 0.1847053072 | -0.6486433346 | -1.269465474 | 0.3400750581 | 0.6760567913 | 0.1863460464 | 0.3891981237 | -0.6478736207 | -0.0911701173 | 0.0977538583 | -0.0167649964 | -0.0255777804 |  |  |  |  |  |  |  |  |  |  |  |  |  |  |  |
| STK35 | -0.1981956444 |  | 1.040049423 |  | 0.5627415557 | 0.1438673552 | -0.4886111004 | 0.0511670134 | 0.1380319911 | 0.2482694023 | -0.0535137211 | 0.1391043565 | -0.2417254856 | -0.0062061669 | 0.4348700347 | 0.1703380257 | -0.428251777 | 0.01683083512 | -0.0332181958 | 0.0761448411 | -0.6796064629 | -0.6985952691 | -0.1918025976 | 0.1204585515 | 0.4900876784 | 0.269303238 | 0.7916013437 | 0.2051591252 | 0.7231743959 | 0.1982634574 | 0.7354809857 | 0.03508155104 |
| TLK2 | -0.0765008793 | 1.128818249 |  |  | 0.0584209162 | 0.1246504197 | 0.0186559229 | -0.2616020081 | -0.419365152 | -0.1727263235 | 0.0279157257 | -0.1931210722 | 0.0216353658 | -0.0359957526 | 0.1445330565 | 0.3003215935 | 0.326848467 | -0.1770990564 | -0.2001417967 | 0.1152290075 | 0.0194530525 | 0.5610416173 | -0.09894851017 | 0.02730117658 | 0.2369846484 | 0.09883130039 | 0.02798100737 | 0.3956446456 | 0.09314891124 | 0.0829241931 | 0.6398482749 | 0.1265642919 |
| RIOK1 | 1.184641357 |  |  |  | -0.0075795646 | -0.4996543064 | 0.1192149159 | 0.1091091326 | -0.0325203366 | -0.2134702585 | -0.2745428319 | -0.0954626079 | 0.2108169646 | 0.2134720495 | -0.4301467075 | 0.0430146715 | -0.0969960354 |  |  | -0.3466308099 | -0.6677147572 | -0.7583867451 | -0.509440899 | -0.1684176393 | 0.1902687846 | -0.2856225817 | -0.4359696235 | -0.5112152861 | -0.3743568515 | -0.7487500733 | -0.689404018 | -0.7641542938 |
| CYP1A2 | 0.1117089974 | 0.5858190645 |  |  | 0.0086466237 | 0.0941524693 | 0.0109424956 | 0.5613962012 | -0.5939513471 | -0.0663574004 | 0.0033365285 | 0.0418202007 | -0.2169892811 | -0.9866394187 | -0.2111403933 | -0.0188357604 | -0.050330887 | -0.3421745154 | -0.4328189557 | -0.6324612949 | -0.04763374164 | -0.1046611227 | 0.004719086729 | 1.153706221 | -0.07166840631 | -0.2369697286 | 0.1963088616 | 0.3220705089 | 0.2551333254 | 0.1800694418 | 0.03026890494 | 0.0845579689 |
| HSPA8 | 0.0235826158 |  |  | 3.413582368 | -0.1285075551 |  | 0.2260315748 |  | -0.2127936273 | -0.0954827685 | -0.0642507351 |  | 0.3654659699 | -0.5405427777 | -0.5583775452 | -0.3258610638 |  | 0.6189376555 | 0.8612752803 |  |  |  |  |  |  |  |  |  |  |  |  |  |
| EXTL2 | 0.6888708759 | 1.928489972 |  |  | -0.0716435802 | 0.2840239652 | 0.6429022006 | -0.6259783115 | 0.2036126482 | -0.0392054982 | 0.3554268381 | 0.9177193383 | 0.7485751505 | 0.114898431 | -0.4022841289 | 0.1505081828 | -0.0913222242 | 0.2243105349 | 0.3104156991 | 0.1719526676 | -0.1842260498 | -0.2791318832 | 0.1907468889 | 0.6239635101 | 0.8051247384 | 0.6807564548 | 0.6955629793 | -0.1070592673 | -0.06565512553 | 0.1405640752 | -0.1642984321 | -0.05984375037 |
| ITPR1 | 1.073853294 |  |  |  | 0.0883789188 | -0.0887526979 | -0.1596265946 | 0.2333420518 | -0.0857959823 | 0.5385978325 | 0.1478732913 | 0.1776963553 | 0.0701531 | -0.1205528169 | 0.5999505046 | 0.18022038 | -0.025732815 | 0.3238973579 | 0.2255785552 | 0.1756262477 |  |  |  |  |  |  |  |  |  |  |  |  |
| NMNAT3 | 0.5561868154 | -0.3986774548 | 1.9672687 |  | -0.1355075922 | 0.2278345224 | 0.3532294705 | 0.0433589376 | 0.3149245504 | -0.0360980236 | 0.5673963632 | -0.3565795017 | 0.3334581801 | 0.6870604337 | -0.0846393734 | -0.0300404662 | 0.3715843107 |  |  |  | -0.3238872413 | -0.7784991707 | -0.1297788518 | 0.1620034634 | 0.2513682798 | 0.3914729921 | 0.1291796315 | 0.3271835316 | 0.1734497508 | 0.03811934777 | 0.3547720132 | 0.006994566781 |
| FABP1 |  |  |  |  |  |  |  |  |  |  |  |  |  |  |  |  |  | 0.2853013422 | 0.4984663805 | 0.3322752007 | -0.0004198509109 | -0.1617178275 | -0.00002684911022 | 2.489454549 | -0.06107899851 | -0.09787311995 | -0.0897137402 | 0.3605474883 | -0.1715182647 | -0.2617919855 | 0.2985002047 | -0.2167719941 |
| SERPINA3 | 0.7385522819 |  | 1.697525399 |  | 0.1748572461 | -0.0047931229 | -0.002351646 | 0.4007621719 | 0.0239927397 | 0.3656525322 | 0.1133168516 | 0.8540173955 | 0.2701199178 | 0.0768679372 | 0.3672270663 | 0.3392016904 | 0.2585932963 | 0.07517274623 | 0.5317975812 | 0.1292941195 | -0.2470592384 | -0.4324242875 | -0.2502732271 | 0.6678454505 | -0.3383832295 | -0.2796088136 | -0.05648765346 | 0.01319451006 | -0.130946831 | 0.06501929687 | -0.1682451116 | -0.1285122787 |
| CYP2J2 | 1.455746211 |  |  |  | 0.4819073607 | 0.0325166103 | 0.659837448 | 0.4010057183 | 0.3821292282 | 0.3413529176 | 0.0962747175 | 0.6028493801 | 0.7561346131 | 0.0900585247 | 0.3104193842 | 0.1139204959 | 0.4986653279 | 0.2536550134 | 0.259712285 | -0.0185475738 | -0.07482648976 | 0.4675242954 | 0.2674329205 | 0.1472599307 | 0.188017408 | 0.1268942117 | 0.01491011504 | -0.1496001549 | 0.2020707715 | -0.008437948951 | 0.03159148435 | 0.1775326461 |
| CA12 | 0.5292580464 | 0.8650705441 |  | 4.163742488 | 0.0166881845 | -0.2356102299 | 0.7309103692 | -0.6048865554 | -0.1375071558 | -0.5276462635 | 0.2173954695 | -0.5152136413 | 0.4527822362 | -0.0019313511 | -0.8425473828 | -0.3869459204 | -0.4001421763 | 0.4123951289 | 0.1641752374 | -0.5797564887 |  |  |  |  |  |  |  |  |  |  |  |  |
| SLC10A2 | -0.5780805871 | 0.7396743023 |  |  | 0.0111990198 | -0.0364256156 | -0.4667847447 | -0.0927653229 | 0.1131658918 | 0.4039841824 | 0.1888242277 | 2.295312473 | -0.5324065444 | -0.1287776152 | 0.4317521786 | 0.1042203041 | 0.3031764599 | -0.1042324164 | -0.1421211961 | 0.3994931495 | -0.004360869086 | -0.1683508926 | -0.05088032547 | -0.4903444176 | -0.628920619 | -0.5269815624 | 0.8587629236 | 0.3492935949 | 0.642469645 | 0.2849832697 | 0.1467300751 | 0.3592534145 |
| PYCR3 |  |  | 2.668966604 |  |  |  |  |  |  |  |  |  |  |  |  |  |  |  |  | -0.3445531055 | -0.6503754839 | -0.9465714652 | -0.2618982946 | 0.09049102777 | 0.09660946064 | -0.1012004097 | -0.08124050108 | 0.5601913695 | -0.1667168761 | 0.1035891715 | -0.09013514869 | 0.2097012285 |
| IGSF10 | -0.4180986267 | 0.5181701709 | 2.46820325 |  | 0.2637690163 | 0.6244905686 | -0.1521179311 | 0.9307778882 | 0.3494965823 | 0.0376412597 | -0.0914351594 | 0.2517187193 | -0.0409201952 | 0.0502762986 | -0.133031113 | -0.0720175694 | 0.3318677586 |  |  |  | -0.1700031169 | 0.6266239314 | 0.09020674286 | 0.08085224609 | 0.119631158 | 0.4011210206 | 0.5922110543 | 0.2406143391 | 0.1102070863 | -0.01417295509 | 0.07577691331 | -0.3109610006 |
| MYH2 | 0.1554646088 |  |  |  | 0.455776523 | 0.2196080317 | 0.8008123205 | 0.2871434785 | 1.556946837 | 0.1185584539 | 0.1935153389 | 0.1621608205 | 0.6580383524 | 0.8214795844 | -0.0550709833 | 0.0554764092 | 0.0209336322 | -0.02256381438 | 0.0116659115 |  |  |  |  |  |  |  |  |  |  |  |  |  |
| CDC7 | 0.2426747979 | 1.568168196 |  |  | 0.1902580616 | 0.3230515815 | 0.6040792209 | -0.0982510184 | 0.5413100399 | 0.1492352041 | 0.336134271 | 0.6524773792 | 0.1360611159 | 0.2719526154 | 0.3938370533 | 0.2451053391 | 0.2448884458 |  |  | -0.8130561147 | 0.132348949 | -0.1826285943 | 0.2489783018 | -0.1085878254 | -0.001578211849 | -0.2307149933 | 0.02029220345 | -0.06630217066 | 0.08619743325 | -0.1990668937 | -0.02216883708 | 0.4657200626 |
| UCHL5 | 0.7803818314 |  | 3.188400979 |  | 0.2005081946 | 0.5612426579 | 0.2109885246 | -0.4396397697 | 0.140588232 | 0.0909813004 | -0.1045349061 | -0.0663650051 | -0.1436390869 | 0.0848947535 | -0.1769144273 | 0.0375532849 | 0.8784467758 | -0.02015570674 | 0.1066583384 | 0.0374219906 | -0.08354453604 | -0.3184808386 | -0.2603685413 | 0.413147629 | 0.5396714234 | 0.309627903 | -0.6277153625 | -0.561606268 | -0.5522710043 | -0.5272584511 | -0.5902640631 | -0.4942769081 |
| RNASE2 |  | 0.5391325847 |  |  |  |  |  |  |  |  |  |  |  |  |  |  |  | -0.04152481816 | -0.1549896932 |  |  |  |  |  |  |  |  |  |  |  |  |  |
| MR1 | 0.4246370441 |  |  |  | -0.0113136226 | -0.1026023066 | 0.0340275932 | -0.2226335305 | -0.2992425122 | -0.1679949792 | 0.3638131892 | -0.5714180922 | 0.0673067121 | 0.1524497997 | -0.0335706137 | 0.0141384968 | 0.0822630483 |  |  | -0.5410321742 |  |  |  |  |  |  |  |  |  |  |  |  |
| FCGR2A |  |  |  |  |  |  |  |  |  |  |  |  |  |  |  |  |  | -0.0241593955 | -0.0830674233 | 0.1335837582 |  |  |  |  |  |  |  |  |  |  |  |  |
| UCHL1 | -0.3355743229 |  |  |  | -0.1929123822 | -0.859099662 | -0.9078135038 | -0.1414700975 | 0.1308755421 | 0.7878168446 | 0.1871200662 | 0.6281000403 | -0.4112384635 | -0.2275365359 | 0.7907932405 | -0.1072851544 | -0.6845023348 | 0.0378500165 | 0.2525352022 | 0.458233022 |  |  |  |  |  |  |  |  |  |  |  |  |
| SLC47A1 | 0.5700490192 |  |  |  | 0.0883965853 | 0.4505342398 | 0.0845373745 | 0.040974647 | 0.1972052903 | -0.0261706099 | 0.1330873416 | 0.1987451547 | 0.3359200686 | -0.3704029259 | -0.6684111493 | 0.230193401 | 0.2694491923 |  |  |  |  |  |  |  |  |  |  |  |  |  |  |  |
| MT1E |  |  |  |  |  |  |  |  |  |  |  |  |  |  |  |  |  | 0.001228934577 | -0.0655052724 |  |  |  |  |  |  |  |  |  |  |  |  |  |
| CLCN2 | 0.0411785414 | 0.9024942604 |  |  | 0.1237949918 | 0.0504664147 | 0.146222687 | -0.4556603136 | -0.024187988 | 0.0597910923 | 0.4551586815 | -0.006059814 | 0.0334901227 | 0.3246175702 | 0.1183696648 | 0.3882817281 | 0.2603263883 | 0.5354160467 | 0.5910718136 | -0.6858717329 | 0.2613148224 | 0.1639380623 | 0.4296560673 | 0.6055035197 | 0.2386400251 | 0.08950297087 | -0.1734223425 | 0.06079349615 | -0.1222944274 | 0.1483814699 | -0.1217702432 | 0.2096536248 |
| SAT2 |  | 0.4355366848 |  |  |  |  |  |  |  |  |  |  |  |  |  |  |  |  |  |  | 0.2097103714 | -0.1417442023 | -0.08276089262 | 0.193728994 | 0.157388043 | 0.3467538191 | -0.1193894945 | 0.04654692254 | -0.179553132 | -0.1053211742 | 0.01525030592 | -0.2489843755 |
| UGT2B7 |  |  |  |  |  |  |  |  |  |  |  |  |  |  |  |  |  | 0.299986492 | 0.2852244365 |  |  |  |  |  |  |  |  |  |  |  |  |  |
| CPN1 | 0.7591289293 |  |  |  | 0.0737661613 | 0.0328179306 | 0.5849968402 | 0.4212489454 | -0.4815675747 | -0.3910564249 | 0.0912935077 | -0.5975756082 | 0.2042776021 | -0.8224177656 | -0.7974429335 | 0.0197399554 | 0.3943370369 | 0.2470524672 | 0.2645446378 | -0.6651397722 | -0.6154788461 | -0.671167464 | -0.7837882576 | 0.3673941819 | -0.297495102 | -0.6696246864 | -0.2313064557 | 0.0345888567 | 0.02721578964 | -0.003860831267 | -0.3641923129 | 0.08605946854 |
| AURKC |  |  |  |  |  |  |  |  |  |  |  |  |  |  |  |  |  |  |  |  | -0.05908975833 | 0.1851457105 | -0.3527321643 | -0.1861171979 | -0.04700174832 | -0.1088378002 | -0.4479073256 | 0.6032791173 | -0.4673711024 | -0.3486975669 | 0.009659821628 | 0.4200941293 |
| DDR1 | 0.5478333671 |  |  |  | -0.0271291945 | 0.0966573217 | 0.3334691804 | 0.1192875081 | -0.1734203229 | 0.5352313151 | -0.0946422613 | 0.9369555462 | 0.0559004312 | -0.1404025082 | 0.314068153 | -0.1877173809 | 0.0739477411 | 0.649792248 | 0.6473524183 |  |  |  |  |  |  |  |  |  |  |  |  |  |
| STK17A |  |  |  |  |  |  |  |  |  |  |  |  |  |  |  |  |  | -0.08532408651 | -0.0956790951 |  |  |  |  |  |  |  |  |  |  |  |  |  |
| PKMYT1 | -0.577919642 |  |  |  | -0.0306083938 | -0.6473569472 | -0.1782279096 | -0.6927310086 | -0.5984302223 | -0.1435882661 | 0.508950371 | -0.2222308524 | -0.1945821286 | 0.1264044182 | 0.5486641894 | 0.2503104424 | -0.3214887674 |  |  |  | -0.3014793424 | 0.09688380873 | 0.2988515151 | 0.4314429532 | 0.8428975704 | 0.8326278343 | -0.4769841716 | -0.4255423535 | -0.6075555147 | -0.4669016554 | 0.2311914482 | -0.3039838982 |
| SLC19A1 | -0.4316515516 |  |  |  | 0.008478399 | -0.4037134431 | -0.203370537 | -0.4310383065 | -0.4088774254 | 0.2148300932 | 0.093855714 | -0.4788205348 | -0.2540831345 | -0.1563765895 | 0.4359974647 | 0.0330108439 | -0.212719291 | -0.09148021552 | -0.1087212873 | -0.4653690496 | -0.110320141 | -0.2332051138 | -0.1307660304 | 0.03709663869 | 0.09668043956 | -0.01265904816 | -0.4731682748 | -0.3059165762 | -0.4934630651 | -0.3951988005 | -0.4780681705 | -0.0931123828 |
| H2BC4; H2BC6; H2BC7; H2BC8; H2BC10 |  |  |  |  |  |  |  |  |  |  |  |  |  |  |  |  |  |  |  |  |  |  |  |  |  |  |  |  |  |  |  |  |
| SIK1 |  |  |  |  |  |  |  |  |  |  |  |  |  |  |  |  |  |  |  |  |  |  |  |  |  |  |  |  |  |  |  |  |
| IMPG2 | -0.4375943101 | 0.4201821699 |  |  | -0.1399169109 | 0.1020969068 | -0.2039507011 | -0.315719649 | 0.4605314152 | 0.4362287404 | 0.0680970907 | -0.1628244891 | -0.6916218227 | 0.3372765893 | 0.7615518118 | 0.1248351226 | 0.1321361194 | -0.2350264298 | -0.0625695273 | -0.0585504183 |  |  |  |  |  |  |  |  |  |  |  |  |
| UQCRH |  |  |  |  |  |  |  |  |  |  |  |  |  |  |  |  |  | 0.2526280752 | 0.2612485973 |  |  |  |  |  |  |  |  |  |  |  |  |  |
| GAPDH |  |  |  |  |  |  |  |  |  |  |  |  |  |  |  |  |  |  |  |  | -0.05645852998 | 0.0582536913 | 0.1895948206 | -0.2887099043 | 0.08171229107 | 0.4695633365 | 0.3909851694 | 0.7930100335 | 0.8066838049 | 0.823408078 | 0.3429397868 | 0.6327389715 |
| CELA2A |  |  |  |  |  |  |  |  |  |  |  |  |  |  |  |  |  |  |  |  |  |  |  |  |  |  |  |  |  |  |  |  |
| AKR1C2 |  |  |  |  |  |  |  |  |  |  |  |  |  |  |  |  |  | 0.129526611 | 0.0878911218 |  |  |  |  |  |  |  |  |  |  |  |  |  |
| NR3C2 | 0.1919077461 | 0.2100926599 |  |  | 0.1293836918 | 0.0315876752 | 0.0216659579 | 0.1564955658 | -0.0332951985 | -0.1500546279 | 0.036529069 | 0.1787364048 | 0.1032769726 | 0.0660457953 | -0.0725789398 | -0.0554607484 | 0.0464936134 | 0.1589118095 | 0.1859075431 |  |  |  |  |  |  |  |  |  |  |  |  |  |
| LGALS9 |  |  |  |  |  |  |  |  |  |  |  |  |  |  |  |  |  | -0.1802547809 | -0.2209347153 |  |  |  |  |  |  |  |  |  |  |  |  |  |
| HLA-A |  |  |  |  |  |  |  |  |  |  |  |  |  |  |  |  |  | 0.3263189537 | 0.2685310096 |  |  |  |  |  |  |  |  |  |  |  |  |  |
| EPRS1 |  |  |  |  |  |  |  |  |  |  |  |  |  |  |  |  |  |  |  |  |  |  |  |  |  |  |  |  |  |  |  |  |
| HCN2 | -0.117098387 | 0.7702887763 |  |  | -0.0628429693 | 0.334494698 | 0.020515941 | -0.0517445234 | -0.7868178452 | 0.0391571097 | -0.1475516915 | -0.0510589641 | -0.5143646131 | -0.4965140537 | 0.2413397757 | -0.0653169 | 0.3292786653 | -0.324952124 | -0.0576022371 | 0.126913205 | -0.07269598893 | -0.02896620763 | -0.03680388627 | -0.2472936927 | -0.6108998165 | -0.2676770198 | -0.2740812585 | -0.2198392668 | -0.2939341709 | -0.2620239705 | -0.5350563209 | -0.1940889642 |
| DRD2 | -0.0507270509 | 0.773093558 |  |  | 0.0307078187 | -0.2599522277 | -0.8424644299 | 0.0516500124 | -0.4018066921 | -0.37905421 | -0.0241184077 | -0.2041858286 | -0.0793405996 | 0.0230075843 | -0.9157237026 | 0.0518560738 | 0.0713907377 | -0.1625110093 | -0.3145434682 |  | -0.2478124269 | 0.09953575076 | -0.1383934394 | 0.1255354361 | -0.08911423192 | -0.127360693 | -0.2935707954 | -0.1928770729 | -0.05473473685 | -0.3403357278 | -0.158872518 | -0.2163500489 |
| GPER1 |  |  |  |  |  |  |  |  |  |  |  |  |  |  |  |  |  |  |  |  |  |  |  |  |  |  |  |  |  |  |  |  |
| GABRB1 | -0.0506481271 |  |  |  | 0.1182059886 | 0.2715722848 | 0.2565762168 | 0.4373672698 | -0.5279422929 | -0.000822744 | -0.00902424 | 0.194179015 | -0.5581275444 | -0.2485487709 | 0.0333730424 | 0.0444120295 | 0.0870412005 | 0.215147776 | 0.1516065702 |  | -0.06152343829 | 0.08709493447 | 0.2057603716 | 0.2032523629 | 0.1278477156 | 0.1054246087 | -0.03183487086 | -0.07199662144 | 0.1443631377 | -0.05596320785 | 0.1163338052 | -0.02998083047 |
| GABRB2 | -0.5421835765 | 0.9493518729 |  |  | 0.0139258925 | -0.0109024091 | -0.9847261858 | -0.0034490261 | 0.1499105313 | 0.3260696308 | -0.0149137659 | 0.0257699831 | -0.617553223 | -0.3795305873 | 0.7045258142 | 0.0419437165 | -0.0690626897 | 0.2228018448 | 0.2092229555 | -0.0171360662 | -0.1588412671 | -0.2375360303 | 0.01005605604 | -0.3854242635 | -0.1886720317 | 0.07016413097 | 0.104842494 | 0.7867979686 | 0.2018084711 | -0.07966680752 | 0.7616674024 | 0.8468556336 |
| TUBG1 |  |  |  |  |  |  |  |  |  |  |  |  |  |  |  |  |  | 0.2434908214 | 0.1999027262 |  | -0.4137137662 | -0.0786549435 | 0.616896734 | 0.1327286724 | 0.39556363 | 0.1902719014 | -0.2799842855 | -0.5713252717 | -0.7243446221 | -0.7971624718 | -0.921506899 | -0.6561113173 |
| SLC6A1 | 0.8840007377 |  |  |  | 0.0492899111 | -0.156555494 | 0.6261701419 | -0.0170435779 | 0.4786340658 | 0.2339530724 | 0.1042803769 | -0.2002609783 | 0.3312640851 | 0.2436434081 | 0.3687742145 | 0.1437241803 | 0.0274096629 | -0.1204314799 | -0.1099431297 | -0.1888365824 | 0.6997921136 | -0.1658165263 | -0.03780279184 | -0.1594499474 | -0.04395886964 | 0.04371931173 | -0.2122525867 | -0.05057970303 | -0.06203425688 | 0.1125629006 | 0.3765784784 | -0.01162361426 |
| SLC22A11 |  |  |  |  |  |  |  |  |  |  |  |  |  |  |  |  |  | -0.3007120695 | -0.1722806568 |  |  |  |  |  |  |  |  |  |  |  |  |  |
| NARS2 | 0.6856466355 |  |  |  | -0.1526648783 | 0.1183817408 | 0.6838750724 | -0.2571286353 | -0.2342281285 | -0.0156179468 | 0.1058768814 | -0.0307929692 | 0.2633272049 | 0.1779769109 | 0.0038875993 | 0.214235451 | 0.3386179823 |  |  |  |  |  |  |  |  |  |  |  |  |  |  |  |
| GRK2 |  | 0.1815134828 |  |  |  |  |  |  |  |  |  |  |  |  |  |  |  |  |  |  |  |  |  |  |  |  |  |  |  |  |  |  |
| AARS1 |  |  |  |  |  |  |  |  |  |  |  |  |  |  |  |  |  |  |  |  |  |  |  |  |  |  |  |  |  |  |  |  |
| DDAH1 | 0.4137546787 |  |  |  | -0.0737647869 | 0.3204134595 | 0.4457979635 | -0.2046612849 | 0.5669042001 | -0.1791410753 | 0.4913083453 | 0.2915940104 | 0.5605917878 | 0.3481324971 | -0.5768136016 | 0.141689106 | -0.0947476211 |  |  | -0.3527064367 | -0.06419959327 | 0.1427543261 | -0.0521147305 | 0.2476858084 | 0.2564000806 | 0.4508287804 | 0.09722651073 | 0.2467722907 | 0.1658899529 | 0.157844241 | 0.2751866869 | 0.2013119745 |
| CARS1 |  |  |  |  |  |  |  |  |  |  |  |  |  |  |  |  |  |  |  |  |  |  |  |  |  |  |  |  |  |  |  |  |
| GARS1 |  |  |  |  |  |  |  |  |  |  |  |  |  |  |  |  |  |  |  |  |  |  |  |  |  |  |  |  |  |  |  |  |
| GPX2 | 0.3964248576 |  |  |  | -0.118377262 | -0.5018718886 | 0.4508772717 | 0.3725236166 | 0.7879422393 | 0.3196770982 | 0.1026296843 | -0.6723816205 | 0.4044373177 | 0.7946536301 | 0.333359862 | 0.4841193521 | 0.1008030085 | 0.3204278227 | 0.4299474466 | -0.187866676 | 0.02119331332 | 0.3707107309 | -0.1676851478 | -0.01237863968 | 0.732902364 | -0.159585096 | -0.1374783501 | -0.516207157 | -0.465486691 | -0.6872934159 | -0.5501746744 | -0.6978031444 |
| GSTA4 |  |  |  |  |  |  |  |  |  |  |  |  |  |  |  |  |  | -0.02835579918 | -0.0994933756 |  |  |  |  |  |  |  |  |  |  |  |  |  |
| FARS2 | 0.2014736689 |  |  |  | 0.046652143 | -0.1084387967 | -0.2043795312 | -0.3640869648 | -0.0934370779 | 0.1712668931 | -0.0315226849 | -0.1765909437 | -0.0948531362 | 0.4152681189 | 0.6807277879 | 0.0913748565 | 0.1245709675 |  |  |  | 0.07812101595 | 0.01623163183 | 0.3090931045 | 0.1590556324 | 0.2920617731 | 0.1938923205 | 0.3548863784 | 0.190323321 | 0.106322581 | -0.09629712594 | 0.4363120798 | -0.004061352008 |
| CEACAM1 |  |  |  |  |  |  |  |  |  |  |  |  |  |  |  |  |  | 0.4128710536 | 0.3839978681 |  |  |  |  |  |  |  |  |  |  |  |  |  |
| FCGR1A |  |  |  |  |  |  |  |  |  |  |  |  |  |  |  |  |  | -0.04145398851 | -0.1043443946 |  |  |  |  |  |  |  |  |  |  |  |  |  |
| FCGR3B |  |  |  |  |  |  |  |  |  |  |  |  |  |  |  |  |  | -0.4328714919 | -0.4552305899 |  |  |  |  |  |  |  |  |  |  |  |  |  |
| NTSR1 | 0.5048248152 |  |  |  | 0.0084885567 | -0.1693345912 | -0.0277649839 | -0.539128569 | 0.27757808 | -0.4520760702 | 0.0583426838 | -0.1704067424 | 0.5659388501 | -0.0145299738 | -0.4430637959 | 0.0130732179 | 0.097409007 | -0.04266608217 | -0.0657715367 |  | -0.1437373464 | 0.06943038279 | -0.1861428506 | 0.176649683 | 0.2607806 | 0.5358072667 | 0.2249894886 | 0.4110831698 | 0.1260384873 | 0.1309629198 | 0.516406135 | 0.2961065286 |
| CCNE1 | -0.4484446468 |  |  |  | 0.0830569428 | -0.4896733227 | 0.0235328208 | -0.4604575975 | -0.058393742 | -0.3344131113 | 0.1592780824 | 0.3584747795 | 0.3632842174 | 0.4215148087 | -0.6873845237 | 0.1600738035 | -0.3105887755 | 0.08963321804 | -0.0149906701 | -0.5286991853 | 0.3946972311 | -0.1410736625 | 0.5807070756 | 0.7964771484 | 0.7276717626 | 0.5399218234 | -0.6498491313 | -0.6349816859 | -0.4925153742 | -0.7647665003 | -0.7203925276 | -0.6313052585 |
| CARM1 | -0.3910072276 |  |  |  | 0.2859017761 | -0.3921860803 | 0.1709120533 | -0.1075699621 | 0.4349721749 | -0.262016011 | 0.1321294952 | -0.700517185 | 0.5717981685 | 0.6075116451 | -0.2391561228 | -0.1568009878 | -0.3969718789 |  |  | -0.0950407611 |  |  |  |  |  |  |  |  |  |  |  |  |

Supplementary Data 6

| Gene | Positive Regulation | Negative Regulation | Total Differential |
| --- | --- | --- | --- |
| POLD1 | 1 | 11 | 12 |
| ACSS1 | 1 | 8 | 9 |
| CBR1 | 1 | 7 | 8 |
| PDXDC1 | 1 | 7 | 8 |
| CD2 | 2 | 10 | 12 |
| CLK4 | 2 | 7 | 9 |
| RXRB | 2 | 7 | 9 |
| ZAP70 | 3 | 8 | 11 |
| RTCB | 3 | 8 | 11 |
| IDH3B | 3 | 8 | 11 |
| CD79B | 5 | 12 | 17 |
| ITK | 4 | 9 | 13 |
| SLC1A5 | 4 | 8 | 12 |
| HSD17B10 | 3 | 6 | 9 |
| FARSB | 6 | 11 | 17 |
| ACSL1 | 5 | 9 | 14 |
| MAP4K2 | 5 | 9 | 14 |
| CD1D | 5 | 9 | 14 |
| PML | 6 | 10 | 16 |
| GOT1 | 3 | 5 | 8 |
| STK17B | 7 | 11 | 18 |
| FECH | 8 | 12 | 20 |
| CD3G | 6 | 9 | 15 |
| DDX39B | 4 | 6 | 10 |
| NDUFS1 | 4 | 6 | 10 |
| TEC | 7 | 10 | 17 |
| STIP1 | 5 | 7 | 12 |
| CSNK1G2 | 5 | 7 | 12 |
| ME2 | 5 | 7 | 12 |
| AIFM1 | 5 | 7 | 12 |
| LGALS2 | 5 | 7 | 12 |
| RPS5 | 6 | 8 | 14 |
| HINT1 | 6 | 8 | 14 |
| MALT1 | 6 | 8 | 14 |
| CACNG5 | 7 | 9 | 16 |
| CCS | 4 | 5 | 9 |
| ALAS2 | 9 | 11 | 20 |
| CYC1 | 5 | 6 | 11 |
| SIRT2 | 6 | 7 | 13 |
| PSME1 | 6 | 7 | 13 |
| ARPC2 | 6 | 7 | 13 |
| AKT2 | 6 | 7 | 13 |
| PFN1 | 6 | 7 | 13 |
| CASP1 | 6 | 7 | 13 |
| S100A1 | 6 | 7 | 13 |
| PTDSS1 | 6 | 7 | 13 |
| CCNA2 | 7 | 8 | 15 |
| ISG20 | 7 | 8 | 15 |
| FLI1 | 7 | 8 | 15 |
| IFNGR1 | 8 | 9 | 17 |
| EPHB6 | 8 | 9 | 17 |
| TBXAS1 | 9 | 10 | 19 |
| RAMP1 | 9 | 10 | 19 |
| SELL | 9 | 10 | 19 |
| IFNAR2 | 10 | 10 | 20 |
| WAS | 9 | 9 | 18 |
| PEF1 | 7 | 7 | 14 |
| ABCB4 | 7 | 7 | 14 |
| TPI1 | 7 | 7 | 14 |
| LPAR2 | 7 | 7 | 14 |
| HCAR2 | 6 | 6 | 12 |
| LDHA | 6 | 6 | 12 |
| SLC20A1 | 6 | 6 | 12 |
| CTSD | 6 | 6 | 12 |
| SLC31A2 | 6 | 6 | 12 |
| FCER1G | 6 | 6 | 12 |
| PAK4 | 6 | 6 | 12 |
| GSK3A | 6 | 6 | 12 |
| EEF1A1 | 6 | 6 | 12 |
| CFL1 | 6 | 6 | 12 |
| CHP1 | 6 | 6 | 12 |
| FGF1 | 6 | 6 | 12 |
| BAG1 | 6 | 6 | 12 |
| AKR1B1 | 6 | 6 | 12 |
| NFKB1 | 6 | 6 | 12 |
| CXCR4 | 6 | 6 | 12 |
| TUBB2A | 6 | 6 | 12 |
| DHPS | 6 | 6 | 12 |
| COX6B1 | 6 | 6 | 12 |
| COX4I1 | 6 | 6 | 12 |
| UBB | 6 | 6 | 12 |
| ALDH2 | 6 | 6 | 12 |
| VKORC1 | 6 | 6 | 12 |
| HRH3 | 6 | 6 | 12 |
| SLC31A1 | 6 | 6 | 12 |
| SLC10A1 | 6 | 6 | 12 |
| SLCO1C1 | 6 | 6 | 12 |
| SMS | 6 | 6 | 12 |
| MAT2A | 6 | 6 | 12 |
| HLA-DQB1 | 6 | 6 | 12 |
| UCP2 | 6 | 6 | 12 |
| IL6R | 5 | 5 | 10 |
| CCR9 | 5 | 5 | 10 |
| TRPM2 | 5 | 5 | 10 |
| GP9 | 10 | 9 | 19 |
| C1QB | 10 | 9 | 19 |
| CTSH | 10 | 9 | 19 |
| P2RY12 | 10 | 9 | 19 |
| G6PD | 9 | 8 | 17 |
| RDH12 | 9 | 8 | 17 |
| INPP5D | 9 | 8 | 17 |
| RPS9 | 9 | 8 | 17 |
| SNCA | 9 | 8 | 17 |
| SYK | 9 | 8 | 17 |
| CRABP1 | 9 | 8 | 17 |
| NDUFS4 | 9 | 8 | 17 |
| SERPINE1 | 9 | 8 | 17 |
| MAPK12 | 8 | 7 | 15 |
| GSTO1 | 8 | 7 | 15 |
| TRAPPC5 | 8 | 7 | 15 |
| TNFRSF11A | 8 | 7 | 15 |
| RPL15 | 8 | 7 | 15 |
| PANX1 | 8 | 7 | 15 |
| NDUFB7 | 8 | 7 | 15 |
| STK33 | 7 | 6 | 13 |
| CLIC1 | 7 | 6 | 13 |
| CACNB2 | 7 | 6 | 13 |
| KCND3 | 7 | 6 | 13 |
| GLIPR1 | 7 | 6 | 13 |
| F7 | 7 | 6 | 13 |
| CDC42 | 7 | 6 | 13 |
| NPR2 | 7 | 6 | 13 |
| KCNJ9 | 7 | 6 | 13 |
| MTR | 7 | 6 | 13 |
| CD52 | 7 | 6 | 13 |
| APOE | 12 | 10 | 22 |
| F10 | 12 | 10 | 22 |
| MT2A | 6 | 5 | 11 |
| ADORA2A | 6 | 5 | 11 |
| LYZ | 6 | 5 | 11 |
| BDH1 | 6 | 5 | 11 |
| HCK | 11 | 9 | 20 |
| S100A6 | 10 | 8 | 18 |
| CA14 | 10 | 8 | 18 |
| RAC2 | 10 | 8 | 18 |
| PTPN6 | 10 | 8 | 18 |
| NAAA | 5 | 4 | 9 |
| FGR | 5 | 4 | 9 |
| UNG | 9 | 7 | 16 |
| ACTB | 9 | 7 | 16 |
| PPP2R5C | 9 | 7 | 16 |
| LSM6 | 9 | 7 | 16 |
| SDHB | 9 | 7 | 16 |
| SLC12A6 | 9 | 7 | 16 |
| TCN2 | 9 | 7 | 16 |
| CALR | 9 | 7 | 16 |
| PI4KA | 9 | 7 | 16 |
| CSF3R | 12 | 9 | 21 |
| HDC | 12 | 9 | 21 |
| SYTL4 | 12 | 9 | 21 |
| RPL14 | 8 | 6 | 14 |
| DMD | 8 | 6 | 14 |
| BAD | 8 | 6 | 14 |
| PAK5 | 8 | 6 | 14 |
| ELOVL5 | 8 | 6 | 14 |
| APOD | 8 | 6 | 14 |
| PSMB1 | 8 | 6 | 14 |
| JAK1 | 8 | 6 | 14 |
| PRKCD | 8 | 6 | 14 |
| MDH2 | 8 | 6 | 14 |
| MMP27 | 8 | 6 | 14 |
| KCNQ2 | 8 | 6 | 14 |
| NDUFS6 | 8 | 6 | 14 |
| NDUFA5 | 8 | 6 | 14 |
| NAGPA | 8 | 6 | 14 |
| CEBPB | 7 | 5 | 12 |
| APOA2 | 7 | 5 | 12 |
| CYB5R1 | 7 | 5 | 12 |
| HMMR | 10 | 7 | 17 |
| RAB8B | 10 | 7 | 17 |
| RRM2 | 10 | 7 | 17 |
| SMOX | 12 | 8 | 20 |
| PRKCI | 12 | 8 | 20 |
| S100A8 | 9 | 6 | 15 |
| FPR1 | 9 | 6 | 15 |
| NCF1 | 9 | 6 | 15 |
| PCMT1 | 9 | 6 | 15 |
| GMPS | 9 | 6 | 15 |
| VIM | 9 | 6 | 15 |
| KRT9 | 9 | 6 | 15 |
| NISCH | 9 | 6 | 15 |
| PSMB7 | 9 | 6 | 15 |
| GBA | 9 | 6 | 15 |
| ACACB | 9 | 6 | 15 |
| AR | 9 | 6 | 15 |
| ST3GAL5 | 9 | 6 | 15 |
| CASP7 | 9 | 6 | 15 |
| ADAM9 | 9 | 6 | 15 |
| ITGAL | 9 | 6 | 15 |
| TH | 9 | 6 | 15 |
| DNTT | 9 | 6 | 15 |
| GRIN2B | 9 | 6 | 15 |
| TGM5 | 9 | 6 | 15 |
| SLC25A15 | 9 | 6 | 15 |
| SLC5A7 | 9 | 6 | 15 |
| RASSF9 | 9 | 6 | 15 |
| HPR | 6 | 4 | 10 |
| CDK4 | 6 | 4 | 10 |
| CDK8 | 6 | 4 | 10 |
| IL1B | 14 | 9 | 23 |
| ALOX5 | 11 | 7 | 18 |
| PFKFB4 | 11 | 7 | 18 |
| TSPO | 11 | 7 | 18 |
| CLU | 11 | 7 | 18 |
| TUBB1 | 11 | 7 | 18 |
| GSR | 11 | 7 | 18 |
| PTGER4 | 11 | 7 | 18 |
| SORD | 11 | 7 | 18 |
| CYBB | 11 | 7 | 18 |
| CYBA | 11 | 7 | 18 |
| NDUFB9 | 11 | 7 | 18 |
| C3 | 8 | 5 | 13 |
| ANXA2 | 10 | 6 | 16 |
| LGALS3 | 10 | 6 | 16 |
| NAPEPLD | 10 | 6 | 16 |
| TAGLN2 | 10 | 6 | 16 |
| RAB5A | 10 | 6 | 16 |
| RAN | 10 | 6 | 16 |
| CDK15 | 10 | 6 | 16 |
| CANX | 10 | 6 | 16 |
| TSHR | 10 | 6 | 16 |
| CBFB | 10 | 6 | 16 |
| CSTB | 12 | 7 | 19 |
| CKS1B | 12 | 7 | 19 |
| ALOX12 | 12 | 7 | 19 |
| TRAPPC4 | 7 | 4 | 11 |
| UQCRB | 7 | 4 | 11 |
| TACR2 | 7 | 4 | 11 |
| GATB | 7 | 4 | 11 |
| MAP4K4 | 9 | 5 | 14 |
| ALB | 9 | 5 | 14 |
| PORCN | 9 | 5 | 14 |
| ABCC9 | 11 | 6 | 17 |
| RALB | 11 | 6 | 17 |
| APOC2 | 14 | 7 | 21 |
| DNMT3A | 12 | 6 | 18 |
| MCL1 | 12 | 6 | 18 |
| BMP4 | 12 | 6 | 18 |
| GLUL | 10 | 5 | 15 |
| OAS2 | 10 | 5 | 15 |
| ANXA1 | 8 | 4 | 12 |
| NDUFS5 | 8 | 4 | 12 |
| GSTM1 | 6 | 3 | 9 |
| PRKAA1 | 6 | 3 | 9 |
| LRG1 | 13 | 6 | 19 |
| DSTN | 13 | 6 | 19 |
| MOCOS | 11 | 5 | 16 |
| SERPINF1 | 9 | 4 | 13 |
| VWF | 14 | 6 | 20 |
| NFKBIA | 14 | 6 | 20 |
| PAH | 10 | 4 | 14 |
| PLTP | 11 | 4 | 15 |
| TF | 14 | 5 | 19 |
| PTPN1 | 9 | 3 | 12 |
| COL1A2 | 9 | 3 | 12 |
| GSTZ1 | 6 | 2 | 8 |
| KIF2C | 6 | 2 | 8 |
| CTSV | 6 | 2 | 8 |
| APOBR | 14 | 4 | 18 |
| LOX | 7 | 2 | 9 |
| MMP8 | 7 | 2 | 9 |
| EGLN3 | 7 | 2 | 9 |
| MELK | 12 | 3 | 15 |
| SLC25A12 | 8 | 2 | 10 |
| NNMT | 8 | 2 | 10 |
| COL3A1 | 8 | 2 | 10 |
| LUM | 9 | 2 | 11 |
| DNM2 | 9 | 2 | 11 |
| CKB | 11 | 2 | 13 |
| AURKA | 7 | 1 | 8 |
| GCSH | 7 | 1 | 8 |
| DTYMK | 8 | 1 | 9 |
| NT5E | 8 | 1 | 9 |
| CDH2 | 8 | 1 | 9 |
| BRD4 | 8 | 1 | 9 |
| SLC7A7 | 9 | 1 | 10 |
| TLR2 | 9 | 1 | 10 |
| IGFBP6 | 9 | 1 | 10 |
| MYD88 | 9 | 1 | 10 |
| FYN | 10 | 1 | 11 |
| SLC2A6 | 10 | 1 | 11 |
| SCD | 11 | 1 | 12 |
| IL1RN | 12 | 1 | 13 |
| MPL | 17 | 0 | 17 |
| CDKN1A | 14 | 0 | 14 |
| PLA2G7 | 14 | 0 | 14 |
| TFPI | 12 | 0 | 12 |
| TREM1 | 11 | 0 | 11 |
| P2RY13 | 10 | 0 | 10 |
| MMP9 | 10 | 0 | 10 |
| IGF1R | 9 | 0 | 9 |
| PYGM | 8 | 0 | 8 |
| KCNJ5 | 8 | 0 | 8 |
